# Lewis Acid Catalyzed Dual Strain-Release Platform for Transforming Azabicyclo[1.1.0]butanes into Functionalized Azetidines with Donor–Acceptor(D-A) Cyclopropanes and Bicyclo[1.1.0]butanes

**DOI:** 10.1021/acs.orglett.6c00880

**Published:** 2026-03-26

**Authors:** Subhadeep Hazra, Manveer Patel, Soumik Mondal, Rezwan Ahmed, Pujan Sasmal, Swati De, Jaideep Saha

**Affiliations:** † Department of Medicinal Chemistry, National Institute of Pharmaceutical Education and Research (NIPER), Mohali-160062, India; ‡ Department of Biological and Synthetic Chemistry, Centre of Biomedical Research, Lucknow 226014, UP, India; § 30132 University of Kalyani, West Bengal 741235, India

## Abstract

Herein, we disclose a catalytic dual strain-release approach that achieves orthogonal N1 and C3 functionalization of azabicyclo[1.1.0]­butanes (ABBs) through synergistic engagement with donor–acceptor cyclopropanes (DACs). Orthogonal Lewis acid polarization of the cyclopropane relays activation to ABB, permitting access to functionalized azetidines, through cleavage of the strained C–N bond of ABB. Extending this activation paradigm to bicyclo[1.1.0]­butanes (BCBs) further demonstrates the generality of this concept.

## Graphical abstract

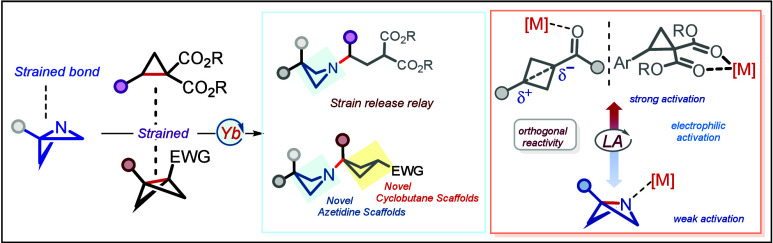

Nitrogen-containing saturated heterocycles are ubiquitous in bioactive molecules, and the presence of piperidine and pyrrolidine rings is widespread in the context.^1^ Recently, azetidines have gained significant prominence in drug discovery owing to their optimal polarity, basicity, and conformational rigidity, which enhance metabolic stability and pharmacokinetic profiles.^2,3^ This trend is exemplified by marketed drugs, such as baricitinib and azelndipine, where 1,3-disubstituted azetidines are frequently encountered (Scheme 1a). However, the development of broadly applicable methods to access diversely functionalized azetidines remain limited.^4^ Recent strain-release strategies, particularly involving azabicyclo[1.1.0]­butane (ABB), have opened promising avenues for accessing functionalized azetidines relevant to drug discovery.^5–7^ ABB featuring a highly strained internal N–C3 bond offers versatility as a precursor for N1/C3-substituted azetidines via strain-release reactivity (Scheme 1b). Due to the inherent electrophilicity of the C3 center, strong nucleophiles such as turboamides, Grignard reagents, thiols, and organocuprates could be directly added to ABB without a *N*-activation. Subsequent *N*-functionalization were followed through transformations such as acylation and sulfonylation, or, in selective cases, via S_N_Ar, Buchwald-Hartwig coupling.^5^ Beyond these stepwise approaches, tandem strain-release processes have been developed, put forth by the Aggarwal group, enabling simultaneous C3 elaboration and *N*-functionalization from tailored ABB carbinols.^7^ While reports represent a significant step forward to drugs like azetidines, still the scope for the incorporation of elaborate functional groups on nitrogen was limited.

**Scheme 1:**
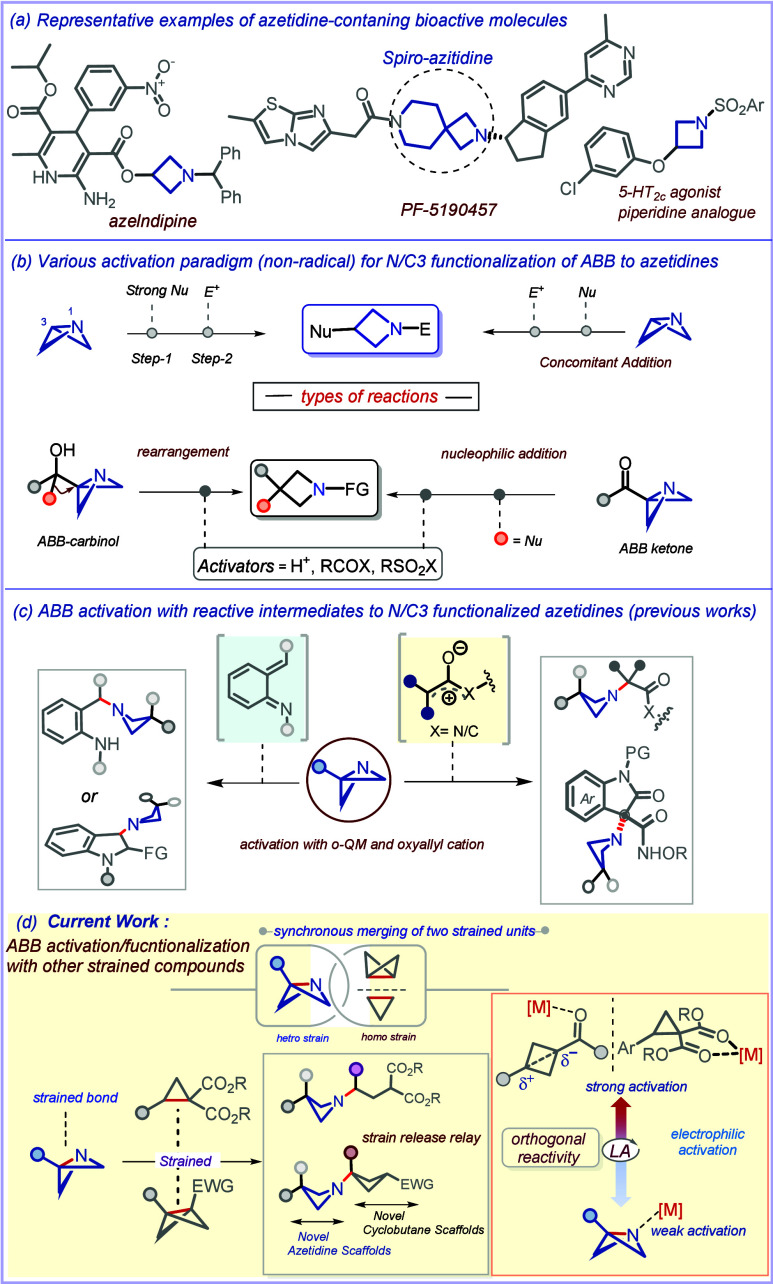
(a–c) Azetidines in Bioactive Scaffolds, Strain-Release-Driven Functionalization of Azabicyclo[1.1.0]­butanes to Azetidines, and (d) the Present Strain-Merged Strategy

We recently expanded the electrophilic activation manifold of azabicyclo[1.1.0]­butane (ABB) to include reactive intermediates such as (oxy)­allyl and aza­(oxy)­allyl cations, enabling access to elaborately *N*-functionalized azetidines.^8a^ Additionally, we also demonstrated tandem N1/C3 functionalization of ABB using aza-*ortho*-quinone methides (aza-o-QMs) (Scheme 1c).^8b^ Building on our continued interest in developing tandem ABB functionalization, we questioned whether another strain ring system could be employed as potential activator for ABB, subsequently leading into elusive *N*-functionalization. To this end, we focused our attention to donor–acceptor cyclopropanes (DACs), which have been widely employed as versatile synthons in organic synthesis.^9^ However, DACs are inherently weak electrophiles and therefore, we envisaged that they most likely would not impart any activation for the *N*-center of ABB. Activation of DACs with Lewis/Brønsted acid is known to polarize the former into an effective electrophile.^10^ Logically through, such strategy is required be orthogonal for ABB, meaning that the activation process of DAC should not affect or promote any activation of ABB, otherwise rendering futile outcome. A second important question was whether the electrophilicity of DAC would suffice to activate the tertiary nitrogen of the ABB system. Herein, we disclose an unprecedented merger of two electronically distinct strained systems, enabling sequential strain-release and a new mode of tandem N1/C3 functionalization of ABB (Scheme 1d). Notably, this activation paradigm is extendable to bicyclo[1.1.0]­butanes (BCBs), further expanding the scope of azetidine *N*-functionalization using nonclassical electrophiles.^11,12^

We initiated our studies using a model DAC **1a** and ABB-carbinol **2a**, and systematically screened conditions to identify an orthogonal Lewis acid activator and other parameters for our desired manifold. Gratifyingly, Yb­(OTf)_3_ (30 mol %) in dichloroethane (DCE) emerged as the optimal conditions, delivering the desired product **3** in 96% yield (Table 1, entry 1). Formation of **3** was supported by the appearance of a benzylic proton at δ 3.24 and an active methylene signal at δ 3.14, consistent with a DAC ring opening. Additionally, two methylene resonances at δ 3.82–3.79 and 3.64–3.62 ppm corroborated azetidine formation. Some observations were noteworthy; (i) lower catalyst loading led to diminished efficiency (Table 1, entry 2); (ii) several commonly used Lewis acid for DACs, including Sc­(OTf)_3_, Cu­(OTf)_2_, MgI_2_, In­(OTf)_3_, and Sn­(OTf)_2_ (20 mol %) were futile, largely due to competitive ABB activation (Table 1, entry 3); (iii) BF_3_·Et_2_O failed to promote the reaction (Table 1, entry 4); (iv) nickel perchlorate was moderately effective, suggesting only weak coordination to ABB nitrogen (Table 1, entry 5); (v) increased loading of Yb­(OTf)_3_ [10 to 30%] or elevated temperature [50 to 80 °C] improved reaction yield (see the Supporting Information (SI)); (vi) solvents such as MeCN, THF, and HFIP gave comparable yields to DCE, whereas toluene, DMF, and MeOH were futile (Table 1, entry 6; see the SI); and (vii) without Lewis acid, no reaction proceeded (Table 1, entry 7).

**Table 1:**
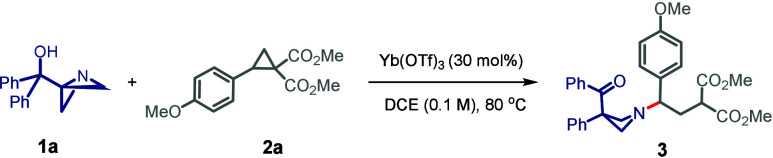
Optimization of the Reaction Conditions

entry	deviation from the standard condition	yield^b^
1​	none	96%
2​	10 mol % Yb(OTf)_3_	68%
3​^c^	other [M]-cat. (20 mol %)	trace–55%
4​	BF_3_·Et_2_O	trace
5​	NiClO_4_·6H_2_O	52%
6​^d^	other solvent	63%–76%
7​	no catalyst	NR

a Reaction conditions: Compound **1a** (1.0 equiv), Compound **2a** (1.0 equiv), Yb­(OTf)_3_ (30 mol %), DCE (0.1 M), 12 h, 80 °C.

b Yields of the isolated product.

c Other­[M]-cat: Sc­(OTf)_3_ [23%], Cu­(OTf)_2_ [35%], MgI_2_ [trace], Sn­(OTf)_2_ [15%], In­(OTf)_3_ [30%], Bi­(OTf)_3_[trace].

d Solvent: DMF [trace], THF [63%], toluene [trace], ACN [70%], HFIP [68%], MeOH [NR].

With the optimized reaction conditions in hand, we next explored the substrate scope of the transformation (Scheme 2). Variation of DACs was explored first. Aryl-donor residues on DACs bearing electron-donating group(s), such as alkyl or alkoxy, at the *para*- and *meta*-positions furnished the corresponding functionalized azetidines products in excellent yields (**3**–**10**). More sterically demanding *ortho*-substitution on the ring also afforded the smooth formation of the desired product (**11**). Similarly, halogen substituents at the *para*-position on the aryl ring delivered excellent yield (**12** and **13**). However, the presence of strong electron-withdrawing groups such as −CN or −NO_2_ completely suppressed the reactivity (**14** and **15**). DACs having heteroaryl motifs at a donor site such as 2-furyl, 2-thienyl, 3-indolyl (**16**–**18**) or an extended π-system such as the 2-naphthyl group (**19**), delivered the azetidines in good to excellent yields. These entries exemplify the scope to incorporate broad range aromatic residues placed at the nitrogen center of resulting azetidines. Vinyl and alkenyl-substituted DACs, which are electronically distinct from earlier examples were also smoothly accommodated with no competitive formation of isomerized product via homoconjugate addition of the ABB to DAC (**20** and **21**).

**Scheme 2:**
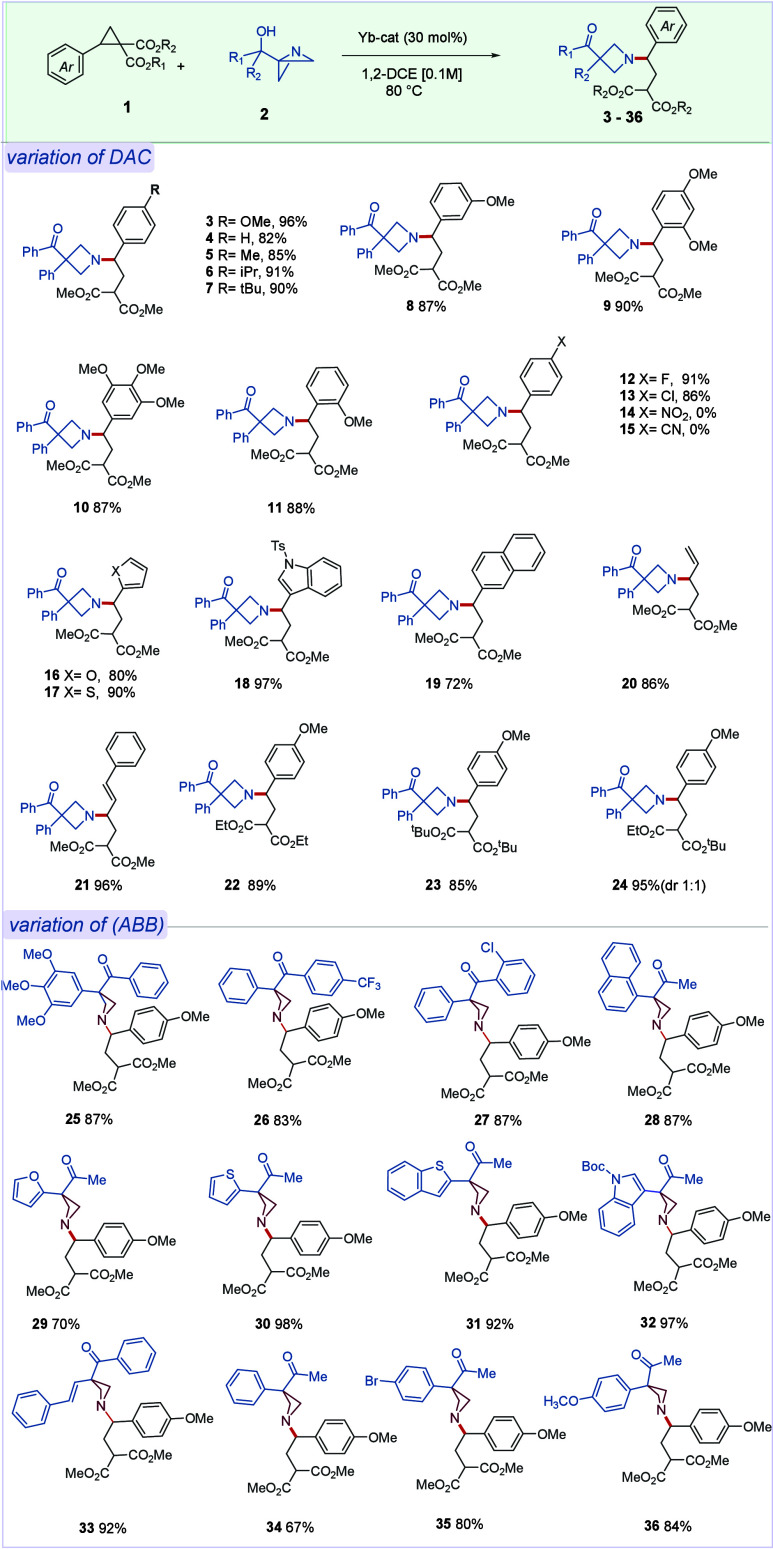
Scope of the Transformation with Various DAC and ABB Derivatives

a Reaction conditions: compound **1** (1.0 equiv 0.1 mmol), compound **2** (1.0 equiv, 0.1 mmol), Yb­(OTf)_3_ (30 mol %), DCE (0.1 M), 12 h, 80 °C (oil bath), N_2_ atmosphere.

Next, we examined the variation in the ester moiety; this change revealed no significant influence on the reaction efficiency (**22**–**24**). To evaluate how varied combination of acceptor groups on DAC would participate in the electronic polarization of DACs and subsequently into the ABB activation process, geminally substituted ketoester, diketone, nitroesters, cyanoesters, and malononitriles (not shown; see the SI) were assessed. These entries rendered unsuccessful reaction; either an intractable mixture or significant recovery of starting materials were obtained. These results collectively suggest that the DACs with diester motif composite an ideal system for imparting optimal activation of DAC with Yb-catalyst and a relayed ABB activation. Having examined the scope for DACs, we proceeded to examine various ABB derivatives (Scheme 2; **25**–**36**). On the ABB carbinols, we first varied the aryl residues at the carbinol portion. Both electron-donating and electron-withdrawing substituents on the aryl ring appeared well-tolerated, and corresponding products were obtained in consistently high yields. For those entries with two different aryl residues, the migration of more electron-rich aryl group to C3 proceeded with high selectivity (**25**–**27**). Carbinols bearing extended π-systems (naphthyl) or heteroaryl residues (thiophene, furan, benzothiophene, and indole) also underwent smooth conversion to the desired azetidines (**28**–**32**) in excellent efficiency. Chalcone-derived ABB-carbinol was an example that delivered C3-alkenylated azetidine **33**, with selective alkenyl migration.

In our previous studies, we noticed that the cycloalkanol segment in an ABB carbinol differs in reactivity and rarely involves Semipinacol rearrangement at C3 as opposed to their (di)­aryl counterpart. Curiously when we employed a range of ABB cycloalkanols of varying size and complexity, the relay of activation from DAC to the nitrogen of ABB delivered the 3-spiroepoxyazetidine in good to excellent yield with no detectable Semipinacol-rearrangement product (Scheme 3a, **37**–**41**). Evidently, DAC-mediated activation of ABB was sufficient to engage the intramolecular cyclization of the carbinol −OH group to C3 center, forming a spiro-epoxide motif, and importantly, such process alleviates the needs of exogenous nucleophile to promote similar cyclization.^7b^ Importantly, benzylic ABB-cycloalkanols underwent ring-expansion through an intramolecular Semipinacol-rearrangement process, leading to spirocyclic azetidines. These certainly illustrate a different reactivity trait from the all-alkyl counterparts (Scheme 3b; **42**–**44**). These entries also indicate the possibility of diversification at C3 while adding to a broad range of functional groups, previously unexplored, at the N1 center of the resulting azetidines, through current *N*-activation/functionalization paradigm.

**Scheme 3:**
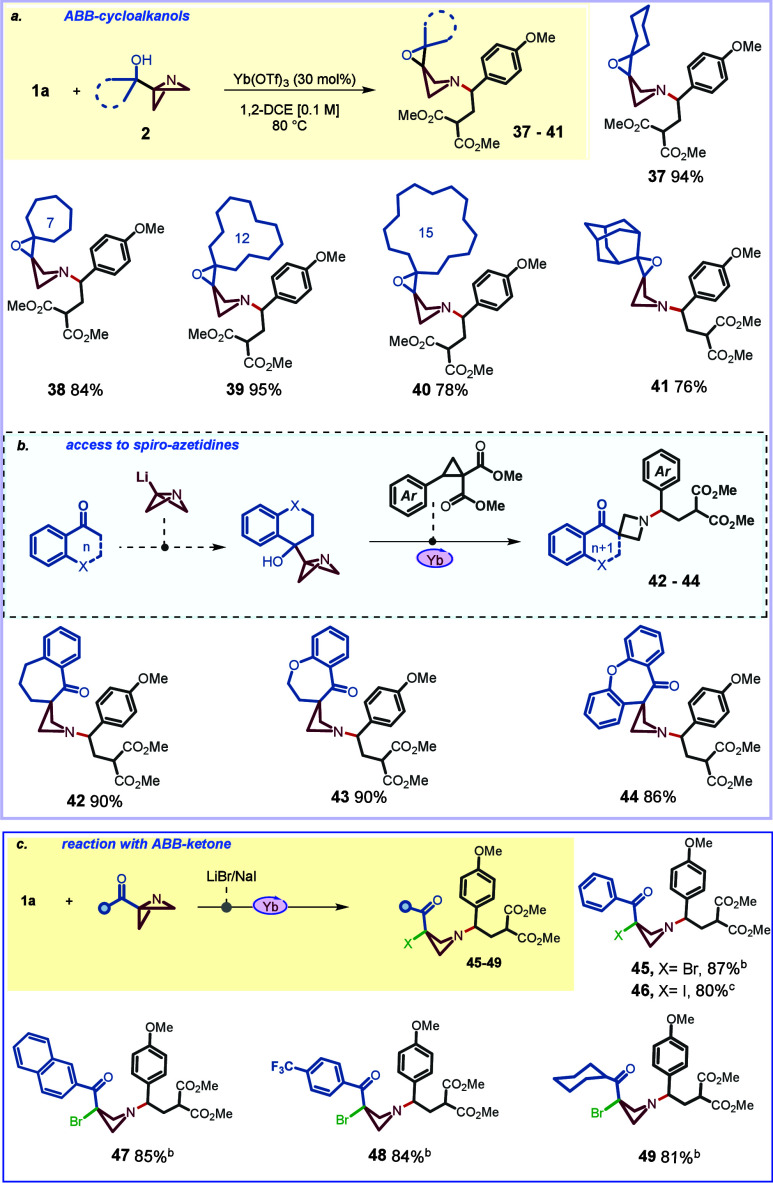
Scope of ABB Cycloalkanols and ABB-Keto with DAC

a Reaction conditions: ABB (1.0 equiv 0.1 mmol), DAC (1.0 equiv, 0.1 mmol), Yb­(OTf)_3_ (30 mol %), DCE (0.1 M), 80 °C. ^*b*^ABB-ketones (1.0 equiv 0.1 mmol), DACs (1.0 equiv, 0.1 mmol), Yb­(OTf)_3_ (30 mol %), DCE (0.1 M), LiBr (5.0 equiv), 80 °C. *^c^* Instead of LiBr, NaI (5.0 equiv) was used.

Having studied the reactivity of ABB-carbinols, we next investigated the transformation of ABB-derived alkyl/aryl ketones (Scheme 3c). Unlike ABB carbinols, these substrates lack an internal nucleophilic residue at C3, and therefore, an exogenous nucleophile is needed for the strain release. Gratifyingly, the addition of LiBr or NaI enabled smooth conversion, affording the corresponding haloketones (**45**–**49**) in synthetically useful yields. Enabling the inclusion of this class of ABB under current orthogonal activation further broadens the scope of the process.

At this juncture, we questioned whether an activated bicyclo[1.1.0]­butanes (BCBs) could similarly and orthogonally be activated with catalytic Lewis acid and the activation could be relayed synchronously to ABB. Of note, while a BCB contains more ring strain than the corresponding DAC system, notably, these do not parallel in reactivity for all their reaction partners.^11c^ Additionally, DA-BCB contains a single electron-withdrawing group and thus features a stark difference in electronics from that of DAC systems that we used in the present study. Of note, the diester motif on the DA-cyclopropanes can effectively form a chelate with the metal center, facilitating the activation, which however differs in the BCBs. At the outset of this exploration, when the phenyl-substituted BCB ester (**1f′′**) was used with ABB carbinol **2a**, the reaction failed to deliver the desired azetidine (**50**, Scheme 4). Use of the BCB containing an acyl pyrazole group as electron-withdrawing segment also failed to engage productively in the current transformation (**51**).^11d^ To our delight, phenyl-substituted BCB with a ketone at acceptor-end (**1a′′**) could match to the desired reactivity and orthogonality for BCB activation relayed to ABB activation. Altogether, this class of BCB closely mirrored the features of transformation to those of DACs, albeit elevated temperature was not needed in the current class (cf. Schemes 2 and 3). Thus, *N*-cyclobutyl azetidine **52** was obtained through the course of the reaction in 88% yield (diastereomeric ratio (dr) = 85:15) using ABB **2a** (Scheme 4). Subsequently, a series of ABB carbinols bearing various alkyl/aryl or heteroaryl **52**–**54**, diaryl (**55**–**58**) residues were tested, which successfully led to *N*-cyclobutylated azetidines in high yield. Entry **59** showcases a cyclic variant of diaryl ABB carbinol leading into a spiro-derivative.

**Scheme 4:**
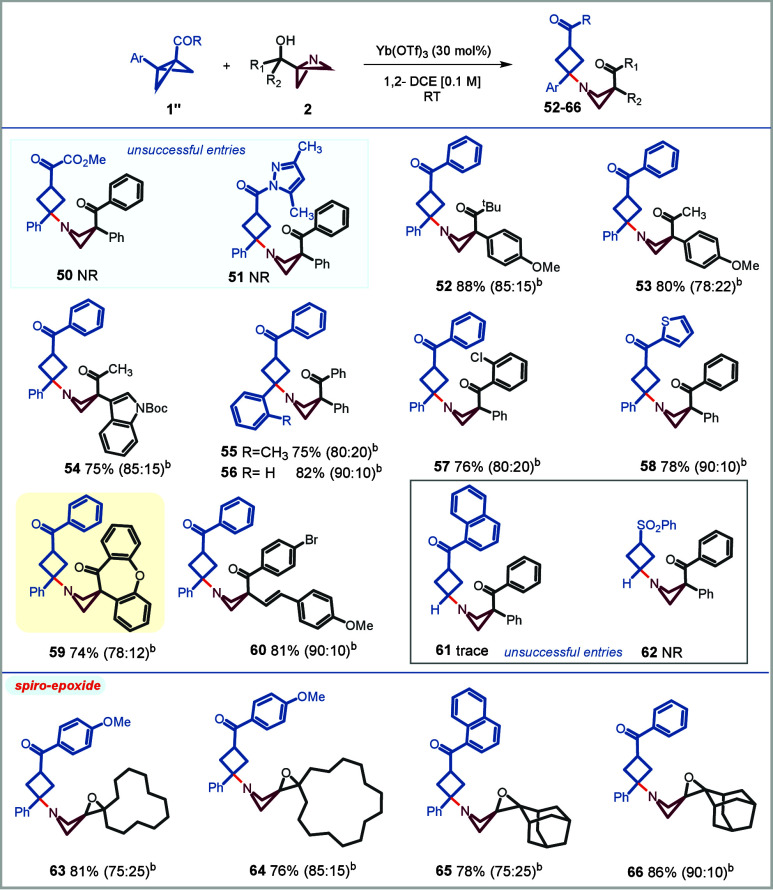
Scope of ABB Carbinols with Bicyclo[1.1.0] Butanes (BCBs)

a Reaction conditions: ABB (1.0 equiv 0.1 mmol), BCBs (1.0 equiv, 0.1 mmol), Yb­(OTf)_3_ (30 mol %), DCE (0.1 M) at RT. *^b^* The dr ratio was determined by ^1^H NMR of crude mixture (see the SI).

Use of the ABB carbinols which were successful with DACs, such as aryl/alkenyl ABB carbinol (**60**), reacted with parallel efficiency. Of note that, BCB ketone or sulfonyl that lacks the donor group (aromatic) on its structure did not engage in the desired transformation (**61** and **62**). The ABB cycloalkanols could also be efficiently engaged in the reaction with BCB, affording the corresponding azetidine spiro-epoxide as a mixture of diastereomers in high yields (**63**–**66**).

Based on the control experiments and other experimental observations (see the SI, Figure S4) a mechanism for the developed transformation is proposed in Scheme 5. First, activation of DAC with Yb­(OTf)_3_ forms a polarized electrophilic species, and the benzylic carbon is attacked by the nitrogen of ABB, with a synchronous rearrangement at C3 and strain release via cleavage of the central C–N bond leading to intermediate **I**. The possibility of ABB transforming into the free azetidine via Semipinacol rearrangement and adding to activated DAC can only be attributed to a minor pathway based on control experiments (see the SI). Dissociation of the metal complex from **II** regenerates the active catalyst and affords the formation of product **3**.

**Scheme 5:**
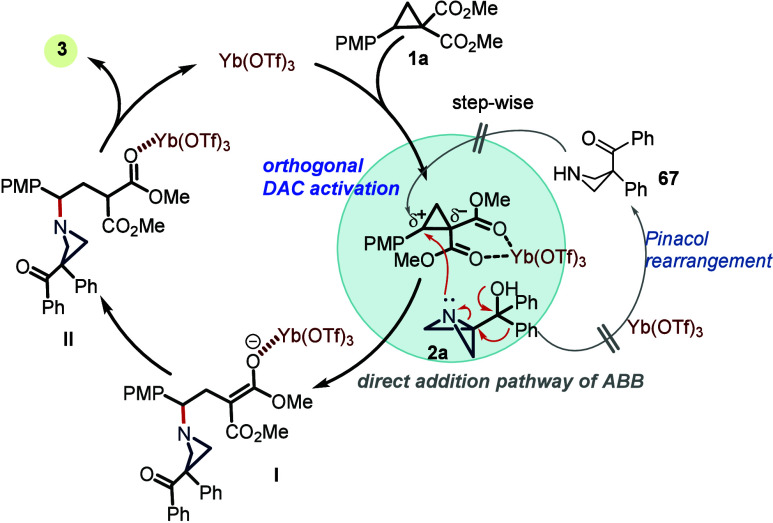
Proposed Mechanism

In summary, we report a conceptually distinct strain-merged activation strategy enabling tandem N1/C3 functionalization of azabicyclo[1.1.0]­butanes through orthogonal engagement of two electronically disparate strained systems. Selective Lewis acid activation of D–A cyclopropanes generates a polarized electrophile that relays activation to the ABB nitrogen, triggering concerted C–N bond cleavage and C3 reorganization. This dual strain-release process affords densely functionalized azetidines with a broad scope, high functional group tolerance, and excellent efficiency under mild conditions. Importantly, the strategy is extendable to bicyclo[1.1.0]­butanes, enabling *N*-cyclobutylation via nonclassical electrophiles. Mechanistic studies support a concerted pathway, featuring orthogonal activation–strain release and electrophilic polarization as crucial pillars for current transformation. Overall, this work establishes a strong platform for constructing a medicinally relevant azetidine architecture.

## Supplementary Material



## Data Availability

The data underlying this study are available in the published article and its Supporting Information.

## Notes

General experimental, methods, experimental data and copies of ^1^H, ^13^C NMR spectra of the products (PDF)

## ∇

For J. Saha: Molecular Design and Synthesis Unit, Rutgers, the State University of New Jersey, Piscataway, New Jersey 08854, USA
