# Supplementary material for: Lewis Acid Catalyzed Dual Strain-Release Platform for Transforming Azabicyclo[1.1.0]butanes into Functionalized Azetidines with Donor–Acceptor(D-A) Cyclopropanes and Bicyclo[1.1.0]butanes
Source: Org Lett. 2026 Mar 26;28(14):4554–9. doi: 10.1021/acs.orglett.6c00880 (PMC13077690; doi:10.1021/acs.orglett.6c00880)
Supplement: Supplementary file 1 [file ol6c00880_si_001.pdf]

Supporting Information

Lewis Acid Catalyzed Dual Strain-Release Platform for Transforming Azabicyclo[1.1.0]butanes into Functionalized Azetidines with Donor–Acceptor(D-A) Cyclopropanes and Bicyclo[1.1.0]butanes

Subhadeep Hazra,[ac] Manveer Patel,[b] Soumik Mondal,[b]# Rezwan Ahmed,[a]# Pujan Sasmal,[a]# Swati De[c] and Jaideep Saha*[a, †]

[a]Department of Medicinal Chemistry, National Institute of Pharmaceutical Education and Research (NIPER), Mohali-160062, India. [†]Molecular Design and Synthesis Unit, Rutgers, the State University of New Jersey, Piscataway, New Jersey 08854, United States. [b]Department of Biological and Synthetic Chemistry, Centre of Biomedical Research, Lucknow 226014. UP India.[c]University of Kalyani, West Bengal, 741235, India.

# Equal contribution

Email: jdsaha2000@gmail.com; js3954@rutgers.edu

Table of contents

Entry	Description	Page
1	General Experimental……………………………………………………………….....	3
2	Preparation of the starting materials with general Procedure ……………………	4-14
3	Reaction Optimization………………………………………………………………	15-17
4	General procedure for the Tandem N/C3 Functionalization of ABB-carbinol with DAC to functionalized Azetidines…………………………………………………..	18
5	Characterization of compounds (**3-36**) (from Scheme 1: main text)……………....	19-34
6	General procedure for the preparation of spiroepoxy azetidines (**37-41**)……….	35
7	General procedure for the preparation of spiro azetidines (**42-44**)………………	35
8	Characterization of compounds (**37-44**) (from Scheme 3: main text)……………	36-39
9	General procedure for the preparation of azetidinyl haloketones (**45-49**)………	40
10	Characterization of compounds (**45-49**) (from Scheme 3: main text)	41-43
11	General procedure for the tandem N/C3 functionalization of ABB-carbinol with BCBs to functionalized azetidines………………………………………………….	44
12	Characterization of compounds (**52-66**) (from Scheme 4: main text)……………	45-51
13	Scale up and reaction profile……………………………………………………...	52
14	Control experiments…………………………………………………………………	53-54
15	1D NOE Experiment for compound **64** and **66** ……………………………………	55-57
16	Plausible Mechanism ………………………………………………………………	58
17	NMR spectra of Starting Materials ………………………………………………..	59 -65
15	NMR spectra of new compounds (**3-69**)……………………………………………..	66-137
16	References…………………………………………………………………………	138

1. General Experimental

Unless otherwise noted, all new reactions reported herein were performed using oven-dried or flame-dried glassware under argon atmosphere and stirred magnetically. Solvents received from commercial sources were dried using standard protocols before using in this study and for THF, it was used as freshly distilled. Unless noted, all the reagents and catalysts were used as it was received from commercial sources and no further purification was made on those. $Yb(OTf)_3$ were purchased Sigma-Aldrich. Reaction monitoring was performed via TLC, using Merck silica gel 60 F 254 plates. TLC plates were visualized either under UV light (254 nm) or by using 10% ethanolic phosphomolybdic acid (PMA) or 1% aqueous $KMnO_4$ or iodine. Silica gel of 230-400 mesh size was used for the flash column chromatography. ^1H, ^{13}C NMR spectra were recorded on Avance III, Bruker at 400 MHz and 800 MHz NMR spectrometers. In the experimental section, the ^1H NMR chemicals shift are expressed in the form of ppm (δ) relative to δ = 7.26 for $CDCl_3$ whereas ^{13}C NMR chemical shift are expressed relative to δ = 77.16. All coupling constants are apparent J values measured in Hertz. The following abbreviations were used to refer to multiplicities: s = singlet, d = doublet, dd = doublet of doublets, t = triplet, q = quartet, m = multiplet. HRMS and Electron Spray Ionization (ESI) (m/z) spectra were recorded on Agilent Technologies 6530 Accurate Mass Q-TOF LC/MS at the Centre of Biomedical Research Mass Spectrometry Service. Structural assignments were made with additional information from gNOESY, gHSQC, and gCOSY experiments. Dry DCE means DCE was dried over CaH_2, distilled under inert atmosphere, and stored over activated 4 Å molecular sieves (anhydrous).

2. Preparation of starting materials

2.1 Donor–Acceptor(D-A) Cyclopropanes used in the current study

Following compounds are known in the literature and were prepared following the reported procedure[1] and characterized via NMR/MS.

List of Donner-Acepector Cyclopropanes (DACs)

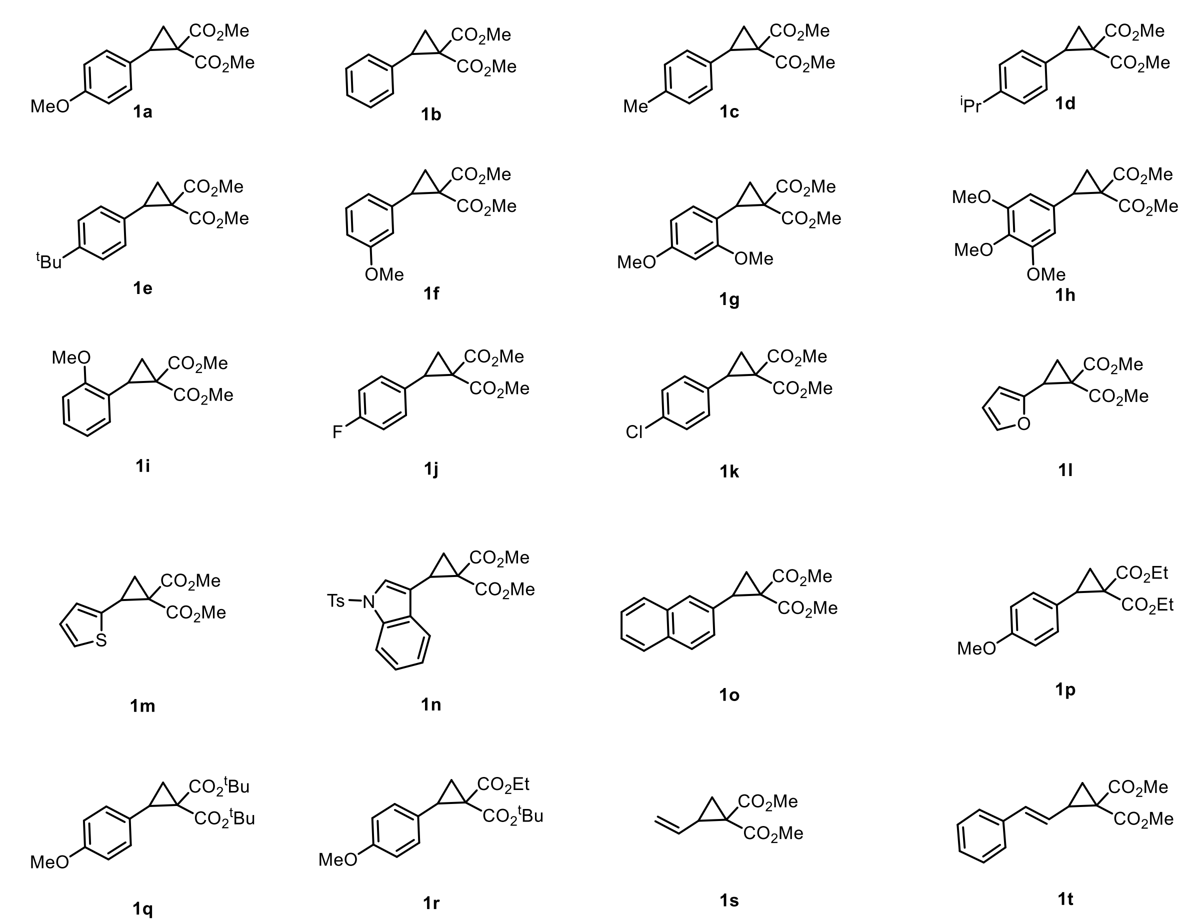

Unsuccessful substrate

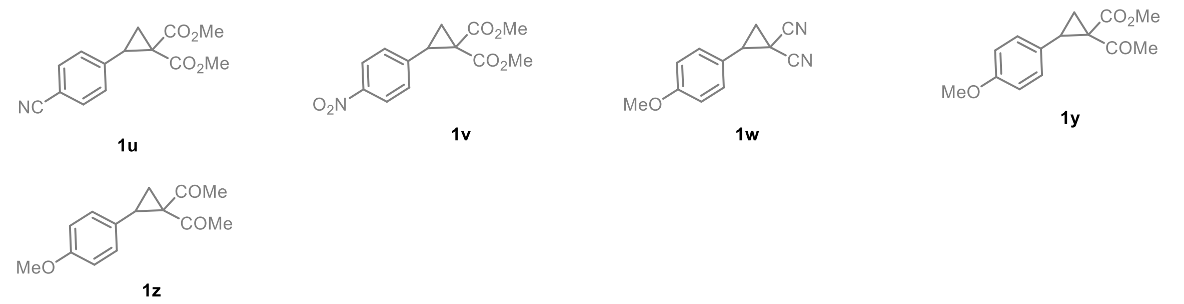

Figure S1: List of different Donner-Acceptor Cyclopropanes (DACs) used in current study.

2.2 Various Azabicyclo[1.1.0]butyl carbinols and keto used in this study

Following compounds are known in the literature and were prepared following the reported procedure[2] and characterized via NMR/MS.

List of ABB-aryl Carbinols

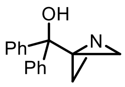

2a

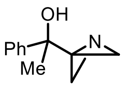

2b

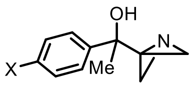

2c, X = Br
2d, X = OMe

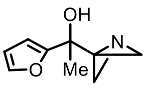

2e

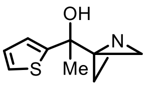

2f

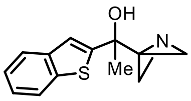

2g

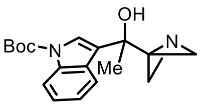

2h

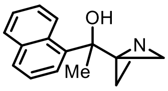

2i

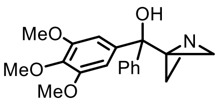

2j

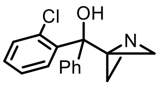

2k

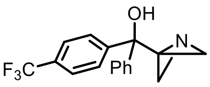

2l

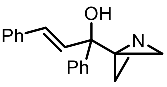

2m

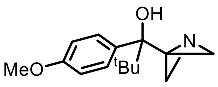

2n

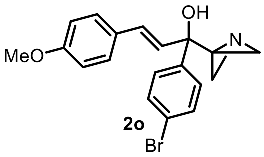

2o

List of ABB-cyclic Carbinols

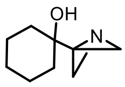

2p

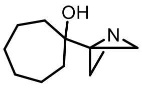

2q

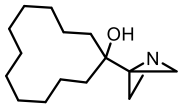

2r

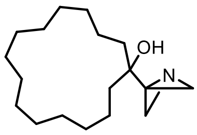

2s

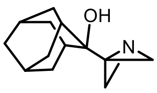

2t

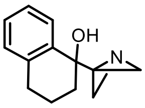

2v

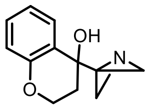

2w

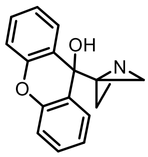

2x

List of ABB-keto

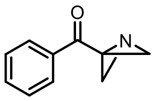

2'a

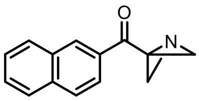

2'b

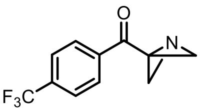

2'c

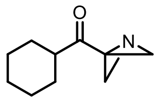

2'd

Figure S2. List of ABB derivatives used in the study

2.3. *Various Bicyclo[1.1.0] butane (BCBs) used in this study*

Following compounds are known in the literature and were prepared following the reported procedure[3] and characterized via NMR/MS

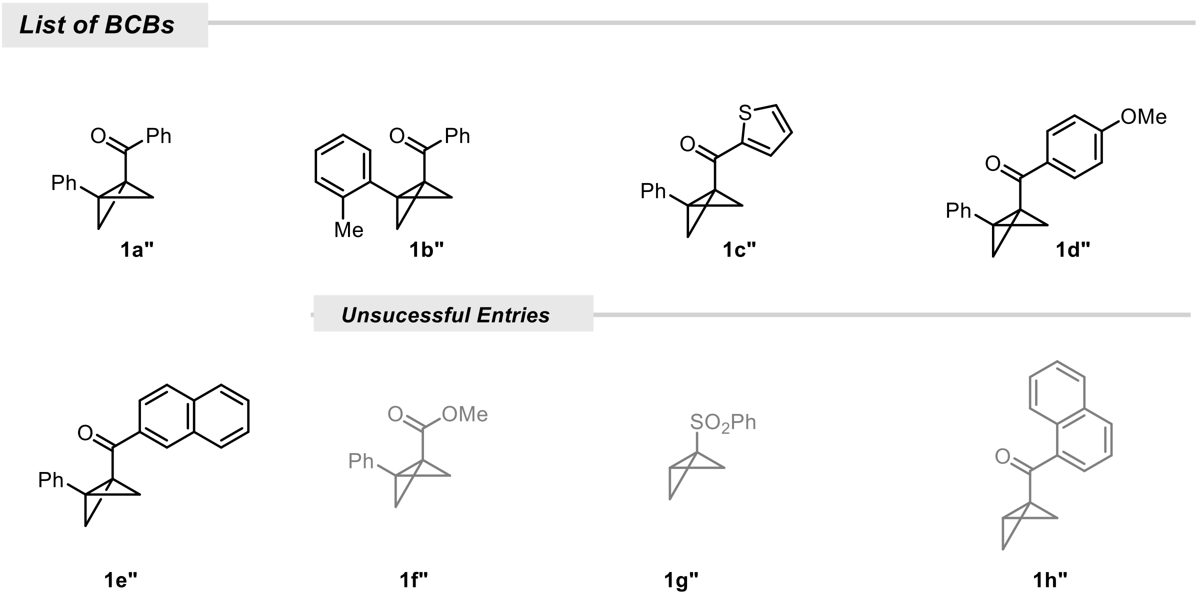

Figure S3. List of BCB derivatives used in the study

2.4. *Synthesis of azabicyclo[1.1.0]butyl carbinols*

General Procedure A:

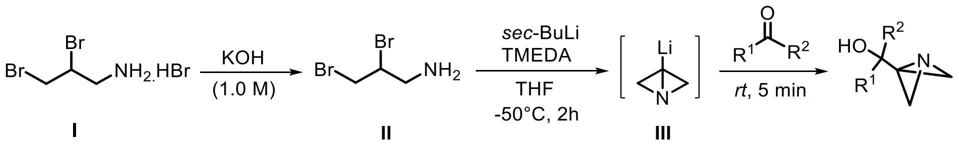

ABB-carbinols were synthesized according to a literature procedure.[2] Free amine (intermediate II; 2,3-dibromopropan-1-amine) was initially obtained from the corresponding HBr salt (I) by treating the latter with KOH. Compound II (1.0 equiv) was dissolved in dry THF (0.4 M) and to this was introduced freshly distilled TMEDA (3.2 equiv). The mixture was cooled to -50°C and into this solution was added sec-butyllithium (1.3 M in cyclohexane; 3.2 equiv) dropwise at the same temperature. After 2 h, a carbonyl derivative (2.0 equiv) in dry THF (2M) was introduced and the solution was stirred for 5 minutes outside the cooling bath. The mixture was quenched using water (10 mL) and extracted using DCM (3 x 10 mL). The combined organic phase was separated, dried over anhydrous Na2SO4, filtered, and concentrated under reduced pressure. The residue was purified by silica gel column chromatography to afford the ABB-carbinols.

All the compounds were prepared by the method delineated above. These are reported compounds that are characterized through comparison with the published NMR data.[2]

General Procedure B:

2.5. Synthesis of azabicyclo[1.1.0]butyl ketone

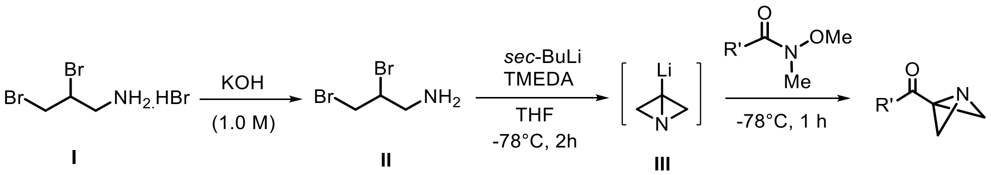

The ABB-ketones employed in these studies were prepared following a literature protocol, characterized, and confirmed by comparison with reported NMR data. In this procedure, the HBr salt (I) was treated with KOH to generate 2,3-dibromopropan-1-amine (II). Subsequently, freshly distilled TMEDA (3.2 equiv.) was added to compound II (1.0 equiv.) in dry THF (0.4 M). The reaction mixture was then cooled to -78°C, followed by dropwise addition of sec-butyllithium (1.3 M in cyclohexane; 3.2 equiv.) at the same temperature. After two hours, the corresponding Weinreb amide (2.0 equiv.) in dry THF (2.0 M) was added, and the mixture was stirred for one hour at -78°C. The reaction mixture was quenched with water (ca. 10 mL) and extracted with DCM (3 × 10 mL). The organic phase was separated, dried over anhydrous Na2SO4, filtered, and concentrated under reduced pressure. The crude residue was purified by silica gel column chromatography to provide the desired ABB-ketone.

(1-Azabicyclo[1.1.0]butan-3-yl)diphenylmethanol (**2a**)

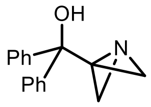

Following the general procedure-**A**, reaction between 2,3-dibromopropan-1-amine (0.524 g, 2.43 mmol, 1.0 equiv.) and Benzophenone (0.884 g, 4.860 mmol, 2.0 equiv.) afforded desired product **2a,** which was purified by silica gel column chromatography (3:10 EtOAc:Hexane as eluent) to give the title compound as white solid 32% (0.200g) yield R_f 0.2 (3:10 EtOAc:Hexane); **^{1}H NMR** (600 MHz, Chloroform-*d*) δ 7.51 – 7.47 (m, 4H), 7.32 (dd, *J* = 8.3, 6.7 Hz, 4H), 7.26 (t, *J* = 7.5 Hz, 2H), 3.09 (s, 1H), 2.30 (t, *J* = 1.4 Hz, 2H), 1.47 (t, *J* = 1.4 Hz, 2H). The analytical and physical data are matching with the reported values.[2]

1-(1-Azabicyclo[1.1.0]butan-3-yl)-1-(furan-2-yl)ethan-1-ol (**2e**)

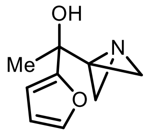

Following the general procedure-**A**, reaction between 2,3-dibromopropan-1-amine (0.524 g, 2.43 mmol, 1.0 equiv.) and 1-(furan-2-yl)ethan-1-one (0.553 g, 4.860 mmol, 2.0 equiv.) afforded desired product **2e,** which was purified by silica gel column chromatography (3:10 EtOAc:Hexane as eluent) to give the title compound as yellow solid 38% (0.150 g) yield R_f 0.2 (3:10 EtOAc:Hexane); **^{1}H NMR** (800 MHz, Chloroform-*d*) δ 7.40 – 7.38 (m, 1H), 6.35 – 6.32 (m, 2H), 2.97 (s, 1H), 2.48 (dd, *J* = 6.6, 2.6 Hz, 1H), 2.33 (dd, *J* = 6.6, 2.7 Hz, 1H), 1.70 (s, 3H), 1.28 (t, *J* = 2.2 Hz, 2H). The analytical and physical data are matching with the reported values.[2]

1-(1-azabicyclo[1.1.0]butan-3-yl)-1-(thiophen-2-yl)ethan-1-ol (**2f**)

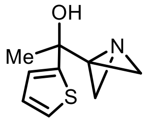

Following the general procedure-**A**, reaction between 2,3-dibromopropan-1-amine (0.524 g, 2.43 mmol, 1.0 equiv.) and 1-(furan-2-yl)ethan-1-one (0.640 g, 4.860 mmol, 2.0 equiv.) afforded desired product **2f,** which was purified by silica gel column chromatography (3:10 EtOAc:Hexane as eluent) to give the title compound as white solid 40% (0.180 g) yield R_f 0.2 (3:10 EtOAc:Hexane); **^{1}H NMR** (800 MHz, Chloroform-*d*) δ 7.26 (d, *J* = 5.0 Hz, 1H), 7.07 (dd, *J* = 3.5, 1.0 Hz, 1H), 7.00 (dd, *J* = 5.0, 3.6 Hz, 1H), 2.95 (s, 1H), 2.52 – 2.48 (m, 2H), 1.74 (s, 3H), 1.36 (d, *J* = 2.4 Hz, 1H), 1.29 (d, *J* = 2.6 Hz, 1H). The analytical and physical data are matching with the reported values.[2]

*1-(1-azabicyclo[1.1.0]butan-3-yl)-1-(4-methoxyphenyl)-2,2-dimethylpropan-1-ol (**2n**)*

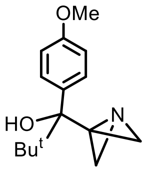

Following the general procedure-**A**, reaction between 2,3-dibromopropan-1-amine (0.524 g, 2.43 mmol, 1.0 equiv.) and 1-(4-methoxyphenyl)-2,2-dimethylpropan-1-one (0.700 g, 4.860 mmol, 2.0 equiv.) afforded desired product **2n,** which was purified by silica gel column chromatography (3:10 EtOAc:Hexane as eluent) to give the title compound as yellow solid 38% (0.200 g) yield R_f 0.2 (3:10 EtOAc:Hexane). **^{1}H NMR** (600 MHz, Chloroform-*d*) δ 7.39 (d, *J* = 8.9 Hz, 2H), 6.81 (d, *J* = 9.0 Hz, 2H), 3.79 (s, 3H), 2.68 (dd, *J* = 6.8, 2.8 Hz, 1H), 2.42 (dd, *J* = 6.8, 2.9 Hz, 1H), 2.35 (dd, *J* = 7.8, 2.9 Hz, 1H), 1.45 (d, *J* = 2.8 Hz, 1H), 1.39 (d, *J* = 2.8 Hz, 1H), 1.00 (s, 9H). The analytical and physical data are matching with the reported values.[2]

*(E)-1-(1-azabicyclo[1.1.0]butan-3-yl)-1-(4-bromophenyl)-3-(4-methoxyphenyl)prop-2-en-1-ol **(2o)***

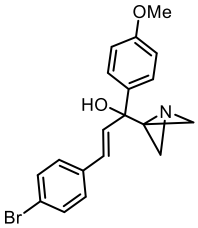

Following the general procedure-**A**, reaction between 2,3-dibromopropan-1-amine (0.524 g, 2.43 mmol, 1.0 equiv.) and (E)-1-(4-bromophenyl)-3-(4-methoxyphenyl)prop-2-en-1-one (0.770 g, 2.430 mmol, 1.0 equiv.) afforded desired product **2o,** which was purified by silica gel column chromatography (3:10 EtOAc:Hexane as eluent) to give the title compound as white solid 32% (0.290g) yield R_f 0.3 (3:10 EtOAc:Hexane); **^{1}H NMR** (400 MHz, $CDCl_3$) δ 7.48-7.34 (m, 4H), 7.13 (d, *J* = 8.6 Hz, 2H), 6.70 (d, *J* = 8.6 Hz, 2H), 6.50 (d, *J* = 15.9 Hz, 1H), 6.20 (d, *J* = 16.0 Hz, 1H), 4.23 (s, 1H), 3.67 (s, 3H), 2.49 (dd, *J* = 6.5, 2.4 Hz, 1H), 2.35 (dd, *J* = 6.5, 2.5 Hz, 1H), 1.39 (d, *J* = 2.3 Hz, 1H), 1.23 (d, *J* = 2.5 Hz, 1H). The analytical and physical data are matching with the reported values.[2]

1-(1-Azabicyclo[1.1.0]butan-3-yl)cyclododecan-1-ol (**2r**)

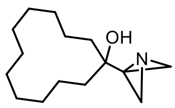

Following the general procedure-**A**, reaction between 2,3-dibromopropan-1-amine (0.524 g, 2.43 mmol, 1.0 equiv.) and cyclododecanone (0.884 g, 4.860 mmol, 2.0 equiv.) afforded desired product **2r,** which was purified by silica gel column chromatography (3:10 EtOAc:Hexane as eluent) to give the title compound as yellow solid 38% (0.220g) yield R_f 0.2 (2:10 EtOAc:Hexane); **^{1}H NMR** (400 MHz, $CDCl_3$) δ 2.93 (s, 1H), 2.39 (s, 2H), 1.61-1.48 (m, 6H), 1.34 (s, 16H), 1.12 (s, 2H). The analytical and physical data are matching with the reported values.[2]

1-(1-Azabicyclo[1.1.0]butan-3-yl)cyclopentadecan-1-ol (**2s**)

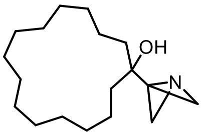

Following the general procedure-**A**, reaction between 2,3-dibromopropan-1-amine (0.524 g, 2.43 mmol, 1.0 equiv.) cyclopentadecanone and (1.08 g, 4.860 mmol, 2.0 equiv.) afforded desired product **2s,** which was purified by silica gel column chromatography (3:10 EtOAc:Hexane as eluent) to give the title compound as yellow solid 68% (0.300 g) yield R_f 0.3 (3:10 EtOAc:Hexane); **^{1}H NMR** (400 MHz, $CDCl_3$) δ 2.90 (s, 1H), 2.39 (s, 2H), 1.53 (s, 4H), 1.38-1.25 (m, 24H), 1.14 (s, 2H). The analytical and physical data are matching with the reported values.[2]

2-(1-Azabicyclo[1.1.0]butan-3-yl)adamantan-2-ol (**2t**)

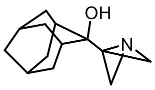

Following the general procedure-**A**, reaction between 2,3-dibromopropan-1-amine (0.524 g, 2.43 mmol, 1.0 equiv.) and adamantan-2-one (0.729 g, 4.860 mmol, 2.0 equiv.) afforded desired product **2t,** which was purified by silica gel column chromatography (3:10 EtOAc:Hexane as eluent) to give the title compound as yellow solid 46% (0.230g) yield R_f 0.3 (3:10 EtOAc:Hexane); **^{1}H NMR** (400 MHz, $CDCl_3$) δ 2.43 (s, 2H), 2.20 (d, J = 12.1 Hz, 2H), 1.99 (d, J = 13.0 Hz, 2H), 1.84-1.70 (m, 4H), 1.66 (s, 4H), 1.51 (d, J = 12.3 Hz, 2H), 1.33 (s, 2H). The analytical and physical data are matching with the reported values.[2]

2.6. General Procedure for the Synthesis of Bicyclo[1.1.0]butanes

General Procedure C:

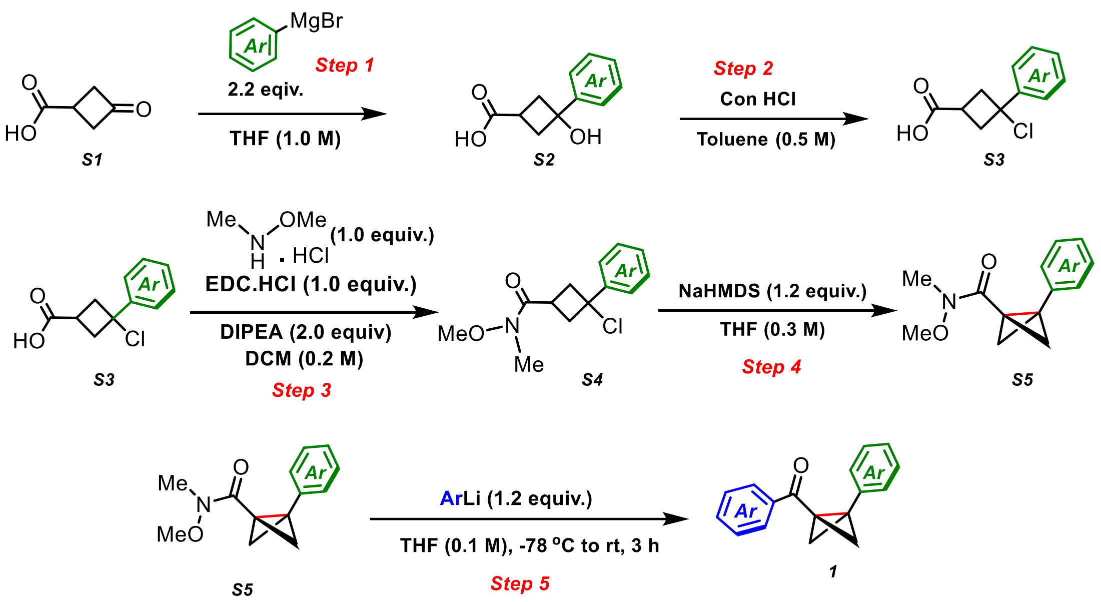

Step 1: In a 250 mL two-neck round-bottom flask, 3-oxocyclobutanecarboxylic acid (8.0 g, 70 mmol, 1.0 equiv.) was dissolved in dry THF (70 mL, 1.0 M) and stirred at 0 °C. To this solution, Grignard reagent (156 mmol, 2.20 equiv.) was added dropwise over 30 min, and the mixture was stirred for 6 h at room temperature under an argon atmosphere. After completion of the reaction, saturated aqueous NH4Cl solution (50 mL) was added, and the mixture was acidified (pH < 7) using 1.0 M HCl. The resulting mixture was extracted with EtOAc. The combined organic layers were washed with brine (50 mL), dried over Na2SO4, and concentrated under reduced pressure to provide the crude product **S2** (12 g, 62 mmol). The crude material was used directly in the next step without additional purification.

Step 2: In a 250 mL round-bottom flask, **S2** (12 g, 62 mmol, 1.0 equiv.) was dissolved in toluene (120 mL, 0.5 M). To this solution, 12 N HCl (120 mL) was added. The resulting mixture was stirred at room temperature for 4 h. After completion of the reaction, the organic layer was separated, washed with water (2 × 50 mL) and brine (2 × 50 mL), and concentrated under reduced pressure to yield the crude product **S3** (11.9 g, 57.0 mmol). The crude material was directly used in the subsequent step without additional purification.

Step 3: **S3** (2.7 g, 13 mmol, 1.0 equiv) was placed in a two-neck flask and dissolved in CH2Cl2 (65 mL, 0.2 M) under an argon atmosphere. Subsequently, N,O-dimethylhydroxylamine hydrochloride (1.3 g, 13.0 mmol, 1.00 equiv.) and DIPEA (2.0 mL, 26 mmol, 2.0 equiv.) were added sequentially. After stirring for 5 minutes, EDC-HCl (3.0 g, 13.0 mmol, 1.0 equiv.) was

introduced at 0 °C, and the reaction mixture was allowed to stir at room temperature for 12 h. The reaction was then quenched with saturated aqueous NH4Cl (25 mL), extracted with CH2Cl2 (2 × 25 mL), dried over Na2SO4, and concentrated under reduced pressure to afford **S4** (1.7 g, 6.7 mmol), which was used directly in the subsequent step without further purification.

Step 4: **S4** (1.4 g, 5.5 mmol, 1.0 equiv) was charged into a 100 mL two-neck flask and dissolved in THF (18 mL, 0.3 M) under an argon atmosphere. Subsequently, NaHMDS (6.6 mL, 6.6 mmol, 1.2 equiv.) was added dropwise at 0 °C, and the reaction mixture was stirred for 4 h at room temperature. The reaction was then quenched with NH4Cl (25 mL) and extracted with EtOAc (25 mL). The combined organic extracts were dried over Na2SO4, and the solvent was removed under reduced pressure. The crude residue was purified by flash column chromatography using EtOAc and petroleum ether (1:20) as the eluent to afford the pure product (**S5**) as a white solid (530 mg, 2.4 mmol, 44%).

Step 5: An oven-dried 50 mL round-bottom flask equipped with a stir bar was evacuated, backfilled with argon three times, and sealed with a septum. N-methoxy-N-methyl-3-phenylbicyclo[1.1.0]butane-1 (**S5**) (1.7 g, 7.8 mmol, 1 equiv.) and THF (0.1 M, 78 mL) were added under an argon atmosphere. The reaction mixture was cooled to -78 °C, followed by dropwise addition of PhLi (6.3 mL, 9.4 mmol, 1.2 equiv., 1.5 M in hexane). The mixture was stirred for 30 minutes at the same temperature and then for 2.5 h at room temperature before being quenched with NH4Cl solution (10 mL). The aqueous layer was extracted with EtOAc (3 × 20 mL). The combined organic extracts were dried over Na2SO4, and the solvent was removed under reduced pressure. The crude product was purified by flash column chromatography using EtOAc and petroleum ether (1:20) as the eluent to provide the pure product (**1**) as a white solid (500 mg, 2.13 mmol, 27%).

Phenyl(3-phenylbicyclo[1.1.0]butan-1-yl)methanone (**1a"**)

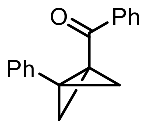

Following the general procedure - **C**, 1a" was isolated as a colorless solid (0.130 g, 30% yield over five steps). The physical data are in agreement with the reported value.[3]

^{1}H NMR (600 MHz, Chloroform-*d*) δ 7.58 – 7.52 (m, 2H), 7.48 – 7.43 (m, 1H), 7.36 – 7.31 (m, 2H), 7.25 – 7.18 (m, 3H), 7.16 – 7.10 (m, 2H), 3.17 (t, *J* = 1.3 Hz, 2H), 1.91 (t, *J* = 1.3 Hz, 2H).

Phenyl(3-(o-tolyl)bicyclo[1.1.0]butan-1-yl)methanone (**1b"**)

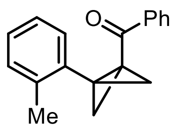

Following the general procedure - **C**, 1b" was isolated as a White solid (0.120 g, 29% yield over five steps). The physical data are in agreement with the reported value.[3]

^{1}H NMR (600 MHz, Chloroform-d) δ 7.80 (dd, J = 8.3, 1.4 Hz, 2H), 7.53 – 7.48 (m, 1H), 7.41 (dd, J = 8.4, 7.1 Hz, 2H), 7.13 – 7.08 (m, 2H), 7.02 (ddd, J = 8.6, 6.6, 2.2 Hz, 1H), 6.90 – 6.85 (m, 1H), 2.90 (d, J = 0.9 Hz, 2H), 2.39 (s, 3H), 1.96 (d, J = 0.9 Hz, 2H).

(3-phenylbicyclo[1.1.0]butan-1-yl)(thiophen-2-yl)methanone (**1c"**)

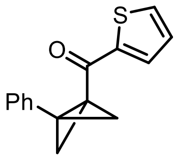

Following the general procedure - **C**, 1c" was isolated as a colorless solid (0.140 g, 34% yield over five steps). The physical data are in agreement with the reported value.[3]

^{1}H NMR (600 MHz, Chloroform-*d*) δ 7.68 (dd, *J* = 3.8, 1.2 Hz, 1H), 7.52 (dd, *J* = 4.9, 1.2 Hz, 1H), 7.26 – 7.22 (m, 4H), 7.22 – 7.17 (m, 1H), 7.08 (dd, *J* = 4.9, 3.7 Hz, 1H), 3.31 (d, *J* = 1.3 Hz, 2H), 1.88 (t, *J* = 1.2 Hz, 2H).

(4-methoxyphenyl)(3-phenylbicyclo[1.1.0]butan-1-yl)methanone (**1d"**)

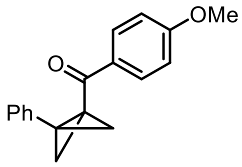

Following the general procedure - **C**, 1d" was isolated as a colorless solid (0.140 g, 33% yield over five steps). The physical data are in agreement with the reported value.[3]

^{1}H NMR (600 MHz, Chloroform-*d*) δ 7.62 (d, *J* = 8.8 Hz, 2H), 7.24 – 7.20 (m, 2H), 7.20 – 7.17 (m, 1H), 7.17 – 7.13 (m, 2H), 6.86 – 6.81 (m, 2H), 3.83 (s, 3H), 3.16 (t, *J* = 1.2 Hz, 2H), 1.88 (t, *J* = 1.2 Hz, 2H).

Naphthalen-2-yl(3-phenylbicyclo[1.1.0]butan-1-yl)methanone (**1e"**)

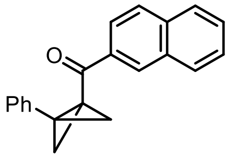

Following the general procedure - **C**, 1e" was isolated as a yellow solid (0.178 g, 38% yield over five steps). The physical data are in agreement with the reported value.[3]

^{1}H NMR (600 MHz, Chloroform-*d*) δ 8.15 (d, *J* = 1.6 Hz, 1H), 7.89 (d, *J* = 8.0 Hz, 1H), 7.84 (d, *J* = 8.0 Hz, 1H), 7.77 (d, *J* = 8.5 Hz, 1H), 7.59 – 7.55 (m, 2H), 7.53 (td, *J* = 7.5, 6.8, 1.4 Hz, 1H), 7.23 – 7.20 (m, 3H), 7.17 – 7.14 (m, 2H), 3.25 (d, *J* = 1.3 Hz, 2H), 1.99 (t, *J* = 1.3 Hz, 2H).

3. Reaction optimization

3.1 Screening of Metal Catalysts:

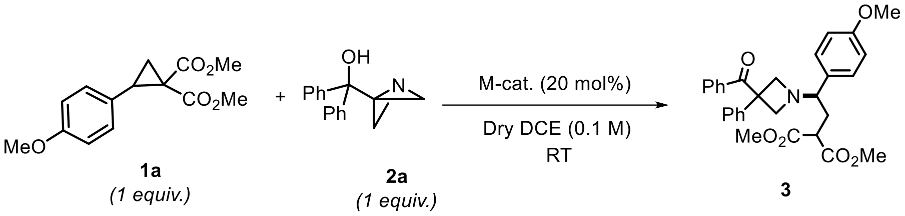

Table S1[a]

Entry	M-cat. [20 mol%]	**3** Yield(%)[b]
1	$Sc(OTf)_3$	23
2	$Yb(OTf)_3$	65
3	$Cu(OTf)_2$	35
4	$NiClO_4.6H_2O$	52
5	MgI_2	Trace
6	$Sn(OTf)_2$	15
7	$In(OTf)_3$	30
8	$BF_3.OEt_2$	0
9	$Bi(OTf)_3$	0
10[c]	$Yb(OTf)_3$	60
11	$Mg(OTf)_2$	48
12	$Zn(OTf)_2$	35
13	$Y(OTf)_3$	55
14	$Ga(OTf)_3$	27
15	$Fe(OTf)_3$	Trace
16[d]	$Mg(OTf)_2$	45
17[d]	$Y(OTf)_3$	48

[a]Reaction conditions: Compound **1a** (1.0 equiv.), Compound **2a** (1.0 equiv.) were taken in DCE solvent (0.1 M) and different kind of Metal-Catalyst (20 mol%) were added. The reaction was run for specific time (12 h) and at RT under inert condition. [b]Yields of the isolated product. [c] 4Å Molecular Sieves was used. [d] Instead of DCE solvent ,THF was used as a solvent.

3.2 Screening of catalyst loading

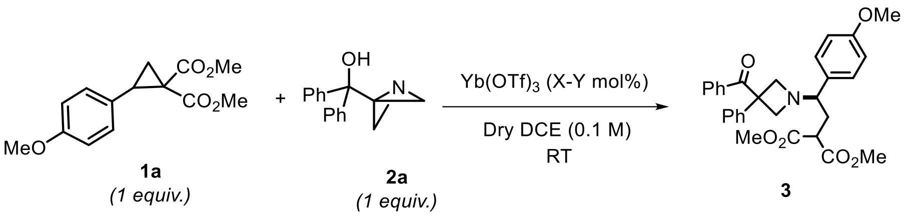

Table S2[a]

Entry	Cat. [mol%]	**3** Yield(%)[b]
1	[10]	50
2	[20]	65
3	[30]	75
4	[40]	55
5	[60]	20
6	[100]	Trace

[a]Reaction conditions: Compound **1a** (1.0 equiv.), Compound **2a** (1.0 equiv.) were taken in DCE solvent (0.1 M) and $Yb(OTf)_3$ (X-Y mol%) were added. The reaction was run for specific time (12 h) at RT under inert condition. [b]Yields of the isolated product.

.

3.3 Screening of Solvent Concentration

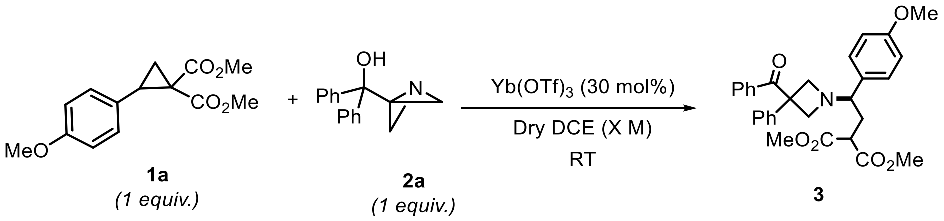

Table S3[a]

Entry	Conc. [M]	**3** Yield(%)[b]
1	0.1	75
2	0.2	68
3	0.05	55

[a]Reaction conditions: Compound **1a** (1.0 equiv.), Compound **2a** (1.0 equiv.) were taken in DCE solvent (X.0 M) and $Yb(OTf)_3$ (30 mol%) were added. The reaction was run for specific time (12 h) at RT under inert condition. [b]Yields of the isolated product.

3.4 Screening of Solvents

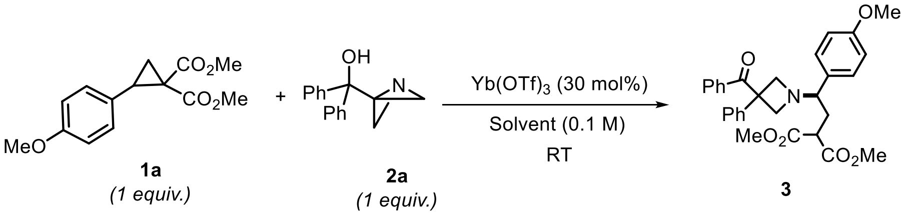

Table S4[a]

Entry	Solvent [0.1 M]	**3** Yield(%)[b]
1	DMF	0
2	DCE	75
3	THF	63
4	Toluene	Trace
5	ACN	70
6	HFIP	68
7	MeOH	0
8	EtOAc	18
9	Acetone	25
10	DMC	Trace
11[c]	DCE	Trace

[a]Reaction conditions: Compound **1a** (1.0 equiv.), Compound **2a** (1.0 equiv.) were taken in different solvent (0.1 M) and $Yb(OTf)_3$ (30 mol%) were added. The reaction was run for specific time (12 h) at RT under inert condition. [b]Yields of the isolated product. [c] 20 equivalent water was added along with the Dry DCE.

3.5 Screening of reaction temperature

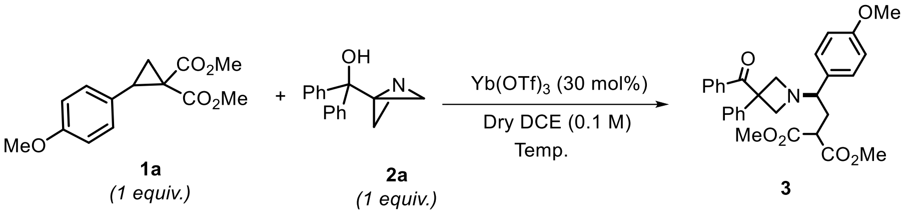

Table S5[a]

Entry	Temperature(°C)	**3** Yield(%)[b]
1	rt	75
2	50	82
3	60	86
4	80	96
5	100	48

[a]Reaction conditions: Compound **1a** (1.0 equiv.), Compound **2a** (1.0 equiv.) were taken in DCE solvent (0.1 M) and $Yb(OTf)_3$ (30 mol%) were added. The reaction was run for specific time (12 h) at elevated Temperature in an oil bath under inert condition. [b]Yields of the isolated product

4. Strain-Release-Driven Tandem N/C3 Functionalization of ABB-carbinol with Donor–Acceptor Cyclopropanes to functionalized Azetidines.

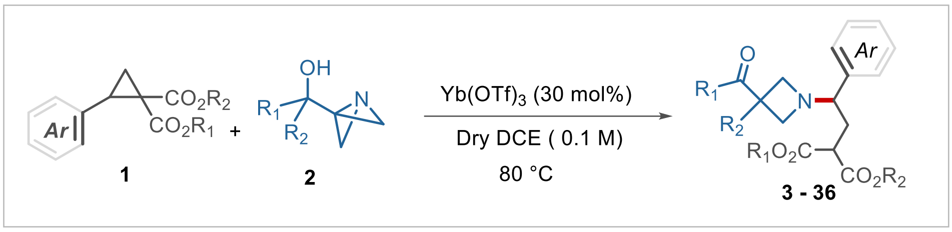

General procedure (1)

To a mixture of Donor–Acceptor Cyclopropanes (1, 1.0 equiv.), azabicyclo[1.1.0]butyl carbinols (2; 1.0 equiv.) and $Yb(OTf)_3$ (30 mol%) was added in dry DCE (0.1 M) in inert atmosphere. The reaction mixture was heated to 80°C in an oil bath and stirred until complete disappearance of the starting material was observed (*ca*. 12 h; TLC monitored). After cooling to room temperature, the mixture was diluted with H_2O and extracted in DCM. Combined organic layers were finally washed with brine, dried over anhydrous Na_2SO_4, filtered, and concentrated under reduced pressure. The crude residue was purified by silica-gel flash column chromatography (using EtOAc /hexanes as eluent) to provide desired products **(3-36).**

Here Dry DCE means, DCE was dried over CaH_2, distilled under inert atmosphere, and stored over activated 4 Å molecular sieves (anhydrous).

5. Characterization of compounds (3-36) (from Scheme 1: main text)

Dimethyl -2-(2-(3-benzoyl-3-phenylazetidin-1-yl)-2-(4-methoxyphenyl)ethyl)malonate (3)

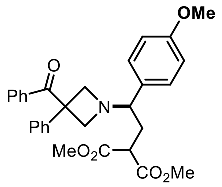

Following the general procedure 1, reaction between D-A cyclopropane (**1a**, 0.027 g, 0.1 mmol) and azabicyclo[1.1.0]butyl carbinols (**2a**, 0.024 g, 0.1 mmol) delivered compound **3**, which was purified by silica gel column chromatography (EtOAc: Hexane 2:8) to furnish the title compound **3** as a yellow liquid in 96% (0.048 g) yield. R_f 0.2 (EtOAc: Hexane 2:8)

^{1}H NMR (400 MHz, $CDCl_3$) δ 7.68 - 7.62 (m, 2H), 7.49 – 7.47(m, 2H), 7.44 – 7.40 (m,1H), 7.36 – 7.32 (m, 2H), 7.31 – 7.27 (m, 2H), 7.25 – 7.22 (m, 1H), 7.18 (d, *J* = 8.6 Hz, 2H), 6.84 (d, *J* = 8.6 Hz, 2H), 3.82 – 3.79 (m, 2H), 3.78 (s, 3H), 3.67 (s, 3H), 3.64 – 3.62 (m, 1H), 3.60 – 3.56 (m, 4H), 3.24 (dd, *J* = 8.4, 3.4 Hz, 1H), 3.14 (dd, *J* = 8.4, 6.1 Hz, 1H), 2.30 – 2.23 (m, 1H), 2.15 – 2.07 (m, 1H).**^{13}C {^{1}H} NMR** (100 MHz, $CDCl_3$) δ 199.6, 169.9, 169.8, 159.2, 141.5, 134.1, 133.0, 131.1, 129.7, 129.4, 129.1, 128.5, 127.2, 126.2, 114.0, 70.5, 62.5, 62.0, 55.3, 52.7, 52.6, 51.8, 48.0, 33.4.

HRMS (ESI-TOF) m/z: $[M+H]^+$ $C_{30}H_{32}NO_6$ Calcd.502.2224, Found 502.2251.

Dimethyl -2-(2-(3-benzoyl-3-phenylazetidin-1-yl)-2-phenylethyl)malonate (4).

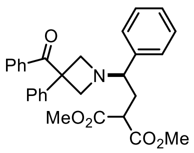

Following the general procedure 1, reaction between D-A cyclopropane (**1b**, 0.023 g, 0.1 mmol) and azabicyclo[1.1.0]butyl carbinols (**2a**, 0.024 g, 0.1 mmol) delivered compound **4**, which was purified by silica gel column chromatography (EtOAc: Hexane 2:8) to furnish the title compound **4** as a colurless liquid in 82% (0.039 g) yield. R_f 0.2 (EtOAc: Hexane 2:8)

^{1}H NMR (400 MHz, DMSO) δ 7.67 – 7.65 (m, 2H), 7.55 – 7.44 (m, 3H), 7.41 – 7.31 (m, 6H), 7.27 – 7.21 (m, 4H), 3.80 (d, *J* = 7.3 Hz, 1H), 3.71 (d, *J* = 7.3 Hz, 1H), 3.59 – 3.57 (m, 5H), 3.45 (s, 3H), 3.35 – 3.29 (m, 1H), 3.06 (t, *J* = 7.2 Hz, 1H), 2.21 – 2.14 (m, 1H), 2.07 – 1.94 (m, 1H). **^{13}C {^{1}H} NMR** (100 MHz, DMSO) δ 198.6, 169.0, 141.0, 139.0, 133.3, 133.2, 129.2, 129.0, 128.8, 128.4, 128.1, 127.6, 127.0, 126.1, 69.8, 61.7, 61.2, 52.4, 52.2, 51.4, 47.3, 32.6.

HRMS (ESI-TOF) m/z: $[M+H]^+$ $C_{29}H_{30}NO_5$ Calcd.472.2118, Found 472.2114.

Dimethyl -2-(2-(3-benzoyl-3-phenylazetidin-1-yl)-2-(p-tolyl)ethyl)malonate (5)

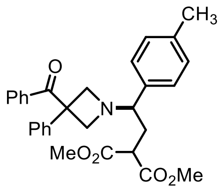

Following the general procedure 1, reaction between D-A cyclopropane (**1c**, 0.025 g, 0.1 mmol) and azabicyclo[1.1.0]butyl carbinols (**2a**, 0.024 g, 0.1 mmol) delivered compound **5**, which was purified by silica gel column chromatography (EtOAc: Hexane 2:8) to furnish the title compound **5** as a colurless liquid in 85% (0.041 g) yield. R_f 0.2 (EtOAc: Hexane 2:8)

^{1}H NMR (400 MHz, DMSO) δ 7.73 – 7.61 (m, 2H), 7.55 – 7.43 (m, 3H), 7.40 – 7.33 (m, 4H), 7.27 – 7.22 (m, 1H), 7.12 – 7.11 (m, 4H), 3.78 (d, *J* = 7.3 Hz, 1H), 3.69 (d, *J* = 7.3 Hz, 1H), 3.60 – 3.54 (m, 5H), 3.46 (s, 3H), 3.30 – 3.24 (m, 1H), 3.06 – 3.03 (m, 1H), 2.26 (s, 3H), 2.20 – 2.11 (m, 1H), 2.02 – 1.94 (m, 1H). **^{13}C {^{1}H} NMR** (100 MHz, DMSO) δ 198.6, 169.0, 141.0, 136.7, 135.9, 133.2, 129.2, 129.0(2), 128.7, 128.4, 127.9, 127.0, 126.1, 69.6, 61.7, 61.2, 52.4, 52.2, 51.4, 47.4, 32.7, 20.7.

HRMS (ESI-TOF) m/z: $[M+H]^+$ $C_{30}H_{32}NO_5$ Calcd.486.2275, Found 486.2259.

Dimethyl-2-(2-(3-benzoyl-3-phenylazetidin-1-yl)-2-(4-isopropylphenyl)ethyl)malonate (6)

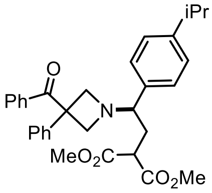

Following the general procedure 1, reaction between D-A cyclopropane (**1d**, 0.028 g, 0.1 mmol) and azabicyclo[1.1.0]butyl carbinols (**2a**, 0.024 g, 0.1 mmol) delivered compound **6**, which was purified by silica gel column chromatography (EtOAc: Hexane 2:8) to furnish the title compound **6** as a yellow liquid in 91% (0.047 g) yield. R_f 0.2 (EtOAc: Hexane 2:8)

^{1}H NMR (600 MHz, DMSO-*d*$_6$) δ 7.71 – 7.64 (m, 2H), 7.54 – 7.49 (m, 1H), 7.49 – 7.44 (m, 2H), 7.41 – 7.35 (m, 4H), 7.26 – 7.23 (m, 1H), 7.19 (d, *J* = 7.8 Hz, 2H), 7.15 (d, *J* = 7.9 Hz, 2H), 3.78 (d, *J* = 7.4 Hz, 1H), 3.70 (d, *J* = 7.4 Hz, 1H), 3.62 – 3.55 (m, 5H), 3.43 (s, 3H), 3.29 – 3.27 (m, 1H), 3.09 – 3.03 (m, 1H), 2.88 – 2.81 (m, 1H), 2.17 – 2.13 (m, 1H), 2.02 – 1.93 (m, 1H), 1.18 (d, *J* = 6.9 Hz, 6H). **^{13}C {^{1}H} NMR** (150 MHz, DMSO-*d*$_6$) δ 198.6, 169.0, 147.5, 141.0, 136.3, 133.2(2), 129.2, 129.0, 128.8, 128.0, 127.0, 126.3, 126.1, 69.6, 61.7, 61.2, 52.4, 52.2, 51.4, 47.4, 33.1, 32.7, 23.8(2).

HRMS (ESI-TOF) m/z: $[M+H]^+$ $C_{32}H_{36}NO_5$ Calcd.514.2588, Found 514.2595.

Dimethyl -2-(2-(3-benzoyl-3-phenylazetidin-1-yl)-2-(4-(tert-butyl)phenyl)ethyl)malonate (7)

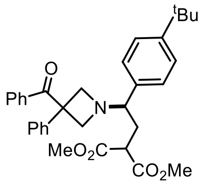

Following the general procedure 1, reaction between D-A cyclopropane (**1e**, 0.029 g, 0.1 mmol) and azabicyclo[1.1.0]butyl carbinols (**2a**, 0.024 g, 0.1 mmol) delivered compound **7**, which was purified by silica gel column chromatography (EtOAc: Hexane 2:8) to furnish the title compound **7** as a pale yellow liquid in 90% (0.047 g) yield. R_f 0.2 (EtOAc: Hexane 2:8)

^{1}H NMR (600 MHz, DMSO-d_6) δ 7.69 – 7.65 (m, 2H), 7.53 – 7.50 (m, 1H), 7.47 (d, *J* = 7.4 Hz, 2H), 7.40 – 7.34 (m, 6H), 7.25 (t, *J* = 7.3 Hz, 1H), 7.16 (d, *J* = 7.9 Hz, 2H), 3.78 (d, *J* = 7.3 Hz, 1H), 3.70 (d, *J* = 7.4 Hz, 1H), 3.57 (s, 5H), 3.43 (s, 3H), 3.29 – 3.27 (m, 1H), 3.08 – 3.04 (m, 1H), 2.17 – 2.12 (m, 1H), 2.02 – 1.94 (m, 1H), 1.26 (s, 9H). **^{13}C {^{1}H} NMR** (150 MHz, DMSO-*d*6) δ 198.6, 169.1, 149.8, 141.0, 136.0, 133.3, 133.2, 129.2, 129.0, 128.8, 127.7, 127.0, 126.1, 125.1, 69.6, 61.7, 61.3, 52.4, 52.2, 51.4, 47.4, 34.2, 32.7, 31.1.

HRMS (ESI-TOF) m/z: $[M+H]^+$ $C_{33}H_{38}NO_5$ Calcd. 528.2744, Found 528.2748.

Dimethyl -2-(2-(3-benzoyl-3-phenylazetidin-1-yl)-2-(3-methoxyphenyl)ethyl)malonate (8)

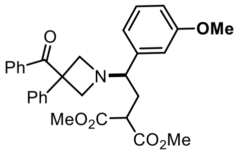

Following the general procedure 1, reaction between D-A cyclopropane (**1f**, 0.026 g, 0.1 mmol) and azabicyclo[1.1.0]butyl carbinols (**2a**, 0.024 g, 0.1 mmol) delivered compound **8**, which was purified by silica gel column chromatography (EtOAc: Hexane 2:8) to furnish the title compound **8** as a yellow liquid in 87% (0.044 g) yield. R_f 0.2 (EtOAc: Hexane 2:8)

^{1}H NMR (400 MHz, $CDCl_3$) δ 7.65 – 7.60 (m, 2H), 7.48 – 7.44 (m, 2H), 7.41 – 7.36 (m, 1H), 7.33 – 7.30 (m, 2H), 7.28 (d, *J* = 1.5 Hz, 1H), 7.24 – 7.22 (m, 1H), 7.22 – 7.17 (m, 2H), 6.83 – 6.80 (m, 2H), 6.79 – 6.75 (m, 1H), 3.80 (s, 2H), 3.76 (s, 3H), 3.64 (s, 3H), 3.62 (d, *J* = 2.9 Hz, 2H), 3.55 (s, 3H), 3.26 (dd, *J* = 8.2, 3.5 Hz, 1H), 3.15 (dd, *J* = 8.3, 6.2 Hz, 1H), 2.28 – 2.22 (m, 1H), 2.16 – 2.08 (m, 1H). **^{13}C {^{1}H} NMR** (100 MHz, $CDCl_3$) δ 199.5, 169.8(2), 159.8, 141.4, 140.8, 134.0, 133.0, 129.6, 129.6, 129.1, 128.5, 127.2, 126.2, 120.7, 113.8, 113.3, 71.0, 62.4, 62.1, 55.3, 52.7, 52.6, 51.8, 47.9, 33.2.
HRMS (ESI-TOF) m/z: $[M+H]^+$ $C_{30}H_{32}NO_6$ Calcd. 502.2224, Found 502.2218.

Dimethyl -2-(2-(3-benzoyl-3-phenylazetidin-1-yl)-2-(2,4-dimethoxyphenyl)ethyl) malonate (9)

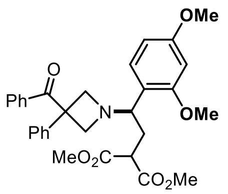

Following the general procedure 1, reaction between D-A cyclopropane (**1g**, 0.029 g, 0.1 mmol) and azabicyclo[1.1.0]butyl carbinols (**2a**, 0.024 g, 0.1 mmol) delivered compound **9**, which was purified by silica gel column chromatography (EtOAc: Hexane 2:8) to furnish the title compound **9** as a yellow gummy liquid in 90% (0.048 g) yield. R_f 0.2 (EtOAc: Hexane 2:8)

^{1}H NMR (400 MHz, $CDCl_3$) δ 7.68 – 7.63 (m, 2H), 7.54 – 7.48 (m, 2H), 7.43 – 7.40 (m,1H), 7.37 – 7.29 (m, 5H), 7.00 (d, *J* = 2.3 Hz, 1H), 6.76 – 6.71 (m, 2H), 3.91 – 3.86 (m, 2H), 3.83 – 3.81 (m, 1H), 3.77 (s, 3H), 3.74 – 3.70 (m, 4H), 3.61 (s, 4H), 3.59 – 3.58(m, 3H), 3.23 (t, *J* = 7.1 Hz, 1H), 2.37 – 2.31 (m, 1H), 2.12 – 2.05 (m, 1H). **^{13}C {^{1}H} NMR** (101 MHz, $CDCl_3$) δ 199.7, 170.3, 169.9, 153.7, 151.6, 141.7, 134.2, 132.9, 129.7, 129.1, 128.5, 128.3, 127.1, 126.3, 114.7, 112.6, 111.3, 63.0, 62.5, 62.3, 55.8, 55.7, 52.5(2), 51.7, 47.6, 31.5.

HRMS (ESI-TOF) m/z: $[M+H]^+$ $C_{31}H_{34}NO_7$ Calcd.532.2330, Found 532.2359.

Dimethyl -2-(2-(3-benzoyl-3-phenylazetidin-1-yl)-2-(3,4,5-trimethoxyphenyl)ethyl) malonate (10)

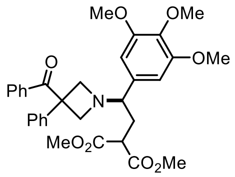

Following the general procedure 1, reaction between D-A cyclopropane (**1h**, 0.032 g, 0.1 mmol) and azabicyclo[1.1.0]butyl carbinols (**2a**, 0.024 g, 0.1 mmol) delivered compound **10**, which was purified by silica gel column chromatography (EtOAc: Hexane 2:8) to furnish the title compound **10** as a reddish liquid in 87% (0.049 g) yield. R_f 0.2 (EtOAc: Hexane 2:8)

^{1}H NMR (400 MHz, $CDCl_3$) δ 7.66 (d, *J* = 7.4 Hz, 2H), 7.48 (d, *J* = 7.4 Hz, 2H), 7.45 – 7.40 (m, 1H), 7.37 – 7.29 (m, 5H), 6.50 – 6.47 (m, 2H), 3.84 – 3.79 (m, 11H), 3.72 – 3.67 (m, 5H), 3.59 (s, 3H), 3.24 – 3.18 (m, 2H), 2.29 – 2.21 (m, 1H), 2.15 – 2.08 (m, 1H). **^{13}C {^{1}H} NMR** (101 MHz, $CDCl_3$) δ 199.6, 169.9, 169.8, 153.3, 141.4, 137.3, 136.7, 134.9, 134.1, 133.0, 129.7, 129.2, 128.6, 127.5, 127.3, 126.2, 105.0, 71.3, 62.5, 62.1, 61.0, 56.2, 52.7, 52.6, 51.9, 48.1, 33.3.

HRMS (ESI-TOF) m/z: $[M+H]^+$ $C_{32}H_{36}NO_8$ Calcd.562.2435, Found 562.2460.

Dimethyl -2-(2-(3-benzoyl-3-phenylazetidin-1-yl)-2-(2-methoxyphenyl)ethyl)malonate (11)

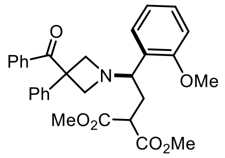

Following the general procedure 1, reaction between D-A cyclopropane (**1i**, 0.026 g, 0.1 mmol) and azabicyclo[1.1.0]butyl carbinols (**2a**, 0.024 g, 0.1 mmol) delivered compound **11**, which was purified by silica gel column chromatography (EtOAc: Hexane 2:8) to furnish the title compound **11** as a yellow gummy liquid in 88% (0.044 g) yield. R_f 0.2 (EtOAc: Hexane 2:8)

^{1}H NMR (400 MHz, $CDCl_3$) δ 7.66 – 7.61 (m, 2H), 7.49 (d, *J* = 7.4 Hz, 2H), 7.40 – 7.34 (m, 2H), 7.32 – 7.30 (m, 2H), 7.28 – 7.27 (m, 1H), 7.24 – 7.14 (m, 3H), 6.91 (t, *J* = 7.3 Hz, 1H), 6.79 (d, *J* = 8.2 Hz, 1H), 3.91 (dd, *J* = 6.9, 3.2 Hz, 1H), 3.85 – 3.77 (m, 2H), 3.73 (s, 3H), 3.67 – 3.63 (m, 1H), 3.61 – 3.59 (m, 1H), 3.55 (d, *J* = 7.7 Hz, 6H), 3.20 (d, *J* = 6.9 Hz, 1H), 2.37 – 2.31 (m, 1H), 2.11 – 2.04 (m, 1H). **^{13}C {^{1}H} NMR** (101 MHz, $CDCl_3$) δ 199.7, 170.3, 169.9, 157.3, 141.7, 134.1, 132.9, 129.6, 129.1, 128.6, 128.5, 128.3, 127.5, 127.1, 126.3, 120.7, 110.3, 62.9, 62.5, 62.3, 55.2, 52.5 (2), 51.7, 47.6, 31.4.

HRMS (ESI-TOF) m/z: $[M+Na]^+$ $C_{30}H_{31}NaNO_6$ Calcd.524.2044, Found 524.2063.

Dimethyl -2-(2-(3-benzoyl-3-phenylazetidin-1-yl)-2-(4-fluorophenyl)ethyl)malonate (12)

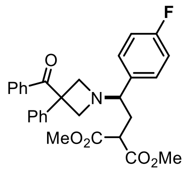

Following the general procedure 1, reaction between D-A cyclopropane (**1j**, 0.025 g, 0.1 mmol) and azabicyclo[1.1.0]butyl carbinols (**2a**, 0.024 g, 0.1 mmol) delivered compound **12**, which was purified by silica gel column chromatography (EtOAc: Hexane 2:8) to furnish the title compound **12** as a white liquid in 91% (0.045 g) yield. R_f 0.2 (EtOAc: Hexane 2:8)

^{1}H NMR (400 MHz, $CDCl_3$) δ 7.66 – 7.63 (m, 2H), 7.51 – 7.45 (m, 2H), 7.45 – 7.39 (m, 1H), 7.37 – 7.33 (m, 2H), 7.32 – 7.28 (m, 2H), 7.26 – 7.21 (m, 3H), 7.00 (t, *J* = 8.7 Hz, 2H), 3.85 – 3.76 (m, 2H), 3.67 (s, 3H), 3.64 – 3.60 (m, 1H), 3.58 – 3.55 (m, 4H), 3.30 – 3.27 (m, 1H), 3.13 – 3.09 (m, 1H), 2.31 – 2.24 (m, 1H), 2.16 – 2.09 (m, 1H). **^{13}C {^{1}H} NMR** (101 MHz, $CDCl_3$) δ 199.5, 169.7, 169.7, 163.6, 161.2, 141.4, 134.9, 134.9, 134.0, 133.1, 129.9, 129.8, 129.7, 129.2, 128.6, 127.3, 126.2, 115.6, 115.4, 70.3, 62.4, 62.1, 52.8, 52.7, 51.8, 47.8, 33.3.

HRMS (ESI-TOF) m/z: $[M+H]^+$ $C_{29}H_{29}FNO_5$ Calcd.490.2024, Found 490.2053.

Dimethyl -2-(2-(3-benzoyl-3-phenylazetidin-1-yl)-2-(4-chlorophenyl)ethyl)malonate (13)

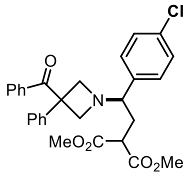

Following the general procedure 1, reaction between D-A cyclopropane (**1k**, 0.027 g, 0.1 mmol) and azabicyclo[1.1.0]butyl carbinols (**2a**, 0.024 g, 0.1 mmol) delivered compound **13**, which was purified by silica gel column chromatography (EtOAc: Hexane 2:8) to furnish the title compound **13** as a brown liquid in 86% (0.043 g) yield. R_f 0.2 (EtOAc: Hexane 2:8)

^{1}H NMR (400 MHz, $CDCl_3$) δ 7.56 (d, J = 7.3 Hz, 2H), 7.39 (d, J = 7.6 Hz, 2H), 7.33 (t, J = 7.4 Hz, 1H), 7.25 – 7.23 (m, 1H), 7.21 – 7.16 (m, 5H), 7.14 – 7.04 (m, 3H), 3.75 – 3.70 (m, 2H), 3.57 (s, 3H), 3.56 – 3.53 (m, 1H), 3.51 – 3.47 (m, 4H), 3.22 – 3.19 (m, 1H), 3.04 – 2.98 (m, 1H), 2.22 – 2.15 (m, 1H), 2.07 – 2.00 (m, 1H). **^{13}C {^{1}H} NMR** (101 MHz, $CDCl_3$) δ 199.41, 169.7, 169.6, 141.3, 137.7, 133.9, 133.6, 133.0, 129.7, 129.6, 129.2, 128.8, 128.6, 127.3, 126.2, 70.4, 62.3, 62.0, 52.7, 52.6, 51.8, 47.7, 33.2.

HRMS (ESI-TOF) m/z: $[M+H]^+$ $C_{29}H_{29}ClNO_5$ Calcd.506.1729, Found 506.1757

Dimethyl (R)-2-(2-(3-benzoyl-3-phenylazetidin-1-yl)-2-(furan-2-yl)ethyl)malonate (16)

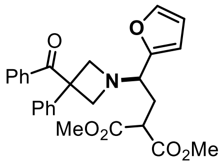

Following the general procedure 1, reaction between D-A cyclopropane (**1l**, 0.022 g, 0.1 mmol) and azabicyclo[1.1.0]butyl carbinols (**2a**, 0.024 g, 0.1 mmol) delivered compound **16**, which was purified by silica gel column chromatography (EtOAc: Hexane 2:8) to furnish the title compound **16** as a yellow liquid in 80% (0.037 g) yield. R_f 0.2 (EtOAc: Hexane 2:8)

^{1}H NMR (400 MHz, $CDCl_3$) δ 7.61 – 7.51 (m, 2H), 7.36 – 7.32(m, 3H), 7.27 – 7.21 (m, 5H), 7.18 – 7.12 (m, 1H), 6.22 – 6.21 (m, 1H), 6.12 (d, J = 3.1 Hz, 1H), 3.81 – 3.71 (m, 3H), 3.62 – 3.59 (m, 1H), 3.58 (s, 3H), 3.56 (s, 3H), 3.45 – 3.42 (m, 1H), 3.31 – 3.22 (m, 1H), 2.25 – 2.12 (m, 2H). **^{13}C {^{1}H} NMR** (100 MHz, $CDCl_3$) δ 199.4, 169.8, 169.7, 152.2, 142.5, 141.2, 134.0, 132.9, 129.7, 129.1, 128.5, 127.2, 126.2, 110.1, 108.8, 62.3, 61.3, 60.9, 52.7, 52.0, 48.4, 30.4.

HRMS (ESI-TOF) m/z: $[M+H]^+$ $C_{27}H_{28}NO_6$ Calcd.462.1911, Found 462.1927.

Dimethyl -2-(2-(3-benzoyl-3-phenylazetidin-1-yl)-2-(thiophen-2-yl)ethyl)malonate (17)

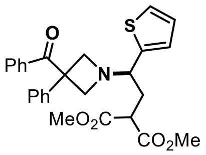

Following the general procedure 1, reaction between D-A cyclopropane (**1m**, 0.024 g, 0.1 mmol) and azabicyclo[1.1.0]butyl carbinols (**2a**, 0.024 g, 0.1 mmol) delivered compound **17**, which was purified by silica gel column chromatography (EtOAc: Hexane 2:8) to furnish the title compound **17** as a colourless liquid in 90% (0.043 g) yield. R_f 0.2 (EtOAc: Hexane 2:8)

^{1}H NMR (400 MHz, DMSO) δ 7.69 – 7.65 (m, 2H), 7.53 (t, *J* = 7.4 Hz, 1H), 7.48 – 7.45 (m, 3H), 7.42 – 7.35 (m, 4H), 7.28 – 7.23 (m, 1H), 6.98 – 6.96 (m, 2H), 3.84 (d, *J* = 7.3 Hz, 1H), 3.74 (d, *J* = 7.4 Hz, 1H), 3.69 – 3.63 (m, 3H), 3.61 (s, 3H), 3.50 (s, 3H), 3.22 – 3.18 (m, 1H), 2.27 – 2.20 (m, 1H), 1.91 – 1.84 (m, 1H). **^{13}C {^{1}H} NMR** (101 MHz, DMSO) δ 198.5, 169.0(2), 142.3, 140.9, 133.4, 133.1, 129.3, 129.0, 128.8, 127.1, 126.6, 126.5, 126.1, 126.0, 64.6, 61.1, 60.8, 52.6, 52.5, 51.3, 47.9, 33.6.

HRMS (ESI-TOF) m/z: $[M+H]^+$ $C_{27}H_{28}NO_5S$ Calcd.478.1683, Found 478.1686.

Dimethyl -2-(2-(3-benzoyl-3-phenylazetidin-1-yl)-2-(1-tosyl-1H-indol-3-yl)ethyl)malonate (18)

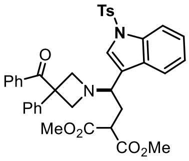

Following the general procedure 1, reaction between D-A cyclopropane (**1n**, 0.043 g, 0.1 mmol) and azabicyclo[1.1.0]butyl carbinols (**2a**, 0.024 g, 0.1 mmol) delivered compound **18**, which was purified by silica gel column chromatography (EtOAc: Hexane 2:8) to furnish the title compound **18** as a reddish liquid in 97% (0.064 g) yield. R_f 0.2 (EtOAc: Hexane 2:8)

^{1}H NMR (400 MHz, $CDCl_3$) δ 7.94 (d, *J* = 8.3 Hz, 1H), 7.77 – 7.72 (m, 2H), 7.69 – 7.61 (m, 3H), 7.50 – 7.40 (m, 4H), 7.38 – 7.28 (m, 6H), 7.23 – 7.16 (m, 3H), 3.87 (d, *J* = 7.5 Hz, 1H), 3.78 (d, *J* = 7.5 Hz, 1H), 3.66 – 3.61 (m, 2H), 3.60 – 3.57 (m, 4H), 3.54 (s, 3H), 3.07 (t, *J* = 7.2 Hz, 1H), 2.31 (s, 3H), 2.29 – 2.24 (m, 2H). **^{13}C {^{1}H} NMR** (101 MHz, $CDCl_3$) δ 199.5, 169.6, 145.0, 141.4, 135.7, 134.9, 134.0, 133.1, 130.0, 129.7, 129.4, 129.2, 128.6, 127.3, 127.0, 126.2, 125.3, 125.1, 123.5, 120.7, 120.2, 114.0, 63.4, 62.7, 62.1, 52.7, 52.6, 51.7, 48.1, 31.0, 21.7.

HRMS (ESI-TOF) m/z: $[M+H]^+$ $C_{38}H_{37}N_2O_7S$ Calcd.665.2316, Found 665.2342.

Dimethyl -2-(2-(3-benzoyl-3-phenylazetidin-1-yl)-2-(naphthalen-2-yl)ethyl)malonate (19)

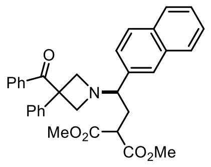

Following the general procedure 1, reaction between D-A cyclopropane (**1o**, 0.028 g, 0.1 mmol) and azabicyclo[1.1.0]butyl carbinols (**2a**, 0.024 g, 0.1 mmol) delivered compound **19**, which was purified by silica gel column chromatography (EtOAc: Hexane 2:8) to furnish the title compound **19** as a colourless liquid in 72% (0.038 g) yield. R_f 0.2 (EtOAc: Hexane 2:8)

^{1}H NMR (400 MHz, $CDCl_3$) δ 7.74 – 7.70(m, 3H), 7.60 – 7.55 (m, 3H), 7.43 – 7.41 (m, 2H), 7.40 – 7.36 (m, 3H), 7.34 – 7.29 (m, 2H), 7.27 – 7.24 (m, 1H), 7.22 – 7.17 (m, 3H), 3.84 – 3.76 (m, 2H), 3.65 – 3.60 (m, 1H), 3.56 – 3.55 (m, 4H), 3.45 (s, 3H), 3.41 – 3.38 (m, 1H), 3.12 – 3.08 (m, 1H), 2.33 – 2.27 (m, 1H), 2.20 – 2.13 (m, 1H). **^{13}C {^{1}H} NMR** (100 MHz, $CDCl_3$) δ 199.6, 169.8, 169.7, 141.5, 136.7, 134.0, 133.3, 133.0, 129.7, 129.2, 128.6, 128.5, 128.0, 127.8, 127.7, 127.5, 127.2, 126.2(2), 126.1, 125.8, 71.3, 62.6, 62.2, 52.7, 52.6, 51.9, 48.0, 33.1.

HRMS (ESI-TOF) m/z: $[M+Na]^+$ $C_{33}H_{31}NaNO_5$ Calcd.544.2094, Found 544.2135.

Dimethyl-2-(2-(3-benzoyl-3-phenylazetidin-1-yl)but-3-en-1-yl)malonate (20)

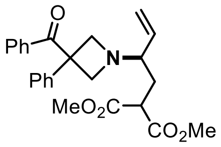

Following the general procedure 1, reaction between D-A cyclopropane (**1s**, 0.018 g, 0.1 mmol) and azabicyclo[1.1.0]butyl carbinols (**2a**, 0.024 g, 0.1 mmol) delivered compound **20**, which was purified by silica gel column chromatography (EtOAc: Hexane 2:8) to furnish the title compound **20** as a yellow gummy liquid in 86% (0.036 g) yield (dr ratio 1:1) . R_f 0.2 (EtOAc: Hexane 2:8)

^{1}H NMR (400 MHz, DMSO-d_6) δ 7.72 – 7.66 (m, 2H), 7.58 – 7.51 (m, 1H), 7.50 – 7.46 (m, 2H), 7.44 – 7.40 (m, 2H), 7.39 – 7.34 (m, 2H), 7.28 – 7.23 (m, 1H), 5.54 – 5.45 (m, 1H), 5.21 – 5.14 (m, 2H), 3.76 (d, J = 7.3 Hz, 1H), 3.68 – 3.63 (m, 3H), 3.61 (s, 3H), 3.55 (s, 3H), 3.39 (s, 1H), 2.71 – 2.66 (m, 1H), 1.98 – 1.91 (m, 1H), 1.79 – 1.72 (m, 1H). **^{13}C {^{1}H} NMR** (101 MHz, DMSO-d_6) δ 198.7, 169.3, 140.9, 136.4, 133.3, 133.2, 129.2, 128.8, 128.5, 127.7, 127.1, 126.2, 119.2, 68.2, 60.8, 60.5, 52.5, 52.3, 51.6, 47.5, 30.4.

HRMS (ESI-TOF) m/z: $[M+H]^+$ $C_{25}H_{28}NO_5$ Calcd.422.1962, Found 422.1964.

Dimethyl -2-(2-(3-benzoyl-3-phenylazetidin-1-yl)-4-phenylbut-3-en-1-yl)malonate (21)

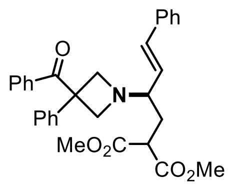

Following the general procedure 1, reaction between D-A cyclopropane (**1t**, 0.026 g, 0.1 mmol) and azabicyclo[1.1.0]butyl carbinols (**2a**, 0.024 g, 0.1 mmol) delivered compound **21**, which was purified by silica gel column chromatography (EtOAc: Hexane 2:8) to furnish the title compound **21** as a colourless liquid in 96% (0.048 g) yield. R_f 0.2 (EtOAc: Hexane 2:8)

^{1}H NMR (600 MHz, DMSO-d_6) δ 7.71 – 7.67 (m, 2H), 7.54 – 7.51 (m, 1H), 7.50 – 7.48 (m, 2H), 7.42 – 7.39 (m, 4H), 7.38 – 7.35 (m, 2H), 7.32 – 7.30 (m, 2H), 7.26 – 7.22 (m, 2H), 6.53 (d, *J* = 15.9 Hz, 1H), 5.97 (dd, *J* = 16.0, 8.4 Hz, 1H), 3.80 (d, *J* = 7.3 Hz, 1H), 3.74 – 3.70 (m, 3H), 3.60 (s, 3H), 3.48 (s, 3H), 3.47 – 3.44 (m, 1H), 2.89 – 2.86 (m, 1H), 2.06 – 2.02 (m, 1H), 1.90 – 1.85 (m, 1H). **^{13}C {^{1}H} NMR** (151 MHz, DMSO-d_6) δ 198.7, 169.4, 140.9, 136.3, 133.3(2), 133.2, 129.2, 129.0, 128.8, 128.6, 127.9, 127.8, 127.0, 126.5, 126.2, 67.9, 61.0, 60.6, 52.5, 52.3, 51.7, 47.7, 30.8.

HRMS (ESI-TOF) m/z: $[M+H]^+$ $C_{31}H_{32}NO_5$ Calcd.498.2275, Found 498.2300.

Diethyl-2-(2-(3-benzoyl-3-phenylazetidin-1-yl)-2-(4-methoxyphenyl)ethyl)malonate (22)

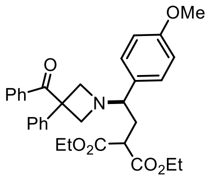

Following the general procedure 1, reaction between D-A cyclopropane (**1p**, 0.029 g, 0.1 mmol) and azabicyclo[1.1.0]butyl carbinols (**2a**, 0.024 g, 0.1 mmol) delivered compound **22**, which was purified by silica gel column chromatography (EtOAc: Hexane 2:8) to furnish the title compound **22** as a yellow liquid in 89% (0.047 g) yield. R_f 0.2 (EtOAc: Hexane 2:8)

^{1}H NMR (600 MHz, DMSO-d_6) δ 7.69 – 7.64 (m, 2H), 7.53 – 7.49 (m, 1H), 7.48 – 7.44 (m, 2H), 7.40 – 7.35 (m, 4H), 7.26 – 7.23 (m, 1H), 7.16 (d, *J* = 8.5 Hz, 2H), 6.90 – 6.86 (m, 2H), 4.07 – 4.03 (m, 2H), 3.94 – 3.88 (m, 2H), 3.79 (d, *J* = 7.4 Hz, 1H), 3.72 (s, 3H), 3.68 (d, *J* = 7.4 Hz, 1H), 3.58 – 3.52 (m, 2H), 3.23 – 3.21 (m, 1H), 2.98 (dd, *J* = 8.4, 6.1 Hz, 1H), 2.18 – 2.10 (m, 1H), 1.94 – 1.89 (m, 1H), 1.12 (t, *J* = 7.1 Hz, 3H), 1.06 (t, *J* = 7.1 Hz, 3H). **^{13}C {^{1}H} NMR** (151 MHz, DMSO-d_6) δ 198.7, 168.6(2), 158.7, 141.0, 133.3,133.2, 130.9, 129.2 (2), 129.0, 128.8, 127.0, 126.1, 113.8, 69.38, 61.76, 61.20, 61.05, 60.95, 54.99, 51.35, 47.82, 32.63, 13.87, 13.74.

HRMS (ESI-TOF) m/z: $[M+H]^+$ $C_{32}H_{36}NO_6$ Calcd.530.2537, Found 530.2565.

Di-tert-butyl-2-(2-(3-benzoyl-3-phenylazetidin-1-yl)-2-(4-methoxyphenyl)ethyl) malonate (23)

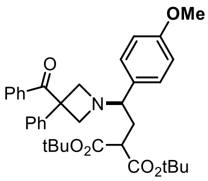

Following the general procedure 1, reaction between D-A cyclopropane (**1q**, 0.035 g, 0.1 mmol) and azabicyclo[1.1.0]butyl carbinols (**2a**, 0.024 g, 0.1 mmol) delivered compound **23**, which was purified by silica gel column chromatography (EtOAc: Hexane 2:8) to furnish the title compound **23** as a yellow liquid in 85% (0.050 g) yield. R_f 0.2 (EtOAc: Hexane 2:8)

^{1}H NMR (400 MHz, $CDCl_3$) δ 7.65 – 7.59 (m, 2H), 7.47 – 7.43 (m, 2H), 7.41 – 7.36 (m, 1H), 7.33 – 7.30(m, 2H), 7.28 (d, *J* = 3.2 Hz, 1H), 7.23 (s, 1H), 7.20 – 7.16 (m, 3H), 6.82 – 6.80 (m, 2H), 3.81 (s, 2H), 3.75 (s, 3H), 3.66 (d, *J* = 7.8 Hz, 1H), 3.56 (d, *J* = 7.8 Hz, 1H), 3.14 (dd, *J* = 10.2, 3.3 Hz, 1H), 2.80 (dd, *J* = 10.6, 4.3 Hz, 1H), 2.24 – 2.17 (m, 1H), 1.91 – 1.84 (m, 1H), 1.41 (s, 9H), 1.33 (s, 9H). **^{13}C {^{1}H} NMR** (100 MHz, $CDCl_3$) δ 199.7, 168.8, 168.7, 159.2, 141.5, 134.1, 132.9, 129.7, 129.5, 129.1, 128.5, 127.2, 126.3, 114.0, 81.6, 81.5, 70.7, 62.5, 62.0, 55.3, 51.9, 50.6, 33.4, 28.1, 28.0.
HRMS (ESI-TOF) m/z: $[M+H]^+$ $C_{36}H_{44}NO_6$ Calcd.586.3163, Found 586.3191.

1-(tert-butyl) 3-ethyl 2-((R)-2-(3-benzoyl-3-phenylazetidin-1-yl)-2-(4-methoxyphenyl) ethyl) malonate (24)

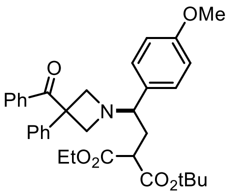

Following the general procedure 1, reaction between D-A cyclopropane (**1r**, 0.032 g, 0.1 mmol) and azabicyclo[1.1.0]butyl carbinols (**2a**, 0.024 g, 0.1 mmol) delivered compound **24**, which was purified by silica gel column chromatography (EtOAc: Hexane 2:8) to furnish the title compound **24** as a yellow liquid in 95% (0.053 g) yield (dr ratio 1:1) . R_f 0.2 (EtOAc: Hexane 2:8)

^{1}H NMR (400 MHz, $CDCl_3$) δ 7.57 (d, *J* = 7.7 Hz, 3H), 7.42 – 7.39 (m, 3H), 7.33 (t, *J* = 7.4 Hz, 2H), 7.27 – 7.23 (m, 4H), 7.22 – 7.20 (m, 4H), 7.18 – 7.16 (m, 2H), 7.15 – 7.09 (m, 5H), 6.79 – 6.73 (m, 4H), 4.16 – 4.00 (m, 2H), 3.99 – 3.90 (m, 2H), 3.77 – 3.72 (m, 3H), 3.72 – 3.66 (m, 6H), 3.62 – 3.56 (m, 2H), 3.51 – 3.49 (m, 2H), 3.17 – 3.07 (m, 2H), 2.94 – 2.82 (m, 2H), 2.25 – 2.12 (m, 2H), 1.97 – 1.85 (m, 2H), 1.34 (s, 9H), 1.29 – 1.27 (m, 9H), 1.18 – 1.14 (m, 3H), 1.11 – 1.07 (m, 3H). **^{13}C {^{1}H} NMR** (101 MHz, $CDCl_3$) δ 199.6(2), 169.8, 169.7, 168.5, 168.4, 159.2(2), 141.5(2), 134.1, 134.0, 132.9, 131.42 (2), 129.6 (2), 129.4, 129.1 (2), 128.5, 128.3(2), 127.5(3), 127.4, 127.1, 126.2, 113.9, 81.9, 81.8, 70.6, 70.5, 62.5, 62.4, 62.1, 62.0, 61.2(2), 55.3, 51.8 (2), 49.6, 49.4, 33.3, 33.2, 28.0, 27.9, 14.2, 14.1

HRMS (ESI-TOF) m/z: $[M+Na]^+$ $C_{34}H_{39}NaNO_6$ Calcd.580.2670, Found 580.2700.

Dimethyl 2-(2-(3-benzoyl-3-(3,4,5-trimethoxyphenyl)azetidin-1-yl)-2-(4-methoxyphenyl)ethyl)malonate (25)

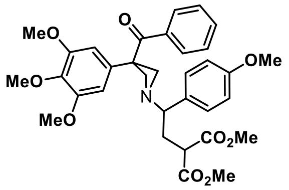

Following the general procedure 1, reaction between D-A cyclopropane (**1a**, 0.027 g, 0.1 mmol) and azabicyclo[1.1.0]butyl carbinols (**2j**, 0.033 g, 0.1 mmol) delivered compound **25**, which was purified by silica gel column chromatography (EtOAc: Hexane 2:8) to furnish the title compound **25** as a pale yellow liquid in 87% (0.056 g) yield. R_f 0.2 (EtOAc: Hexane 2:8)

1H NMR (400 MHz, DMSO) δ 7.75 (d, *J* = 7.4 Hz, 2H), 7.54 (t, *J* = 7.3 Hz, 1H), 7.42 (t, *J* = 7.7 Hz, 2H), 7.15 (d, *J* = 8.5 Hz, 2H), 6.89 (d, *J* = 8.6 Hz, 2H), 6.69 (s, 2H), 3.80 (d, *J* = 7.0 Hz, 1H), 3.74 – 3.72 (m, 9H), 3.68 – 3.62 (m, 2H), 3.60 – 3.56 (m, 7H), 3.45 (s, 3H), 3.23 – 3.21 (m, 1H), 3.05 (t, *J* = 7.2 Hz, 1H), 2.18 – 2.12 (m, 1H), 2.00 – 1.88 (m, 1H). **^{13}C {^{1}H} NMR** (101 MHz, DMSO) δ 198.4, 169.1 (2), 158.7, 153.1, 136.4, 136.2, 133.3, 133.2, 130.8, 129.3, 129.2, 128.8, 113.8, 103.7, 69.2, 61.5, 61.2, 60.0, 56.0, 55.0, 52.5, 52.3, 51.7, 47.5, 32.7.

HRMS (ESI-TOF) m/z: $[M+H]^+$ $C_{33}H_{38}NO_9$ Calcd.592.2541, Found 592.2556.

Dimethyl 2-(2-(4-methoxyphenyl)-2-(3-phenyl-3-(4-(trifluoromethyl)benzoyl)azetidin-1-yl)ethyl)malonate (26)

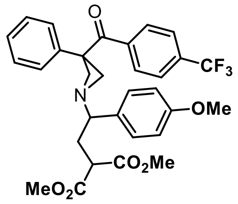

Following the general procedure 1, reaction between D-A cyclopropane (**1a**, 0.027 g, 0.1 mmol) and azabicyclo[1.1.0]butyl carbinols (**2l**, 0.032 g, 0.1 mmol) delivered compound **26**, which was purified by silica gel column chromatography (EtOAc: Hexane 2:8) to furnish the title compound **26** as a gummy liquid in 83% (0.047 g) yield. R_f 0.2 (EtOAc: Hexane 2:8)

^{1}H NMR (400 MHz, DMSO) δ 7.85 (d, *J* = 8.2 Hz, 2H), 7.76 (d, *J* = 8.4 Hz, 2H), 7.46 (d, *J* = 7.3 Hz, 2H), 7.38 (t, *J* = 7.8 Hz, 2H), 7.26 (t, *J* = 7.9 Hz, 1H), 7.16 (d, *J* = 8.6 Hz, 2H), 6.89 (d, *J* = 8.6 Hz, 2H), 3.79 – 3.68 (m, 5H), 3.61 – 3.55 (m, 5H), 3.46 (s, 3H), 3.28 – 3.25 (m 1H), 3.04 (t, *J* = 7.2 Hz, 1H), 2.18 – 2.10 (m, 1H), 1.98 – 1.91 (m, 1H). **^{13}C {^{1}H} NMR** (101 MHz, DMSO) δ 197.8, 169.1, 158.7, 140.3, 136.5, 134. 2 (q, J= 250 Hz),130.7, 130.0, 129.2, 128.0, 127.9 (q, J= 33Hz), 127.3, 126.2, 125.8 (q, J = 18 Hz) 113.8, 69.1, 61.5, 60.8, 55.0, 52.5, 52.3, 51.5, 47.4, 32.7.

HRMS (ESI-TOF) m/z: $[M+H]^+$ $C_{31}H_{31}F_3NO_6$ Calcd. 570.2098, Found 570.2124.

Dimethyl 2-(2-(3-benzoyl-3-(2-chlorophenyl)azetidin-1-yl)-2-(4-methoxyphenyl) ethyl)malonate (27)

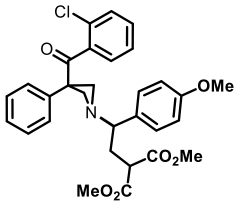

Following the general procedure 1, reaction between D-A cyclopropane (**1a**, 0.027 g, 0.1 mmol) and azabicyclo[1.1.0]butyl carbinols (**2k**, 0.027 g, 0.1 mmol) delivered compound **27**, which was purified by silica gel column chromatography (EtOAc: Hexane 2:8) to furnish the title compound **27** as a colourless liquid in 87% (0.043 g) yield. R_f 0.2 (EtOAc: Hexane 2:8)

^{1}H NMR (400 MHz, DMSO) δ 7.46 – 7.31 (m, 4H), 7.27 – 7.15 (m, 4H), 7.13 – 7.11 (m, 2H), 7.06 – 7.04 (m, 1H), 6.88 (d, *J* = 8.6 Hz, 2H), 3.80 – 3.66 (m, 5H), 3.61 – 3.52 (m, 4H), 3.50 – 3.38 (m, 4H), 3.24 – 3.21 (m, 1H), 3.07 – 3.04 (m, 1H), 2.17 – 2.09 (m, 1H), 1.95 – 1.88 (m, 1H).
^{13}C {^{1}H} NMR (101 MHz, DMSO) δ 201.5, 169.1, 169.0, 158.7, 139.7, 137.0, 131.6, 130.6, 130.2, 129.1, 128.6, 128.0, 127.0, 126.7, 118.8, 115.2, 113.8, 69.0, 61.0, 60.9, 55.0, 53.5, 52.4, 52.3, 47.4, 32.7.

HRMS (ESI-TOF) m/z: $[M+H]^+$ $C_{30}H_{31}ClNO_6$ Calcd. 536.1834, Found 536.1856.

Dimethyl 2-(2-(3-acetyl-3-(naphthalen-1-yl)azetidin-1-yl)-2-(4-methoxyphenyl)ethyl) malonate (28)

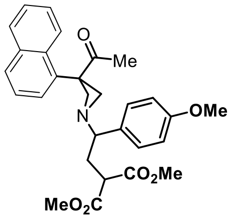

Following the general procedure 1, reaction between D-A cyclopropane (**1a**, 0.027 g, 0.1 mmol) and azabicyclo[1.1.0]butyl carbinols (**2i**, 0.023 g, 0.1 mmol) delivered compound **28**, which was purified by silica gel column chromatography (EtOAc: Hexane 2:8) to furnish the title compound **28** as a reddish liquid in 87% (0.043 g) yield. R_f 0.2 (EtOAc: Hexane 2:8)

^{1}H NMR (400 MHz, DMSO-d_6) δ 7.98 – 7.87 (m, 3H), 7.82 (s, 1H), 7.56 – 7.47 (m, 2H), 7.30 (dd, *J* = 8.5, 1.6 Hz, 1H), 7.14 (d, *J* = 8.6 Hz, 2H), 6.90 (d, *J* = 8.6 Hz, 2H), 3.91 (d, *J* = 7.0 Hz, 1H), 3.74 (s, 3H), 3.71 – 3.67 (m, 1H), 3.59 (s, 3H), 3.48 (s, 3H), 3.41 – 3.38 (m, 2H), 3.18 (dd, *J* = 8.4, 3.1 Hz, 1H), 3.04 (dd, *J* = 8.1, 6.3 Hz, 1H), 2.20 – 2.13 (m, 1H), 2.05 (s, 3H), 1.99 – 1.89 (m, 1H). **^{13}C {^{1}H} NMR** (101 MHz, DMSO-d_6) δ 206.6, 169.1, 158.7, 137.8, 132.9, 131.9, 130.8, 129.2, 128.4, 127.8, 127.5, 126.5, 126.1, 125.2, 124.5, 113.8, 69.4, 60.4, 60.3, 55.0, 53.9, 52.4, 52.3, 47.5, 32.7, 24.8.

HRMS (ESI-TOF) m/z: $[M+H]^+$ $C_{29}H_{32}NO_6$ Calcd. 490.2224, Found 490.2226.

Dimethyl -2-(2-(3-acetyl-3-(furan-2-yl)azetidin-1-yl)-2-(4-methoxyphenyl)ethyl)malonate (29)

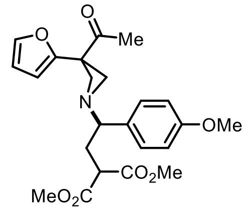

Following the general procedure 1, reaction between D-A cyclopropane (**1a**, 0.027 g, 0.1 mmol) and azabicyclo[1.1.0]butyl carbinols (**2e**, 0.017 g, 0.1 mmol) delivered compound **29**, which was purified by silica gel column chromatography (EtOAc: Hexane 2:8) to furnish the title compound **29** as a yellow liquid in 70% (0.030 g) yield. R_f 0.2 (EtOAc: Hexane 2:8)

^{1}H NMR (400 MHz, Chloroform-*d*) δ 7.37 (dd, *J* = 1.9, 0.8 Hz, 1H), 7.17 (d, *J* = 8.2 Hz, 2H), 6.85 (d, *J* = 8.7 Hz, 2H), 6.37 (dd, *J* = 3.3, 1.8 Hz, 1H), 6.25 (d, *J* = 3.3 Hz, 1H), 3.78 (s, 3H), 3.72 (d, *J* = 13.7 Hz, 1H), 3.68 (s, 3H), 3.66 – 3.62 (m, 1H), 3.59 (s, 3H), 3.53 (s, 1H), 3.47 – 3.41 (m, 1H), 3.27 (d, *J* = 2.9 Hz, 1H), 3.11 (dd, *J* = 8.7, 5.9 Hz, 1H), 2.29 – 2.21 (m, 1H), 2.16 – 2.09 (m, 1H), 2.05 (s, 3H) **^{13}C {^{1}H} NMR** (101 MHz, Chloroform-*d*) δ 204.7, 169.8, 169.7, 159.4, 153.2, 142.6, 129.5, 128.4, 114.1, 110.9, 107.2, 70.1, 59.2, 55.4, 52.7, 52.7, 49.1, 47.9, 33.0, 25.8.

HRMS (ESI-TOF) m/z: $[M+H]^+$ $C_{23}H_{28}NO_7$ Calcd. 430.1860, Found 430.1874.

Dimethyl 2-(2-(3-acetyl-3-(thiophen-2-yl)azetidin-1-yl)-2-(4-methoxyphenyl)ethyl)malonate (30)

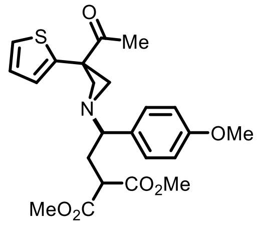

Following the general procedure 1, reaction between D-A cyclopropane (**1a**, 0.027 g, 0.1 mmol) and azabicyclo[1.1.0]butyl carbinols (**2f**, 0.018 g, 0.1 mmol) delivered compound **30**, which was purified by silica gel column chromatography (EtOAc: Hexane 2:8) to furnish the title compound **30** as a colourless liquid in 98% (0.043 g) yield. R_f 0.2 (EtOAc: Hexane 2:8)

^{1}H NMR (400 MHz, DMSO-*d*$_6$) δ 7.47 (dd, *J* = 4.4, 1.9 Hz, 1H), 7.15 – 7.12 (m, 2H), 7.03 – 7.00 (m, 2H), 6.91 – 6.88 (m, 2H), 3.73 (s, 3H), 3.66 (d, *J* = 7.1 Hz, 1H), 3.60 (s, 3H), 3.57 (d, *J* = 7.3 Hz, 1H), 3.48 (s, 3H), 3.29 – 3.19 (m, 3H), 3.05 (dd, *J* = 8.1, 6.2 Hz, 1H), 2.16 – 2.09 (m, 1H), 2.05 (s, 3H), 1.98 –1.89 (m, 1H). **^{13}C {^{1}H} NMR** (101 MHz, DMSO-*d*$_6$) δ 205.4, 169.1, 158.7, 143.7, 130.6, 129.1, 127.4, 125.6, 125.5, 113.8, 69.0, 61.4(2), 55.0, 52.5, 52.3, 50.4, 47.5, 32.7, 24.7.

HRMS (ESI-TOF) m/z: $[M+H]^+$ $C_{23}H_{28}NO_6S$ Calcd.446.1632, Found 446.1654.

Dimethyl 2-(2-(3-acetyl-3-(benzo[b]thiophen-2-yl)azetidin-1-yl)-2-(4-methoxyphenyl)ethyl) malonate (31)

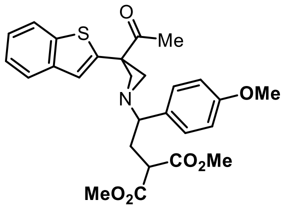

Following the general procedure 1, reaction between D-A cyclopropane (**1a**, 0.027 g, 0.1 mmol) and azabicyclo[1.1.0]butyl carbinols (**2g**, 0.023 g, 0.1 mmol) delivered compound **31**, which was purified by silica gel column chromatography (EtOAc: Hexane 2:8) to furnish the title compound **31** as a white liquid in 92% (0.048 g) yield. R_f 0.2 (EtOAc: Hexane 2:8)

^{1}H NMR (400 MHz, DMSO-d_6) δ 7.96 – 7.90 (m, 1H), 7.83 – 7.77 (m, 1H), 7.41 – 7.31 (m, 3H), 7.19 – 7.13 (m, 2H), 6.94 – 6.87 (m, 2H), 3.74 (s, 3H), 3.69 (s, 1H), 3.63 – 3.60 (m, 4H), 3.48 – 3.45 (m, 4H), 3.36 (s, 1H), 3.26 – 3.23 (m, 1H), 3.08 – 3.04 (m, 1H), 2.18 – 2.12 (m, 4H), 2.00 – 1.90 (m, 1H). **^{13}C {^{1}H} NMR** (101 MHz, DMSO-d_6) δ 205.2, 169.1, 158.7, 144.4, 139.4, 138.8, 130.5, 129.1, 124.7, 124.5, 123.6, 122.5, 122.2, 113.8, 69.0, 61.0, 60.9, 55.0, 52.5, 52.3, 51.1, 47.5, 32.7, 25.0.

HRMS (ESI-TOF) m/z: $[M+H]^+$ $C_{27}H_{30}NO_6S$ Calcd.496.1788, Found 496.1791.

Dimethyl 2-(2-(3-acetyl-3-(1-(tert-butoxycarbonyl)-1H-indol-3-yl)azetidin-1-yl)-2-(4-methoxyphenyl)ethyl)malonate (32)

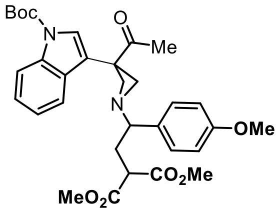

Following the general procedure 1, reaction between D-A cyclopropane (**1a**, 0.027 g, 0.1 mmol) and azabicyclo[1.1.0]butyl carbinols (**2h**, 0.021 g, 0.1 mmol) delivered compound **32**, which was purified by silica gel column chromatography (EtOAc: Hexane 2:8) to furnish the title compound **32** as a reddish liquid in 97% (0.056 g) yield. R_f 0.2 (EtOAc: Hexane 2:8)

^{1}H NMR (400 MHz, DMSO-d_6) δ 8.05 (d, J = 8.4 Hz, 1H), 7.78 (s, 1H), 7.33 – 7.29 (m, 1H), 7.18 – 7.12 (m, 4H), 6.88 (d, J = 8.3 Hz, 2H), 3.87 (d, J = 7.2 Hz, 1H), 3.73 (s, 3H), 3.67 (d, J = 7.4 Hz, 1H), 3.60 (s, 3H), 3.50 (s, 3H), 3.39 (s, 1H), 3.31 (d, J = 7.2 Hz, 1H), 3.20 (dd, J = 8.6, 3.5 Hz, 1H), 3.04 (dd, J = 8.1, 6.3 Hz, 1H), 2.20 – 2.11 (m, 1H), 2.04 (s, 3H), 1.98 – 1.91 (m, 1H), 1.63 (s, 9H). **^{13}C {^{1}H} NMR** (101 MHz, DMSO-d_6) δ 206.3, 169.1, 158.7, 149.0, 135.0, 130.8, 129.2, 128.1, 125.4, 124.7, 123.8, 122.9, 120.1, 119.7, 115.1, 113.8, 84.1, 69.4, 59.8, 59.5, 55.0, 52.5, 52.3, 47.9, 47.5, 32.7, 27.7, 24.5.

HRMS (ESI-TOF) m/z: $[M+H]^+$ $C_{32}H_{39}N_2O_8$ Calcd. 579.2701, Found 579.2705.

Dimethyl -2-(2-(3-benzoyl-3-styrylazetidin-1-yl)-2-(4-methoxyphenyl)ethyl)malonate (33)

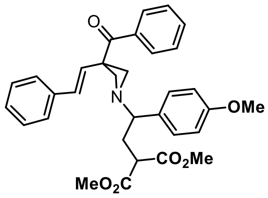

Following the general procedure 1, reaction between D-A cyclopropane (**1a**, 0.027 g, 0.1 mmol) and azabicyclo[1.1.0]butyl carbinols (**2m**, 0.026 g, 0.1 mmol) delivered compound **33**, which was purified by silica gel column chromatography (EtOAc: Hexane 2:8) to furnish the title compound **33** as a yellow liquid in 92% (0.049 g) yield. R_f 0.2 (EtOAc: Hexane 2:8)

^{1}H NMR (400 MHz, DMSO) δ 7.82 – 7.76 (m, 2H), 7.63 – 7.56 (m, 1H), 7.48 (t, *J* = 7.6 Hz, 2H), 7.44 – 7.39 (m, 2H), 7.31 – 7.28 (m, 2H), 7.25 – 7.20 (m, 1H), 7.18 – 7.12 (m, 2H), 6.92 – 6.85 (m, 3H), 6.47 (d, *J* = 16.3 Hz, 1H), 3.73 (s, 3H), 3.64 (d, *J* = 7.1 Hz, 1H), 3.60 (s, 3H), 3.53 – 3.44 (m, 5H), 3.38 (d, *J* = 7.3 Hz, 1H), 3.22 – 3.16 (m, 1H), 3.04 (dd, *J* = 8.4, 6.1 Hz, 1H), 2.13 (td, *J* = 13.5, 6.8 Hz, 1H), 1.99 – 1.89 (m, 1H). **^{13}C {^{1}H} NMR** (101 MHz, DMSO) δ 198.6, 169.1(2), 158.7, 136.2, 133.4, 133.2, 131.2, 130.8, 129.2, 129.1, 128.9, 128.6, 127.8, 126.3, 113.8, 69.2, 61.6, 61.2, 55.0, 52.4, 52.3, 49.3, 47.6, 32.7.

HRMS (ESI-TOF) m/z: $[M+H]^+$ $C_{32}H_{34}NO_6$ Calcd. 528.2381, Found 528.2407.

Dimethyl 2-(2-(3-acetyl-3-phenylazetidin-1-yl)-2-(4-methoxyphenyl)ethyl)malonate (34)

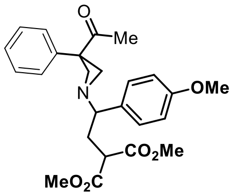

Following the general procedure 1, reaction between D-A cyclopropane (**1a**, 0.027 g, 0.1 mmol) and azabicyclo[1.1.0]butyl carbinols (**2b**, 0.018 g, 0.1 mmol) delivered compound **34**, which was purified by silica gel column chromatography (EtOAc: Hexane 2:8) to furnish the title compound **34** as a colourless liquid in 67% (0.029 g) yield. R_f 0.2 (EtOAc: Hexane 2:8)

^{1}H NMR (400 MHz, Chloroform-*d*) δ 7.35 – 7.31 (m, 2H), 7.27 – 7.23 (m, 1H), 7.18 – 7.13 (m, 4H), 6.85 (d, *J* = 8.7 Hz, 2H), 3.94 (d, *J* = 7.2 Hz, 1H), 3.79 (s, 3H), 3.76 – 3.72 (m, 1H), 3.68 (s, 3H), 3.59 (s, 3H), 3.40 (d, *J* = 7.1 Hz, 1H), 3.34 (d, *J* = 7.3 Hz, 1H), 3.19 – 3.16 (m, 1H), 3.11 (dd, *J* = 8.7, 5.9 Hz, 1H), 2.29 – 2.23 (m, 1H), 2.13 – 2.06 (m, 1H), 2.03 (s, 3H). **^{13}C {^{1}H} NMR** (101 MHz, Chloroform-*d*) δ 207.0, 169.9, 169.8, 159.3, 140.4, 129.4, 129.0, 128.3, 127.3, 126.5, 125.8, 70.5, 60.9, 60.7, 55.4, 54.3, 52.7, 52.6, 47.9, 33.3, 25.0.

HRMS (ESI-TOF) m/z: $[M+H]^+$ $C_{25}H_{30}NO_6$ Calcd. 440.2068, Found 440.2063.

Dimethyl 2-(2-(3-acetyl-3-(4-bromophenyl)azetidin-1-yl)-2-(4-methoxyphenyl)ethyl) malonate (35)

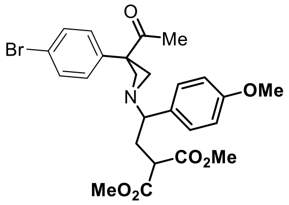

Following the general procedure 1, reaction between D-A cyclopropane (**1a**, 0.027 g, 0.1 mmol) and azabicyclo[1.1.0]butyl carbinols (**2c**, 0.025 g, 0.1 mmol) delivered compound **35**, which was purified by silica gel column chromatography (EtOAc: Hexane 2:8) to furnish the title compound **35** as a brown liquid in 80% (0.041 g) yield. R_f 0.2 (EtOAc: Hexane 2:8)

^{1}H NMR (400 MHz, Chloroform-*d*) δ 7.46 (d, *J* = 8.5 Hz, 2H), 7.13 (d, *J* = 8.6 Hz, 2H), 7.05 (d, *J* = 8.5 Hz, 2H), 6.85 (d, *J* = 8.6 Hz, 2H), 3.88 (d, *J* = 7.1 Hz, 1H), 3.79 (s, 3H), 3.72 – 3.70 (m, 1H), 3.68 (s, 3H), 3.60 (s, 3H), 3.32 (t, *J* = 8.0 Hz, 2H), 3.16 – 3.09 (m, 2H), 2.28 – 2.22 (m, 1H), 2.11 – 2.04 (m, 1H), 2.03 (s, 3H). **^{13}C {^{1}H} NMR** (101 MHz, Chloroform-*d*) δ 206.4, 169.9, 169.8, 159.3, 139.4, 132.1, 130.8, 129.4, 128.3, 121.4, 114.1, 70.5, 60.8, 60.7, 55.4, 54.0, 52.7, 52.6, 47.9, 33.3, 25.0.

HRMS (ESI-TOF) m/z: $[M+H]^+$ $C_{25}H_{29}BrNO_6$ Calcd. 518.1173, Found 518.1149.

Dimethyl 2-(2-(3-acetyl-3-(4-methoxyphenyl)azetidin-1-yl)-2-(4-methoxyphenyl)ethyl) malonate (36)

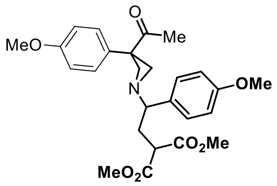

Following the general procedure 1, reaction between D-A cyclopropane (**1a**, 0.027 g, 0.1 mmol) and azabicyclo[1.1.0]butyl carbinols (**2d**, 0.021 g, 0.1 mmol) delivered compound **36**, which was purified by silica gel column chromatography (EtOAc: Hexane 2:8) to furnish the title compound **36** as a yellow liquid in 84% (0.039 g) yield. R_f 0.2 (EtOAc: Hexane 2:8)

^{1}H NMR (800 MHz, DMSO-*d*$_6$) δ 7.14 – 7.11 (m, 4H), 6.90 (dd, *J* = 17.6, 8.2 Hz, 4H), 3.75 – 3.73 (m, 7H), 3.59 (s, 3H), 3.58 – 3.54 (m, 1H), 3.48 (s, 3H), 3.23 (t, *J* = 8.5 Hz, 2H), 3.13 (dd, *J* = 8.7, 3.6 Hz, 1H), 3.02 (dd, *J* = 8.3, 6.2 Hz, 1H), 2.16 – 2.09 (m, 1H), 1.98 (s, 3H), 1.92 – 1.86 (m, 1H). **^{13}C {^{1}H} NMR** (101 MHz, DMSO-*d*$_6$) δ 206.8, 169.1, 158.7, 158.3, 132.2, 130.8, 129.1, 127.6, 114.2, 113.8, 69.4, 60.3(2), 55.1, 55.0, 53.0, 52.4, 52.3, 47.5, 32.7, 24.6.

HRMS (ESI-TOF) m/z: $[M+H]^+$ $C_{26}H_{32}NO_7$ Calcd.470.2173, Found 470.2187.

6. Preparation of spiroepoxy azetidines (37-41)

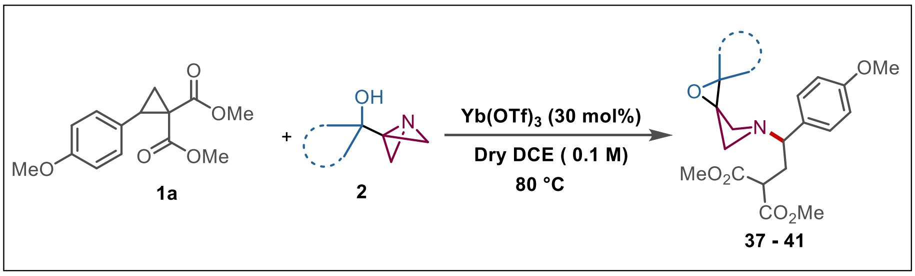

General procedure (2)

To a mixture of Donor–Acceptor Cyclopropanes (1, 1.0 equiv.), ABB–cycloalkanols (2; 1.0 equiv.) and $Yb(OTf)_3$ (30 mol%) was added in dry DCE (0.1 M) in inert atmosphere. The reaction mixture was heated to 80°C in an oil bath and stirred until complete disappearance of the starting material was observed (*ca.* 12 h; TLC monitored). After cooling to room temperature, the mixture was diluted with H_2O and extracted in DCM. Combined organic layers were finally washed with brine, dried over anhydrous Na_2SO_4, filtered, and concentrated under reduced pressure. The crude residue was purified by silica-gel flash column chromatography (using EtOAc /hexanes as eluent) to provide desired spiroepoxy azetidines products **(37-41).**

7. Preparation of spiroepoxy azetidines (42-44)

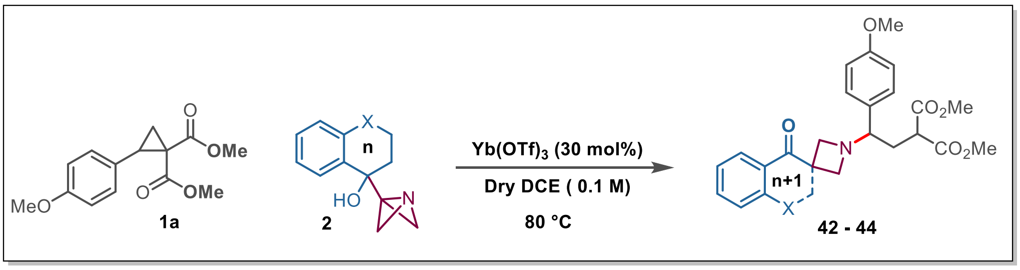

General procedure (3)

To a mixture of Donor–Acceptor Cyclopropanes (1, 1.0 equiv.), ABB–cycloalkanols (2; 1.0 equiv.) and $Yb(OTf)_3$ (30 mol%) was added in dry DCE (0.1 M) in inert atmosphere. The reaction mixture was heated to 80°C in an oil bath and stirred until complete disappearance of the starting material was observed (*ca.* 12 h; TLC monitored). After cooling to room temperature, the mixture was diluted with H_2O and extracted in DCM. Combined organic layers were finally washed with brine, dried over anhydrous Na_2SO_4, filtered, and concentrated under reduced pressure. The crude residue was purified by silica-gel flash column chromatography (using EtOAc /hexanes as eluent) to provide desired spiroepoxy azetidines products **(42-44).**

8. Characterization of compounds (37-44) (from Scheme 2: main text)

***Dimethyl 2-(2-(11-oxa-2-azadispiro[3.0.5^5.1^4]undecan-2-yl)-2-(4-methoxyphenyl)ethyl) malonate. (*37)**

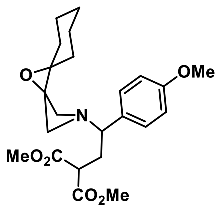

Following the general procedure 2, reaction between D-A cyclopropane (**1a**, 0.027 g, 0.1 mmol) and azabicyclo[1.1.0]butyl cycloalkanols (**2p**, 0.015 g, 0.1 mmol) delivered compound **37**, which was purified by silica gel column chromatography (EtOAc: Hexane 2:8) to furnish the title compound **37** as a colourless liquid in 94% (0.039 g) yield. R_f 0.2 (EtOAc: Hexane 1:9)

^{1}H NMR (400 MHz, $CDCl_3$) δ 7.19 (d, *J* = 8.6 Hz, 2H), 6.85 (d, *J* = 8.7 Hz, 2H), 3.79 (s, 3H), 3.72 (s, 3H), 3.67 – 3.64 (m, 1H), 3.62 (s, 3H), 3.32 – 3.24 (m, 3H), 3.17 – 3.11 (m, 2H), 2.37 – 2.31 (m, 1H), 2.21 – 2.04 (m, 1H), 1.70 – 1.55 (m, 4H), 1.52 – 1.42 (m, 4H), 1.41 – 1.34 (m, 2H). **^{13}C {^{1}H} NMR** (101 MHz, $CDCl_3$) δ 169.8, 169.7, 159.3, 131.4, 129.4, 114.0, 70.8, 64.1, 64.0, 59.0, 58.5, 55.4, 52.7, 52.7, 48.4, 33.9, 31.1(2), 25.4, 24.6.

HRMS (ESI-TOF) m/z: $[M+H]^+$ $C_{23}H_{32}NO_6$ Calcd. 418.2224, Found 418.2236.

***Dimethyl 2-(2-(12-oxa-2-azadispiro[3.0.6^5.1^4]dodecan-2-yl)-2-(4-methoxyphenyl)ethyl) malonate.*(38)**

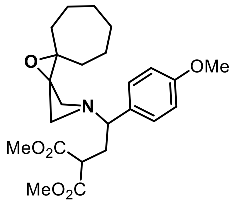

Following the general procedure 2, reaction between D-A cyclopropane (**1a**, 0.027 g, 0.1 mmol) and azabicyclo[1.1.0]butyl cycloalkanols (**2q**, 0.017 g, 0.1 mmol) delivered compound **38**, which was purified by silica gel column chromatography (EtOAc: Hexane 2:8) to furnish the title compound **38** as a yellow liquid in 84% (0.036 g) yield. R_f 0.2 (EtOAc: Hexane 1:9)

^{1}H NMR (400 MHz, $CDCl_3$) δ 7.22 – 7.17 (m, 2H), 6.88 – 6.83 (m, 2H), 3.79 (s, 3H), 3.72 (s, 3H), 3.64 – 3.61 (m, 4H), 3.33 – 3.23 (m, 3H), 3.18 – 3.12 (m, 2H), 2.38 – 2.32 (m, 1H), 2.12 – 2.05 (m, 1H), 1.71 – 1.56 (m, 10H), 1.50 – 1.39 (m, 2H). **^{13}C {^{1}H} NMR** (101 MHz, $CDCl_3$) δ 169.8 (2), 159.4, 131.5, 129.4, 114.1, 70.8, 65.5, 64.8, 59.3, 58.9, 55.4, 52.7, 52.7, 48.4, 34.0, 33.2, 33.2, 29.4, 29.4, 24.2, 24.2.

HRMS (ESI-TOF) m/z: $[M+H]^+$ $C_{24}H_{34}NO_6$ Calcd. 432.2381, Found 432.2401.

Dimethyl 2-(2-(17-oxa-2-azadispiro[3.0.11^5.1^4]heptadecan-2-yl)-2-(4-methoxyphenyl) ethyl)malonate (39)

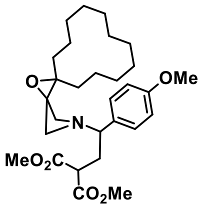

Following the general procedure 2, reaction between D-A cyclopropane (**1a**, 0.027 g, 0.1 mmol) and azabicyclo[1.1.0]butyl cycloalkanols (**2r**, 0.024 g, 0.1 mmol) delivered compound **39**, which was purified by silica gel column chromatography (EtOAc: Hexane 2:8) to furnish the title compound **39** as a yellow liquid in 95% (0.048 g) yield. R_f 0.2 (EtOAc: Hexane 1:9)

^{1}H NMR (400 MHz, DMSO) δ 7.17 (d, *J* = 8.6 Hz, 2H), 6.88 (d, *J* = 8.6 Hz, 2H), 3.73 (s, 3H), 3.62 (s, 3H), 3.53 (s, 3H), 3.48 (d, *J* = 9.0 Hz, 1H), 3.28 – 3.25 (m, 1H), 3.20 (d, *J* = 9.1 Hz, 1H), 3.13 – 3.03 (m, 3H), 2.20 – 2.14 (m, 1H), 1.98 – 1.91 (m, 1H), 1.38 – 1.21 (m, 22H). **^{13}C {^{1}H} NMR** (101 MHz, DMSO) δ 169.1(2), 158.7, 131.3, 129.1, 113.7, 69.3, 63.8, 63.0, 58.3, 58.1, 55.0, 52.4, 52.4, 47.7, 36.2, 33.4, 27.3, 27.3, 25.6, 25.5, 25.0, 22.2, 22.1(2), 20.0.

HRMS (ESI-TOF) m/z: $[M+H]^+$ $C_{29}H_{44}NO_6$ Calcd. 502.3163, Found 502.3188.

Dimethyl 2-(2-(20-oxa-2-azadispiro[3.0.14^5.1^4]icosan-2-yl)-2-(4-methoxyphenyl)ethyl) Malonate (40)

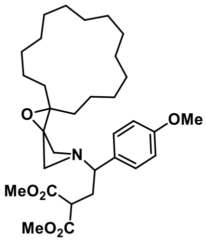

Following the general procedure 2, reaction between D-A cyclopropane (**1a**, 0.027 g, 0.1 mmol) and azabicyclo[1.1.0]butyl cycloalkanols (**2s**, 0.028 g, 0.1 mmol) delivered compound **40**, which was purified by silica gel column chromatography (EtOAc: Hexane 2:8) to furnish the title compound **40** as a pale yellow liquid in 78% (0.042 g) yield. R_f 0.2 (EtOAc: Hexane 2:8)

^{1}H NMR (400 MHz, DMSO) δ 7.17 (d, *J* = 8.5 Hz, 2H), 6.89 (d, *J* = 8.6 Hz, 2H), 3.73 (s, 3H), 3.62 (s, 3H), 3.53 (s, 3H), 3.46 (d, *J* = 8.8 Hz, 1H), 3.27 (d, *J* = 4.6 Hz, 1H), 3.20 (d, *J* = 8.8 Hz, 1H), 3.14 – 3.01 (m, 3H), 2.19 – 2.14 (m, 1H), 1.98 – 1.91 (m, 1H), 1.36 – 1.24 (m, 28H). **^{13}C {^{1}H} NMR** (101 MHz, DMSO) δ 169.1(2), 158.7, 131.2, 129.2, 113.8, 69.2, 63.7, 63.6, 58.2, 58.0, 55.0, 52.5, 52.4, 47.7, 33.4, 30.4, 30.4, 27.1, 27.0, 26.3, 26.1, 26.0, 22.5(2).

HRMS (ESI-TOF) m/z: $[M+H]^+$ $C_{32}H_{50}NO_6$ Calcd. 544.3633, Found 544.3621.

Dimethyl 2-(2-(-dispiro[adamantane-2,2'-oxirane-3',3''-azetidin]-1''-yl)-2-(4-methoxyphenyl)ethyl)malonate (41)

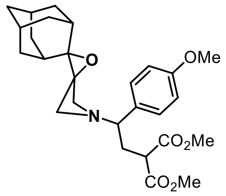

Following the general procedure 2, reaction between D-A cyclopropane (**1a**, 0.027 g, 0.1 mmol) and azabicyclo[1.1.0]butyl cycloalkanols (**2t**, 0.021 g, 0.1 mmol) delivered compound **41**, which was purified by silica gel column chromatography (EtOAc: Hexane 2:8) to furnish the title compound **41** as a white liquid in 76% (0.036 g) yield. R_f 0.2 (EtOAc: Hexane 2:8)

^{1}H NMR (400 MHz, DMSO) δ 7.19 (d, J = 8.6 Hz, 2H), 6.89 (d, J = 8.6 Hz, 2H), 3.73 (s, 3H), 3.62 (s, 3H), 3.54 (s, 3H), 3.46 (d, J = 9.0 Hz, 1H), 3.33 – 3.30 (m, 1H), 3.20 (d, J = 9.1 Hz, 1H), 3.15 (s, 1H), 3.11 – 3.03 (m, 2H), 2.19 – 2.13 (m, 1H), 2.01 – 1.93 (m, 1H), 1.88 (d, J = 6.1 Hz, 2H), 1.84 – 1.78 (m, 2H), 1.76 – 1.74 (m, 3H), 1.70 – 1.65 (m, 5H), 1.23 – 1.18 (m, 2H). **^{13}C {^{1}H} NMR** (101 MHz, DMSO) δ 169.2, 169.1, 158.7, 131.3, 129.1, 113.8, 69.2, 67.4, 63.8, 57.6, 57.5, 55.0, 52.5, 52.4, 47.6, 35.9, 35.7, 35.7, 34.1, 33.4, 32.6, 32.6, 26.6, 26.3.

HRMS (ESI-TOF) m/z: $[M+H]^+$ $C_{27}H_{36}NO_6$ Calcd.470.2537, Found 470.2557.

Dimethyl 2-(2-(4-methoxyphenyl)-2-(5'-oxo-5',7',8',9'-tetrahydrospiro[azetidine-3,6'-benzo[7]annulen]-1-yl)ethyl)malonate (42)

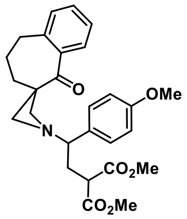

Following the general procedure 3, reaction between D-A cyclopropane (**1a**, 0.027 g, 0.1 mmol) and azabicyclo[1.1.0]butyl cycloalkanols (**2v**, 0.020 g, 0.1 mmol) delivered compound **42**, which was purified by silica gel column chromatography (EtOAc: Hexane 2:8) to furnish the title compound **42** as a yellow liquid in 90% (0.042 g) yield. R_f 0.2 (EtOAc: Hexane 2:8)

^{1}H NMR (400 MHz, DMSO) δ 7.22 – 7.18 (m, 3H), 7.16 – 7.11 (m, 3H), 6.89 (d, J = 8.6 Hz, 2H), 3.96 (d, J = 6.7 Hz, 1H), 3.73 (s, 3H), 3.70 (d, J = 6.9 Hz, 1H), 3.60 (s, 3H), 3.50 (s, 3H), 3.28 – 3.23 (m, 2H), 3.19 – 3.17 (m, 1H), 3.03 (t, J = 7.2 Hz, 1H), 2.84 – 2.63 (m, 4H), 2.17 – 2.11 (m, 1H), 1.96 – 1.91 (m, 1H), 1.87 – 1.68 (m, 2H). **^{13}C {^{1}H} NMR** (101 MHz, DMSO) δ 209.3, 169.2, 169.1, 158.7, 140.9, 137.7, 130.8, 129.9, 129.1, 127.6, 127.0, 126.1, 113.8, 69.2, 61.2, 60.5, 55.0, 54.0, 52.5, 47.4, 32.7, 32.3, 27.8, 20.8, 14.1.

HRMS (ESI-TOF) m/z: $[M+H]^+$ $C_{27}H_{32}NO_6$ Calcd.466.2224, Found 466.2236

Dimethyl 2-(2-(4-methoxyphenyl)-2-(5'-oxo-2',3'-dihydro-5'H-spiro[azetidine-3,4'-benzo[b]oxepin]-1-yl)ethyl)malonate (43)

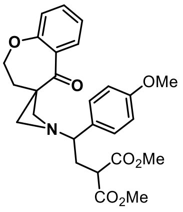

Following the general procedure 3, reaction between D-A cyclopropane (**1a**, 0.027 g, 0.1 mmol) and azabicyclo[1.1.0]butyl cycloalkanols (**2w**, 0.020 g, 0.1 mmol) delivered compound **43**, which was purified by silica gel column chromatography (EtOAc: Hexane 2:8) to furnish the title compound **43** as a yellow liquid in 90% (0.042 g) yield. R_f 0.2 (EtOAc: Hexane 2:8)

^{1}H NMR (400 MHz, DMSO) δ 7.47 (d, *J* = 6.9 Hz, 1H), 7.29 – 7.26 (m, 1H), 7.19 – 7.13 (m, 3H), 7.01 (d, *J* = 7.8 Hz, 1H), 6.89 (d, *J* = 8.6 Hz, 2H), 4.20 – 4.12 (m, 2H), 3.73 (s, 3H), 3.65 (d, *J* = 7.5 Hz, 1H), 3.60 (s, 3H), 3.49 (s, 3H), 3.45 – 3.41 (m, 1H), 3.23 (s, 1H), 3.05 (t, *J* = 7.2 Hz, 1H), 2.96 – 2.89 (m, 2H), 2.87 – 2.79 (m, 1H), 2.73 (s, 1H), 2.18 – 2.10 (m, 1H), 1.99 – 1.89 (m, 1H). **^{13}C {^{1}H} NMR** (101 MHz, DMSO) δ 206.5, 169.1(2), 158.7, 157.4, 131.3, 130.7, 129.1, 129.1, 127.4, 124.3, 121.6, 113.8, 69.4, 69.1, 60.7, 60.1, 55.0, 52.5, 52.3, 51.5, 47.4, 41.4, 32.7.

HRMS (ESI-TOF) m/z: $[M+H]^+$ $C_{26}H_{30}NO_7$ Calcd.468.2017, Found 468.2023.

Dimethyl 2-(2-(4-methoxyphenyl)-2-(11'-oxo-11'H-spiro[azetidine-3,10'-dibenzo[b,f]oxepin]-1-yl)ethyl)malonate (44)

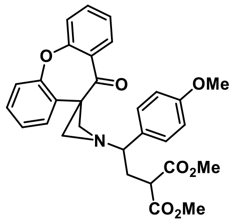

Following the general procedure 3, reaction between D-A cyclopropane (**1a**, 0.027 g, 0.1 mmol) and azabicyclo[1.1.0]butyl cycloalkanols (**2x**, 0.024 g, 0.1 mmol) delivered compound **44**, which was purified by silica gel column chromatography (EtOAc: Hexane 2:8) to furnish the title compound **44** as a brown liquid in 86% (0.044 g) yield. R_f 0.2 (EtOAc: Hexane 2:8)

^{1}H NMR (400 MHz, DMSO) δ 7.99 (dd, *J* = 7.9, 1.6 Hz, 1H), 7.72 – 7.66 (m, 1H), 7.42 (d, *J* = 7.7 Hz, 1H), 7.36 – 7.27 (m, 5H), 7.11 (d, *J* = 8.5 Hz, 2H), 6.86 (d, *J* = 8.6 Hz, 2H), 3.98 (d, *J* = 6.8 Hz, 1H), 3.71 (s, 3H), 3.61 (s, 1H), 3.58 (s, 3H), 3.45 (s, 3H), 3.43 (s, 1H), 3.30 (d, *J* = 7.3 Hz, 1H), 3.20 (dd, *J* = 8.0, 2.8 Hz, 1H), 3.00 (t, *J* = 7.2 Hz, 1H), 2.14 – 2.08 (m, 1H), 1.95 – 1.88 (m, 1H). **^{13}C {^{1}H} NMR** (101 MHz, DMSO) δ 190.4, 169.1, 158.7, 158.5, 155.8, 135.7, 130.8, 130.6, 129.2, 127.0, 126.7, 124.4, 123.1, 121.3, 120.9, 113.8, 69.3, 59.1, 58.5, 55.0, 52.5, 52.3, 51.6, 47.4, 32.7.

HRMS (ESI-TOF) m/z: $[M+H]^+$ $C_{30}H_{30}NO_7$ Calcd.516.2017, Found 516.2020.

9. Preparation of azetidinyl haloketones (45-49)

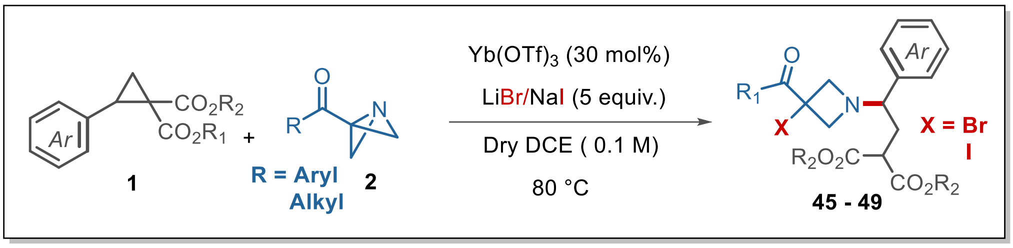

General procedure (4)

To a mixture of Donor–Acceptor Cyclopropanes (1, 1.0 equiv.), azabicyclo[1.1.0]butyl ketone (2;1.0 equiv.), $Yb(OTf)_3$ (30 mol%) and LiBr/NaI (5.0 equiv.) was added in dry DCE (0.1 M) in inert atmosphere. The reaction mixture was heated to 80°C in an oil bath and stirred until complete disappearance of the starting material was observed (*ca*. 12 h; TLC monitored). After cooling to room temperature, the mixture was diluted with H_2O and extracted in DCM. Combined organic layers were finally washed with brine, dried over anhydrous Na_2SO_4, filtered, and concentrated under reduced pressure. The crude residue was purified by silica-gel flash column chromatography (using EtOAc /hexanes as eluent) to provide desired azetidinyl haloketones products **(45 – 49)**

10. Characterization of compounds (45-49) (from Scheme 1: main text)

Dimethyl 2-(2-(3-benzoyl-3-bromoazetidin-1-yl)-2-(4-methoxyphenyl)ethyl)malonate (45)

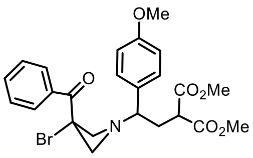

Following the general procedure 4, reaction between D-A cyclopropane (**1a**, 0.027 g, 0.1 mmol) and azabicyclo[1.1.0]butyl ketone (**2'a**, 0.016 g, 0.1 mmol) delivered compound **45**, which was purified by silica gel column chromatography (EtOAc: Hexane 2:8) to furnish the title compound **45** as a yellow liquid in 87% (0.044 g) yield. R_f 0.2 (EtOAc: Hexane 2:8)

^{1}H NMR (600 MHz, DMSO-d_6) δ 7.89 – 7.83 (m, 2H), 7.71 – 7.64 (m, 1H), 7.54 (t, J = 7.8 Hz, 2H), 7.17 (d, J = 8.5 Hz, 2H), 6.95 – 6.87 (m, 2H), 3.90 (d, J = 9.6 Hz, 1H), 3.81 (d, J = 9.5 Hz, 1H), 3.74 (s, 3H), 3.72 – 3.68 (m, 1H), 3.60 (s, 3H), 3.56 – 3.53 (m, 4H), 3.39 – 3.35 (m, 1H), 3.05 (dd, J = 8.2, 6.4 Hz, 1H), 2.16 – 2.08 (m, 1H), 2.02 – 1.93 (m, 1H). **^{13}C {^{1}H} NMR** (151 MHz, DMSO-d_6) δ 192.4, 169.1, 169.0, 158.8, 134.1, 131.4, 130.3, 129.5, 129.2, 129.0, 113.8, 68.1, 64.0, 63.6, 55.0, 52.5, 52.4, 52.1, 47.5, 32.6.

HRMS (ESI-TOF) m/z: $[M+H]^+$ $C_{24}H_{27}BrNO_6$ Calcd.504.1016, Found 504.1036.

Dimethyl 2-(2-(3-benzoyl-3-iodoazetidin-1-yl)-2-(4-methoxyphenyl)ethyl)malonate (46)

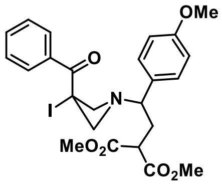

Following the general procedure 4, reaction between D-A cyclopropane (**1a**, 0.027 g, 0.1 mmol) and azabicyclo[1.1.0]butyl ketone (**2'a**, 0.016 g, 0.1 mmol) delivered compound **46**, which was purified by silica gel column chromatography (EtOAc: Hexane 2:8) to furnish the title compound **46** as a gummy liquid in 80% (0.044 g) yield. R_f 0.2 (EtOAc: Hexane 2:8)

^{1}H NMR (600 MHz, DMSO-d_6) δ 7.88 – 7.83 (m, 2H), 7.64 (t, J = 7.3 Hz, 1H), 7.52 (t, J = 7.7 Hz, 2H), 7.17 (d, J = 8.1 Hz, 2H), 6.90 (d, J = 8.3 Hz, 2H), 3.92 – 3.84 (m, 1H), 3.74 (s, 3H), 3.72 – 3.69 (m, 2H), 3.60 (s, 3H), 3.58 (s, 3H), 3.57 – 3.55 (m, 1H), 3.50 – 3.46 (m, 1H), 3.05 (dd, J = 8.2, 6.3 Hz, 1H), 2.13 – 2.09 (m, 1H), 1.99 – 1.95 (m, 1H). **^{13}C {^{1}H} NMR** (151 MHz, DMSO-d_6) δ 194.6, 169.1, 158.8, 133.9, 131.4, 130.4, 129.6, 129.2, 129.0, 113.9, 68.1, 66.3, 65.9, 55.0, 52.5, 47.5, 32.6, 27.7.

HRMS (ESI-TOF) m/z: $[M+H]^+$ $C_{24}H_{27}INO_6$ Calcd.552.0878, Found 552.0868.

Dimethyl 2-(2-(3-(2-naphthoyl)-3-bromoazetidin-1-yl)-2-(4-methoxyphenyl)ethyl) malonate (47)

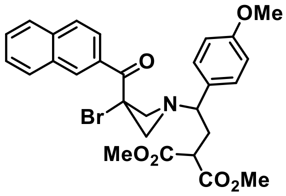

Following the general procedure 4, reaction between D-A cyclopropane (**1a**, 0.027 g, 0.1 mmol) and azabicyclo[1.1.0]butyl ketone (**2'b**, 0.021 g, 0.1 mmol) delivered compound **47**, which was purified by silica gel column chromatography (EtOAc: Hexane 2:8) to furnish the title compound **47** as a colourless liquid in 85% (0.047 g) yield. R_f 0.2 (EtOAc: Hexane 2:8)

^{1}H NMR (600 MHz, DMSO-d_6) δ 8.47 (d, J = 1.8 Hz, 1H), 8.20 (d, J = 8.2 Hz, 1H), 8.04 (d, J = 8.7 Hz, 1H), 8.00 (d, J = 8.2 Hz, 1H), 7.90 (dd, J = 8.6, 1.9 Hz, 1H), 7.69 (t, J = 7.5 Hz, 1H), 7.62 (t, J = 7.6 Hz, 1H), 7.19 (d, J = 8.2 Hz, 2H), 6.91 (d, J = 8.2 Hz, 2H), 4.08 – 3.96 (m, 1H), 3.90 – 3.84 (m, 1H), 3.79 – 3.73 (m, 5H), 3.67 – 3.66 (m, 1H), 3.61 (s, 3H), 3.56 (s, 3H), 3.09 – 3.04 (m, 1H), 2.22 – 2.11 (m, 1H), 2.00 – 1.98 (m, 1H). **^{13}C {^{1}H} NMR** (151 MHz, DMSO-d_6) δ 192.5,169.0, 158.8, 135.3, 132.0, 131.7, 130.4(2), 130.0, 129.4, 129.3, 128.8, 127.7, 127.2, 124.5, 113.9, 68.2, 64.2, 63.7, 55.0, 52.5(2), 52.48, 47.6, 32.7.

HRMS (ESI-TOF) m/z: $[M+H]^+$ $C_{28}H_{29}BrNO_6$ Calcd.554.1173, Found 554.1162.

Dimethyl 2-(2-(3-bromo-3-(4-(trifluoromethyl)benzoyl)azetidin-1-yl)-2-(4-methoxyphenyl)ethyl) malonate (48)

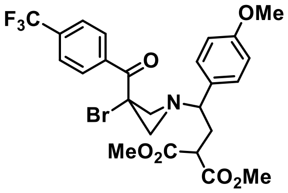

Following the general procedure 4, reaction between D-A cyclopropane (**1a**, 0.027 g, 0.1 mmol) and azabicyclo[1.1.0]butyl ketone (**2'c**, 0.023 g, 0.1 mmol) delivered compound **48**, which was purified by silica gel column chromatography (EtOAc: Hexane 2:8) to furnish the title compound **48** as a yellow liquid in 84% (0.048 g) yield. R_f 0.2 (EtOAc: Hexane 2:8)

^{1}H NMR (600 MHz, DMSO-d_6) δ 8.05 (d, J = 8.1 Hz, 2H), 7.90 (d, J = 8.3 Hz, 2H), 7.17 (d, J = 8.6 Hz, 2H), 6.90 (d, J = 8.7 Hz, 2H), 3.94 – 3.89 (m, 1H), 3.87 – 3.85 (m, 1H), 3.78 – 3.75 (m, 1H), 3.74 (s, 3H), 3.60 (s, 3H), 3.57 (s, 3H), 3.56 – 3.53 (m, 1H), 3.39 – 3.37 (m, 1H), 3.06 (dd, J = 8.2, 6.4 Hz, 1H), 2.15 – 2.10 (m, 1H), 2.03 – 1.93 (m, 1H). **^{13}C {^{1}H} NMR** (151 MHz, DMSO-d_6) δ 191.5, 169.1(2), 158.8, 134.8, 133.3 (q, J=126 Hz), 130.4, 130.3, 129.3 (d, J=72 Hz), 126.0, 124.5, 122.7, 113.9 (d, J=42 Hz), 68.1, 63.8, 63.4, 55.0(2), 52.5, 52.1, 47.4, 32.6.

HRMS (ESI-TOF) m/z: $[M+H]^+$ $C_{25}H_{26}BrF_3NO_6$ Calcd.572.0878, Found 572.0879.

Dimethyl2-(2-(3-bromo-3-(cyclohexanecarbonyl)azetidin-1-yl)-2-(4-methoxyphenyl)ethyl) malonate (49)

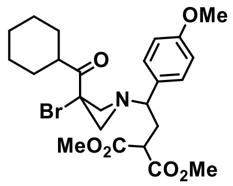

Following the general procedure 4, reaction between D-A cyclopropane (**1a**, 0.027 g, 0.1 mmol) and azabicyclo[1.1.0]butyl ketone (**2'd**, 0.017 g, 0.1 mmol) delivered compound **49**, which was purified by silica gel column chromatography (EtOAc: Hexane 2:8) to furnish the title compound **49** as a colourless liquid in 81% (0.041 g) yield. R_f 0.2 (EtOAc: Hexane 2:8)

^{1}H NMR (600 MHz, DMSO-*d*$_6$) δ 7.14 (d, *J* = 8.7 Hz, 2H), 6.90 (d, *J* = 8.7 Hz, 2H), 3.73 (s, 3H), 3.70 – 3.66 (m, 1H), 3.65 – 3.62 (m, 1H), 3.61 (s, 3H), 3.55 (s, 3H), 3.47 (d, *J* = 9.8 Hz, 1H), 3.35 – 3.32 (m, 1H), 3.30 (d, *J* = 9.3 Hz, 1H), 3.08 – 3.03 (m, 1H), 2.82 – 2.77 (m, 1H), 2.12 – 2.07 (m, 1H), 1.98 – 1.91 (m, 1H), 1.81 – 1.74 (m, 2H), 1.72 – 1.66 (m, 2H), 1.62 – 1.59 (m, 1H), 1.35 – 1.28 (m, 2H), 1.28 – 1.21 (m, 2H), 1.20 – 1.11 (m, 1H). **^{13}C {^{1}H} NMR** (151 MHz, DMSO-*d*$_6$) δ 205.3, 169.1, 169.0, 158.8, 130.1, 129.2, 129.1, 113.9, 113.8, 68.4, 62.8, 55.0(2), 53.7, 52.4(2), 47.5, 47.4, 45.3(2), 32.6, 30.0, 25.2, 25.0.

HRMS (ESI-TOF) m/z: $[M+H]^+$ $C_{24}H_{33}BrNO_6$ Calcd.510.1486, Found 510.1489.

11. Strain-Release-Driven Tandem N/C3 Functionalization of ABB-carbinol with *Bicyclo[1.1.0] butane(BCBs)* to functionalized Azetidines (52-64)

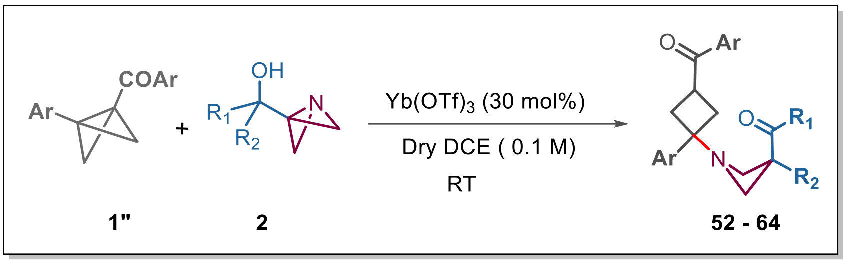

General procedure (5)

To a mixture of Donor–Acceptor Cyclopropanes (1'', 1.0 equiv.), azabicyclo[1.1.0]butyl carbinols (2; 1.0 equiv.) and $Yb(OTf)_3$ (30 mol%) was added in dry DCE (0.1 M) in inert atmosphere at RT stirred until complete disappearance of the starting material was observed (*ca*. 12 h; TLC monitored). After cooling to room temperature, the mixture was diluted with H_2O and extracted in DCM. Combined organic layers were finally washed with brine, dried over anhydrous Na_2SO_4, filtered, and concentrated under reduced pressure. The crude residue was purified by silica-gel flash column chromatography (using EtOAc /hexanes as eluent) to provide desired products **(52-64).**

12. Characterization of compounds (52-66) (from Scheme 4: main text)

(1-(3-benzoyl-1-phenylcyclobutyl)-3-(tert-butyl)azetidin-3-yl)(4-methoxyphenyl) methanone (52)

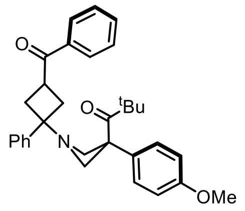

Following the general procedure 5, reaction between Bicyclo[1.1.0]butane (**1a”**, 0.023 g, 0.1 mmol) and azabicyclo[1.1.0]butyl carbinols (**2n**, 0.025 g, 0.1 mmol) delivered compound **52**, which was purified by silica gel column chromatography (EtOAc: Hexane 2:8) to furnish the title compound **52** as a gummy liquid in 88% (0.042 g) yield with dr ratio (85 : 15). R_f 0.2 (EtOAc: Hexane 2:8)

^{1}H NMR (600 MHz, Chloroform-*d*) δ 7.95 – 7.89 (m, 2H), 7.57 – 7.54 (m, 1H), 7.48 – 7.45 (m, 2H), 7.37 – 7.34 (m, 2H), 7.28 – 7.23 (m, 3H), 7.18 – 7.12 (m, 2H), 6.93 – 6.89 (m, 2H), 4.19 – 4.11 (m, 1H), 3.83 (s, 3H), 3.72 (d, *J* = 6.9 Hz, 2H), 3.54 (d, *J* = 7.0 Hz, 2H), 2.66 – 2.56 (m, 4H), 0.92 (s, 9H). **^{13}C {^{1}H} NMR** (151 MHz, Chloroform-*d*) δ 214.8, 201.0, 158.6, 141.3, 135.7, 133.1, 132.0, 128.7, 128.5, 128.0, 127.7, 126.9, 126.7, 114.2, 61.2, 55.4, 55.2, 52.6, 44.5, 37.0, 32.7, 28.4.

HRMS (ESI-TOF) m/z: $[M+Na]^+$ $C_{32}H_{35}NaNO_3$ Calcd.504.2509, Found 504.2528.

(1-(3-benzoyl-1-phenylcyclobutyl)-3-methylazetidin-3-yl)(4-methoxyphenyl) methanone (53)

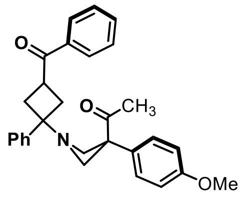

Following the general procedure 5, reaction between Bicyclo[1.1.0]butane (**1a”**, 0.023 g, 0.1 mmol) and azabicyclo[1.1.0]butyl carbinols (**2d**, 0.021 g, 0.1 mmol) delivered compound **53**, which was purified by silica gel column chromatography (EtOAc: Hexane 2:8) to furnish the title compound **53** as a yellow liquid in 80% (0.035 g) yield with dr ratio (78 : 22). R_f 0.2 (EtOAc: Hexane 2:8)

^{1}H NMR (600 MHz, Chloroform-*d*) δ 7.89 (d, *J* = 7.9 Hz, 2H), 7.54 (t, *J* = 7.5 Hz, 1H), 7.45 (t, *J* = 7.5 Hz, 2H), 7.30 (t, *J* = 7.5 Hz, 2H), 7.20 (t, *J* = 7.4 Hz, 1H), 7.11 (d, *J* = 7.6 Hz, 2H), 7.04 (d, *J* = 8.5 Hz, 2H), 6.84 (d, *J* = 8.5 Hz, 2H), 4.14 – 4.08 (m, 1H), 3.83 (d, *J* = 6.5 Hz, 2H), 3.78 (d, *J* = 4.0 Hz, 3H), 3.41 (d, *J* = 6.7 Hz, 2H), 2.64 – 2.51 (m, 4H), 2.00 (s, 3H). **^{13}C {^{1}H} NMR** (151 MHz, Chloroform-*d*) δ 207.5, 200.9, 158.8, 141.5, 135.6, 133.1, 131.9, 128.7, 128.5, 128.0, 127.6, 127.0, 126.7, 114.3, 61.3, 55.4, 54.7, 53.4, 36.9, 32.7, 25.0.

HRMS (ESI-TOF) m/z: $[M+H]^+$ $C_{29}H_{30}NO_3$ Calcd.440.2220, Found 440.2237.

Tert-butyl 3-(1-(3-benzoyl-1-phenylcyclobutyl)-3-methylazetidine-3-carbonyl)-1H-indole-1-carboxylate (54)

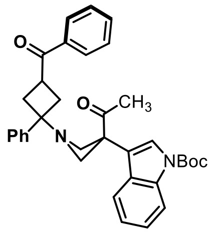

Following the general procedure 5, reaction between Bicyclo[1.1.0]butane (**1a"**, 0.023 g, 0.1 mmol) and azabicyclo[1.1.0]butyl carbinols (**2h**, 0.021 g, 0.1 mmol) delivered compound **54**, which was purified by silica gel column chromatography (EtOAc: Hexane 2:8) to furnish the title compound **54** as a reddish liquid in 75% (0.041 g) yield with dr ratio (85 : 15). R_f 0.2 (EtOAc: Hexane 2:8)

^{1}H NMR (600 MHz, Chloroform-*d*) δ 8.14 – 7.99 (m, 1H), 7.93 – 7.87 (m, 2H), 7.56 – 7.52 (m, 1H), 7.45 (t, *J* = 7.7 Hz, 3H), 7.33 – 7.28 (m, 3H), 7.25 – 7.20 (m, 2H), 7.18 – 7.15 (m, 1H), 7.14 – 7.11 (m, 2H), 4.15 – 4.09 (m, 1H), 3.93 (d, *J* = 6.7 Hz, 2H), 3.51 – 3.49 (m, 2H), 2.70 – 2.57 (m, 4H), 2.01 (s, 3H), 1.69 (s, 9H). **^{13}C {^{1}H} NMR** (151 MHz, Chloroform-*d*) δ 206.8, 200.9, 141.6, 135.8, 135.6, 133.2, 128.7(2), 128.6, 128.5(2), 128.1, 127.0, 126.7, 124.9, 123.3, 123.0, 120.0, 115.5, 84.3, 61.4, 53.9, 48.2, 36.8, 32.7, 28.4, 25.0.

HRMS (ESI-TOF) m/z: $[M+H]^+$ $C_{35}H_{37}N_2O_4$ Calcd.549.2748, Found 549.2767.

(1-(3-benzoyl-1-(o-tolyl)cyclobutyl)-3-phenylazetidin-3-yl)(phenyl)methanone (55)

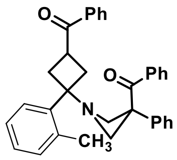

Following the general procedure 5, reaction between Bicyclo[1.1.0]butane (**1b"**, 0.025 g, 0.1 mmol) and azabicyclo[1.1.0]butyl carbinols (**2a**, 0.024 g, 0.1 mmol) delivered compound **55**, which was purified by silica gel column chromatography (EtOAc: Hexane 2:8) to furnish the title compound **55** as a yellow liquid in 75% (0.036 g) yield with dr ratio (80 : 20). R_f 0.2 (EtOAc: Hexane 2:8)

^{1}H NMR (600 MHz, DMSO-*d*$_6$) δ 7.93 – 7.91 (m, 2H), 7.68 – 7.64 (m, 2H), 7.63 – 7.60 (m, 1H), 7.53 – 7.49 (m, 3H), 7.44 – 7.38 (m, 4H), 7.36 – 7.33 (m, 2H), 7.25 – 7.19 (m, 2H), 7.11 – 7.08 (m, 2H), 7.06 – 7.02 (m, 1H), 4.16 – 4.08 (m, 1H), 3.85 (d, *J* = 6.7 Hz, 2H), 3.74 – 3.59 (m, 2H), 2.84 – 2.74 (m, 2H), 2.64 – 2.59 (m, 2H), 2.26 (s, 3H). **^{13}C {^{1}H} NMR** (151 MHz, DMSO-*d*$_6$) δ 200.3, 198.5, 141.1, 140.2, 136.4, 135.0, 133.4, 133.2, 131.2, 129.2, 129.0, 128.8(2), 128.3, 127.9, 127.0, 126.9, 126.0, 125.1, 61.7, 56.0, 51.3, 36.0, 19.8.

HRMS (ESI-TOF) m/z: $[M+H]^+$ $C_{34}H_{32}NO_2$ Calcd.486.2428, Found 486.2432.

(1-(3-benzoyl-1-phenylcyclobutyl)-3-phenylazetidin-3-yl)(phenyl)methanone (56)

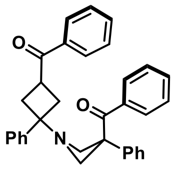

Following the general procedure 5, reaction between Bicyclo[1.1.0]butane (**1a”**, 0.023 g, 0.1 mmol) and azabicyclo[1.1.0]butyl carbinols (**2a**, 0.024 g, 0.1 mmol) delivered compound **56**, which was purified by silica gel column chromatography (EtOAc: Hexane 2:8) to furnish the title compound **56** as a colourless liquid in 82% (0.039 g) yield with dr ratio (90 : 10). R_f 0.2 (EtOAc: Hexane 2:8)

^{1}H NMR (600 MHz, Chloroform-*d*) δ 7.88 – 7.86 (m, 2H), 7.64 – 7.63 (m, 2H), 7.58 – 7.55 (m, 2H), 7.54 – 7.52 (m, 1H), 7.45 – 7.38 (m, 7H), 7.33 – 7.27 (m, 5H), 7.23 – 7.21 (m, 1H), 3.86 (d, *J* = 7.4 Hz, 2H), 3.67 – 3.59 (m, 1H), 3.57 (d, *J* = 7.4 Hz, 2H), 2.83 – 2.79 (m, 2H), 2.58 – 2.50 (m, 2H). **^{13}C {^{1}H} NMR** (151 MHz, Chloroform-*d*) δ 200.4, 199.4, 143.3, 141.6, 135.8, 134.1, 133.2, 132.9, 129.8, 129.2, 129.1, 128.8, 128.5(2), 127.1, 126.2, 62.0, 56.5, 51.1, 34.0, 31.5.

HRMS (ESI-TOF) m/z: $[M+Na]^+$ $C_{33}H_{29}NNaO_2$ Calcd.494.2091, Found 494.2105.

(1-(3-benzoyl-1-phenylcyclobutyl)-3-(2-chlorophenyl)azetidin-3-yl)(phenyl)methanone (57)

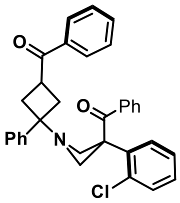

Following the general procedure 5, reaction between Bicyclo[1.1.0]butane (**1a”**, 0.023 g, 0.1 mmol) and azabicyclo[1.1.0]butyl carbinols (**2k**, 0.027 g, 0.1 mmol) delivered compound **57**, which was purified by silica gel column chromatography (EtOAc: Hexane 2:8) to furnish the title compound **57** as a yellow liquid in 76% (0.038 g) yield with dr ratio (80 : 20). R_f 0.2 (EtOAc: Hexane 2:8)

^{1}H NMR (600 MHz, Chloroform-*d*) δ 7.89 – 7.86 (m, 2H), 7.54 – 7.51 (m, 1H), 7.45 – 7.42 (m, 2H), 7.35 (dd, *J* = 8.0, 1.1 Hz, 1H), 7.31 – 7.27 (m, 4H), 7.25 – 7.22 (m, 4H), 7.20 – 7.16 (m, 1H), 7.08 – 7.03 (m, 3H), 6.72 (dd, *J* = 7.8, 1.6 Hz, 1H), 4.17 – 4.08 (m, 1H), 3.90 (d, *J* = 7.0 Hz, 2H), 3.46 (d, *J* = 6.6 Hz, 2H), 2.56 (d, *J* = 8.9 Hz, 4H). **^{13}C {^{1}H} NMR** (151 MHz, Chloroform-*d*) δ 203.2, 201.0, 141.03,139.6, 137.9, 135.7, 133.1, 131.2, 130.9, 130.5, 128.9, 128.7, 128.5, 128.2, 128.0(2), 127.4, 127.0, 127.1(2), 127.0, 126.7, 126.1, 61.2, 55.9, 54.1, 37.2, 32.8.

HRMS (ESI-TOF) m/z: $[M+H]^+$ $C_{33}H_{29}NClO_2$ Calcd.506.1881, Found 506.1896.

(3-(3-benzoyl-3-phenylazetidin-1-yl)-3-phenylcyclobutyl)(thiophen-2-yl)methanone (58)

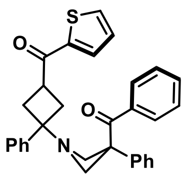

Following the general procedure 5, reaction between Bicyclo[1.1.0]butane (**1c"**, 0.024 g, 0.1 mmol) and azabicyclo[1.1.0]butyl carbinols (**2a**, 0.024 g, 0.1 mmol) delivered compound **58**, which was purified by silica gel column chromatography (EtOAc: Hexane 2:8) to furnish the title compound **58** as a pale yellow liquid in 78% (0.037 g) yield with dr ratio (90 :10). R_f 0.2 (EtOAc: Hexane 2:8)

^{1}H NMR (600 MHz, DMSO-d_6) δ 7.98 (d, *J* = 5.0 Hz, 1H), 7.92 (d, *J* = 3.7 Hz, 1H), 7.63 (d, *J* = 7.9 Hz, 2H), 7.52 (t, *J* = 7.4 Hz, 1H), 7.40 (t, *J* = 7.7 Hz, 3H), 7.36 – 7.32 (m, 5H), 7.25 – 7.21 (m, 3H), 7.16 (d, *J* = 7.5 Hz, 2H), 4.14 – 4.02 (m, 1H), 3.80 (d, *J* = 7.0 Hz, 2H), 3.70 (d, *J* = 6.9 Hz, 2H), 2.72 – 2.62 (m, 2H), 2.48 – 2.45(m, 2H). **^{13}C {^{1}H} NMR** (151 MHz, DMSO-d_6) δ 198.5, 193.8, 142.1, 141.0, 134.7, 133.4, 133.2, 129.2, 129.0, 128.7, 127.9, 127.0, 126.7, 126.5, 126.0, 60.8, 55.6, 51.0, 35.9, 32.0.

HRMS (ESI-TOF) m/z: $[M+H]^+$ $C_{31}H_{28}NO_2S$ Calcd.478.1835, Found 478.1839.

1-(3-benzoyl-1-phenylcyclobutyl)-11'H-spiro[azetidine-3,10'-dibenzo[b,f]oxepin]-11'-one (59)

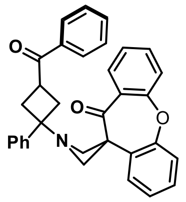

Following the general procedure 5, reaction between Bicyclo[1.1.0]butane (**1a"**, 0.023 g, 0.1 mmol) and azabicyclo[1.1.0]butyl carbinols (**2x**, 0.024 g, 0.1 mmol) delivered compound **59**, which was purified by silica gel column chromatography (EtOAc: Hexane 2:8) to furnish the title compound **59** as a yellow liquid in 74% (0.036 g) yield with dr ratio (78 :12). R_f 0.2 (EtOAc: Hexane 2:8)

^{1}H NMR (600 MHz, Chloroform-*d*) δ 8.14 – 8.13 (m, 1H), 7.90 – 7.87 (m, 2H), 7.57 – 7.54 (m, 1H), 7.54 – 7.51 (m, 1H), 7.45 – 7.42 (m, 2H), 7.34 – 7.32 (m, 1H), 7.28 – 7.27 (m, 2H), 7.25 – 7.22 (m, 1H), 7.20 – 7.14 (m, 4H), 7.11 – 7.07 (m, 3H), 4.14 – 4.08 (m, 1H), 4.07 – 4.01 (m, 2H), 3.54 (d, *J* = 7.1 Hz, 2H), 2.63 – 2.57 (m, 4H). **^{13}C {^{1}H} NMR** (151 MHz, Chloroform-*d*) δ 201.0, 191.2, 159.2, 156.5, 141.4, 135.6, 134.9, 133.1, 131.6, 131.1, 128.7, 128.5, 128.0, 127.1, 126.9, 126.8, 126.7, 126.4, 124.1, 123.9, 121.1, 121.0, 61.3, 53.6, 52.1, 36.9, 32.8.

HRMS (ESI-TOF) m/z: $[M+Na]^+$ $C_{33}H_{27}NNaO_3$ Calcd.508.1883, Found 508.1904.

(E)-(1-(3-benzoyl-1-phenylcyclobutyl)-3-(4-methoxystyryl)azetidin-3-yl)(4-bromophenyl) methanone (60)

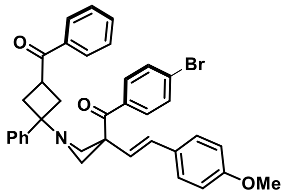

Following the general procedure 5, reaction between Bicyclo[1.1.0]butane (**1a”**, 0.023 g, 0.1 mmol) and azabicyclo[1.1.0]butyl carbinols (**2o**, 0.037 g, 0.1 mmol) delivered compound **60**, which was purified by silica gel column chromatography (EtOAc: Hexane 2:8) to furnish the title compound **60** as a gummy liquid in 81% (0.049 g) yield with dr ratio (90 :10). R_f 0.2 (EtOAc: Hexane 2:8)

^{1}H NMR (600 MHz, Chloroform-*d*) δ 7.94 – 7.85 (m, 2H), 7.65 – 7.60 (m, 2H), 7.56 – 7.50 (m, 3H), 7.45 (t, *J* = 7.7 Hz, 2H), 7.34 (t, *J* = 7.6 Hz, 2H), 7.26 – 7.24 (m, 1H), 7.21 – 7.16 (m, 2H), 7.16 – 7.08 (m, 2H), 6.81 (d, *J* = 8.8 Hz, 2H), 6.43 (d, *J* = 16.3 Hz, 1H), 6.17 (d, *J* = 16.3 Hz, 1H), 4.18 – 4.12(m, 1H), 3.80 (d, *J* = 6.9 Hz, 2H), 3.78 (s, 3H), 3.51 – 3.46 (m, 2H), 2.66 – 2.55 (m, 4H). **^{13}C {^{1}H} NMR** (151 MHz, Chloroform-*d*) δ 200.9, 199.0, 159.6, 141.3, 135.6, 133.1, 132.7, 132.0, 131.2, 131.1, 129.2, 128.7(2), 128.5, 128.3, 128.0, 127.6, 127.0, 126.8, 114.1, 61.3, 56.5, 55.4, 49.1, 36.9, 32.8.

HRMS (ESI-TOF) m/z: $[M+H]^+$ $C_{36}H_{33}BrNO_3$ Calcd.606.1638, Found 606.1639

(3-(17-oxa-2-azadispiro [3.0.11^5.1^4] heptadecan-2-yl)-3-phenylcyclobutyl) (4-methoxyphenyl) methanone (63)

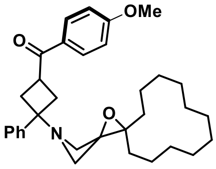

Following the general procedure 5, reaction between Bicyclo[1.1.0]butane (**1d”**, 0.026 g, 0.1 mmol) and azabicyclo[1.1.0]butyl carbinols (**2r**, 0.024 g, 0.1 mmol) delivered compound **63**, which was purified by silica gel column chromatography (EtOAc: Hexane 2:8) to furnish the title compound **63** as a yellow liquid in 81% (0.041 g) yield with dr ratio (75 :25). R_f 0.2 (EtOAc: Hexane 2:8)

^{1}H NMR (600 MHz, Chloroform-*d*) δ 7.90 – 7.87 (m, 2H), 7.32 (t, *J* = 7.6 Hz, 2H), 7.24 – 7.21 (m, 1H), 7.14 – 7.13 (m, 2H), 6.93 – 6.91 (m, 2H), 4.17 – 4.11 (m, 1H), 3.85 (s, 3H), 3.49 (d, *J* = 9.2 Hz, 2H), 3.41 – 3.38 (m, 2H), 2.71 – 2.64 (m, 2H), 2.62 – 2.58 (m, 2H), 1.43 – 1.38 (m, 7H), 1.35 – 1.29 (m, 15H). **^{13}C {^{1}H} NMR** (151 MHz, Chloroform-*d*) δ 199.5, 163.5, 141.8, 130.7, 128.7, 128.0, 127.0, 126.8, 113.9, 65.2, 63.6, 61.6, 55.6, 53.4, 36.6, 33.4, 28.1, 26.1, 25.4, 22.9, 22.8, 20.6.

HRMS (ESI-TOF) m/z: $[M+Na]^+$ $C_{33}H_{43}NNaO_3$ Calcd.524.3135, Found 524.3151.

(3-(20-oxa-2-azadispiro [3.0.14^5.1^4] icosan-2-yl)-3-phenylcyclobutyl) (4-methoxyphenyl) methanone (64)

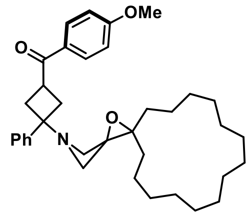

Following the general procedure 5, reaction between Bicyclo[1.1.0]butane (**1d"**, 0.026 g, 0.1 mmol) and azabicyclo[1.1.0]butyl carbinols (**2s**, 0.026 g, 0.1 mmol) delivered compound **64**, which was purified by silica gel column chromatography (EtOAc: Hexane 2:8) to furnish the title compound **64** as a colourless liquid in 76% (0.041 g) yield with dr ratio (85 :15). R_f 0.2 (EtOAc: Hexane 2:8)

^{1}H NMR (600 MHz, Chloroform-*d*) δ 7.91 – 7.89 (m, 2H), 7.33 (t, *J* = 7.6 Hz, 2H), 7.23 (t, *J* = 7.4 Hz, 1H), 7.15 – 7.11 (m, 2H), 6.94 – 6.91 (m, 2H), 4.17 – 4.11 (m, 1H), 3.86 (s, 3H), 3.49 (d, *J* = 9.0 Hz, 2H), 3.43 – 3.38 (m, 2H), 2.71 – 2.64 (m, 2H), 2.62 – 2.59 (m, 2H), 1.38 – 1.30 (m, 28H). **^{13}C {^{1}H} NMR** (151 MHz, Chloroform-*d*) δ 199.5, 163.5, 141.7, 130.8, 128.7, 128.0, 127.0, 126.8, 113.9, 65.0, 64.2, 61.6, 55.6, 53.3, 40.9, 36.6, 33.5, 31.1, 27.7, 26.9, 26.7, 26.6(2), 23.0.

HRMS (ESI-TOF) m/z: $[M+Na]^+$ $C_{36}H_{49}NNaO_3$ Calcd.566.3605, Found 566.3611.

(3-((-dispiro[adamantane-2,2'-oxirane-3',3''-azetidin]-1''-yl)-3-phenylcyclobutyl) (naphthalen-1-yl) methanone (65)

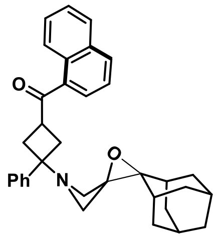

Following the general procedure 5, reaction between Bicyclo[1.1.0]butane (**1e"**, 0.028 g, 0.1 mmol) and azabicyclo[1.1.0]butyl carbinols (**2t**, 0.021 g, 0.1 mmol) delivered compound **65**, which was purified by silica gel column chromatography (EtOAc: Hexane 2:8) to furnish the title compound **65** as a yellow liquid in 78% (0.038 g) yield with dr ratio (75 :25). R_f 0.2 (EtOAc: Hexane 2:8)

^{1}H NMR (600 MHz, Chloroform-*d*) δ 8.44 (d, *J* = 1.7 Hz, 1H), 8.01 – 7.98 (m, 2H), 7.90 – 7.87 (m, 2H), 7.61 – 7.59 (m, 1H), 7.57 – 7.53 (m, 1H), 7.36 – 7.33 (m, 2H), 7.26 – 7.23 (m, 1H), 7.19 – 7.15 (m, 2H), 4.41 – 4.35 (m, 1H), 3.56 (d, *J* = 9.3 Hz, 2H), 3.52 (d, *J* = 9.3 Hz, 2H), 2.76 – 2.72 (m, 2H), 2.70 – 2.65 (m, 2H), 1.94 – 1.90 (m, 3H), 1.85 – 1.82 (m,1H), 1.80 – 1.77 (m, 2H), 1.74 – 1.68 (m, 4H), 1.58 (d, *J* = 12.5 Hz, 2H), 1.24 (d, *J* = 3.0 Hz, 2H). **^{13}C {^{1}H} NMR** (151 MHz, Chloroform-*d*) δ 201.0, 141.4, 135.7, 133.0, 132.7, 130.1(2), 129.7, 128.6, 128.5, 128.0, 127.9, 127.1, 126.9, 124.3, 68.8, 64.2, 61.6, 52.8, 36.9, 36.6, 36.2, 34.6, 33.8, 33.3, 27.3, 26.9.

HRMS (ESI-TOF) m/z: $[M+H]^+$ $C_{34}H_{36}NO_2$ Calcd.490.2741, Found 490.2761.

(3-((-dispiro[adamantane-2,2'-oxirane-3',3''-azetidin]-1''-yl)-3-phenylcyclobutyl) (phenyl) methanone (66)

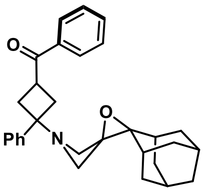

Following the general procedure 5, reaction between Bicyclo[1.1.0]butane (**1a"**, 0.023 g, 0.1 mmol) and azabicyclo[1.1.0]butyl carbinols (**2t**, 0.021 g, 0.1 mmol) delivered compound **66**, which was purified by silica gel column chromatography (EtOAc: Hexane 2:8) to furnish the title compound **66** as a yellow liquid in 86% (0.038 g) yield with dr ratio (90 :10). R_f 0.2 (EtOAc: Hexane 2:8)

^{1}H NMR (600 MHz, Chloroform-*d*) δ 7.95 – 7.90 (m, 2H), 7.55 (t, *J* = 7.4 Hz, 1H), 7.46 (t, *J* = 7.8 Hz, 2H), 7.34 (t, *J* = 7.5 Hz, 2H), 7.24 (t, *J* = 7.4 Hz, 1H), 7.17 – 7.13 (m, 2H), 4.26 – 4.20 (m,1H), 3.52 (d, *J* = 9.2 Hz, 2H), 3.48 (d, *J* = 9.2 Hz, 2H), 2.69 – 2.66 (m, 2H), 2.62 – 2.58 (m, 2H), 1.89 (d, *J* = 11.0 Hz, 3H), 1.84 – 1.81 (m, 1H), 1.80 – 1.75 (m, 2H), 1.73 – 1.66 (m, 4H), 1.58 (d, *J* = 12.5 Hz, 2H), 1.22 (s, 2H). **^{13}C {^{1}H} NMR** (151 MHz, Chloroform-*d*) δ 201.0, 141.4, 135.7, 133.2, 128.8, 128.5, 128.0, 127.0, 126.9, 68.8, 64.3, 61.5, 52.7, 36.9, 36.6, 36.2, 34.6, 33.6, 33.3, 27.3, 26.9.

HRMS (ESI-TOF) m/z: $[M+Na]^+$ $C_{30}H_{33}NNaO_2$ Calcd.462.2404, Found 462.2424.

13. Scale up and Reaction Profile:

Synthesis of compound 3 in 2.0 mmol scale

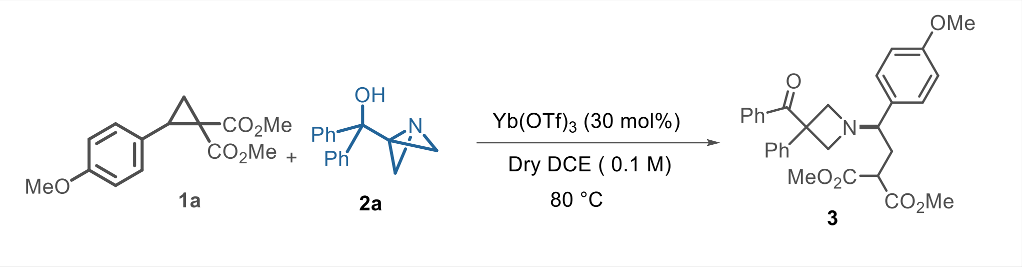

To a mixture of Donor–Acceptor Cyclopropanes (1a, 1.0 equiv.; 2.0 mmol; 0.528 g), azabicyclo[1.1.0]butyl carbinols (2a; 1.0 equiv.; 2.0 mmol; 0.474 g) and $Yb(OTf)_3$ (30 mol%; 0.372 g) was added in dry DCE (0.1 M, 20 ml) in inert atmosphere. The reaction mixture was heated to 80°C in an oil bath and stirred until complete disappearance of the starting material was observed (*ca.* 12 h; TLC monitored). After cooling to room temperature, the mixture was diluted with H_2O and extracted in DCM. Combined organic layers were finally washed with brine, dried over anhydrous Na_2SO_4, filtered, and concentrated under reduced pressure. The crude residue was purified by silica-gel flash column chromatography (using EtOAc /hexanes as eluent) to furnish the title compound 3 as a yellow liquid in 88% (0.882 g) yield. Rf 0.2 (EtOAc: Hexane 2:8).

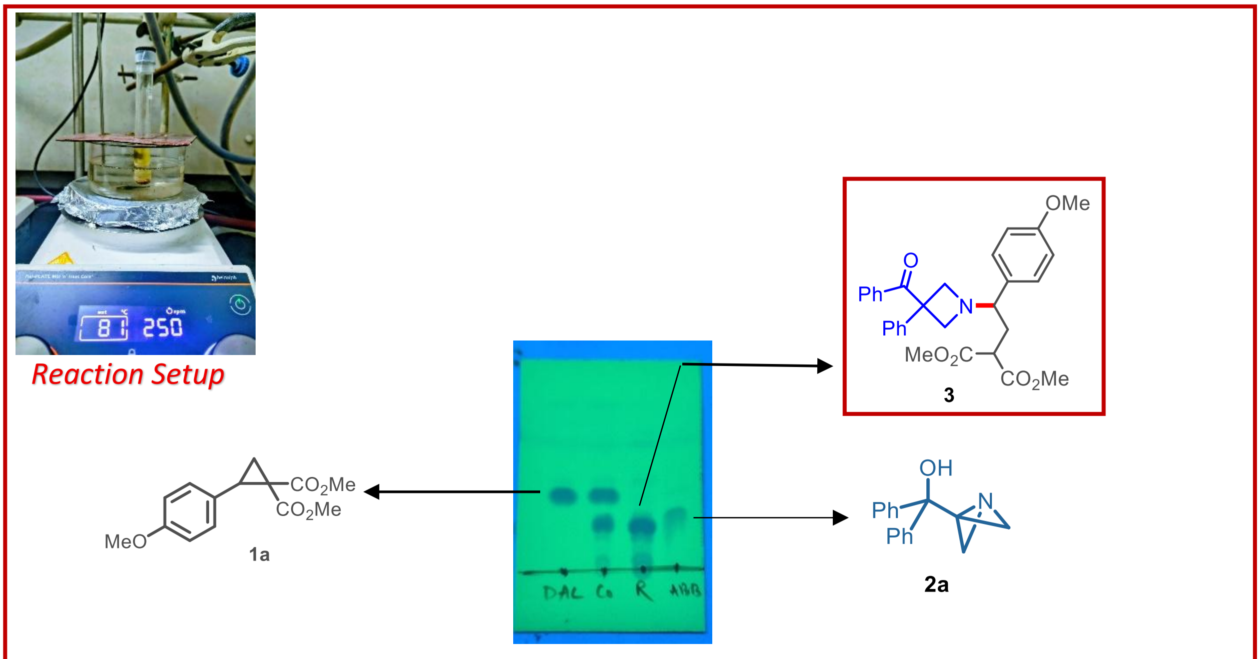

Rf 0.2 (EtOAc: Hexane 2:8).

14. Control Experiments:

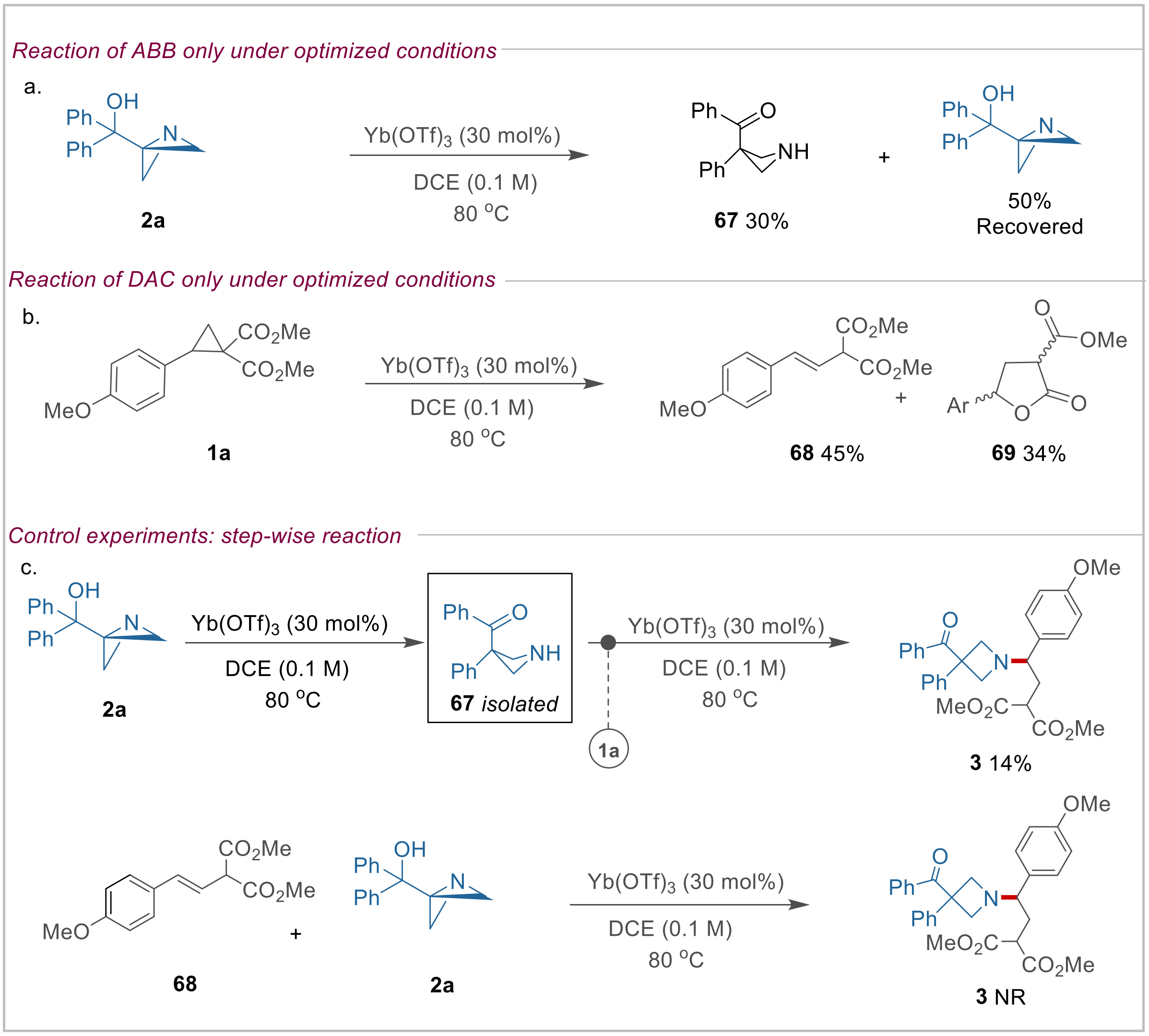

Figure S4: List of control experiments

Reaction of ABB only under optimized conditions

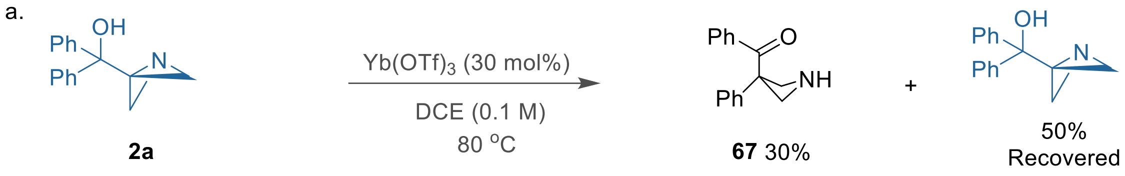

2a **67** 30%

To a mixture azabicyclo[1.1.0]butyl carbinols (2a; 1.0 equiv.; 0.2 mmol) and $Yb(OTf)_3$ (30 mol%) was added in dry DCE (0.1 M) in inert atmosphere. The reaction mixture was heated to 80°C in an oil bath and stirred until complete disappearance of the starting material was observed (*ca*. 12 h; TLC monitored). After cooling to room temperature, the mixture was diluted with H_2O and extracted in DCM. Combined organic layers were finally washed with brine, dried over anhydrous Na_2SO_4, filtered, and concentrated under reduced pressure. Compound **67** was purified by preparative TLC.

Compound 67

1H NMR (400 MHz, CDCl3) δ 7.67 (d, J = 7.3 Hz, 2H), 7.50 (d, J = 7.5 Hz, 2H), 7.45 (t, J = 7.4 Hz, 1H), 7.39-7.32 (m, 5H), 4.47 (d, J = 8.5 Hz, 2H), 4.01 (d, J = 8.5 Hz, 2H), 2.06 (brs, 1H) ;**13C{^{1}H}NMR** (100 MHz, CDCl3) δ 199.9, 141.6, 133.9, 132.9, 129.7, 129.3, 128.8, 128.6, 128.1, 127.2, 125.8, 57.2, 56.6; HRMS (ESI-TOF) m/z: [M+H]+ calculated for C16H16NO 238.1226 mass found 238.1233

Reaction of DAC only under optimized conditions

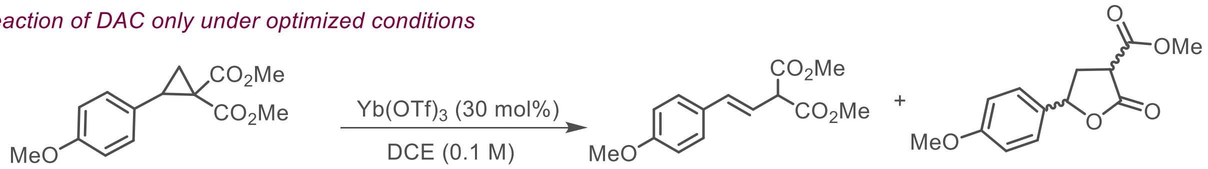

1a **68** 45% **69** 34%

To a mixture of Donor–Acceptor Cyclopropanes (1, 1.0 equiv.), $Yb(OTf)_3$ (30 mol%) was added in dry DCE (0.1 M) in inert atmosphere. The reaction mixture was heated to 80°C in an oil bath and stirred until complete disappearance of the starting material was observed (*ca*. 12 h; TLC monitored). After cooling to room temperature, the mixture was diluted with H_2O and extracted in DCM. Combined organic layers were finally washed with brine, dried over anhydrous Na_2SO_4, filtered, and concentrated under reduced pressure. The crude residue was purified by silica-gel flash column chromatography (using EtOAc /hexanes as eluent) to provide desired products **68** and **69**

NMR for that compound has been reported; we have matched with that reported data.[4]

15. 1D NOE Experiment for compound 66 and 64

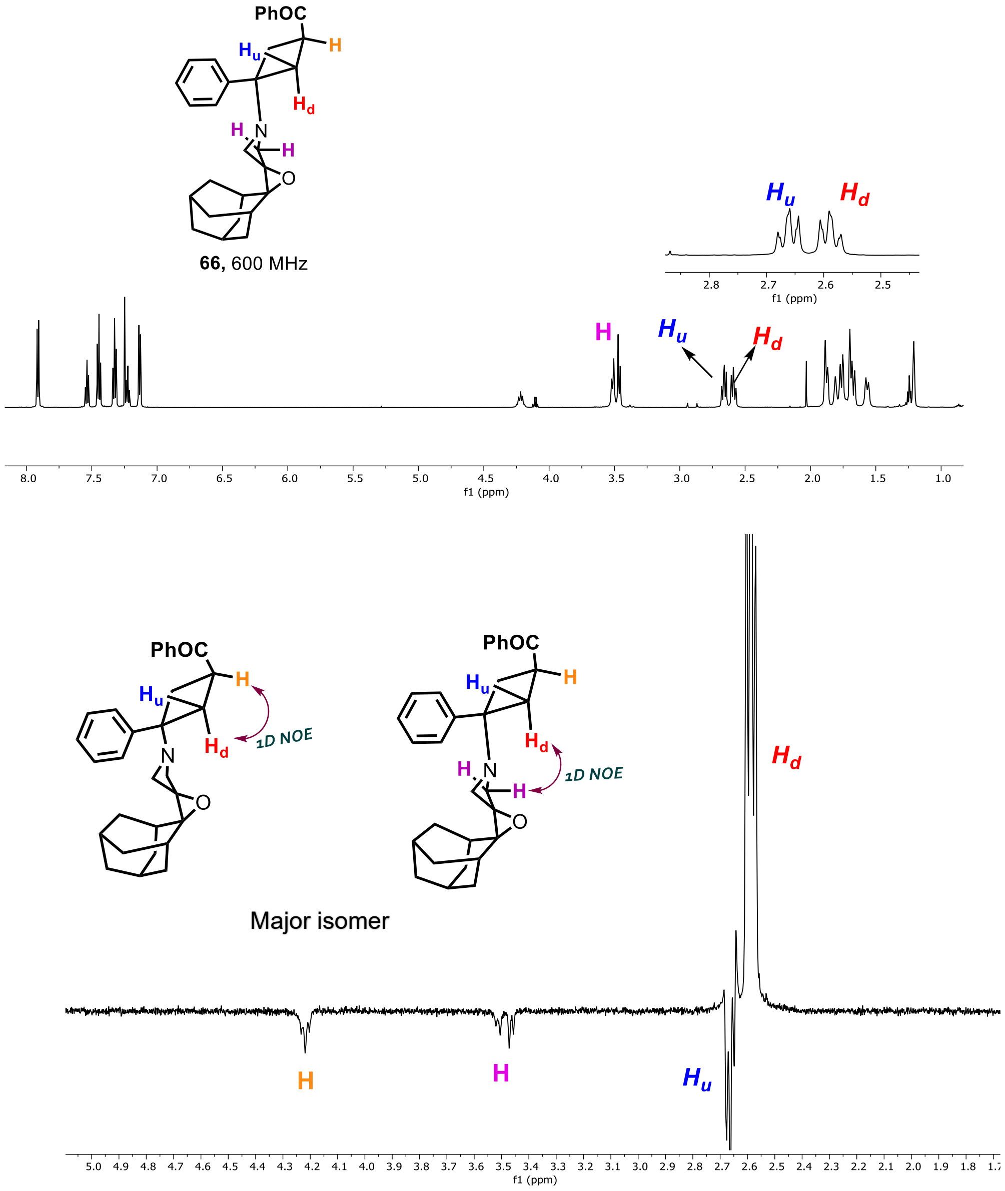

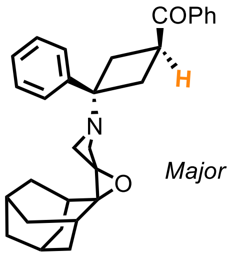

Major isomer

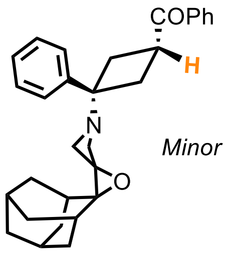

Minor isomer

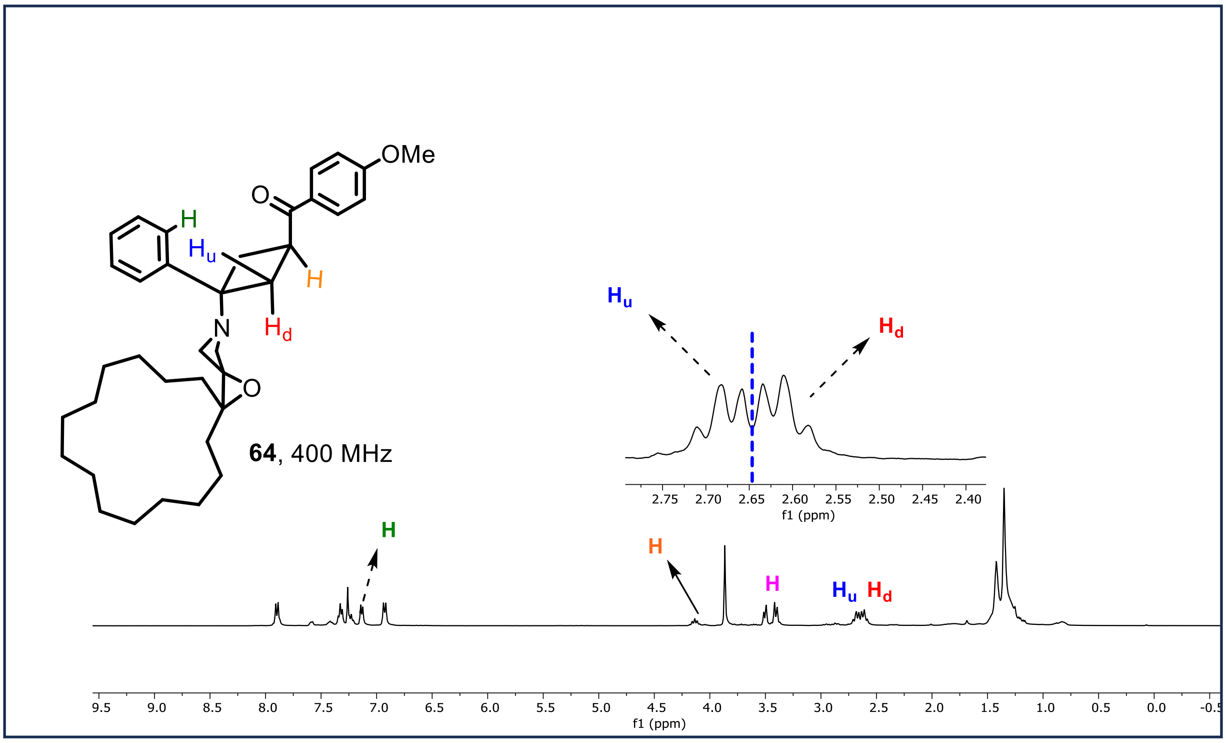
OMe
O
H
Hu
H
Hd
N
O
64, 400 MHz
Hu
Hd
2.75 2.70 2.65 2.60 2.55 2.50 2.45 2.40
f1 (ppm)
H
H
H
Hu Hd
9.5 9.0 8.5 8.0 7.5 7.0 6.5 6.0 5.5 5.0 4.5 4.0 3.5 3.0 2.5 2.0 1.5 1.0 0.5 0.0
f1 (ppm)

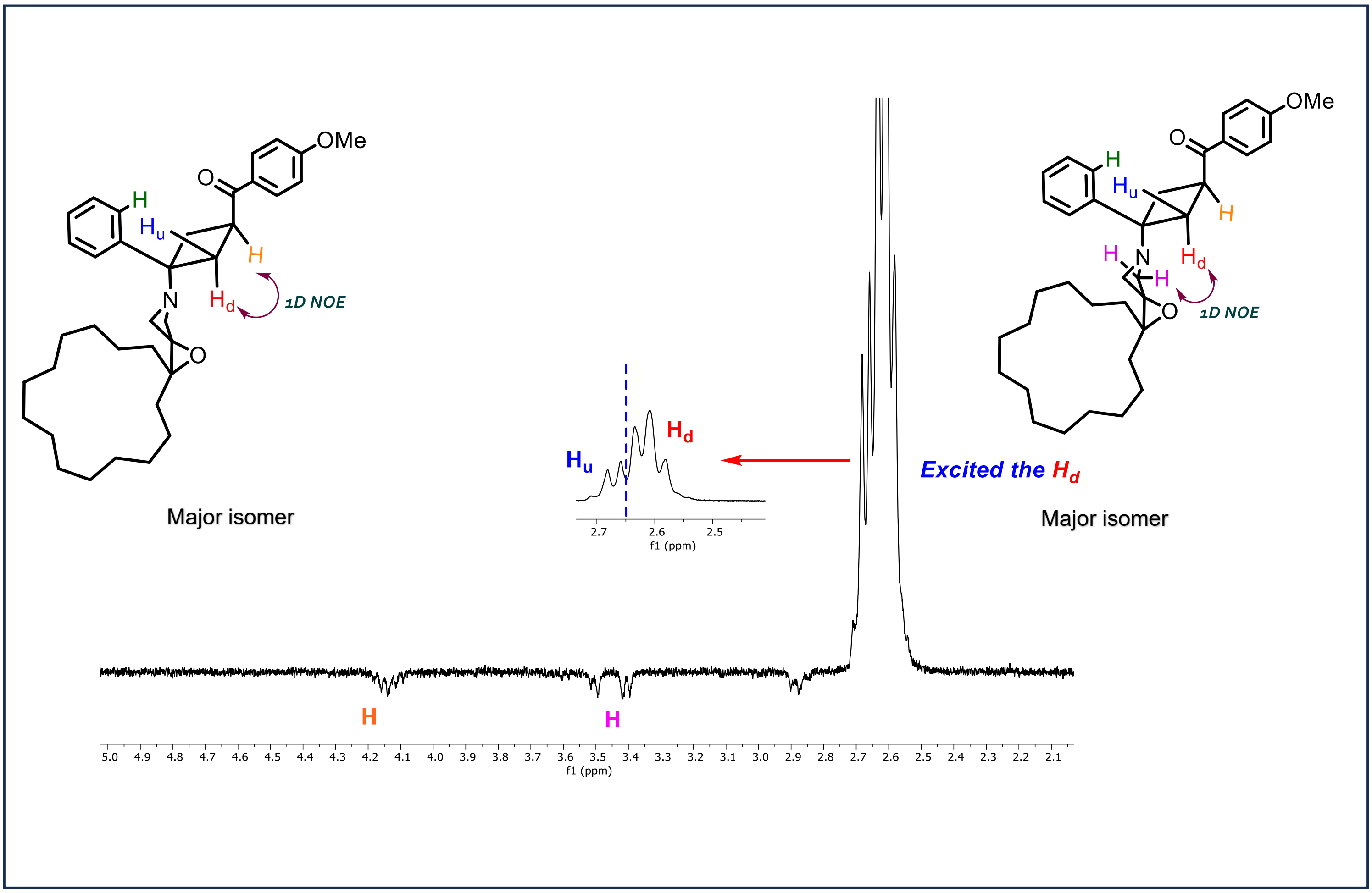
OMe
O
H
Hu
H
Hd
1D NOE
N
O
Major isomer
Hu
Hd
2.7 2.6 2.5
f1 (ppm)
Excited the Hd
Major isomer
H
H
5.0 4.9 4.8 4.7 4.6 4.5 4.4 4.3 4.2 4.1 4.0 3.9 3.8 3.7 3.6 3.5 3.4 3.3 3.2 3.1 3.0 2.9 2.8 2.7 2.6 2.5 2.4 2.3 2.2 2.1
f1 (ppm)

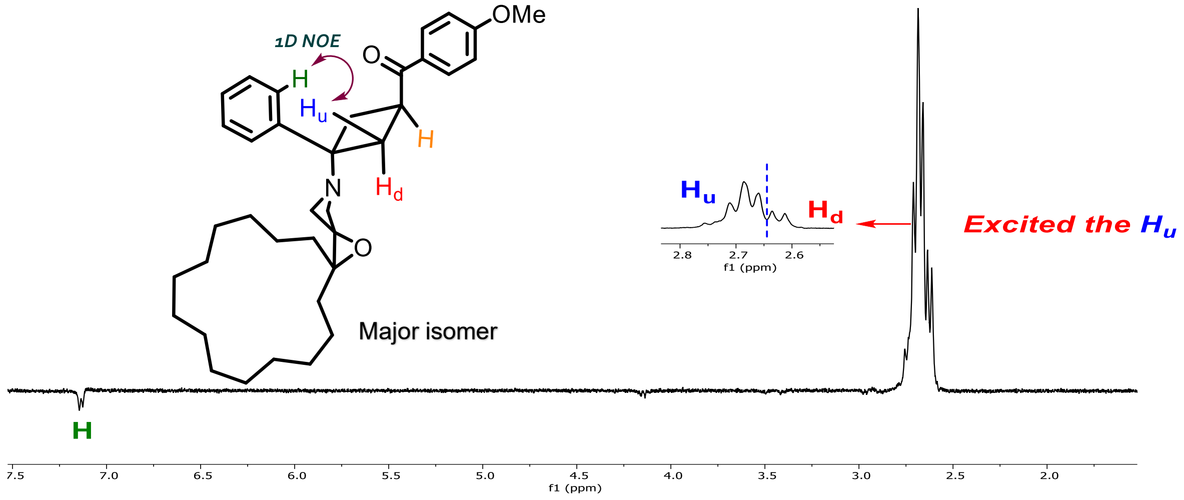

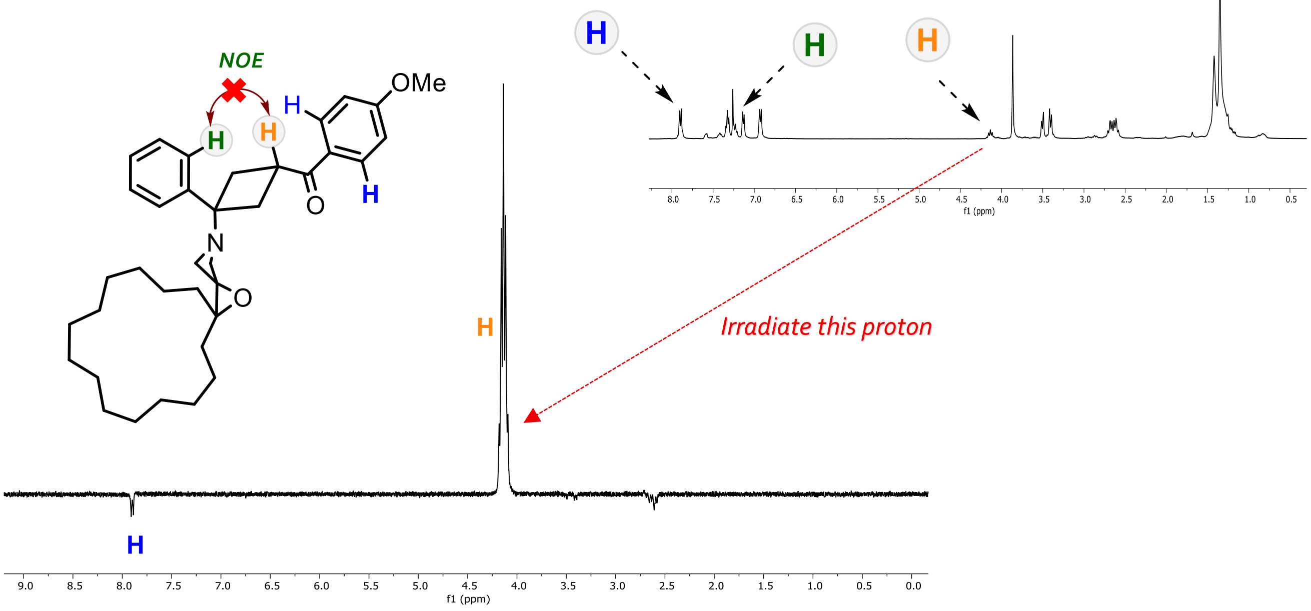

Conclusion :

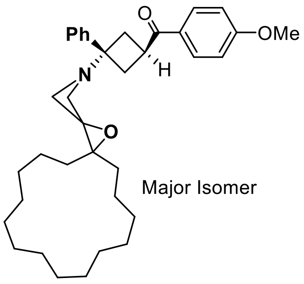

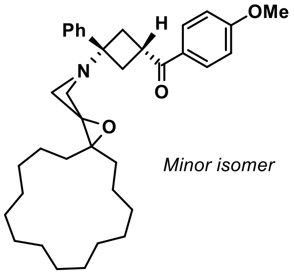

Similar 1D NOE correlations have also been reported in related systems (see **Ref. 6**), further supporting our structural assignment.

16. Plausible Mechanism:

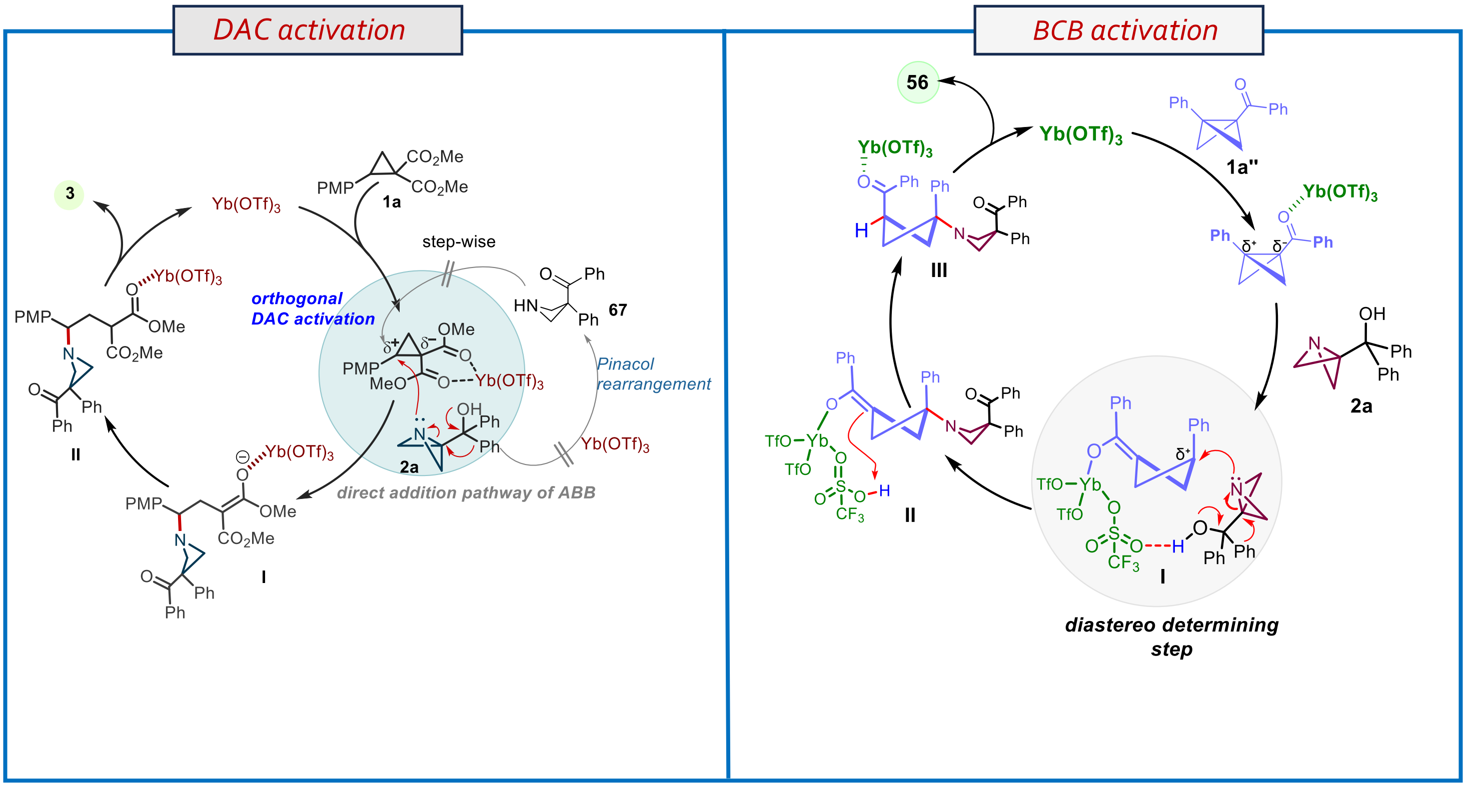

The plausible mechanism for BCB activation and diastereoselectivity is also supported by the **Reff. 5.**

^{1}H spectra at 600 MHz in $CDCl_3$ (**2a**)

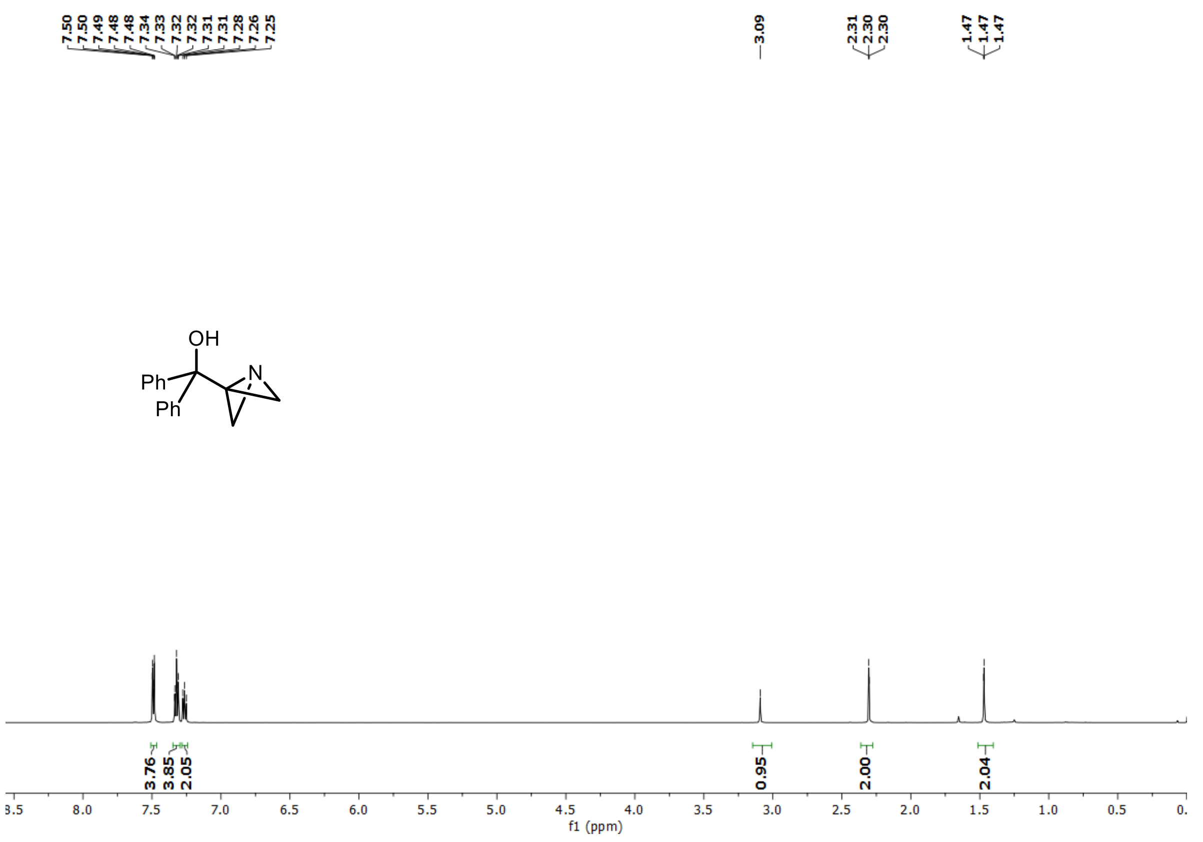

^{1}H spectra at 800 MHz in $CDCl_3$ (**2e**)

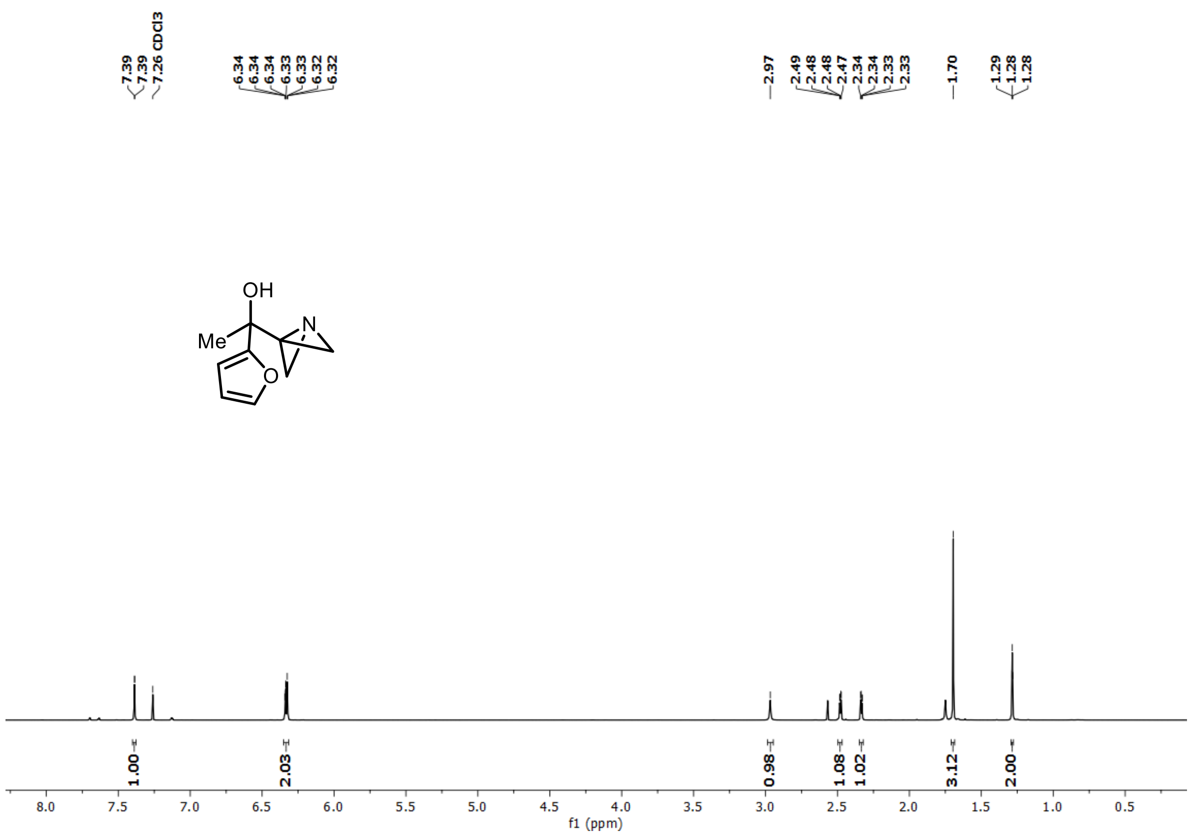

^{1}H spectra at 800 MHz in $CDCl_3$ (**2f**)

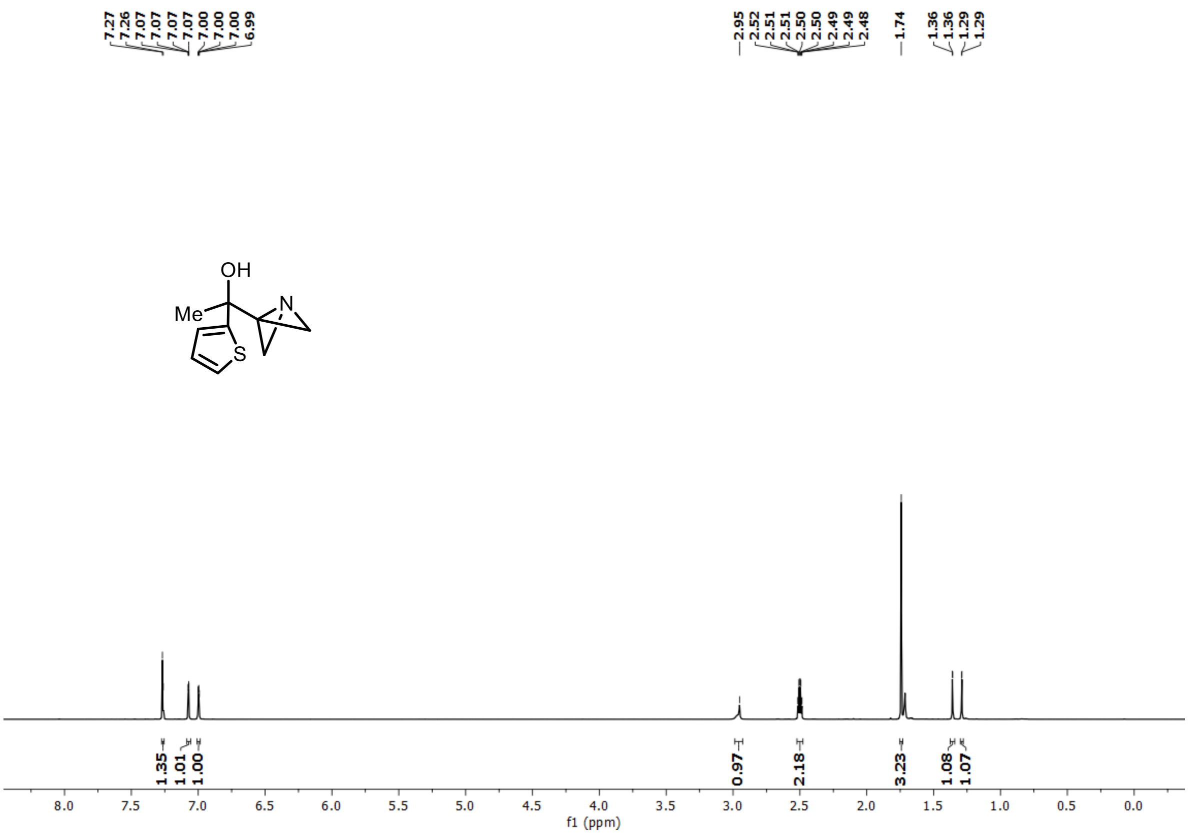

^{1}H spectra at 600 MHz in $CDCl_3$ (**2n**)

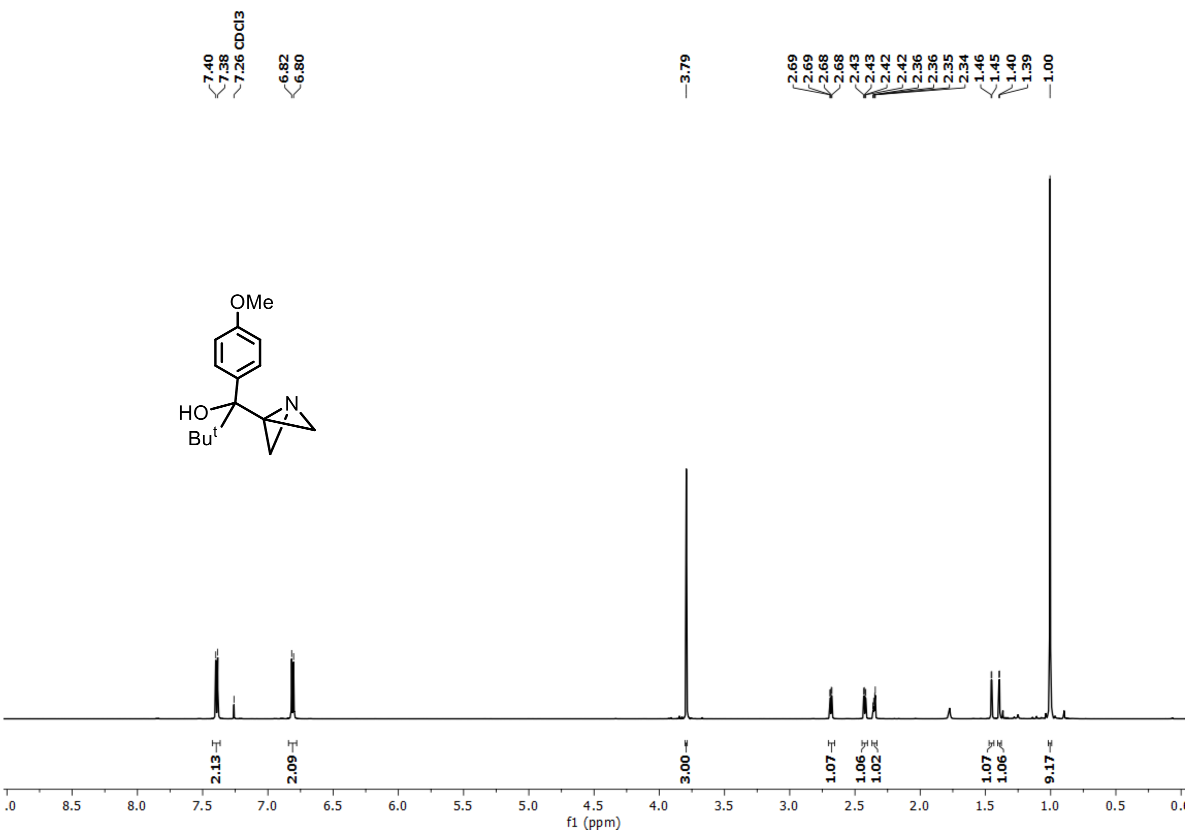

^{1}H spectra at 600 MHz in $CDCl_3$ (**2o**)

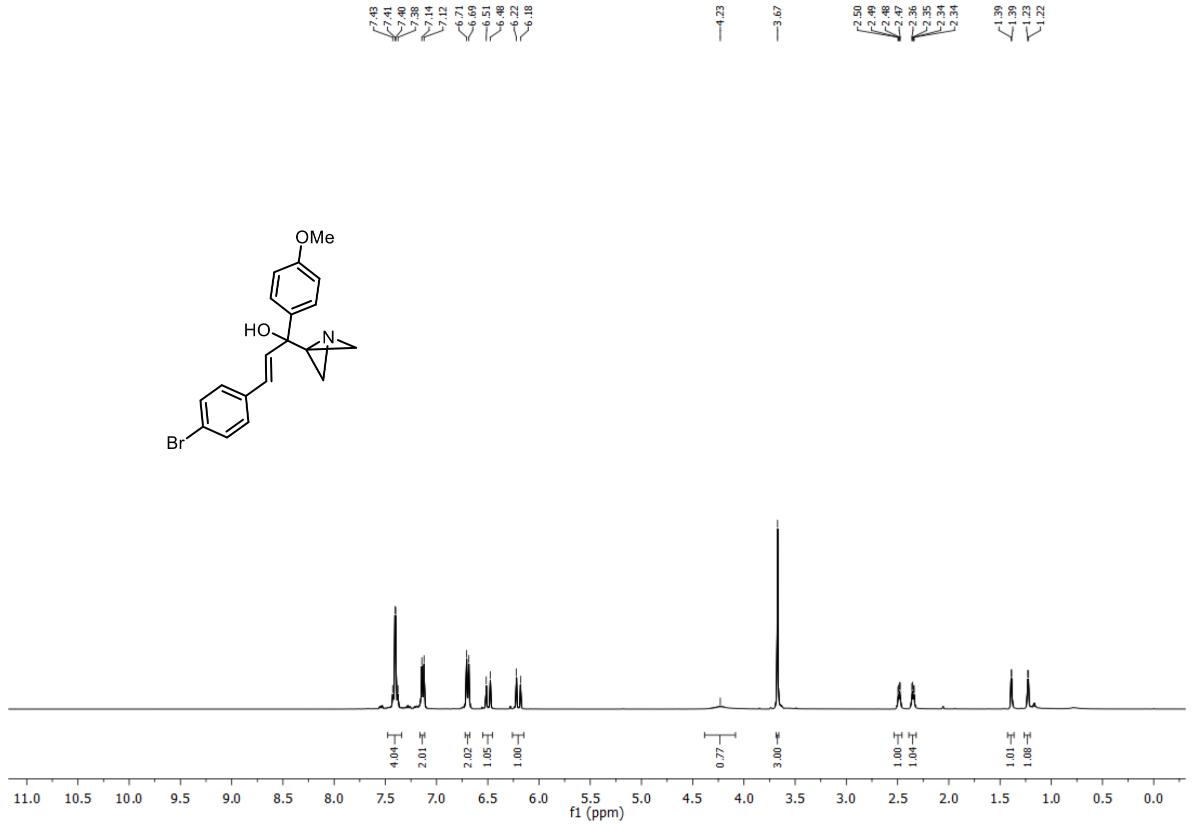

^{1}H spectra at 400 MHz in $CDCl_3$ (**2r**)

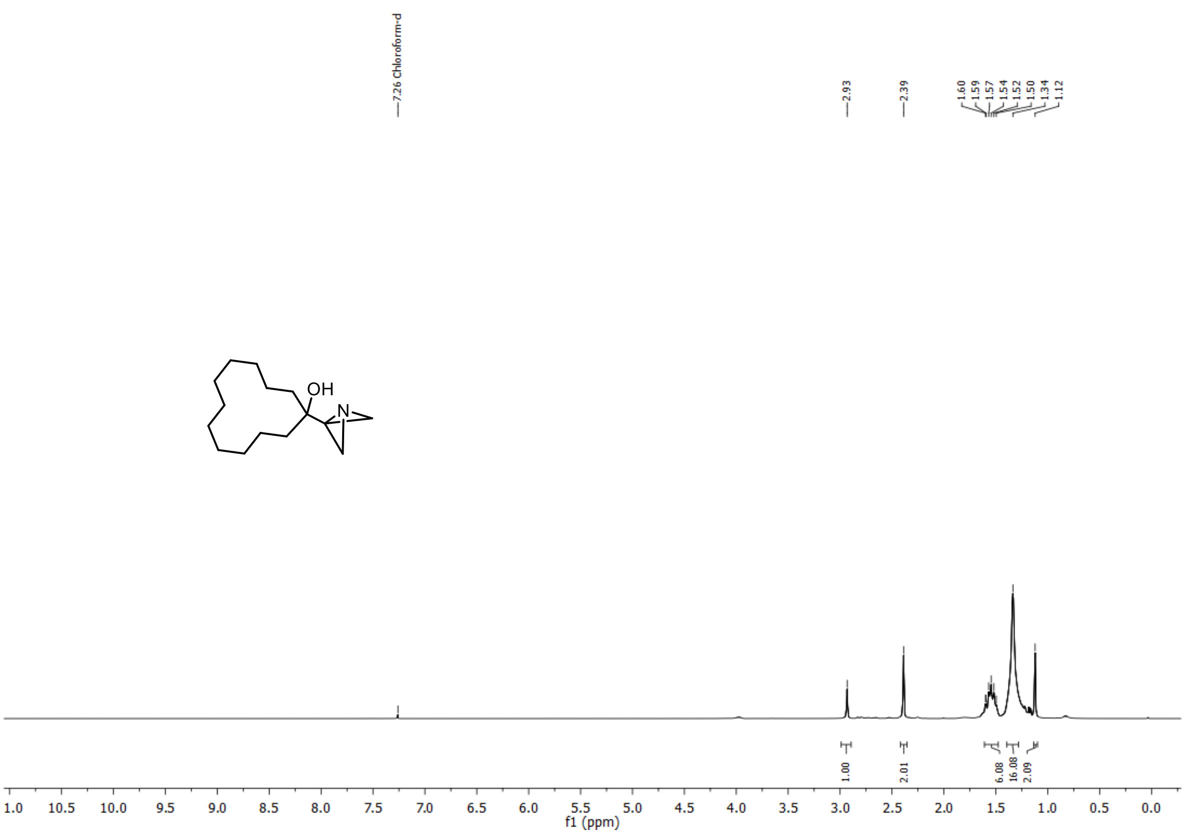

^1H spectra at 400 MHz in $CDCl_3$ (**2s**)

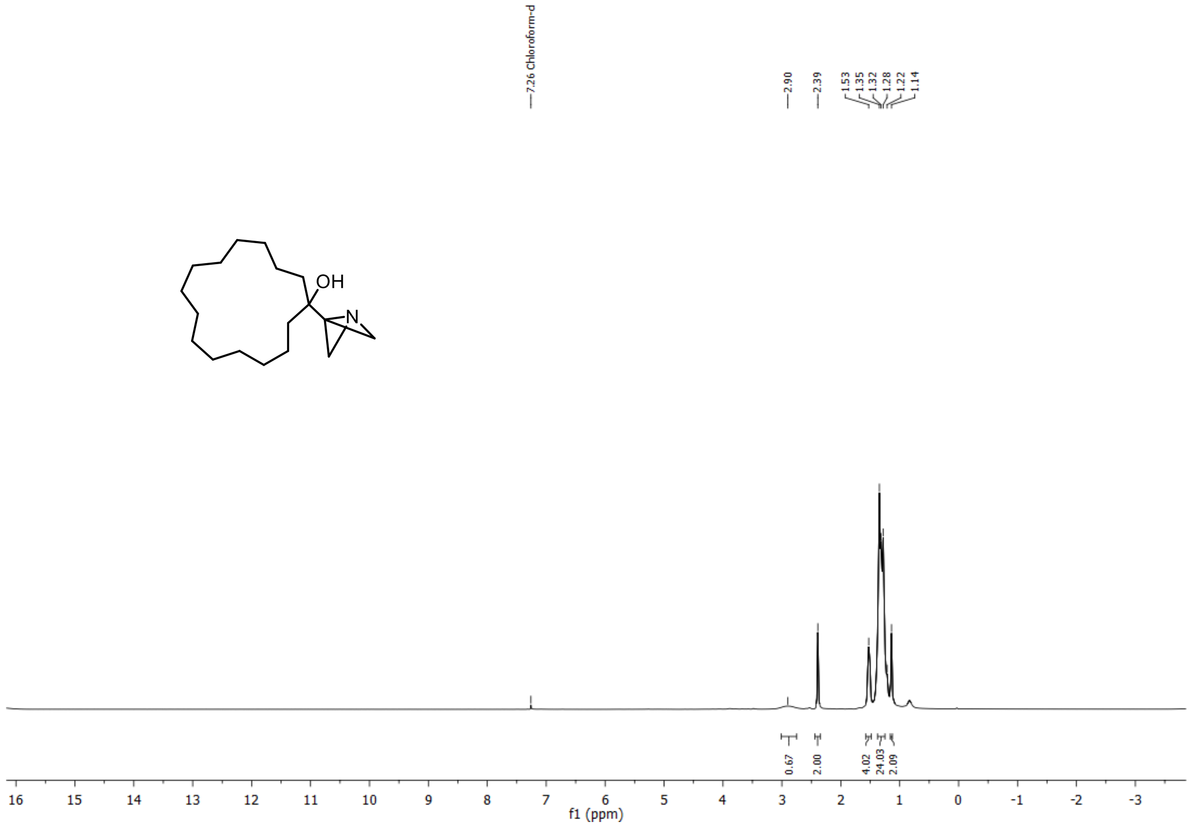

^1H spectra at 400 MHz in $CDCl_3$ (**2t**)

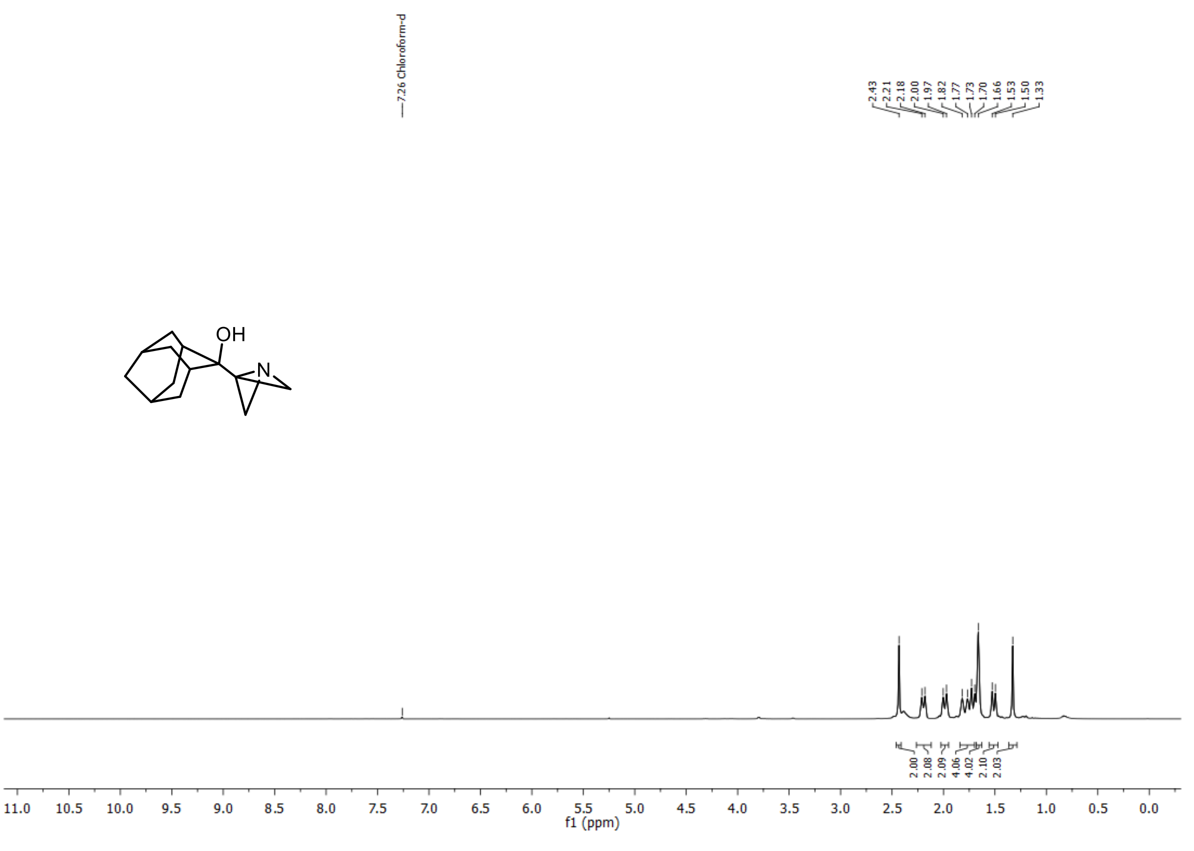

^{1}H spectra at 600 MHz in $CDCl_3$ (**1a''**)

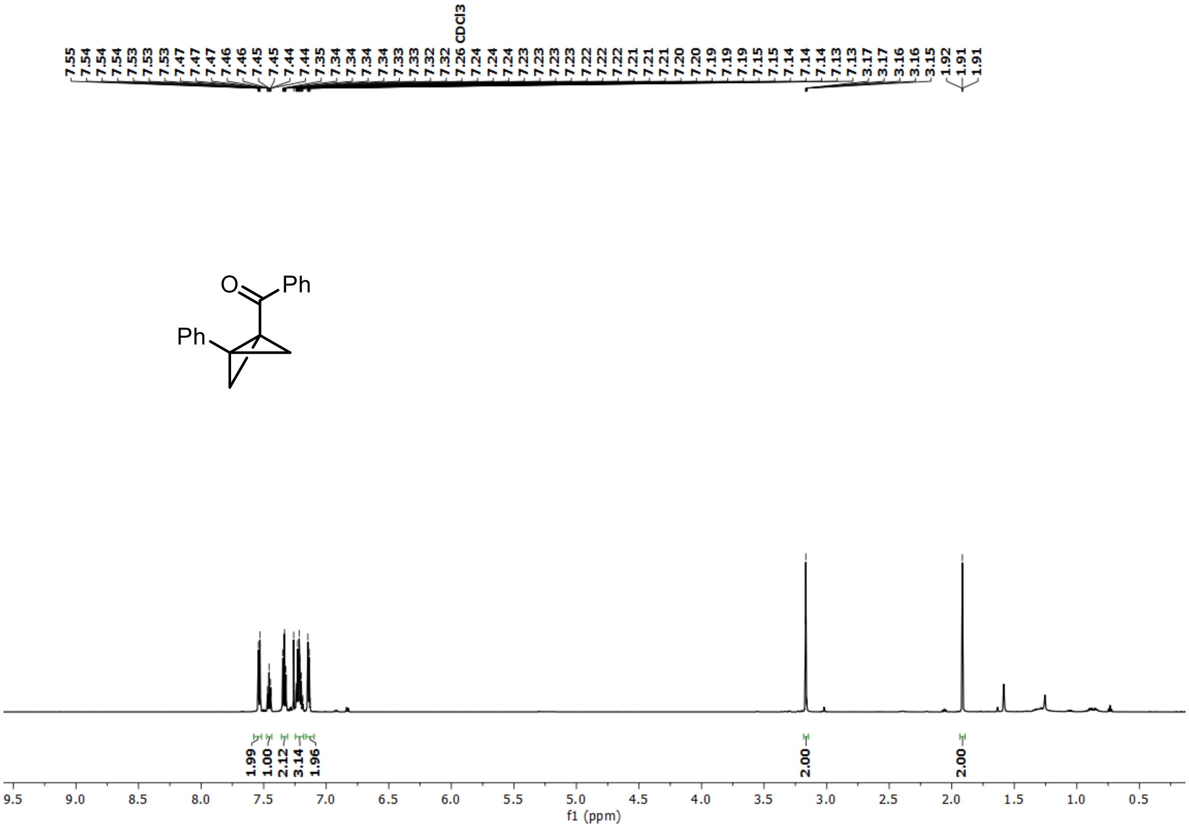

^{1}H spectra at 600 MHz in $CDCl_3$ (**1b''**)

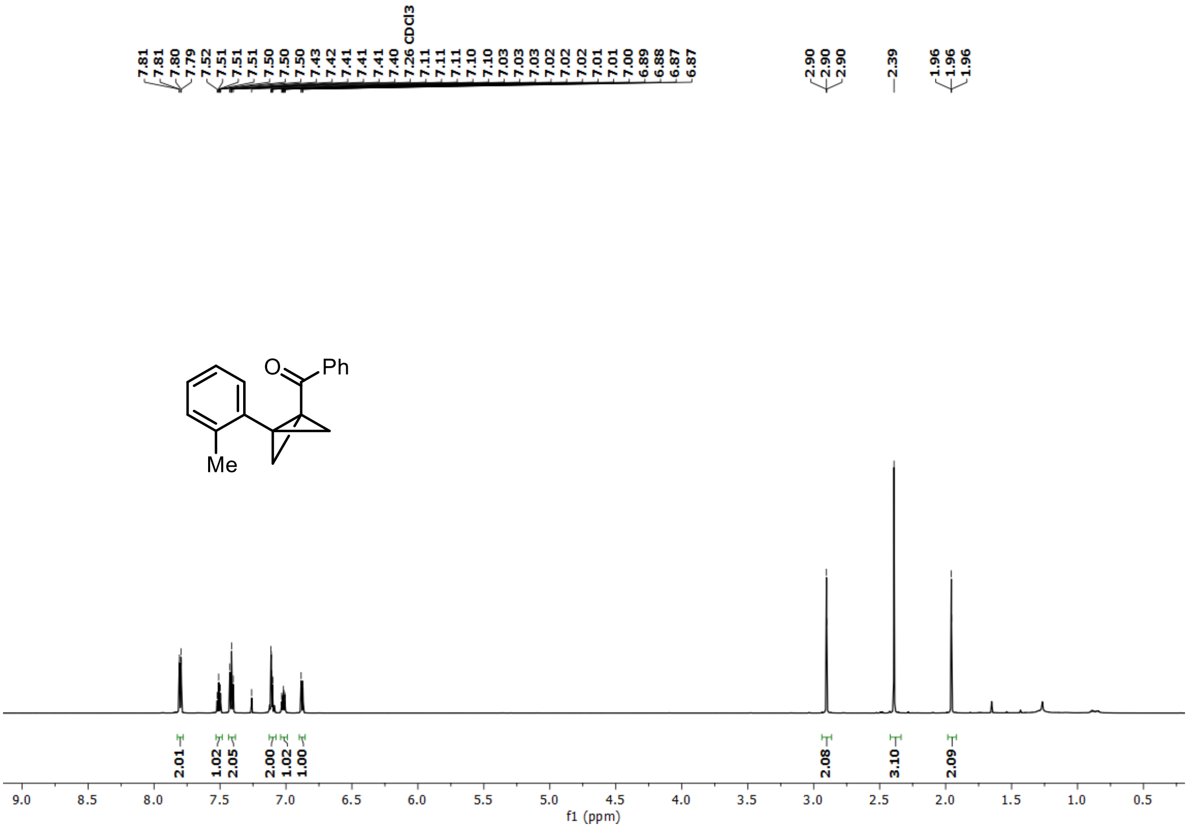

^{1}H spectra at 600 MHz in $CDCl_3$ (**1c"**)

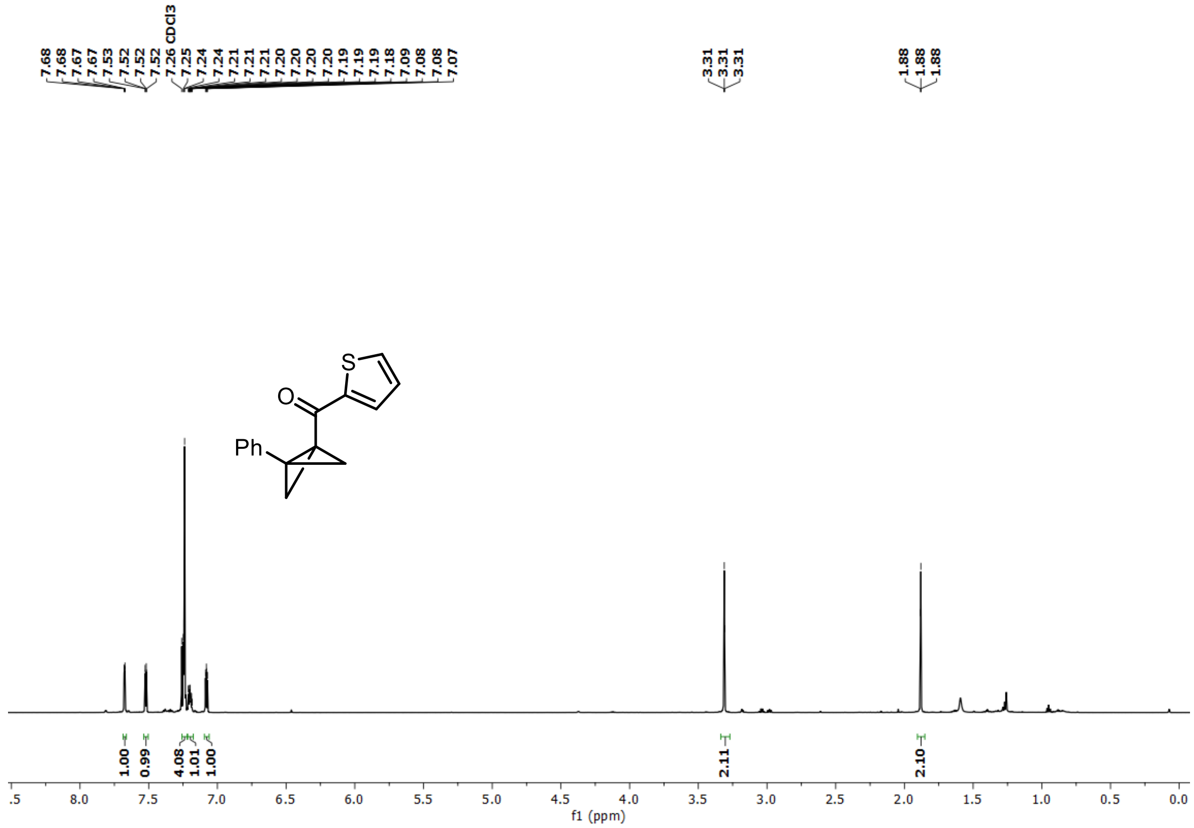

^{1}H spectra at 600 MHz in $CDCl_3$ (**1d"**)

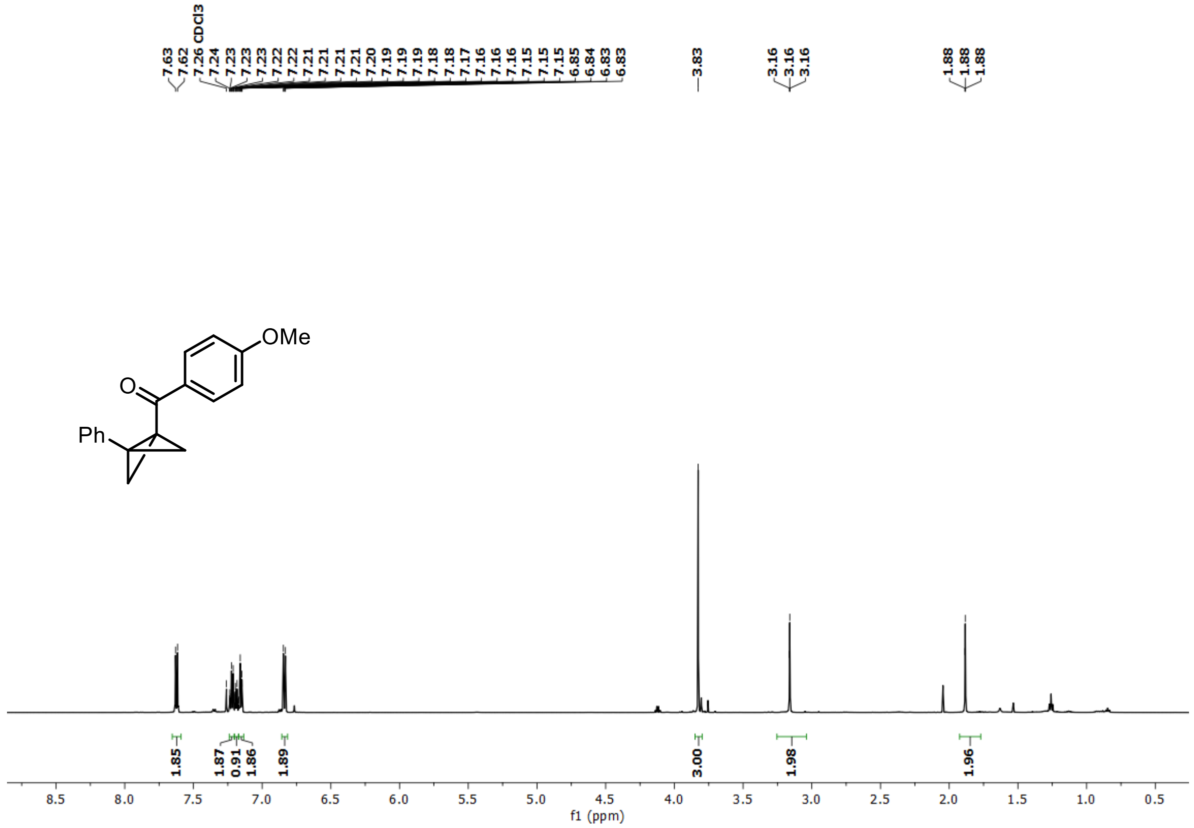

^{1}H spectra at 600 MHz in $CDCl_3$ (**1e"**)

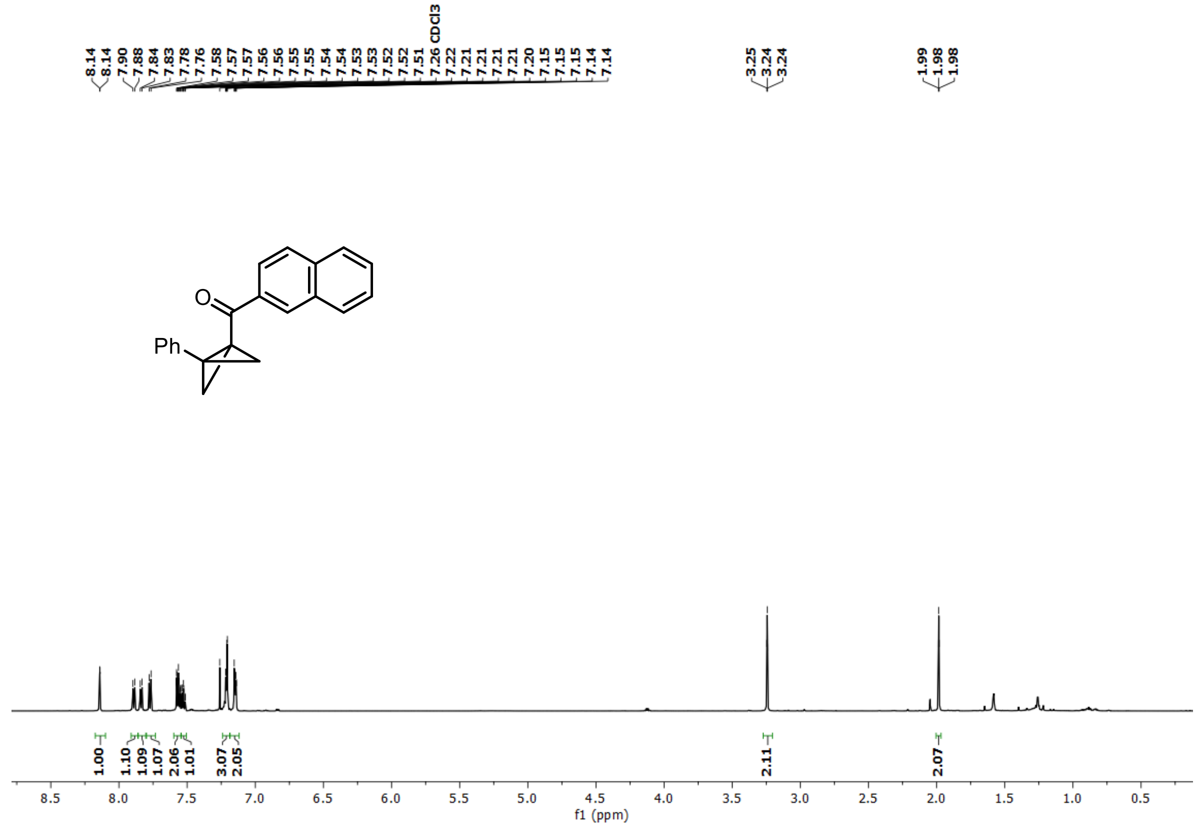

18.0 NMR spectra of new compounds (3-69)

^{1}H spectra at 400 MHz and ^{13}C{^{1}H} NMR spectra at 100 MHz in $CDCl_3$ (**3**)

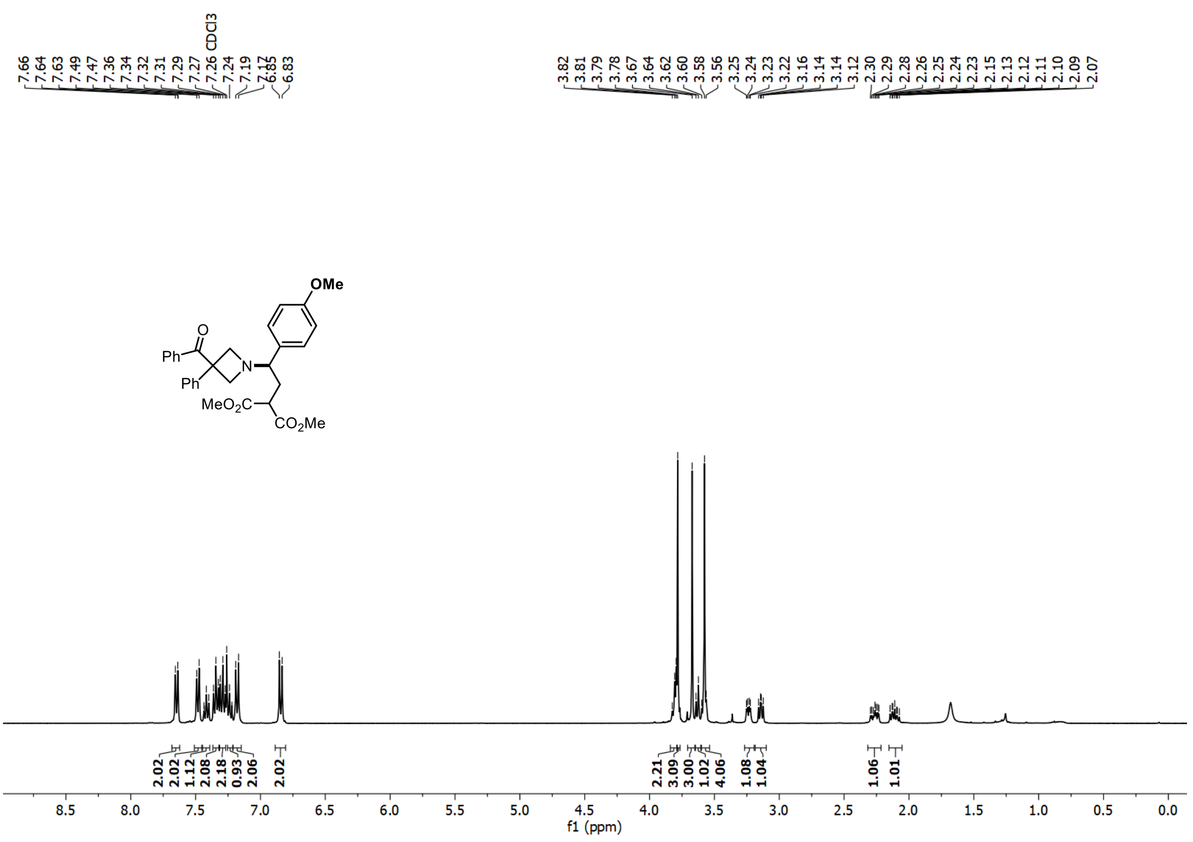

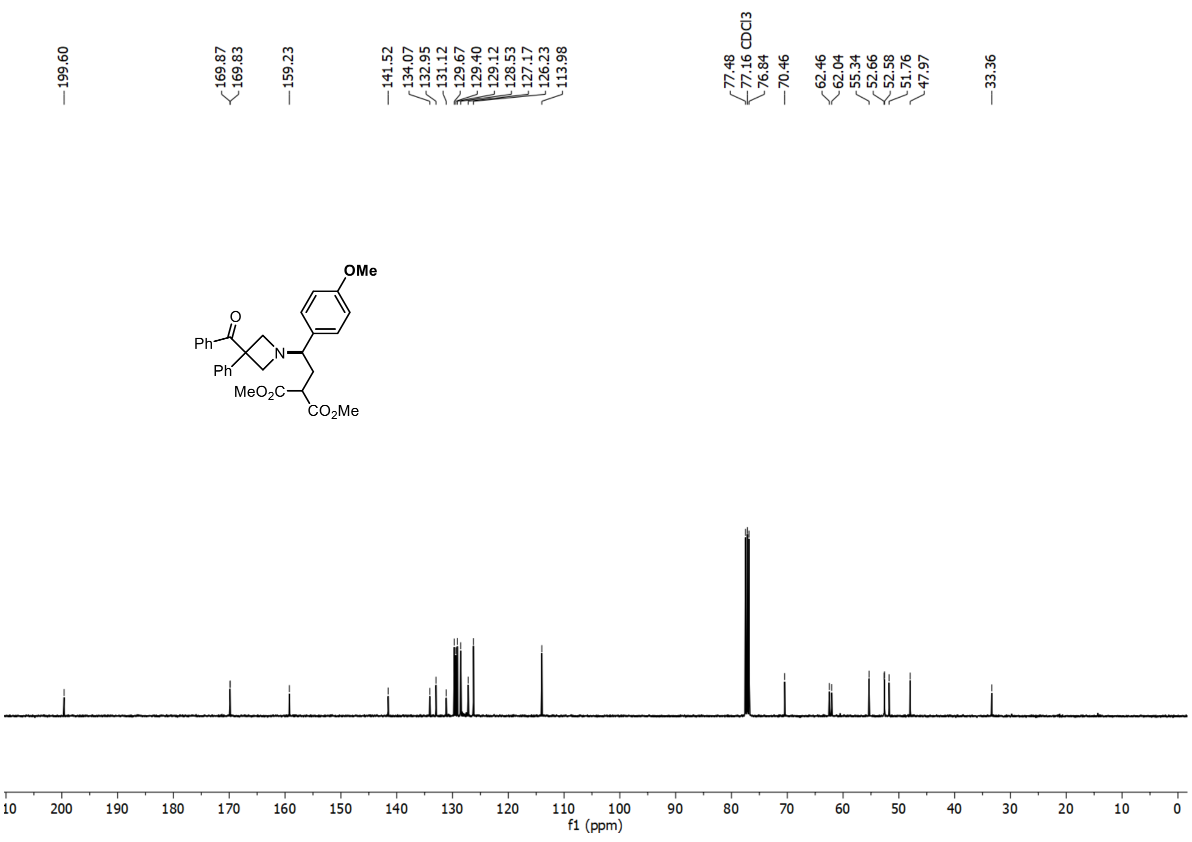

DEPT spectra at 400 MHz 100 MHz in $CDCl_3$ (**3**)

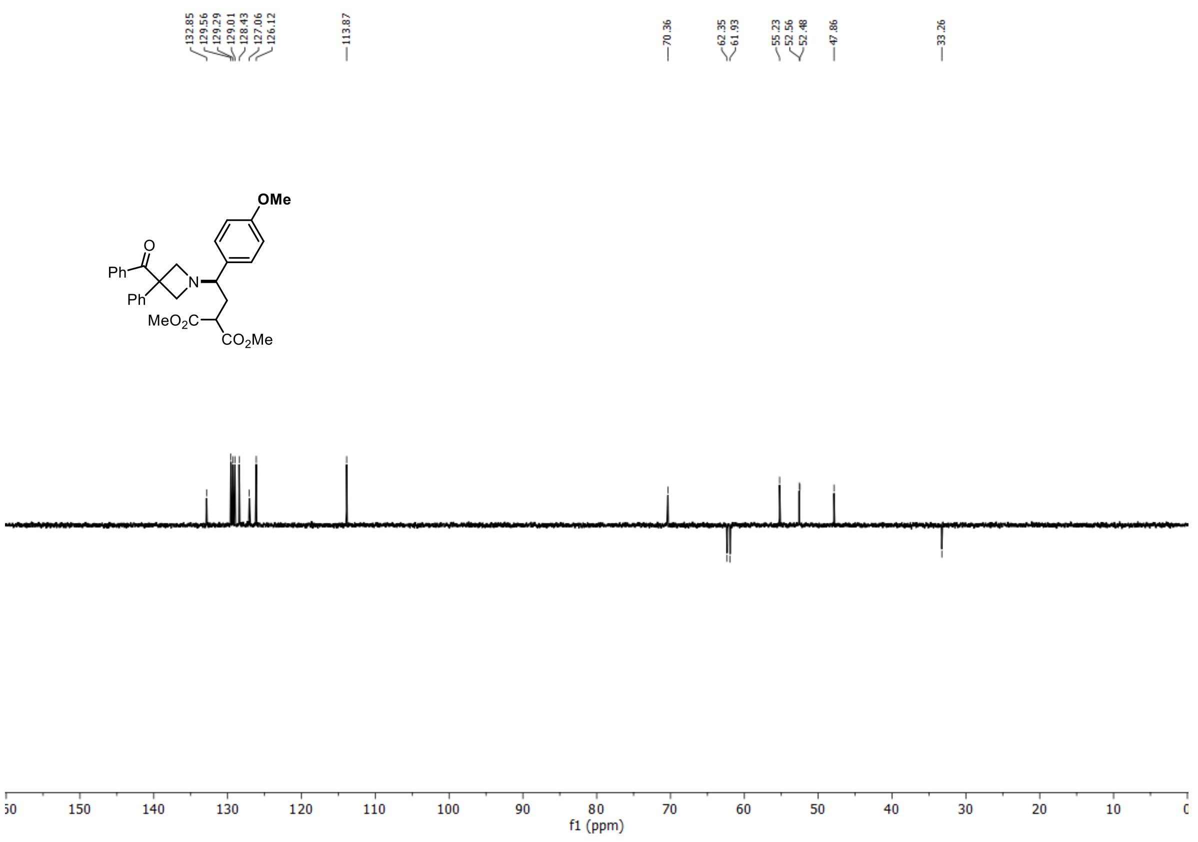

^{1}H spectra at 400 MHz and ^{13}C{^{1}H} NMR spectra at 100 MHz in DMSO- d6 (**4**)

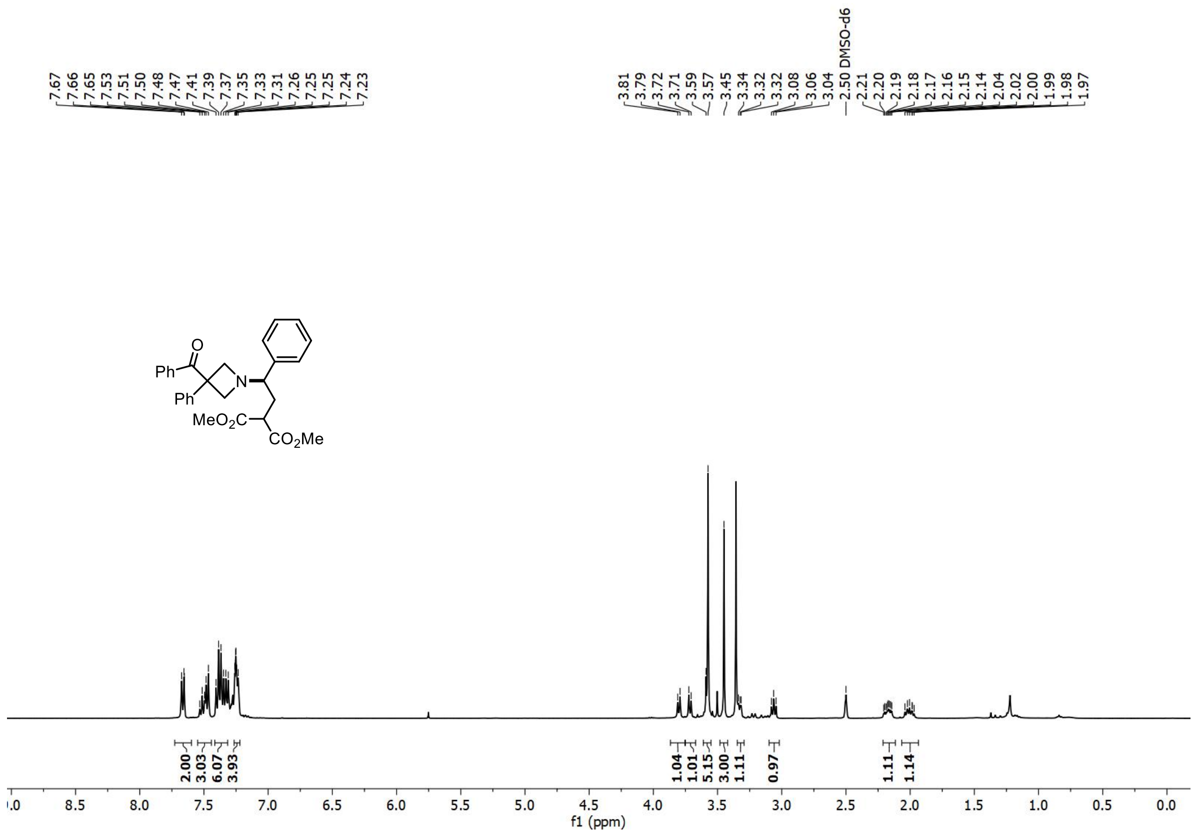

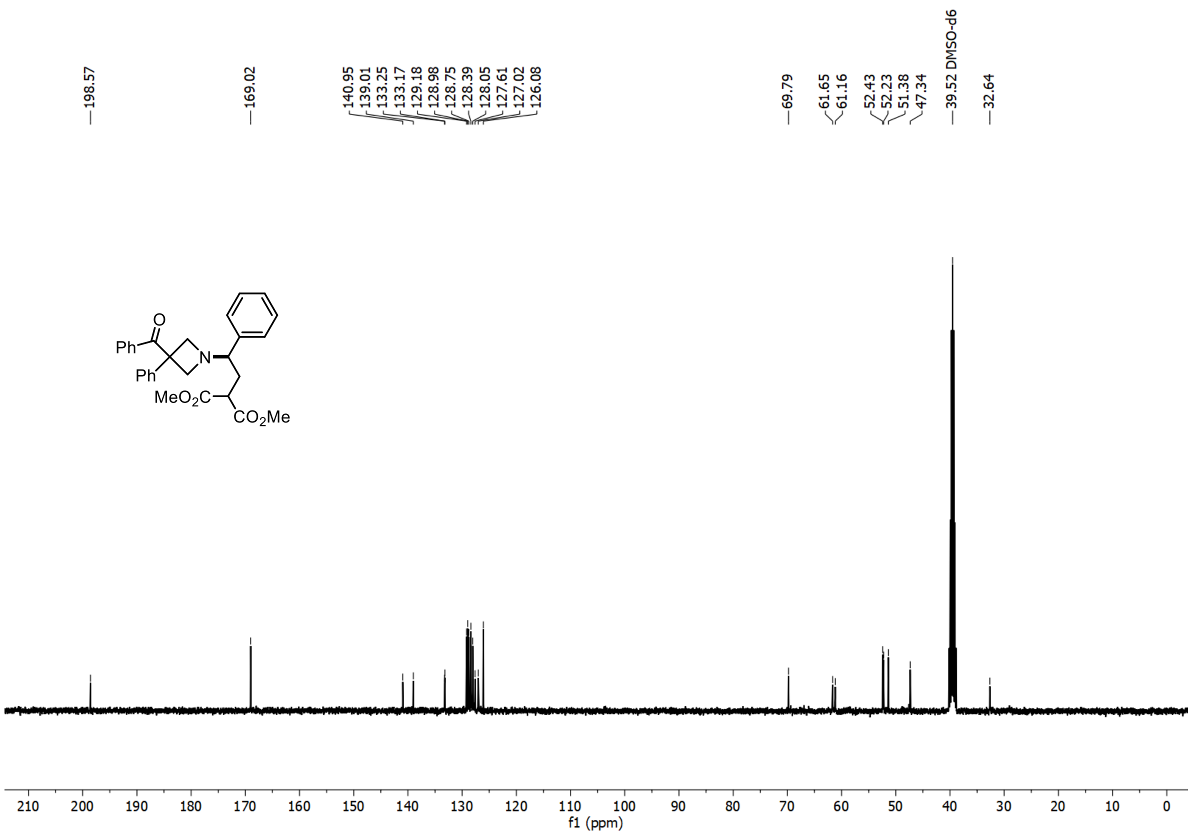

^{1}H spectra at 400 MHz and $^{13}C\{^{1}H\}$ NMR spectra at 100 MHz in DMSO- d6 (**5**)

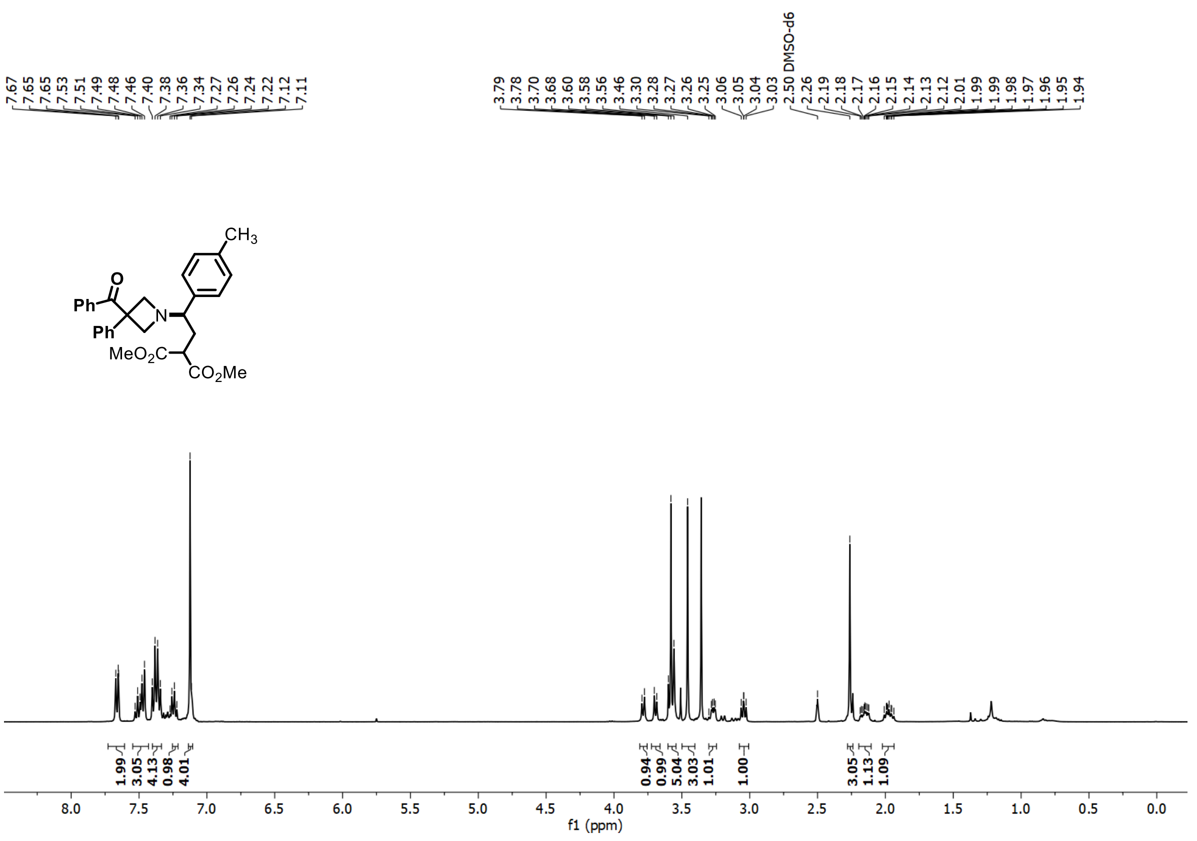

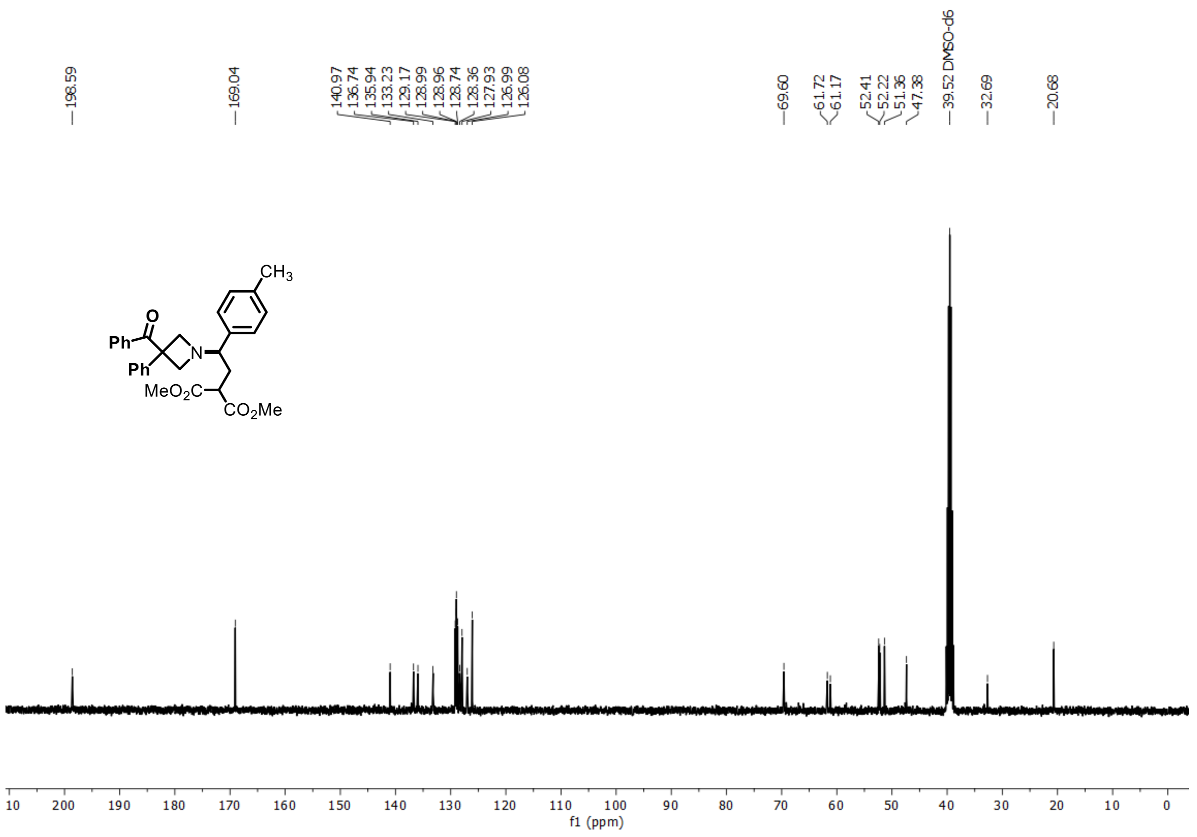

^{1}H spectra at 600 MHz and ^{13}C{^{1}H} NMR spectra at 150 MHz in DMSO-d6 (**6**)

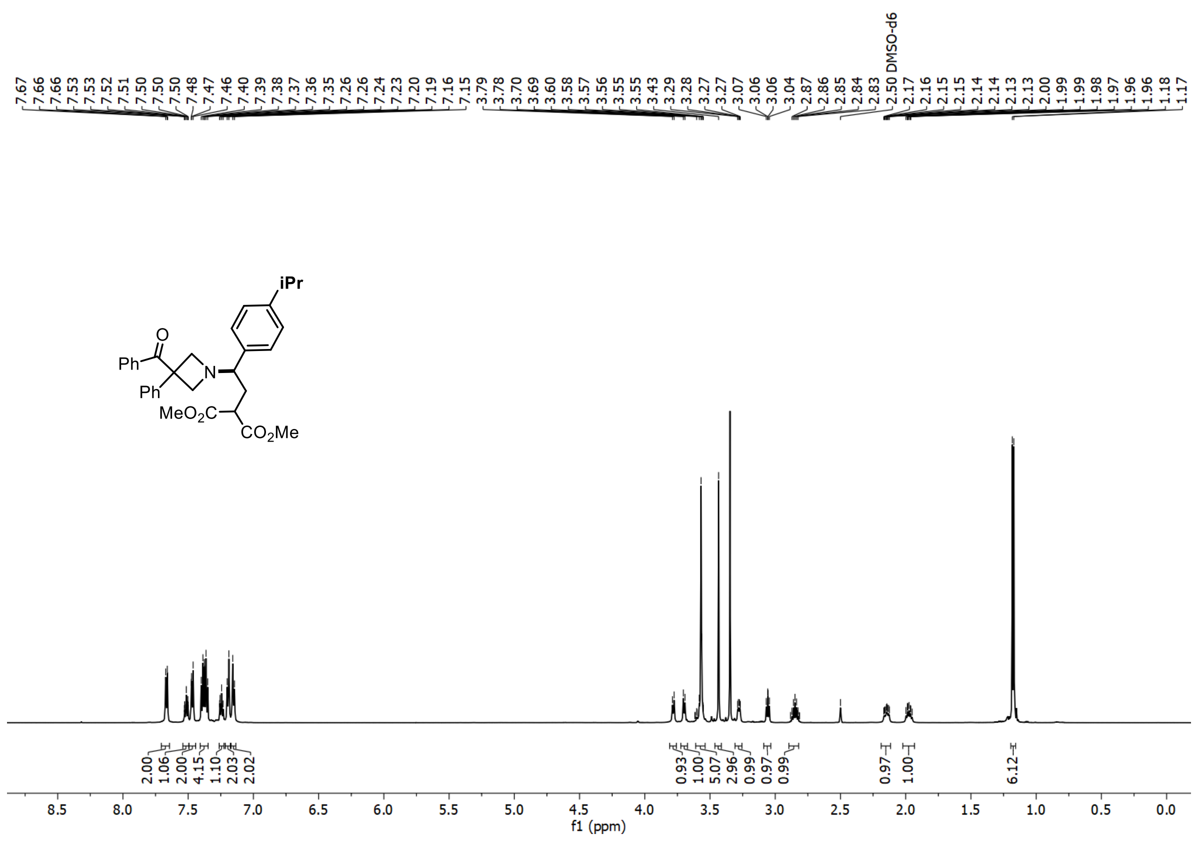

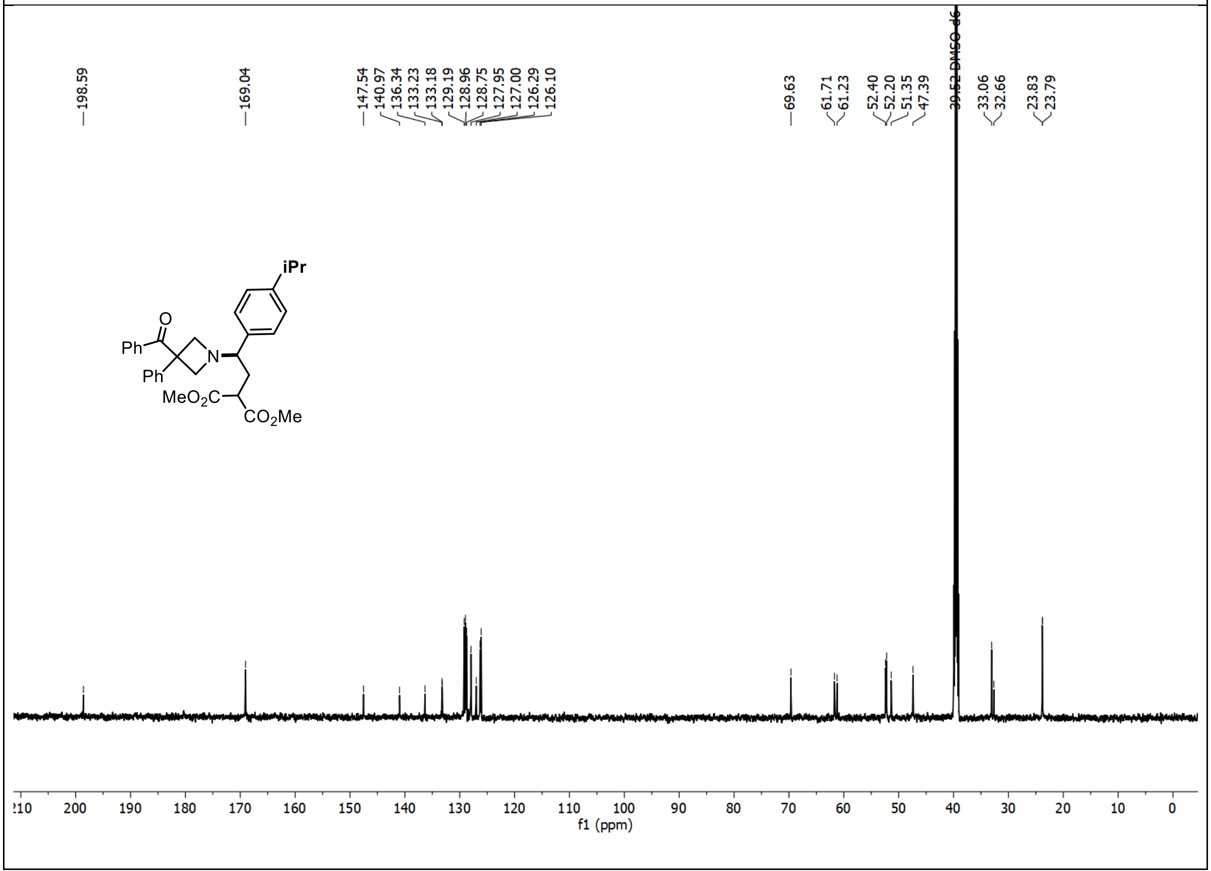

^{1}H spectra at 600 MHz and $^{13}C\{^1H\}$ NMR spectra at 150 MHz in DMSO-d6 (**7**)

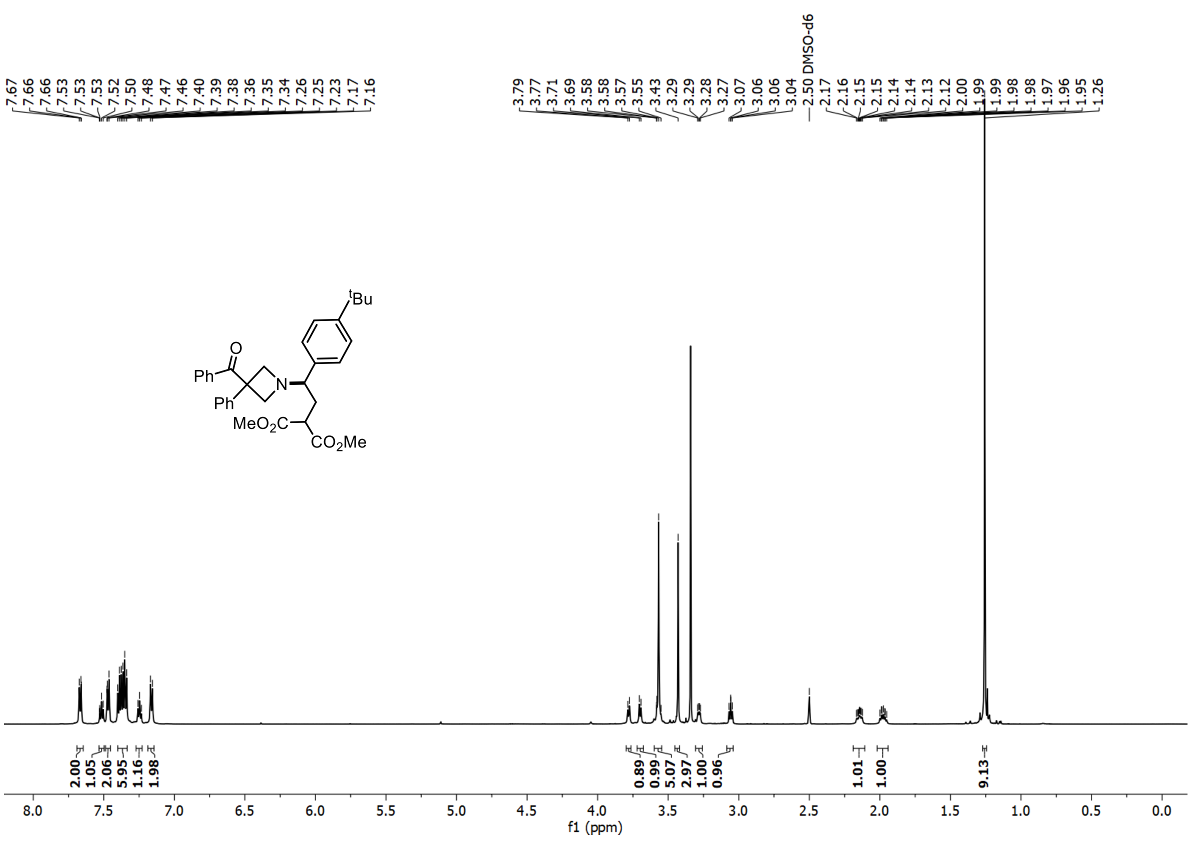

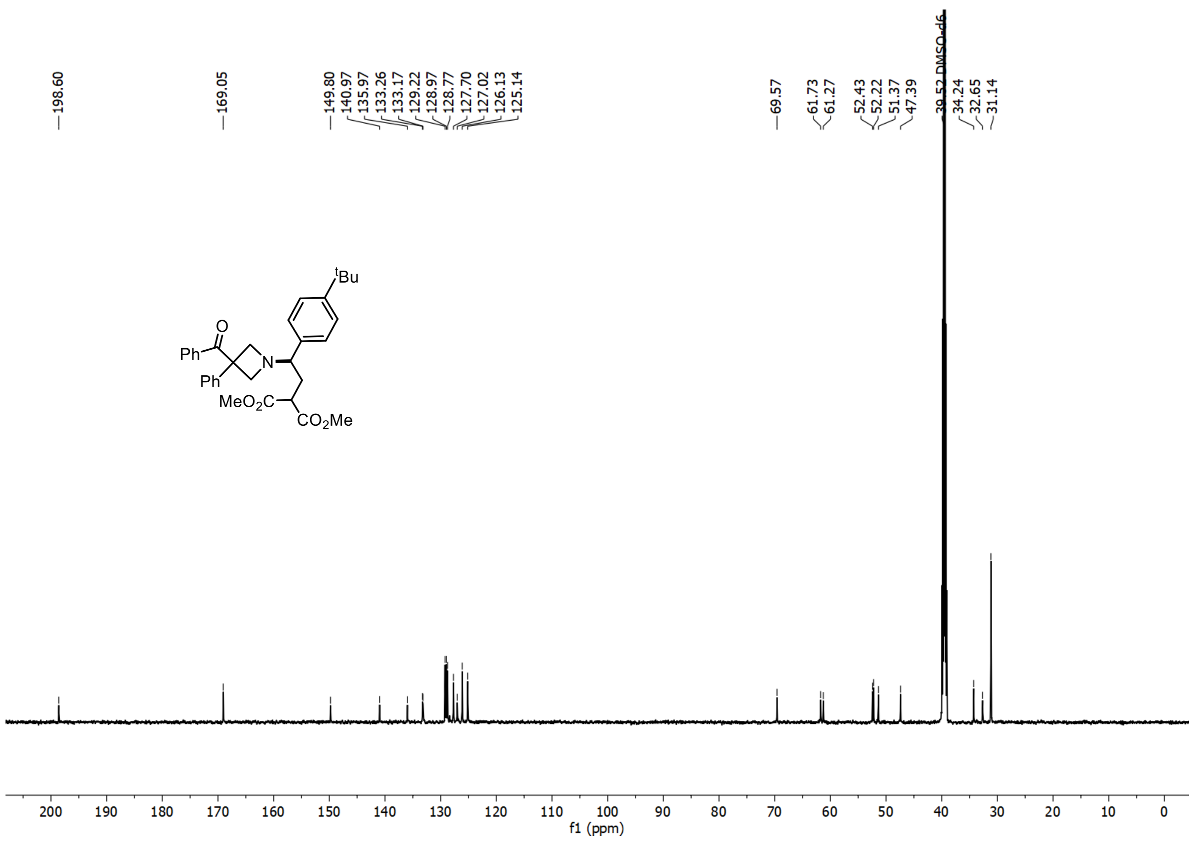

^{1}H spectra at 400 MHz and ^{13}C{^{1}H} NMR spectra at 100 MHz in $CDCl_3$ (**08**)

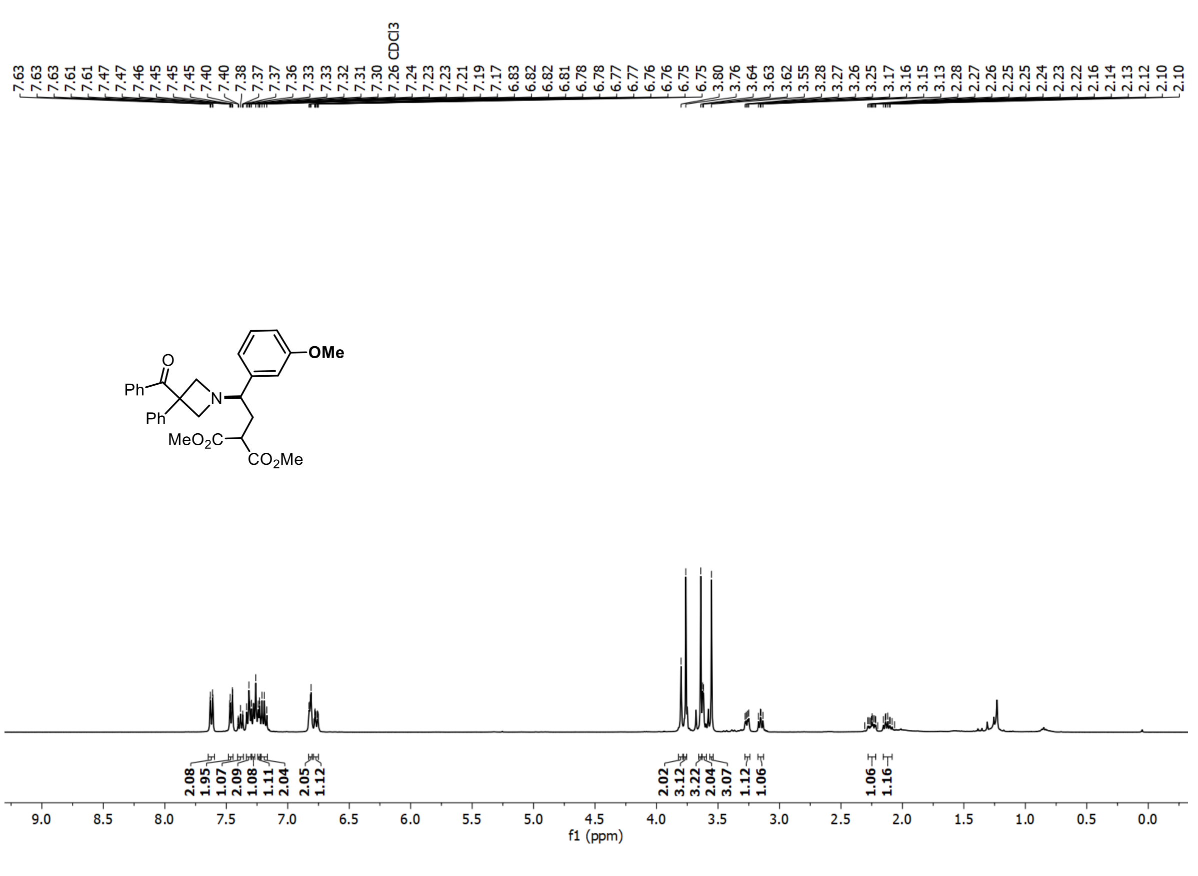

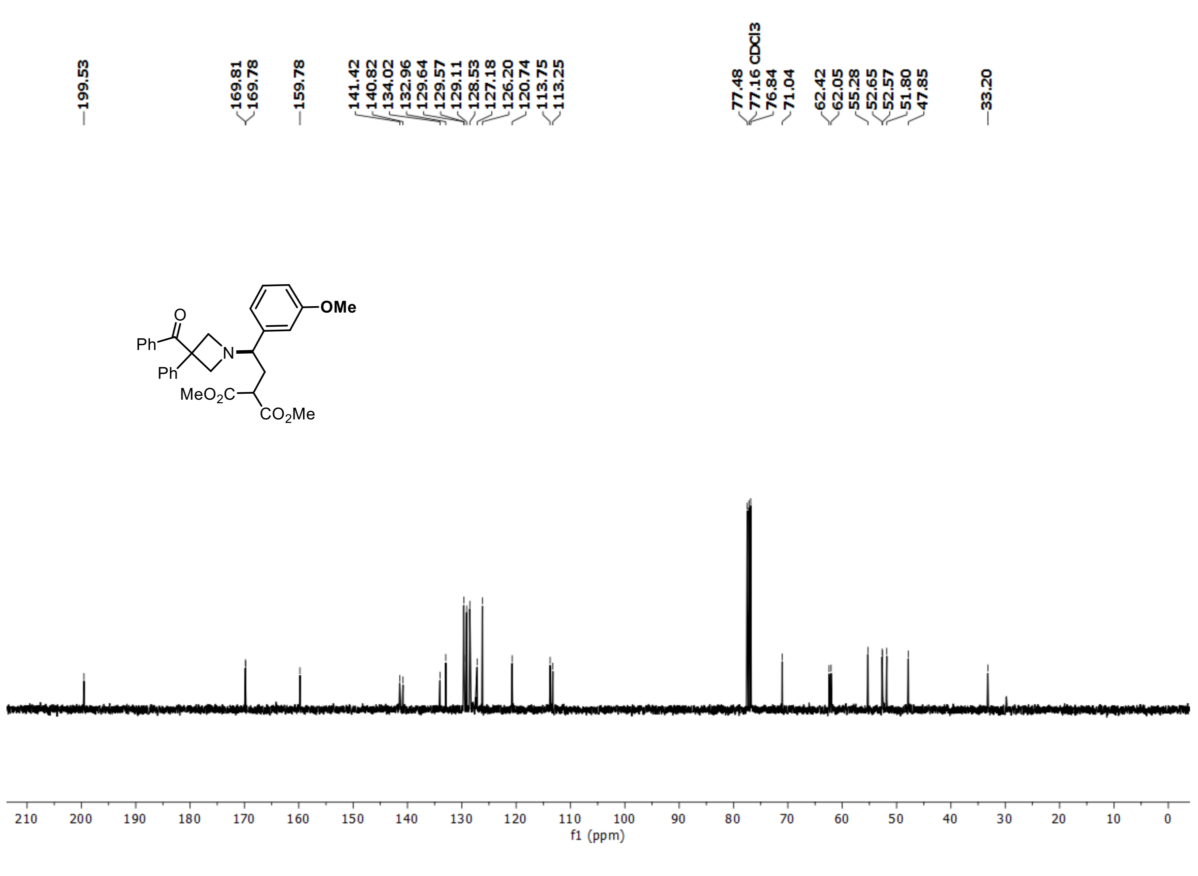

^{1}H spectra at 400 MHz and ^{13}C{^{1}H} NMR spectra at 100 MHz in $CDCl_3$ (**09**)

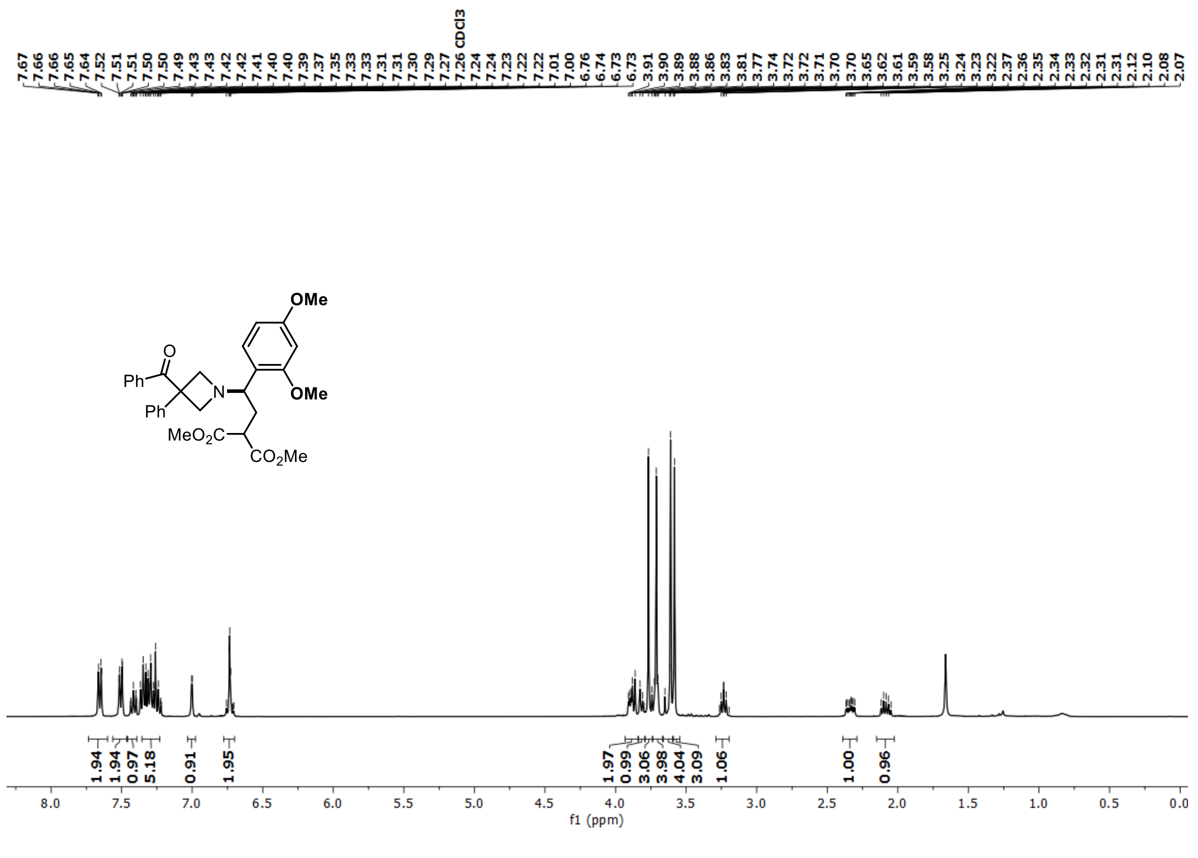

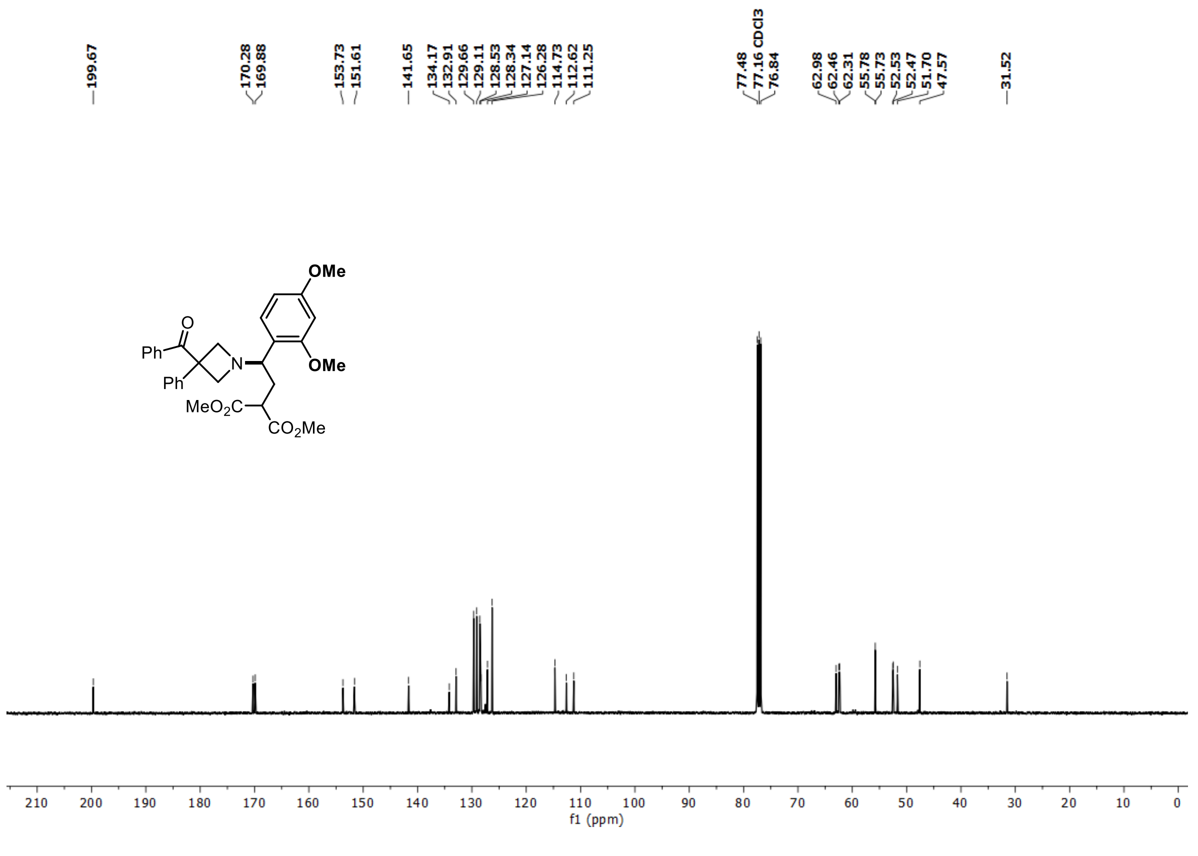

^{1}H spectra at 400 MHz and ^{13}C{^{1}H} NMR spectra at 100 MHz in $CDCl_3$ (**10**)

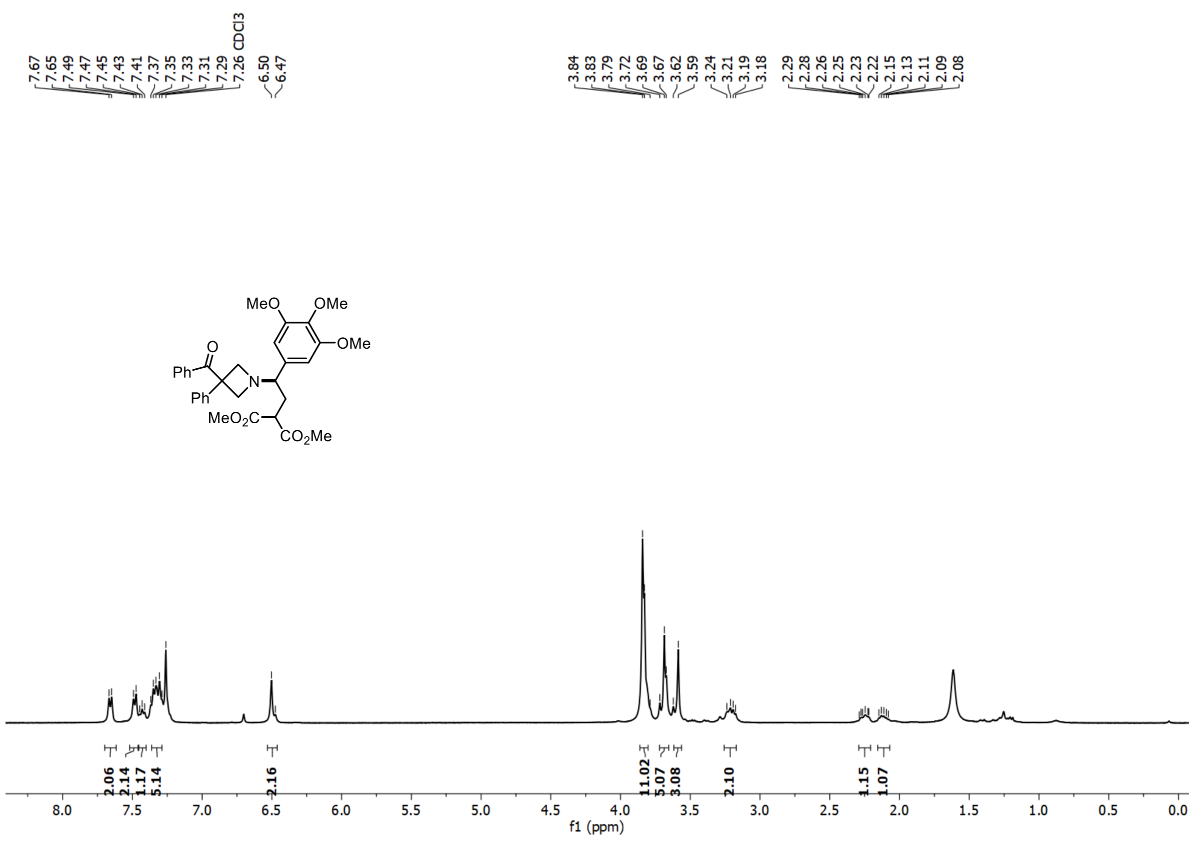

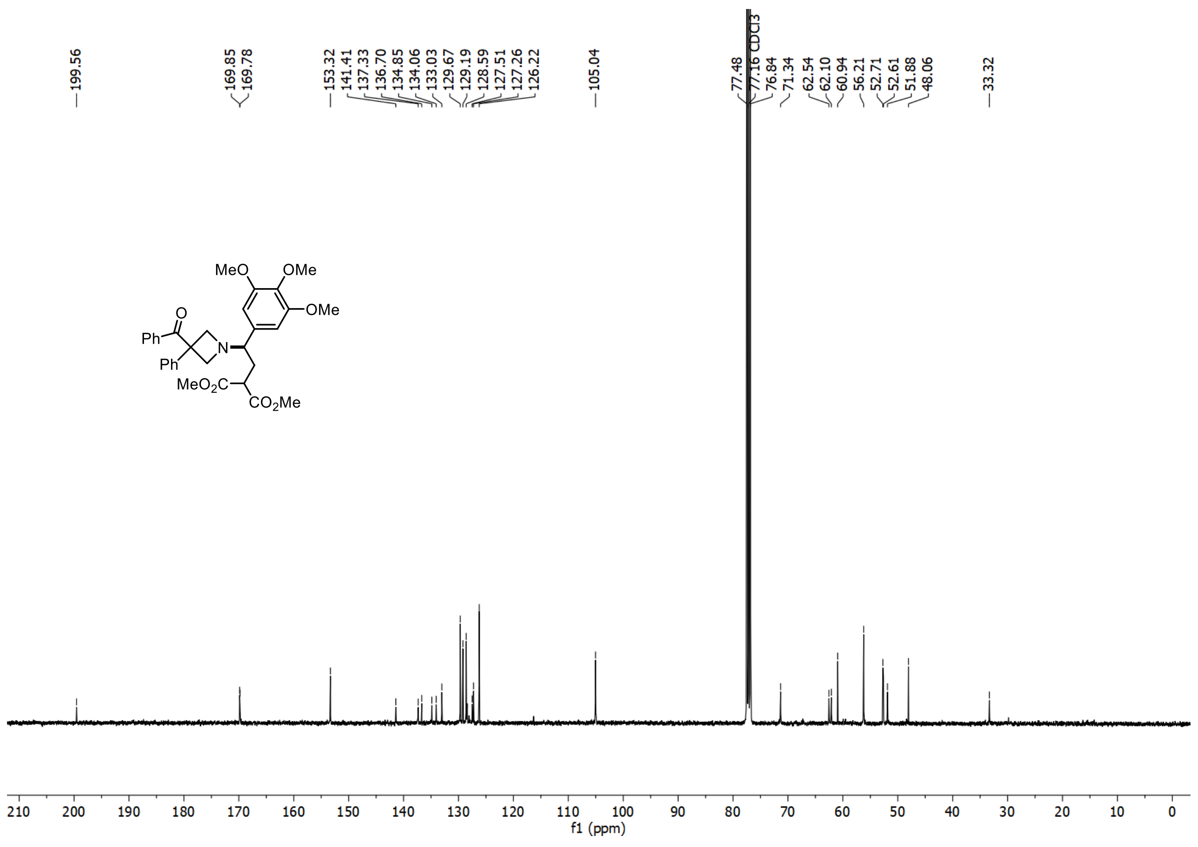

^{1}H spectra at 400 MHz and $^{13}C\{^1H\}$ NMR spectra at 100 MHz in $CDCl_3$ (**11**)

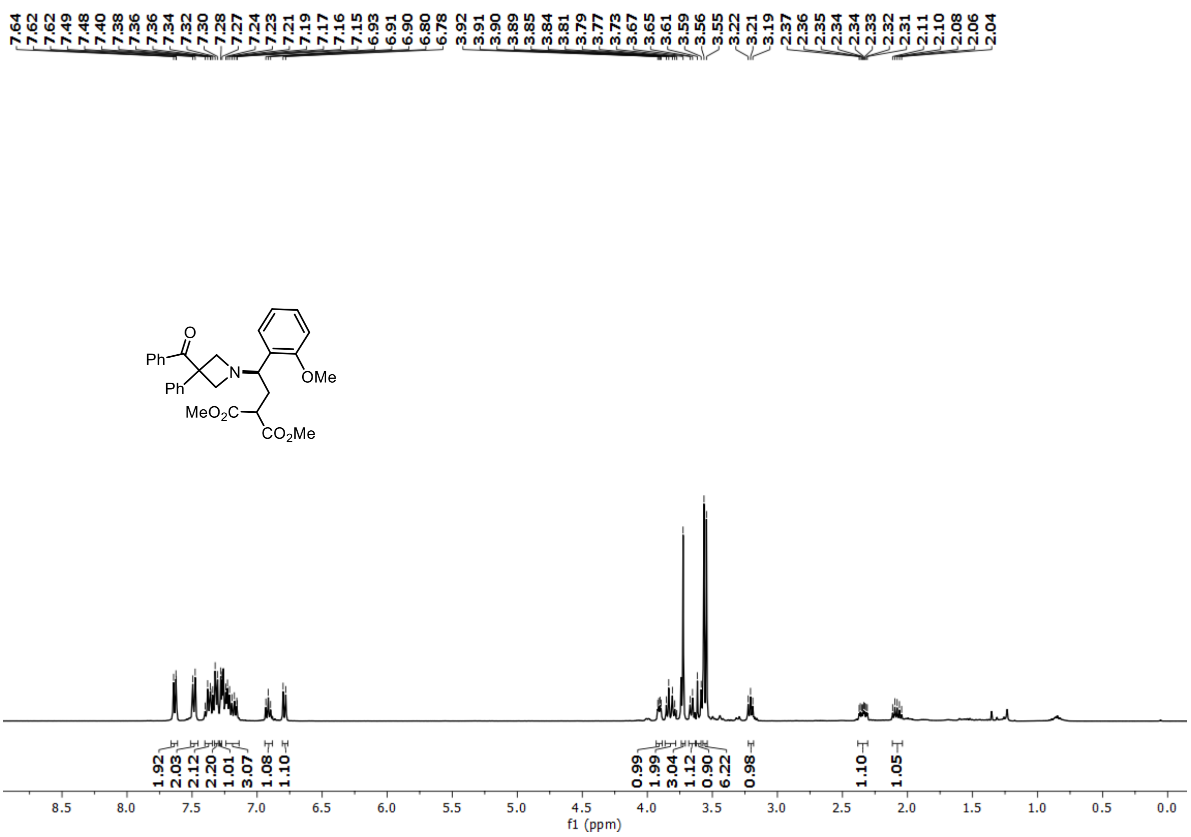

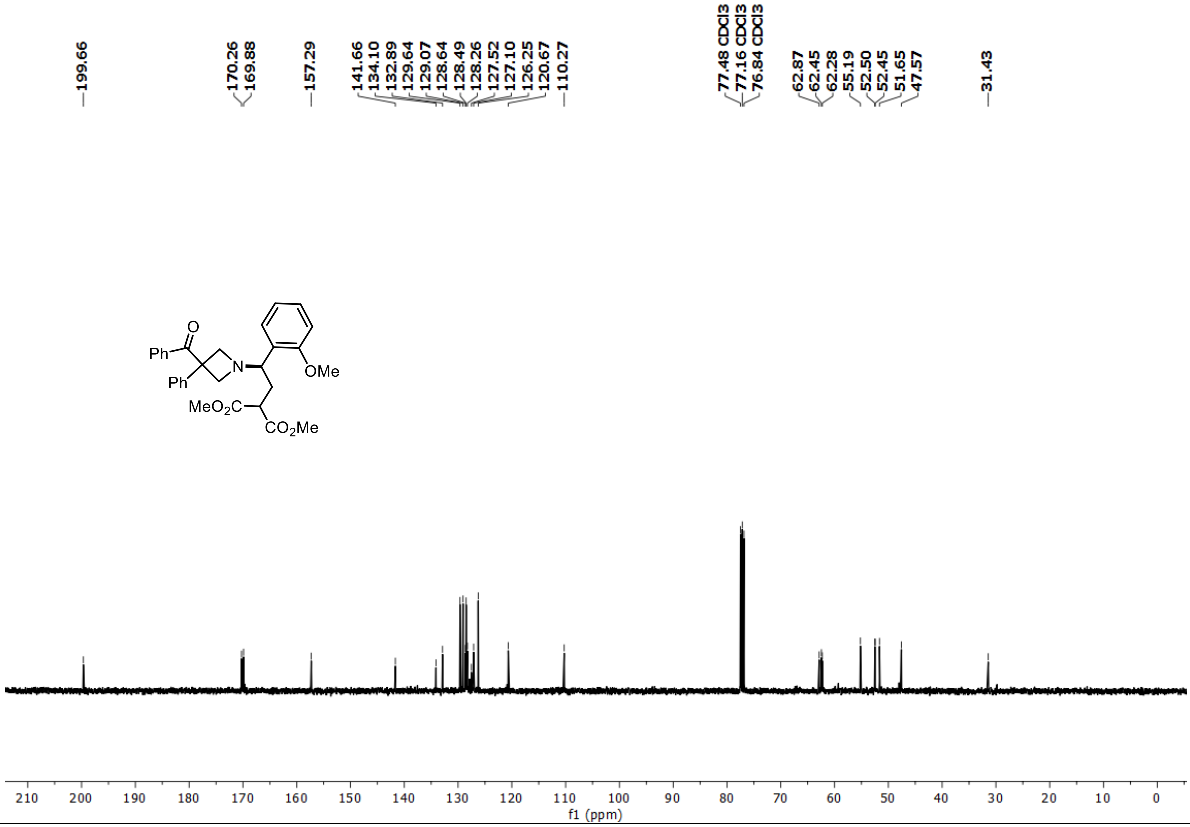

^{1}H spectra at 400 MHz and $^{13}C\{^{1}H\}$ NMR spectra at 100 MHz in $CDCl_3$ (**12**)

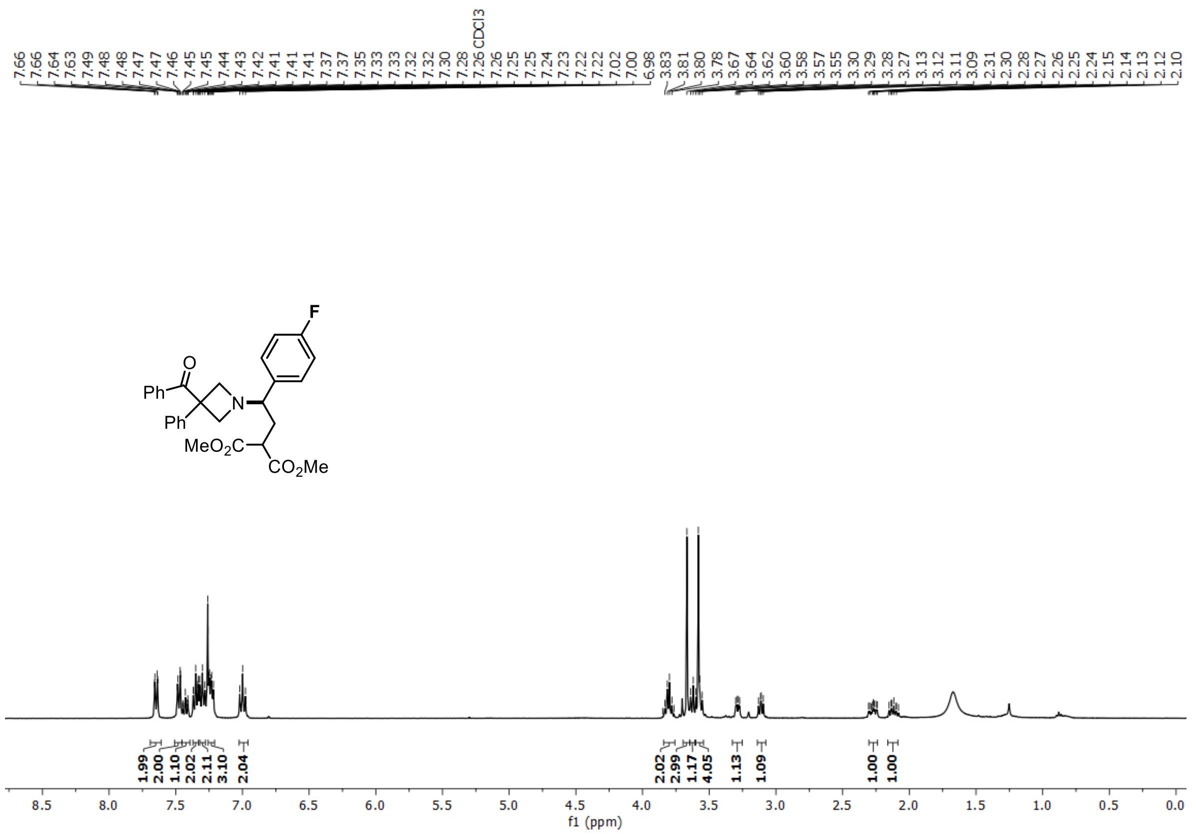

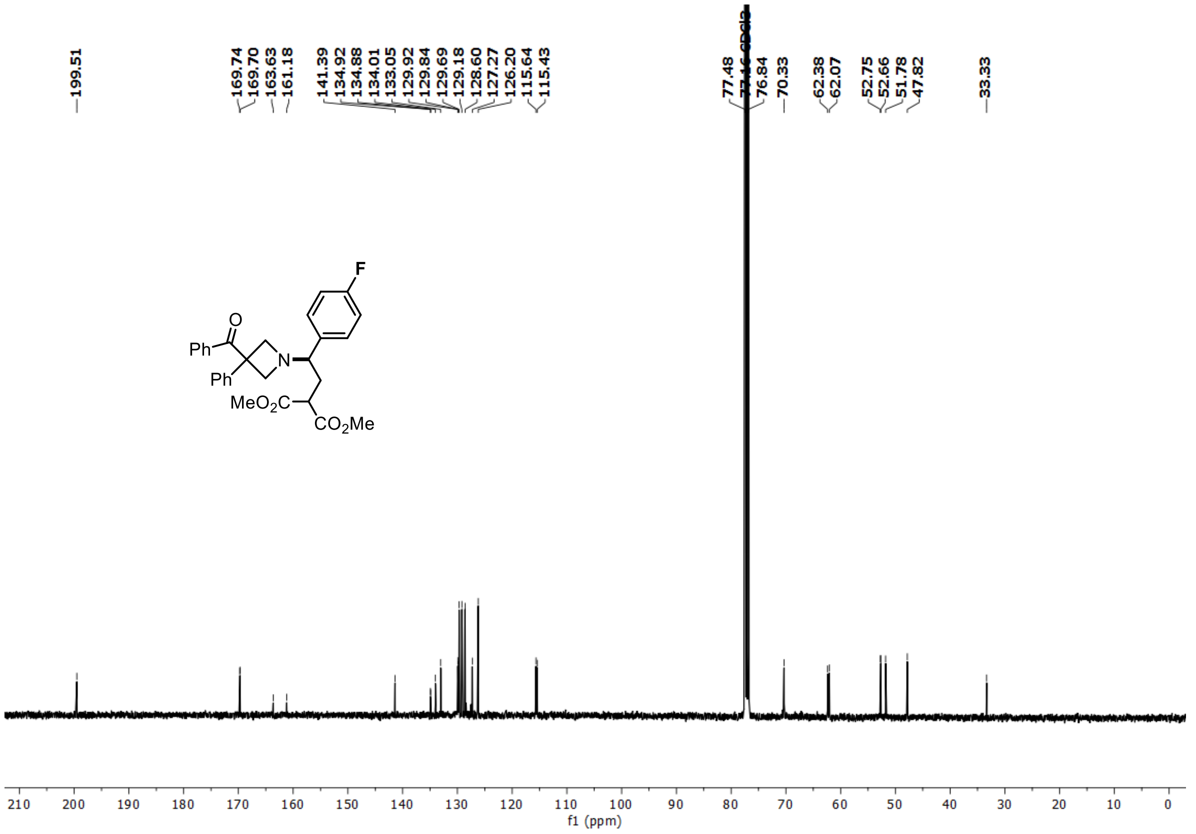

^{19}Fspectra at 374 MHz NMR spectra at 100 MHz in CDCl3 (**12**)

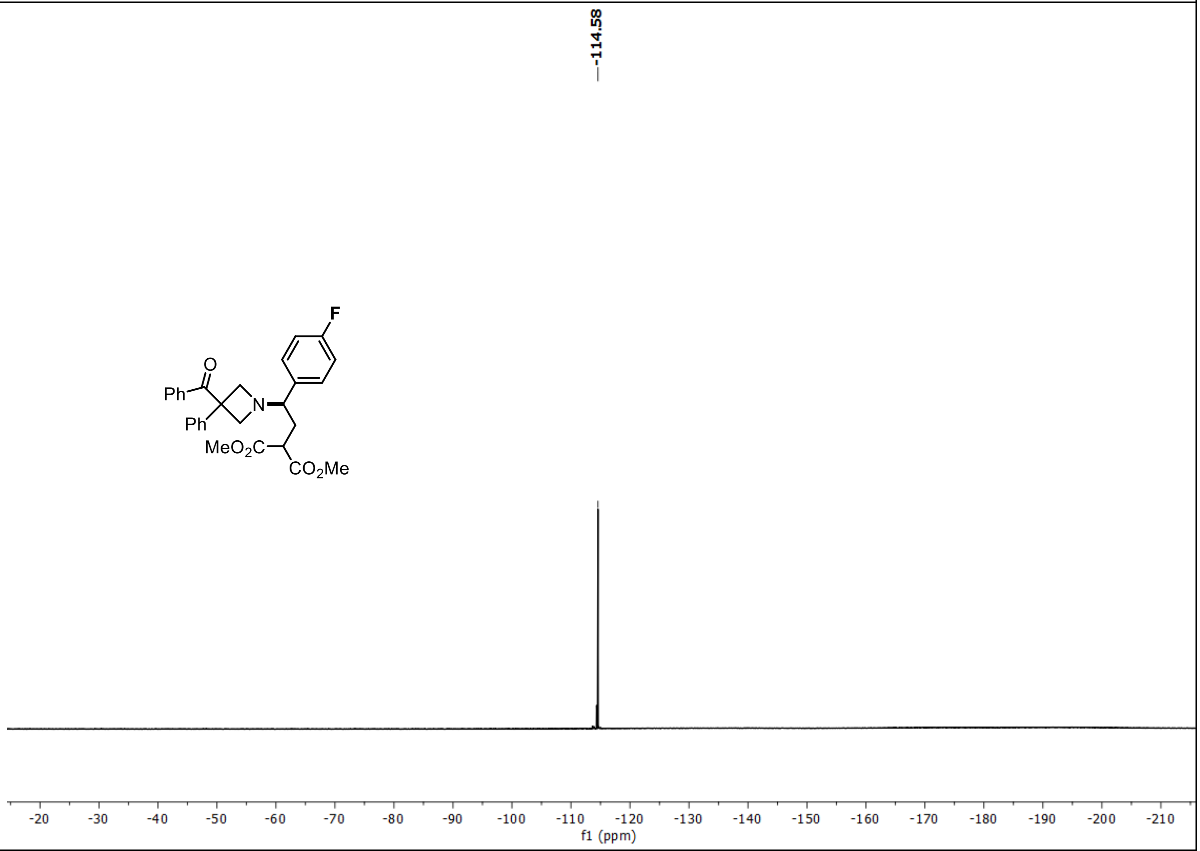

^{1}H spectra at 400 MHz and ^{13}C{^{1}H} NMR spectra at 100 MHz in $CDCl_3$ (**13**)

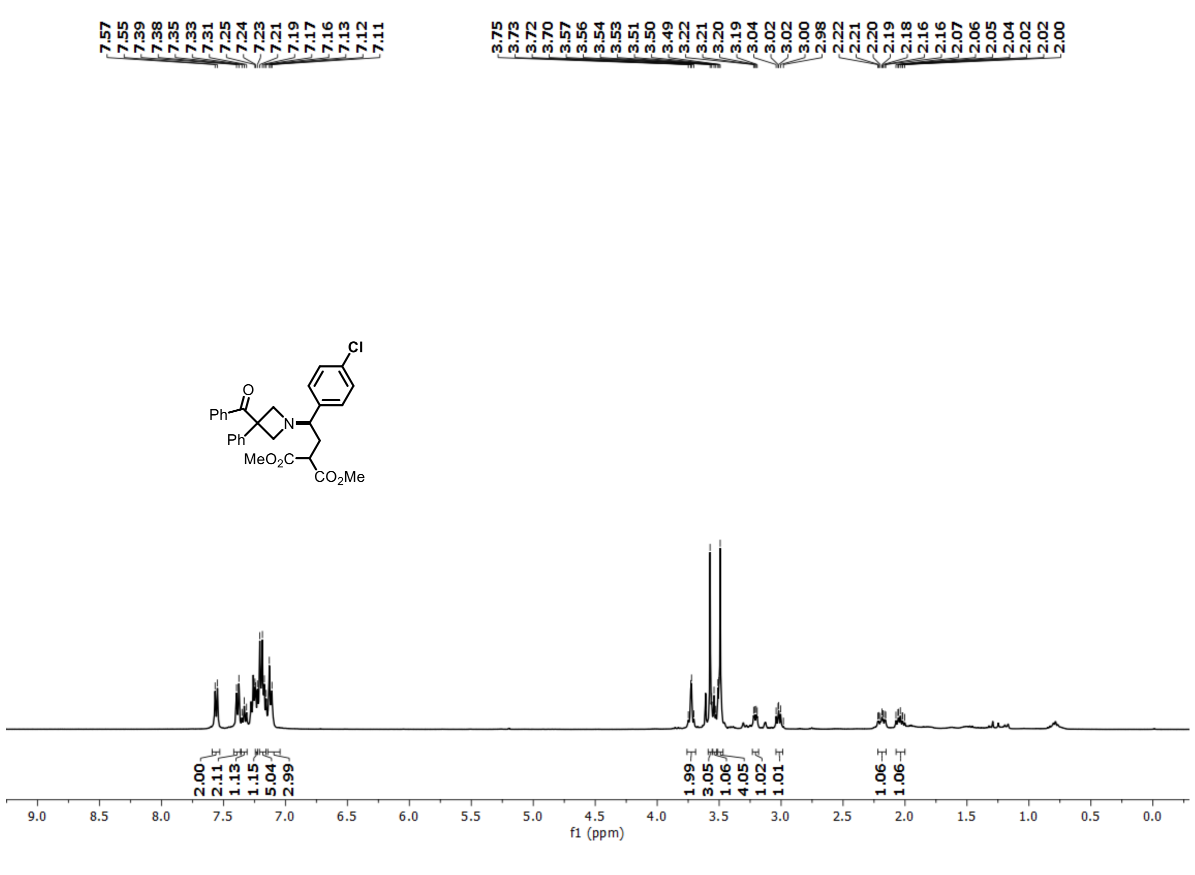

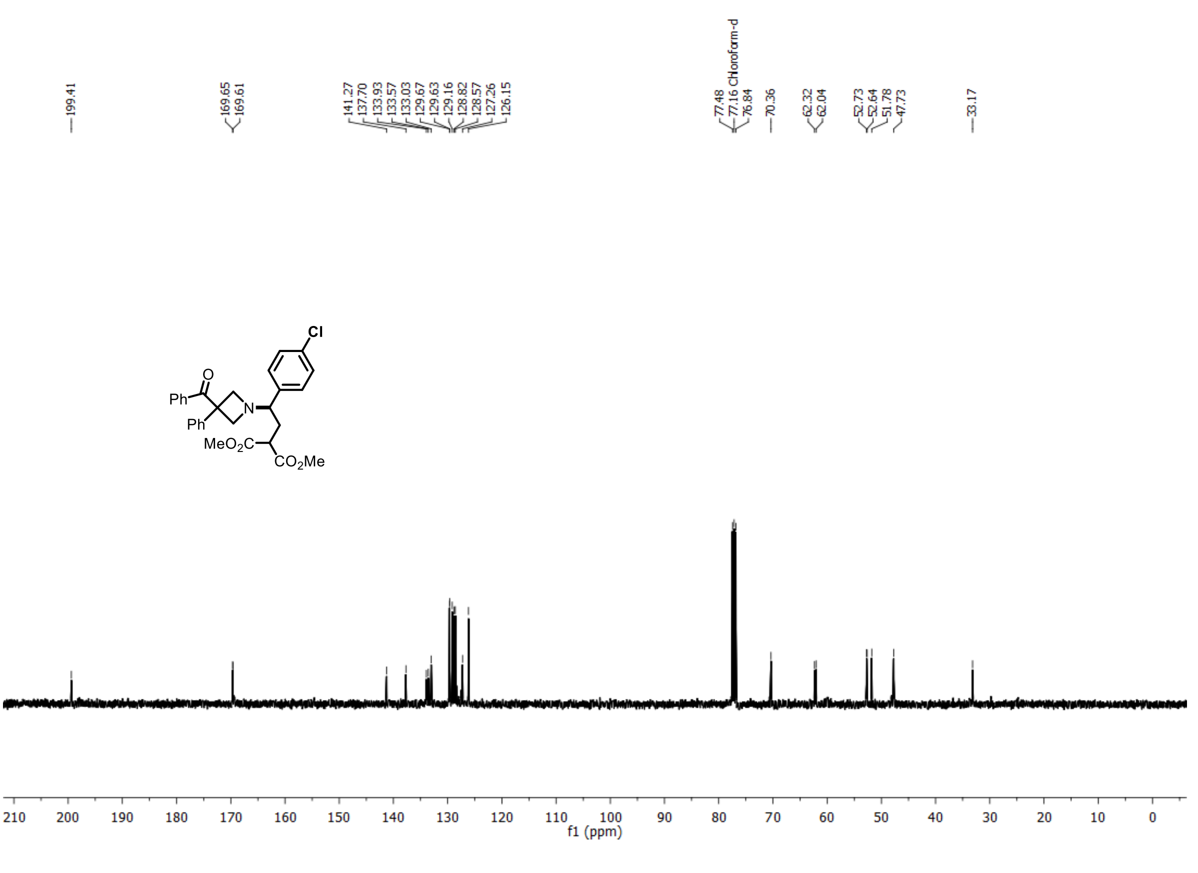

^{1}H spectra at 400 MHz and $^{13}C\{^{1}H\}$ NMR spectra at 100 MHz in $CDCl_3$ (**16**)

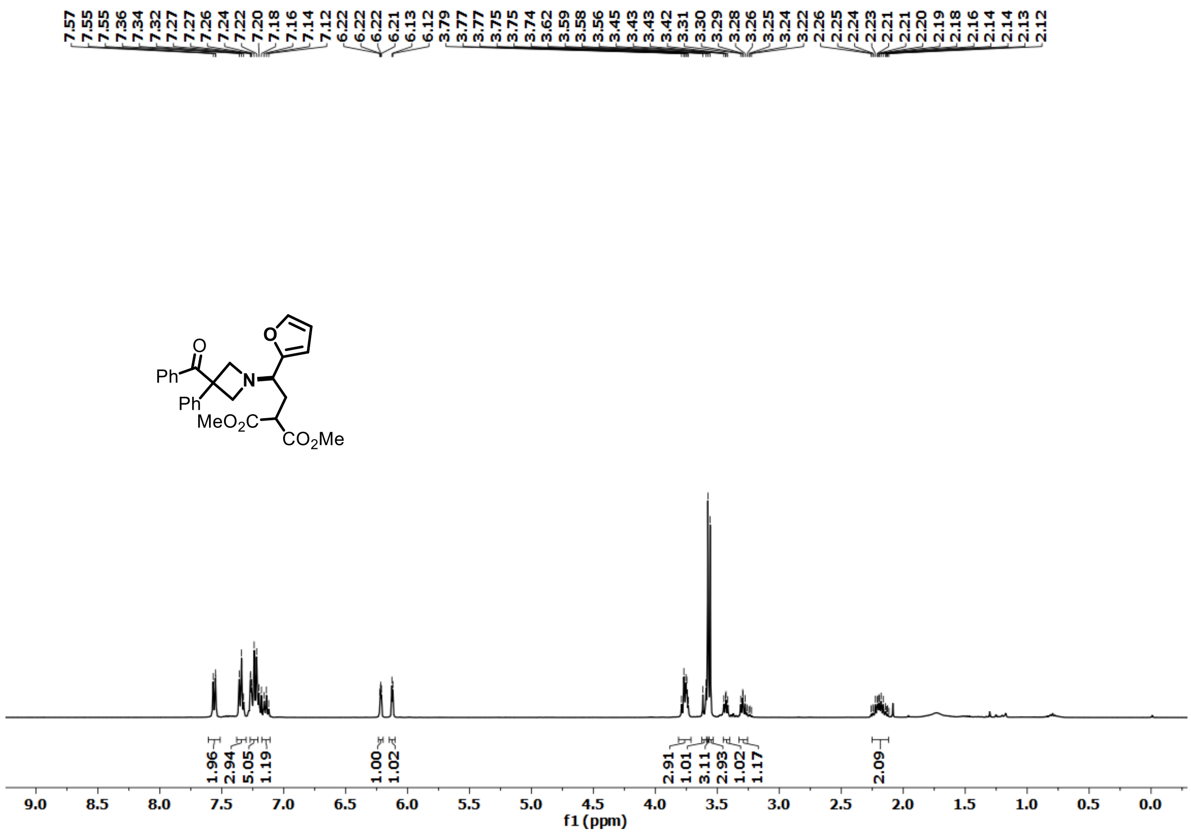

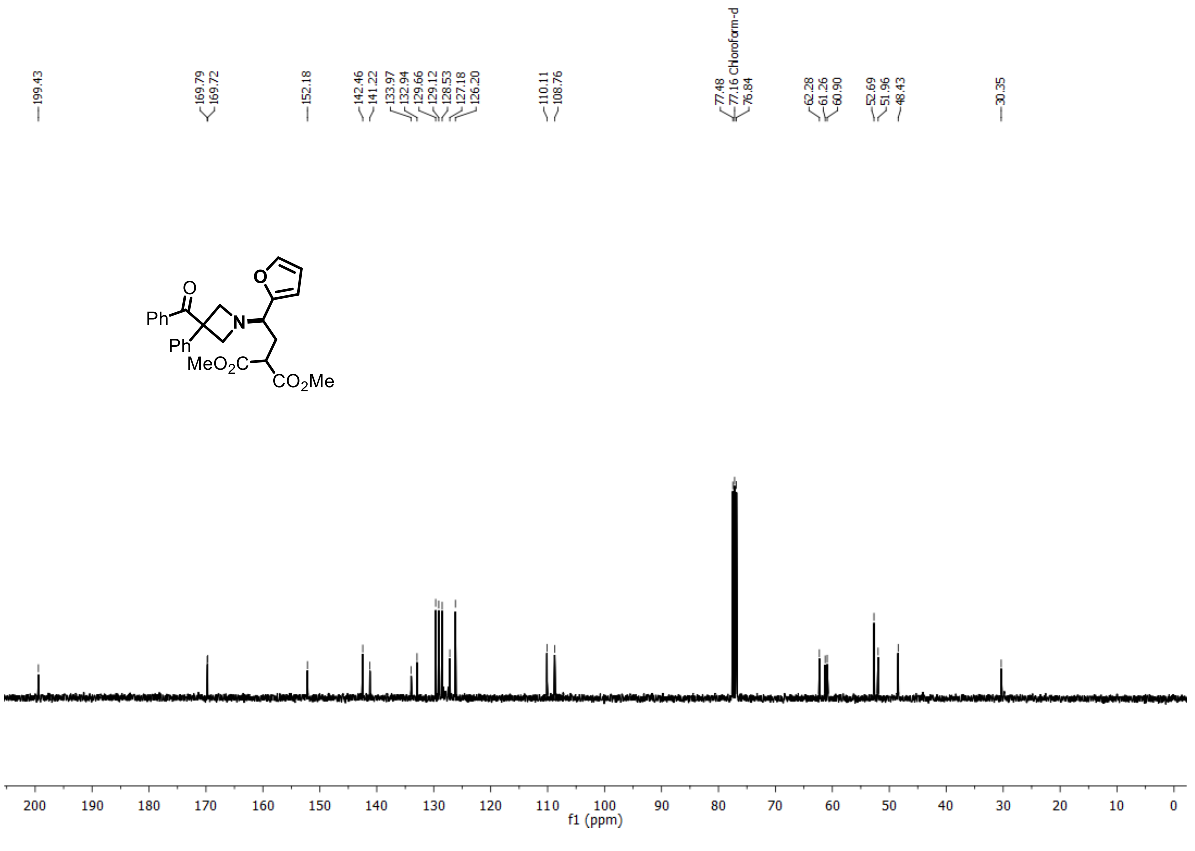

^{1}H spectra at 400 MHz and $^{13}C\{^{1}H\}$ NMR spectra at 100 MHz in DMSO- d^6 (**17**)

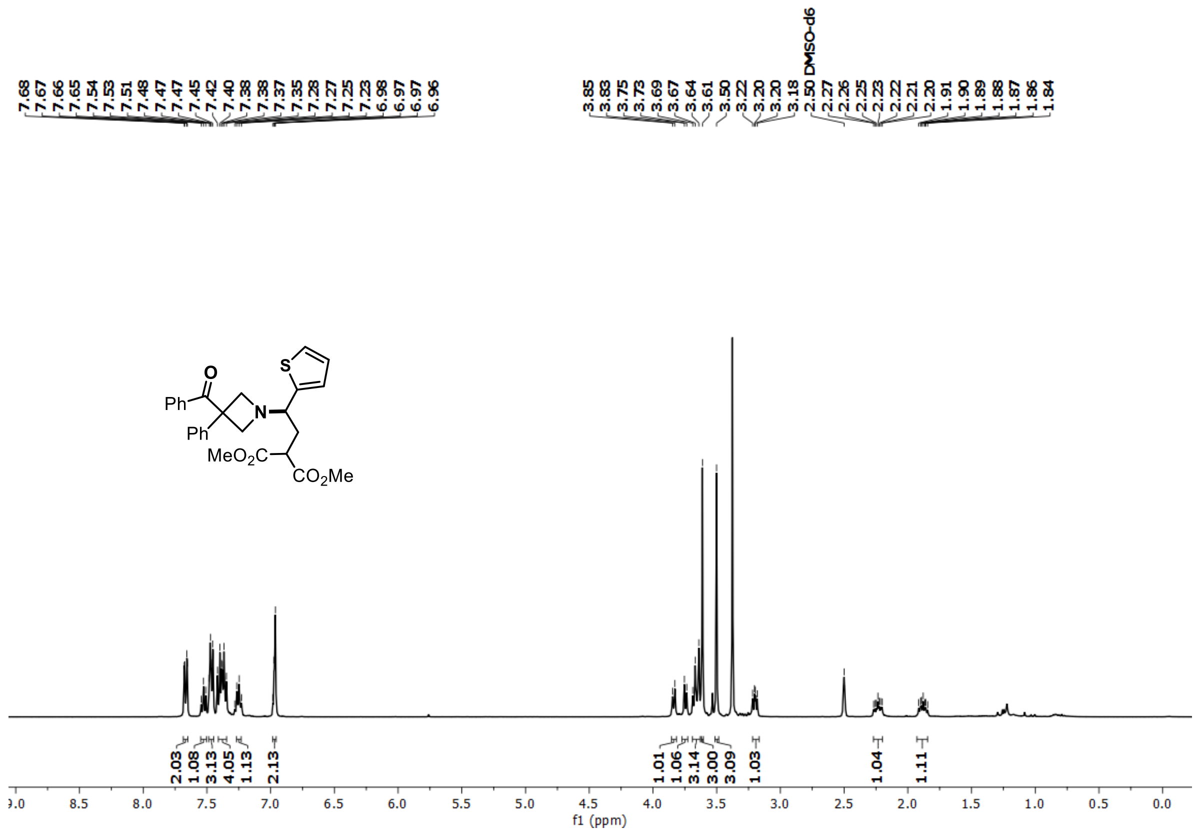

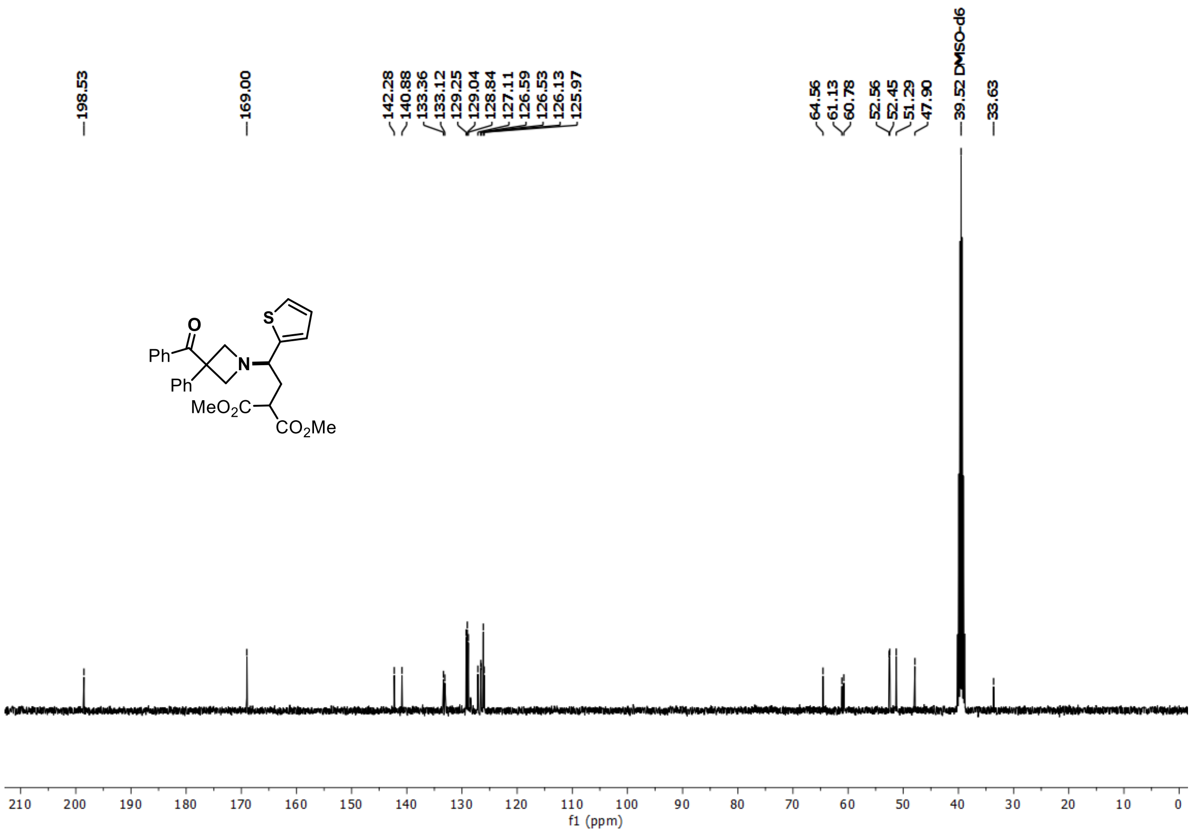

^{1}H spectra at 400 MHz and $^{13}C\{^{1}H\}$ NMR spectra at 100 MHz in $CDCl_3$ (**18**)

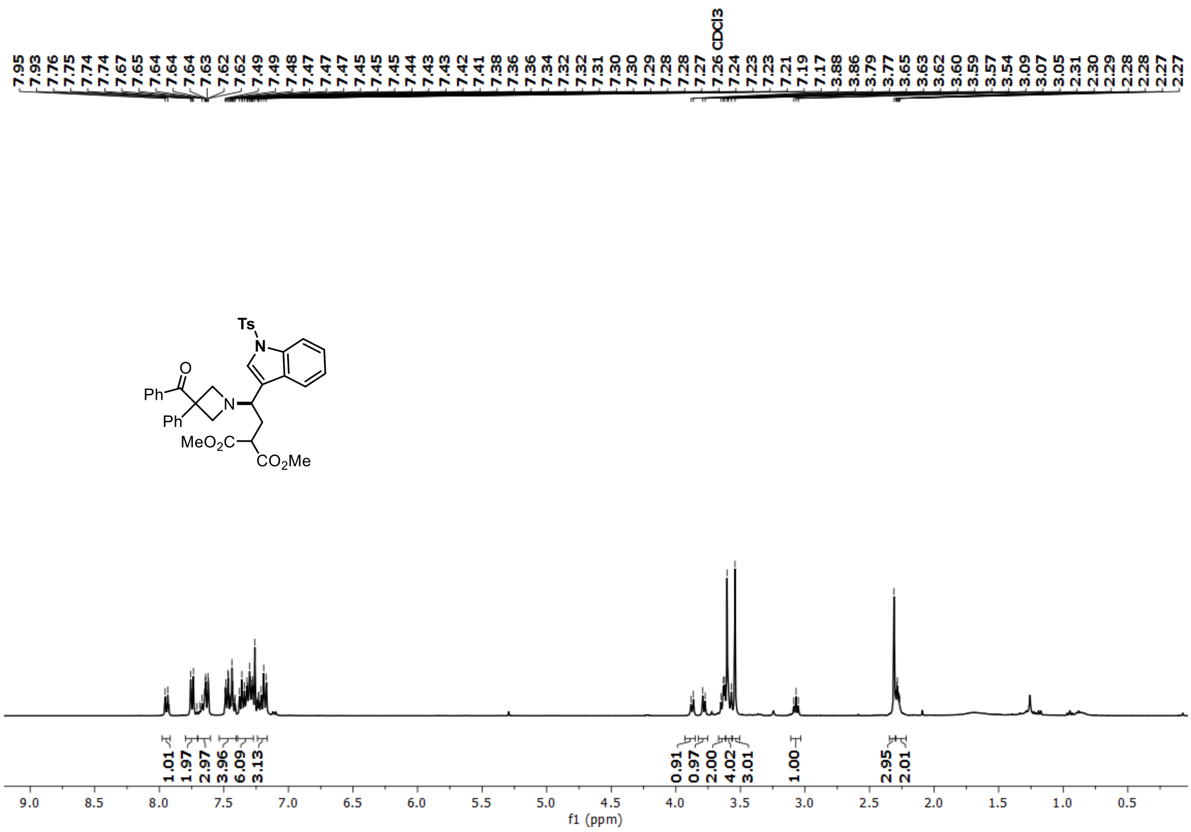

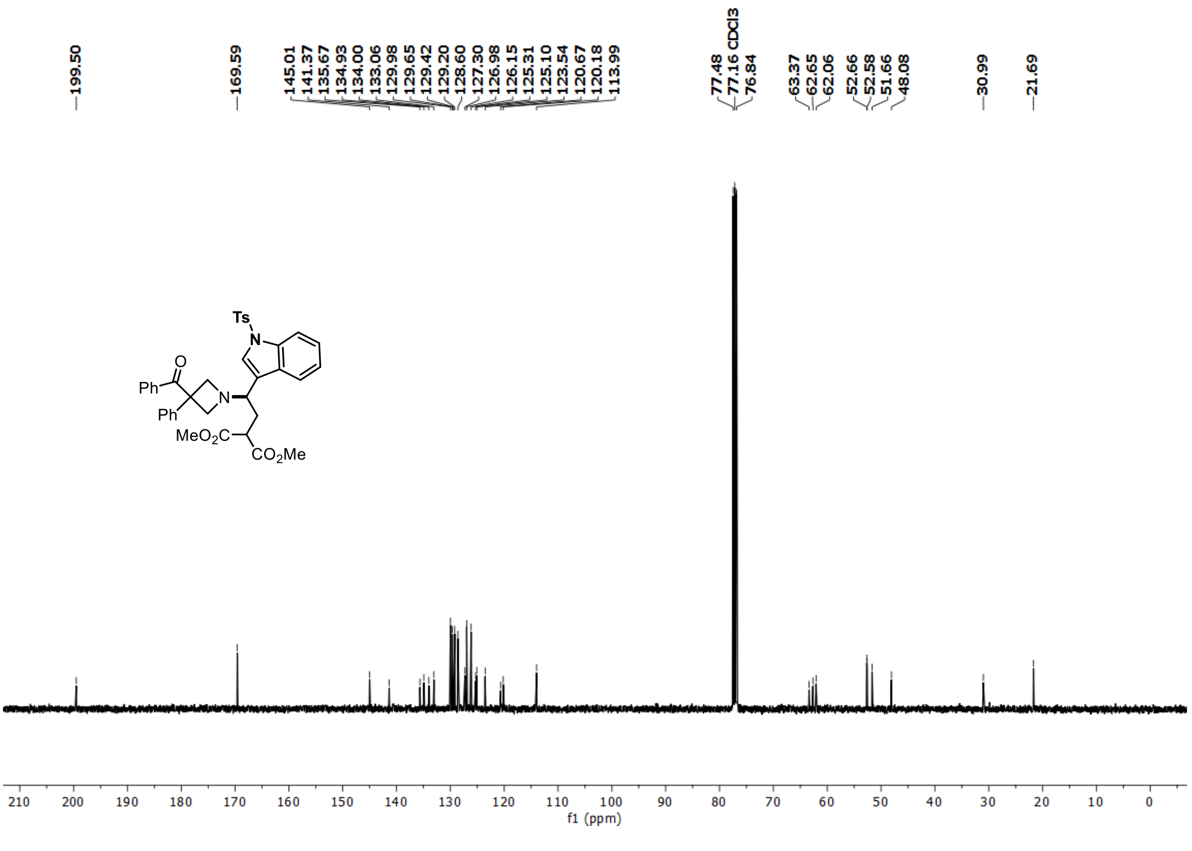

^{1}H spectra at 400 MHz and $^{13}C\{^{1}H\}$ NMR spectra at 100 MHz in $CDCl_3$ (**19**)

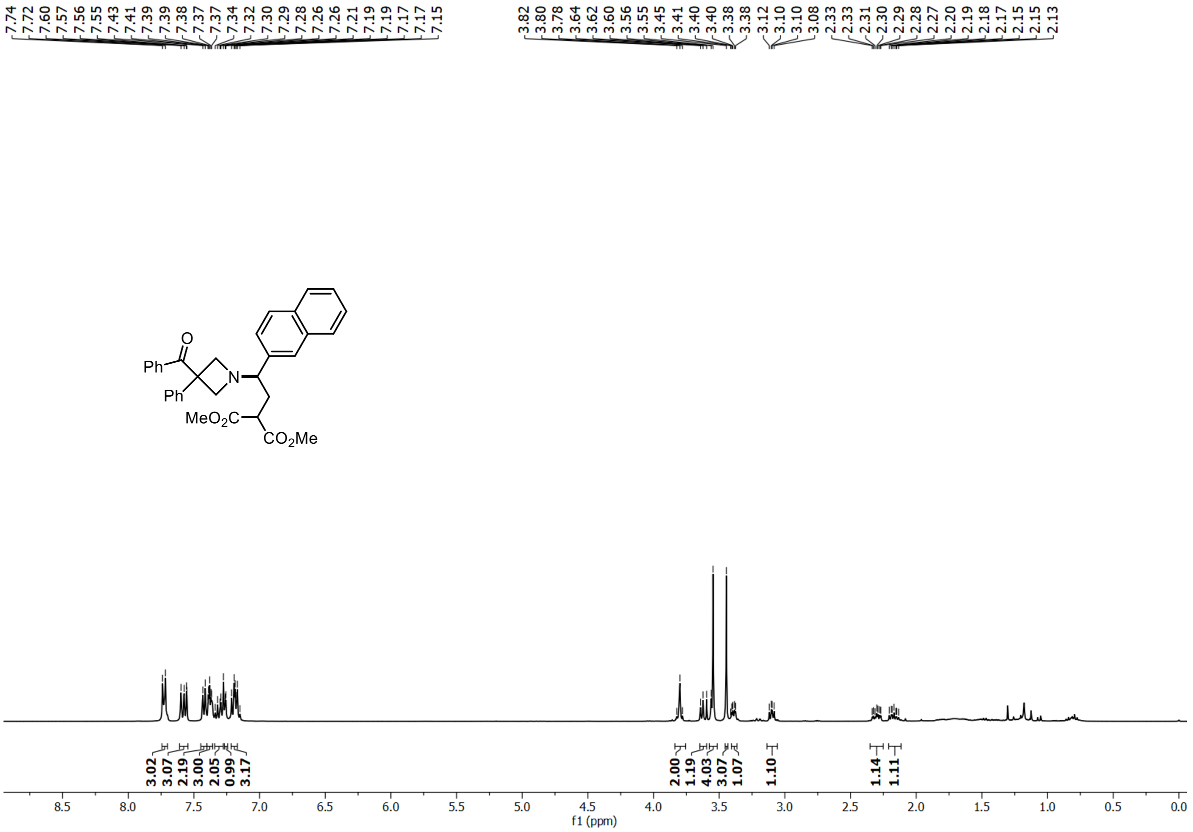

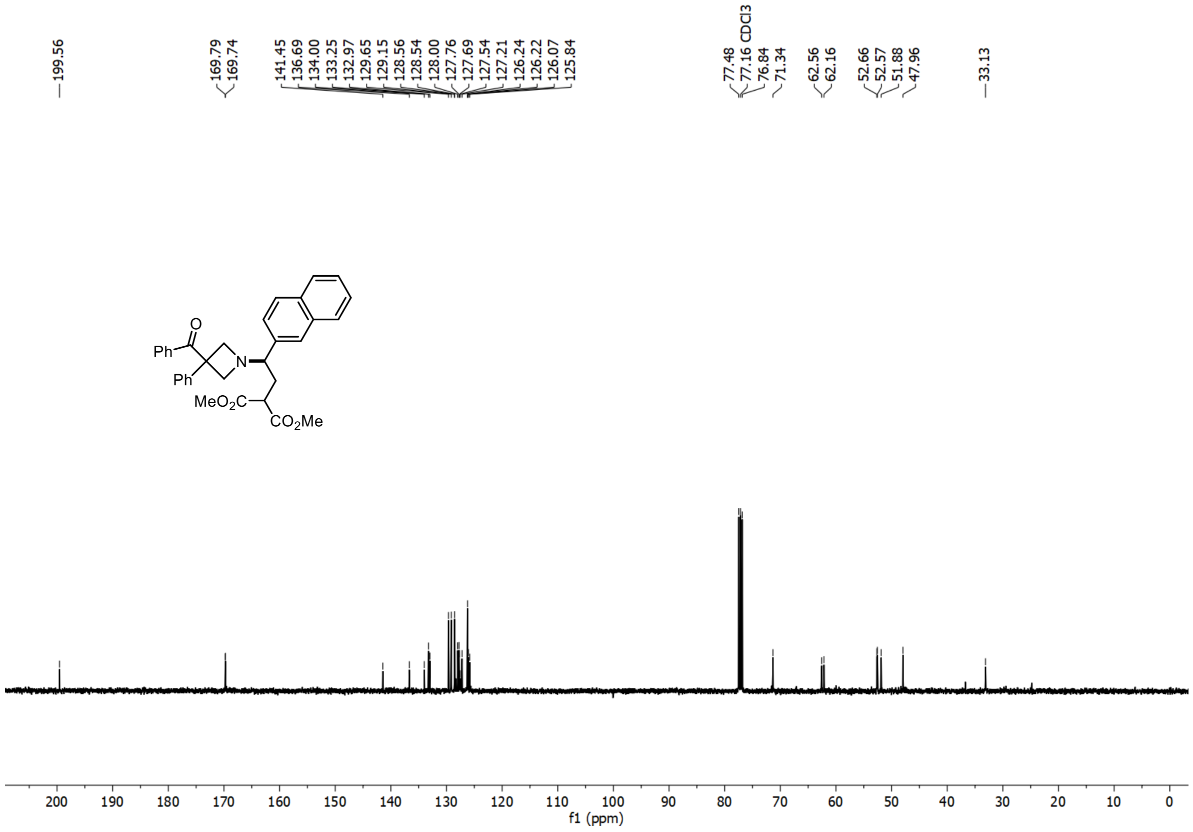

^{1}H spectra at 400 MHz and $^{13}C\{^{1}H\}$ NMR spectra at 100 MHz in DMSO-d6 (**20**)

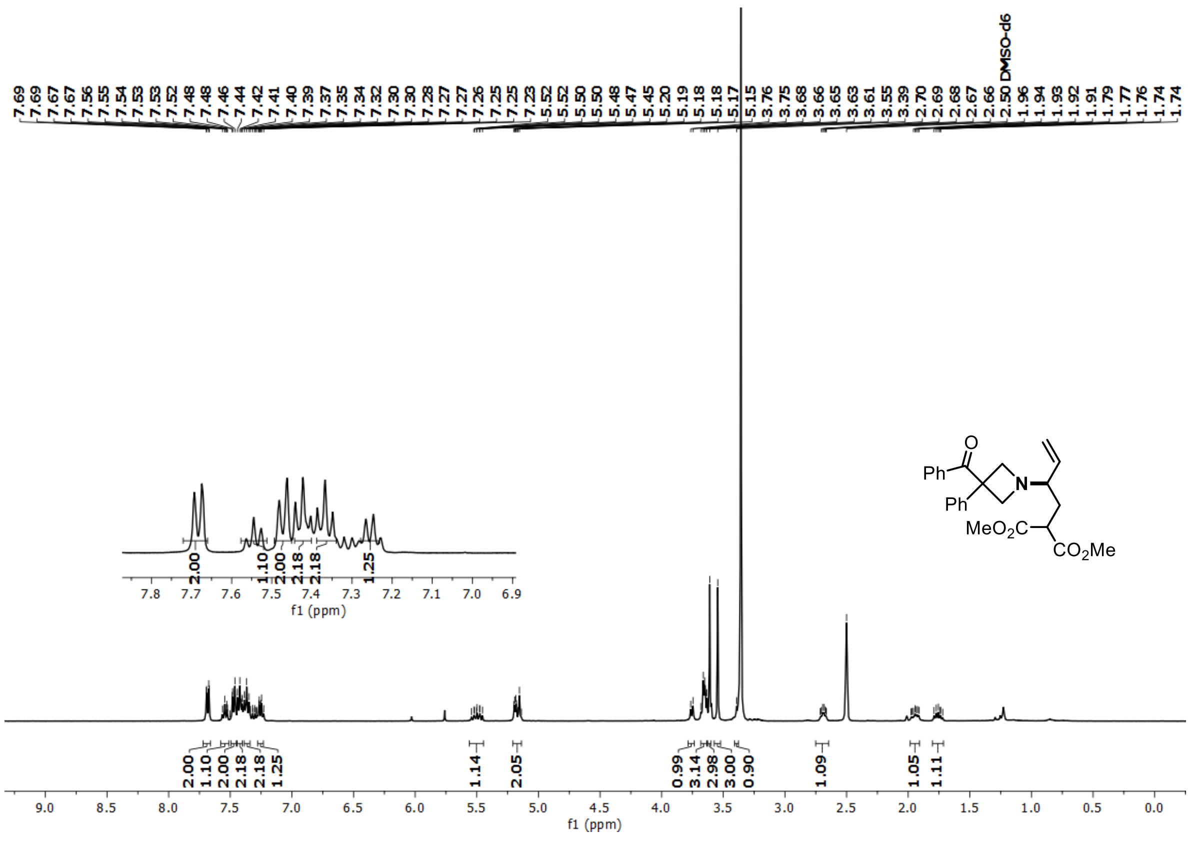

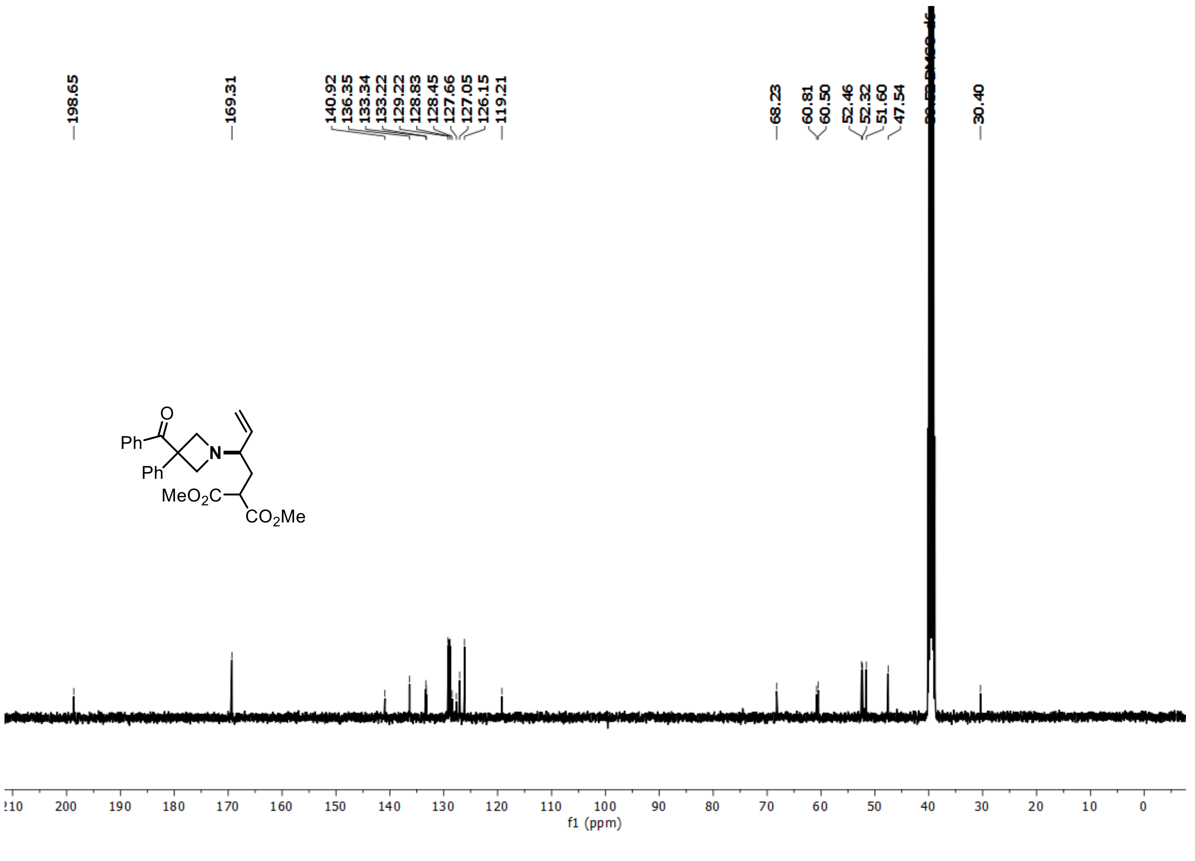

^{1}H spectra at 600 MHz and ^{13}C{^{1}H} NMR spectra at 150 MHz in DMSO-d6 (**21**)

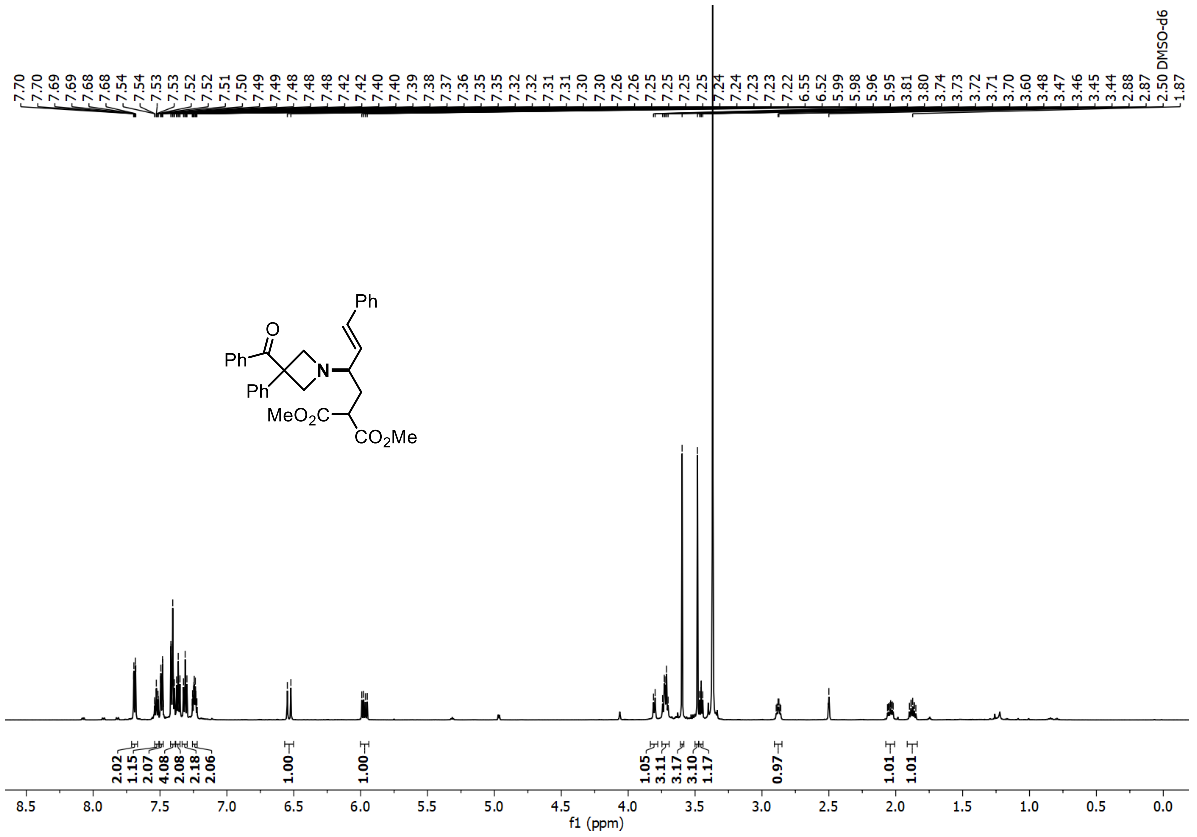

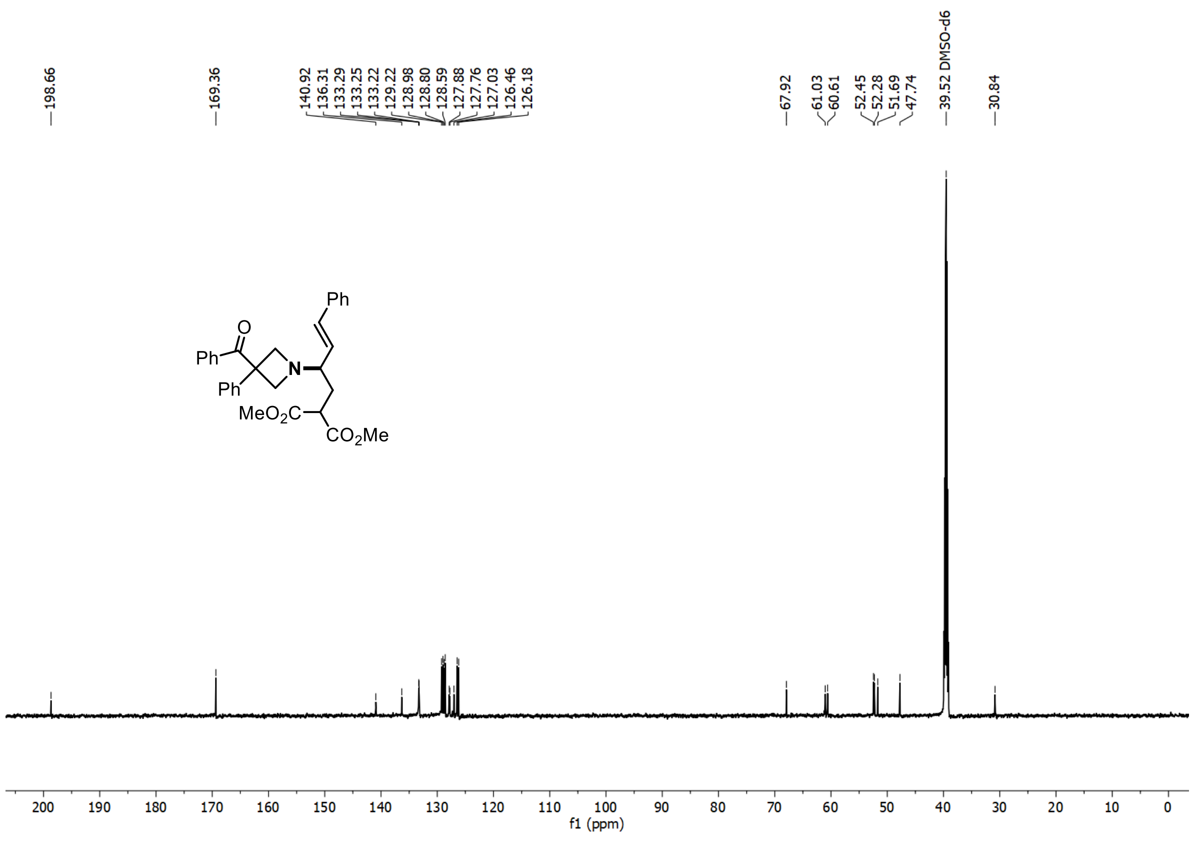

^1H spectra at 600 MHz and $^{13}C\{^1H\}$ NMR spectra at 150 MHz in DMSO-d6 (**22**)

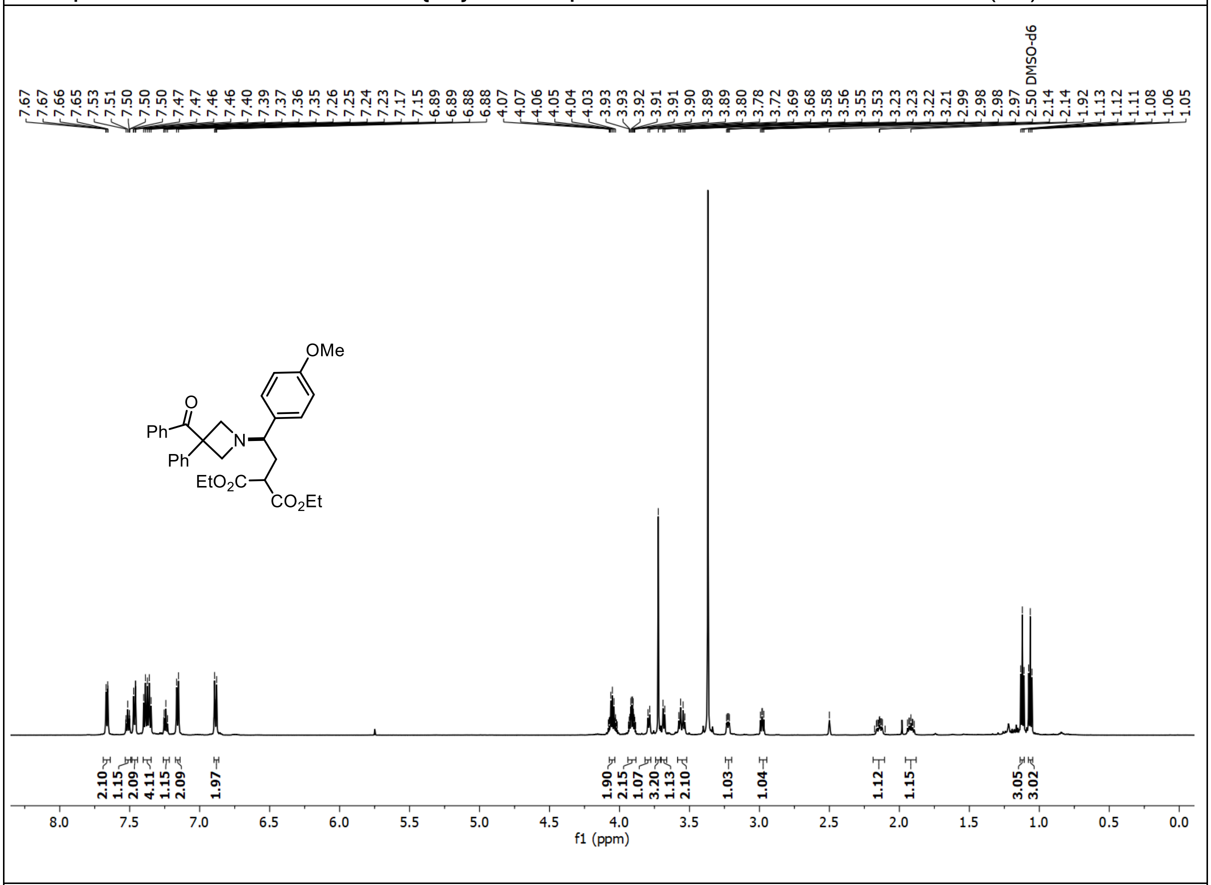

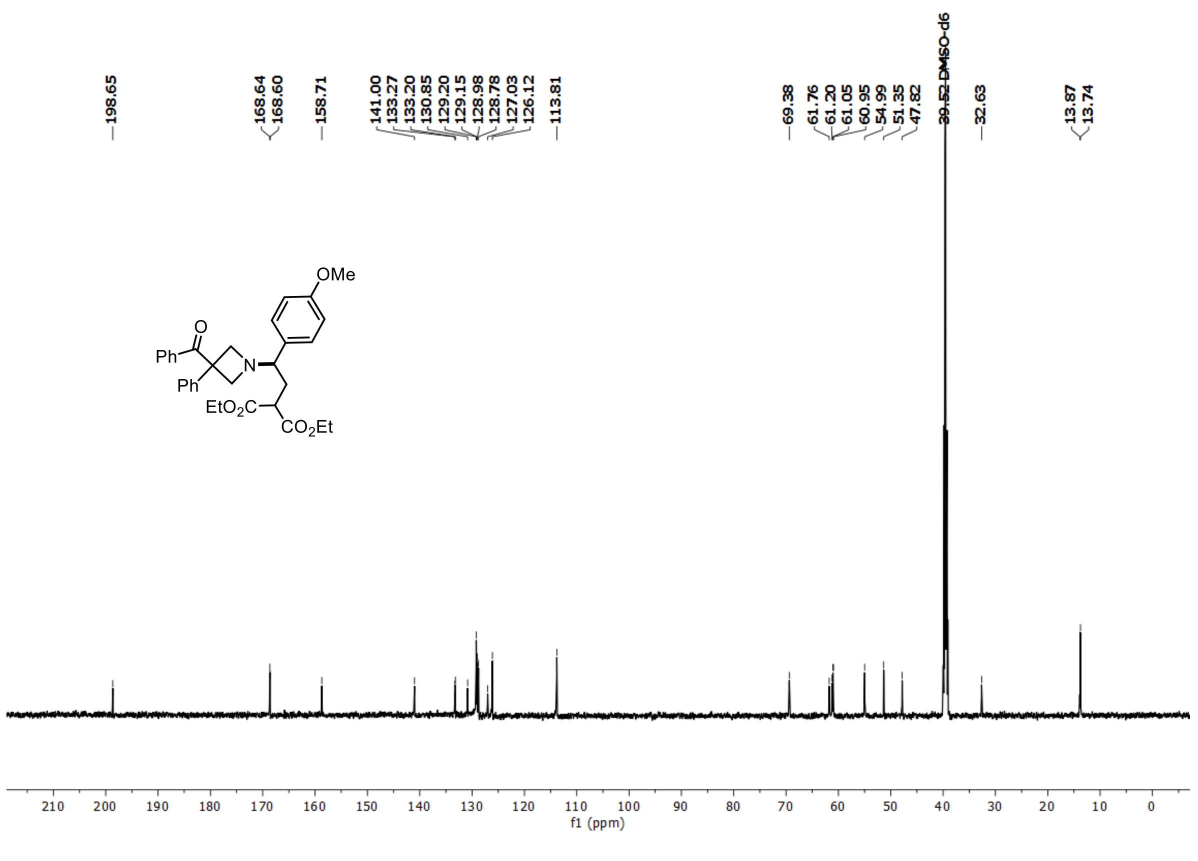

^{1}H spectra at 400 MHz and $^{13}C\{^1H\}$ NMR spectra at 100 MHz in $CDCl_3$ (**23**)

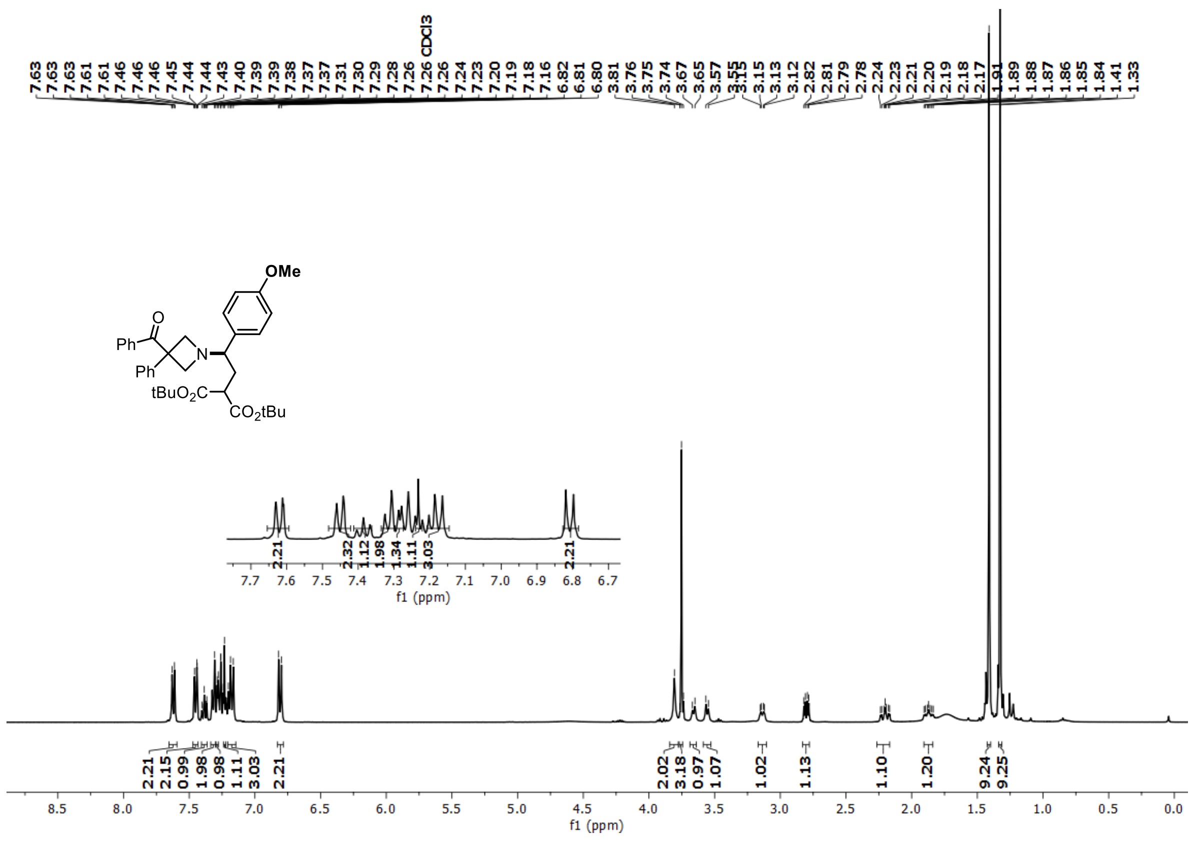

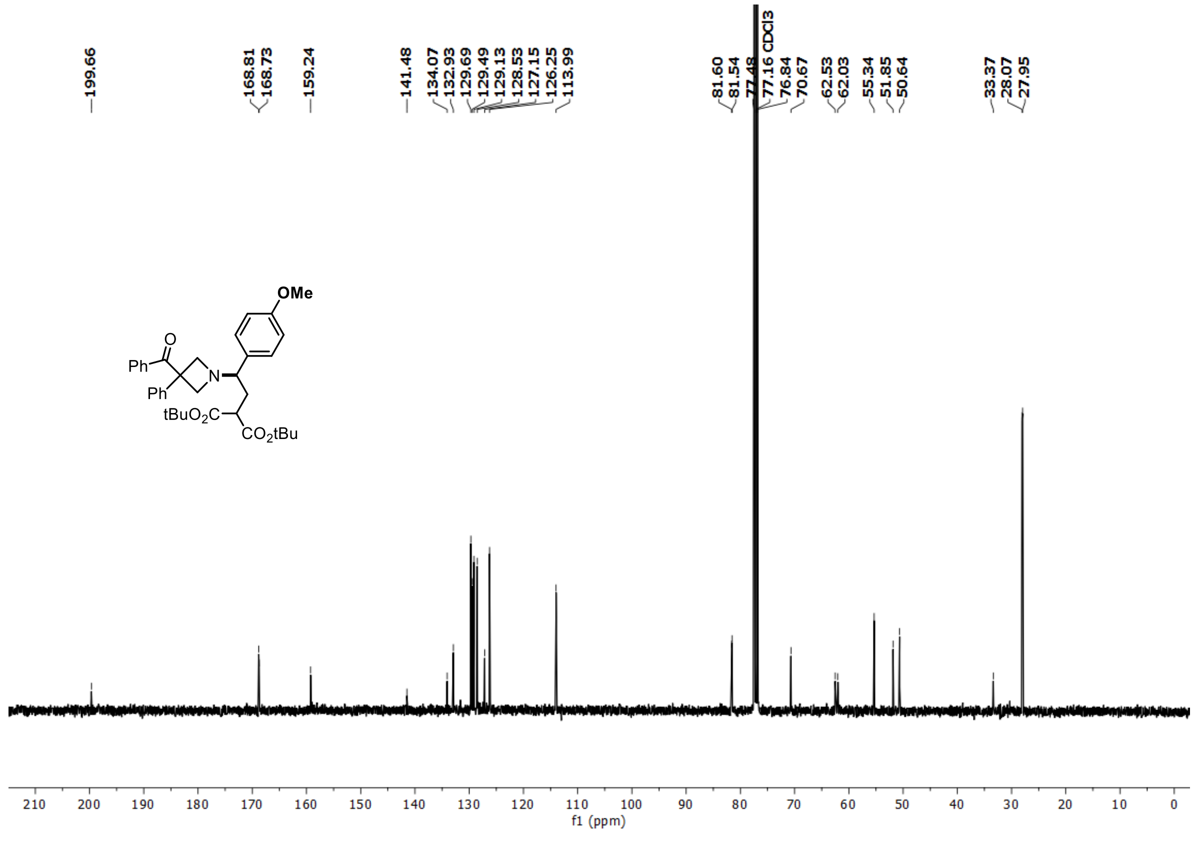

^{1}H spectra at 400 MHz and ^{13}C{^{1}H} NMR spectra at 100 MHz in CDCl3 (**24**)

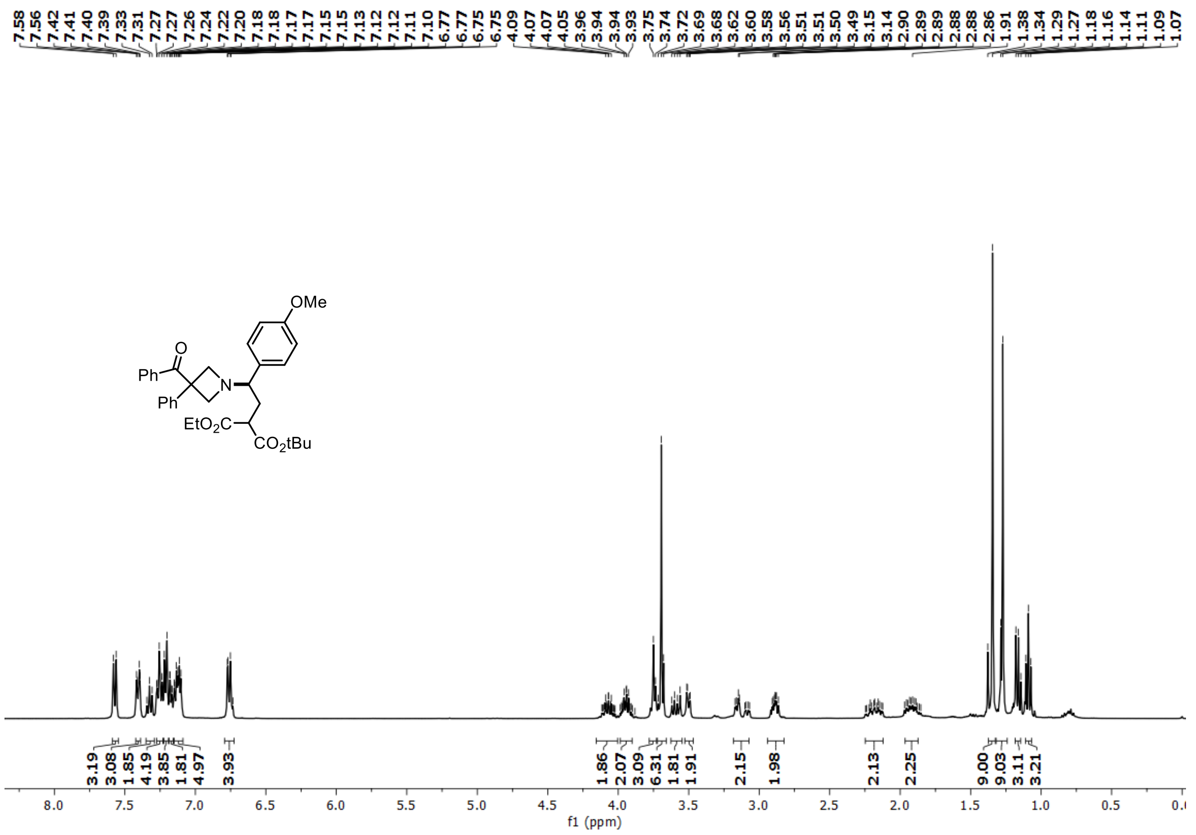

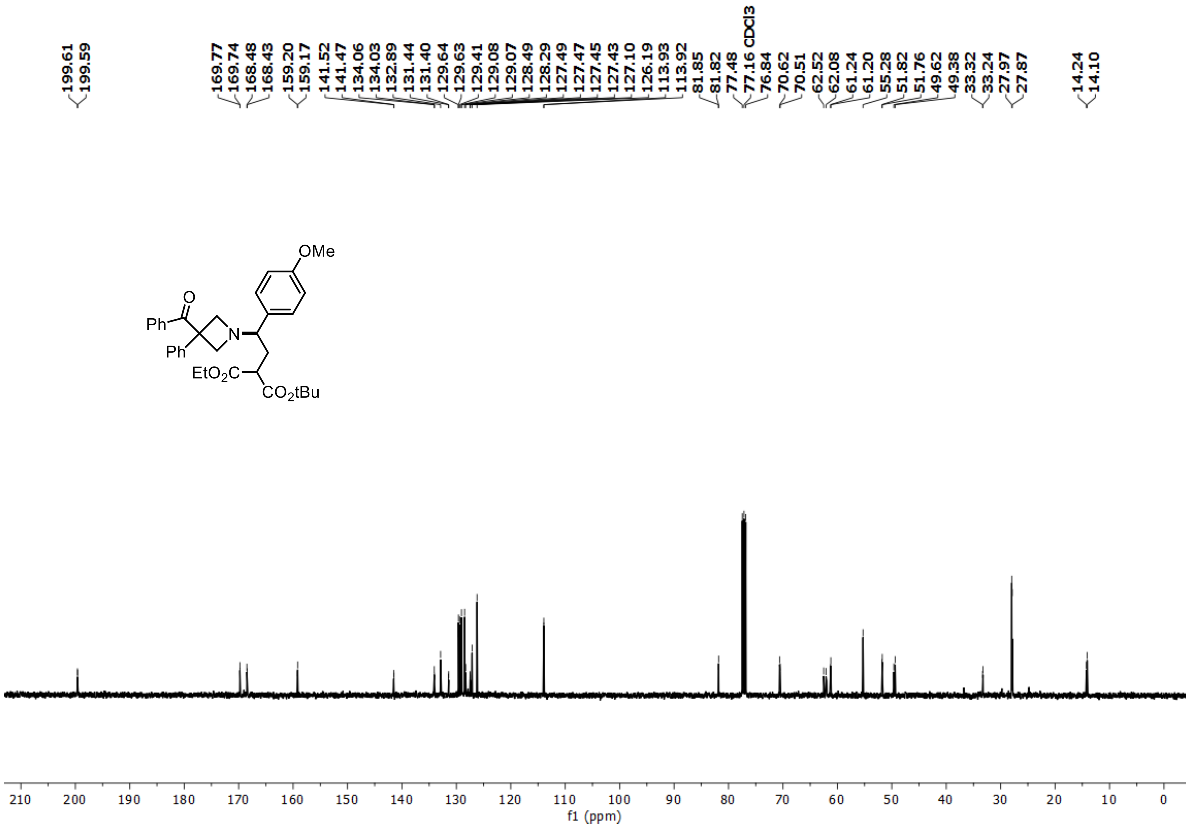

^{1}H spectra at 400 MHz and ^{13}C{^{1}H} NMR spectra at 100 MHz in DMSO-d6 (**25**)

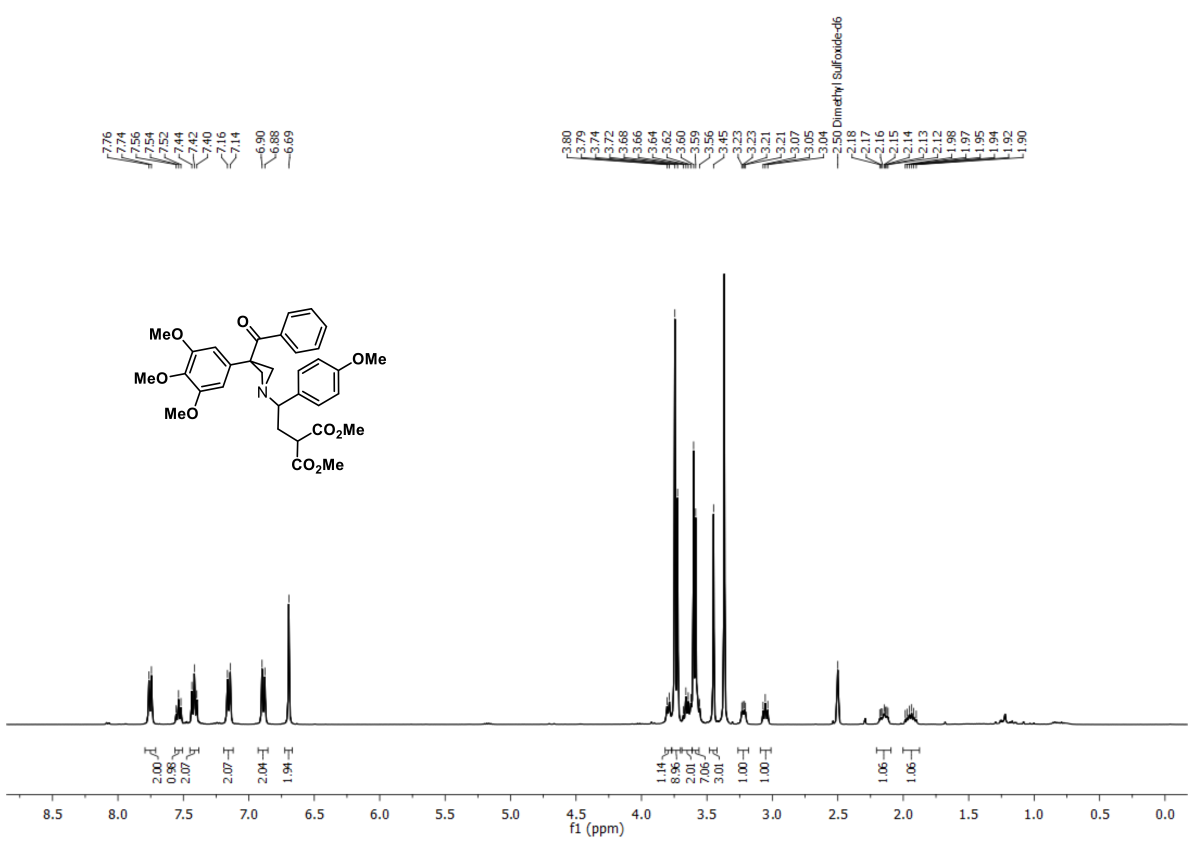

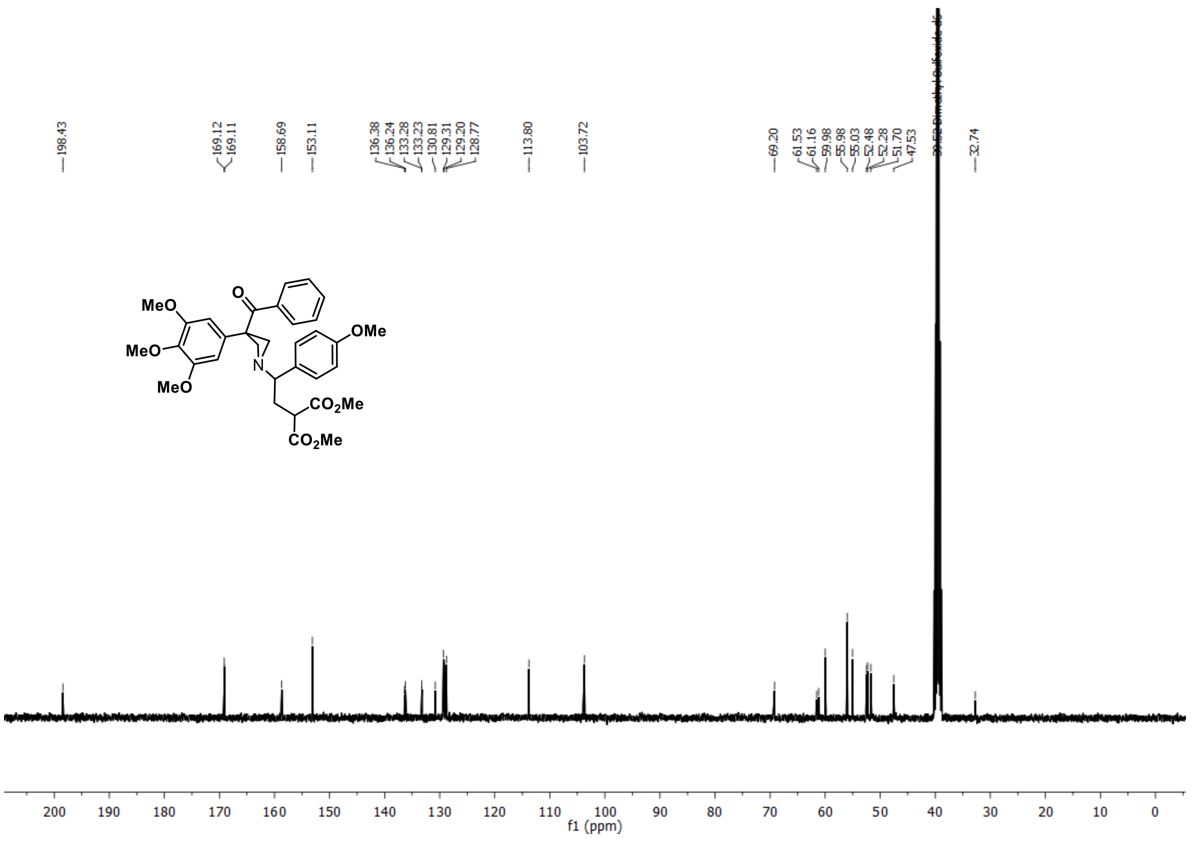

1H spectra at 400 MHz and 13C{^{1}H} NMR spectra at 100 MHz in DMSO- d6 (**26**)

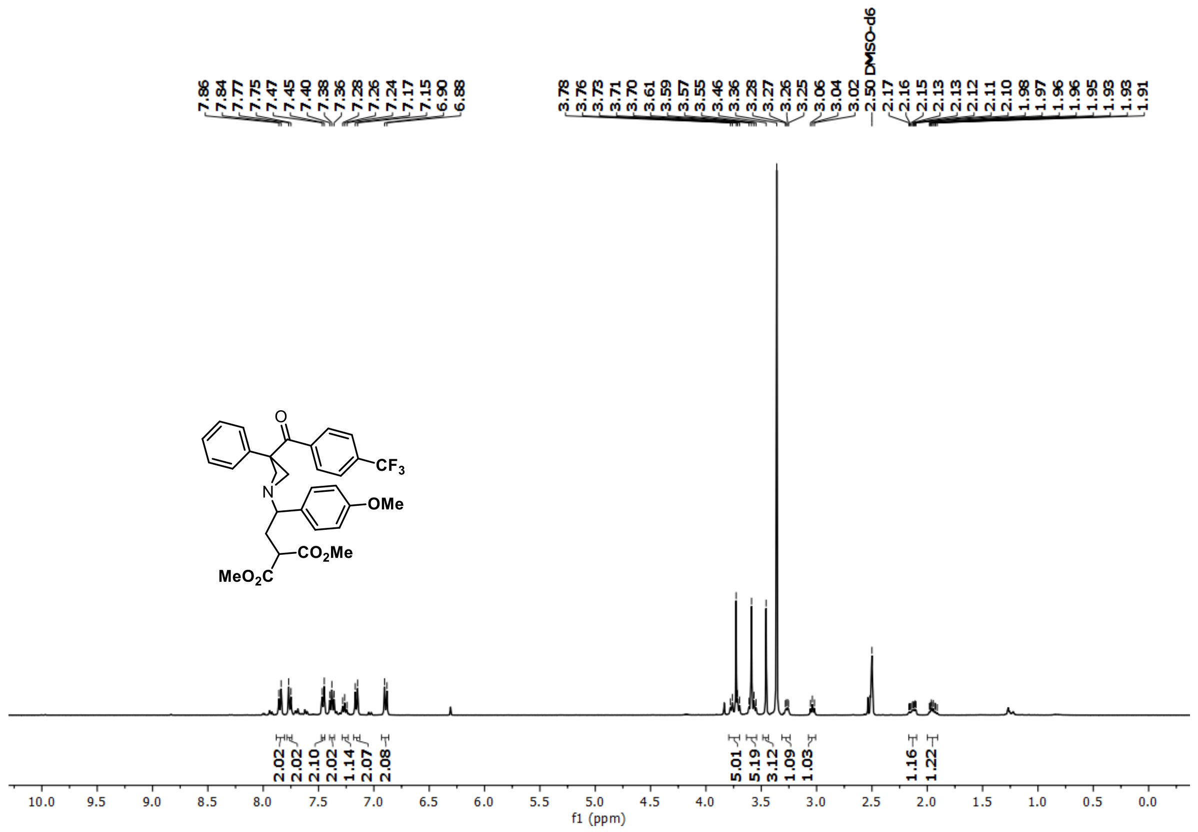

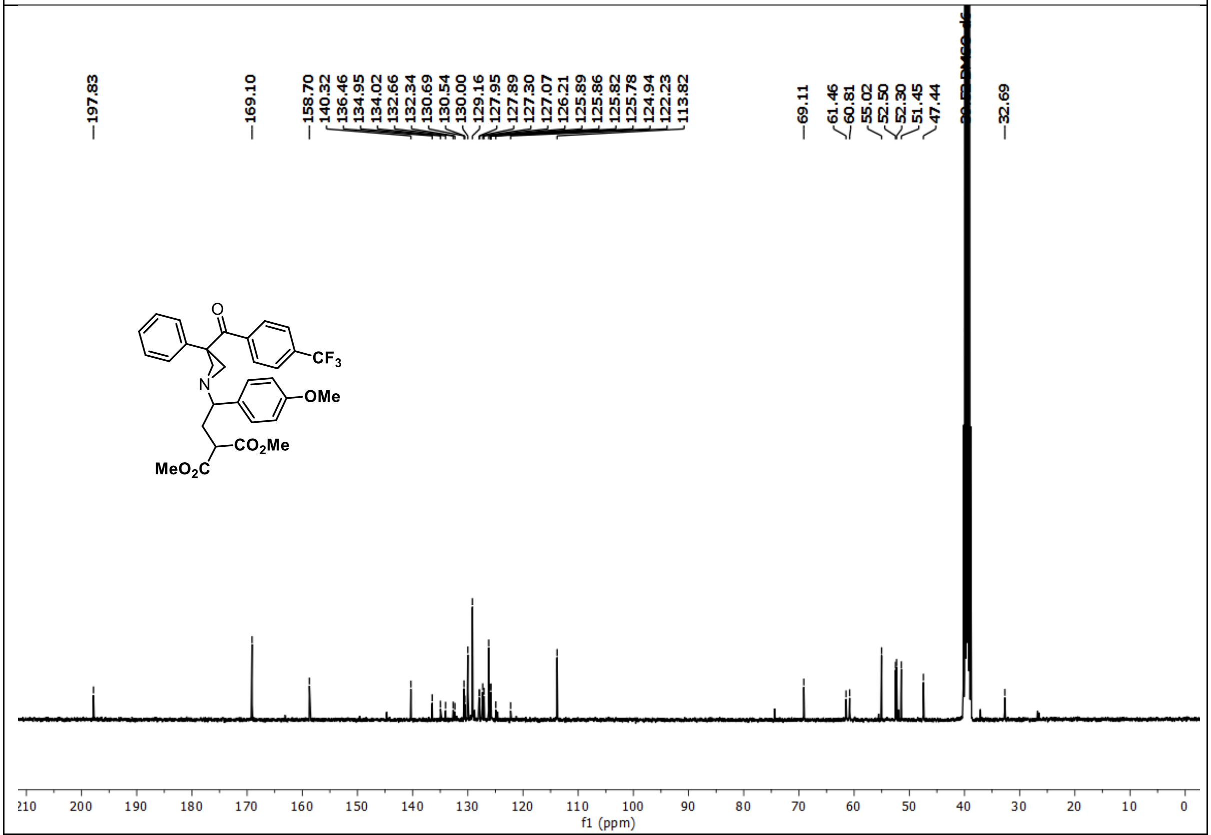

^{1}H spectra at 400 MHz and ^{13}C{^{1}H} NMR spectra at 100 MHz in DMSO-d6 (**27**)

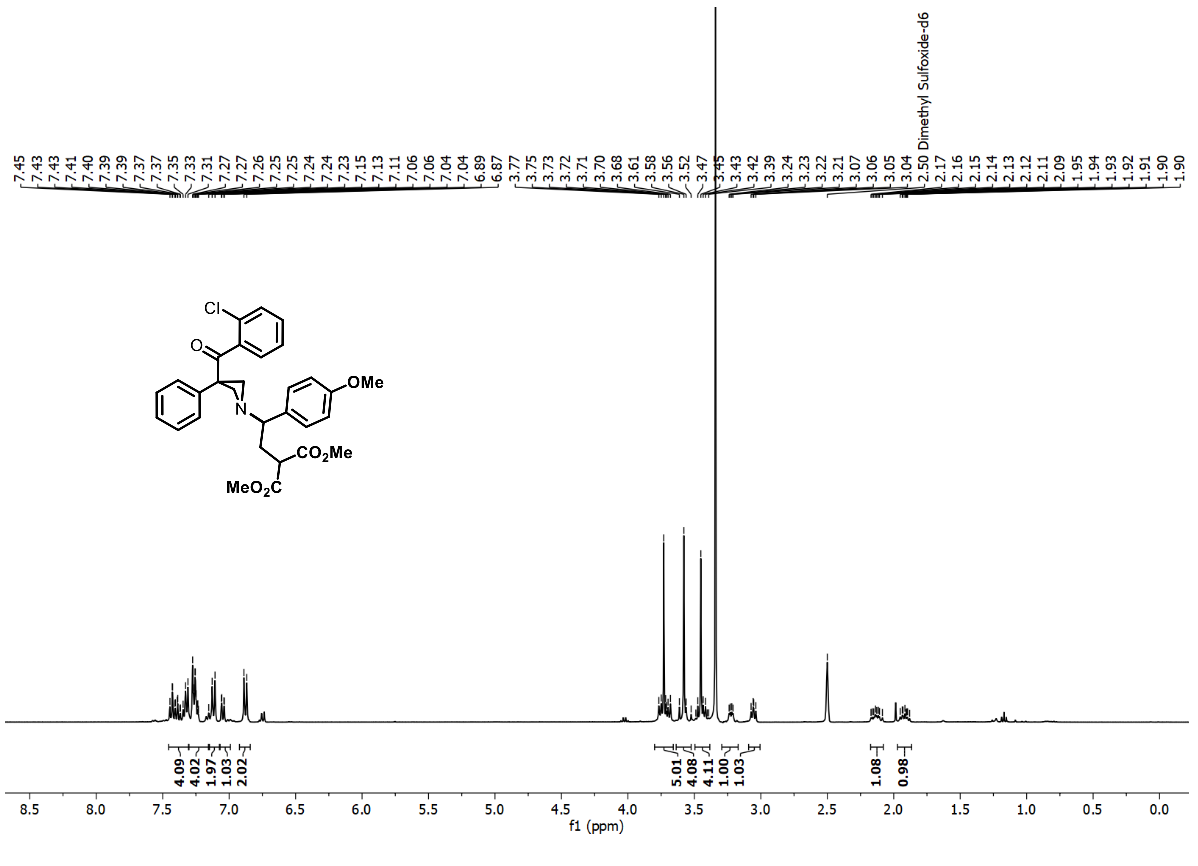

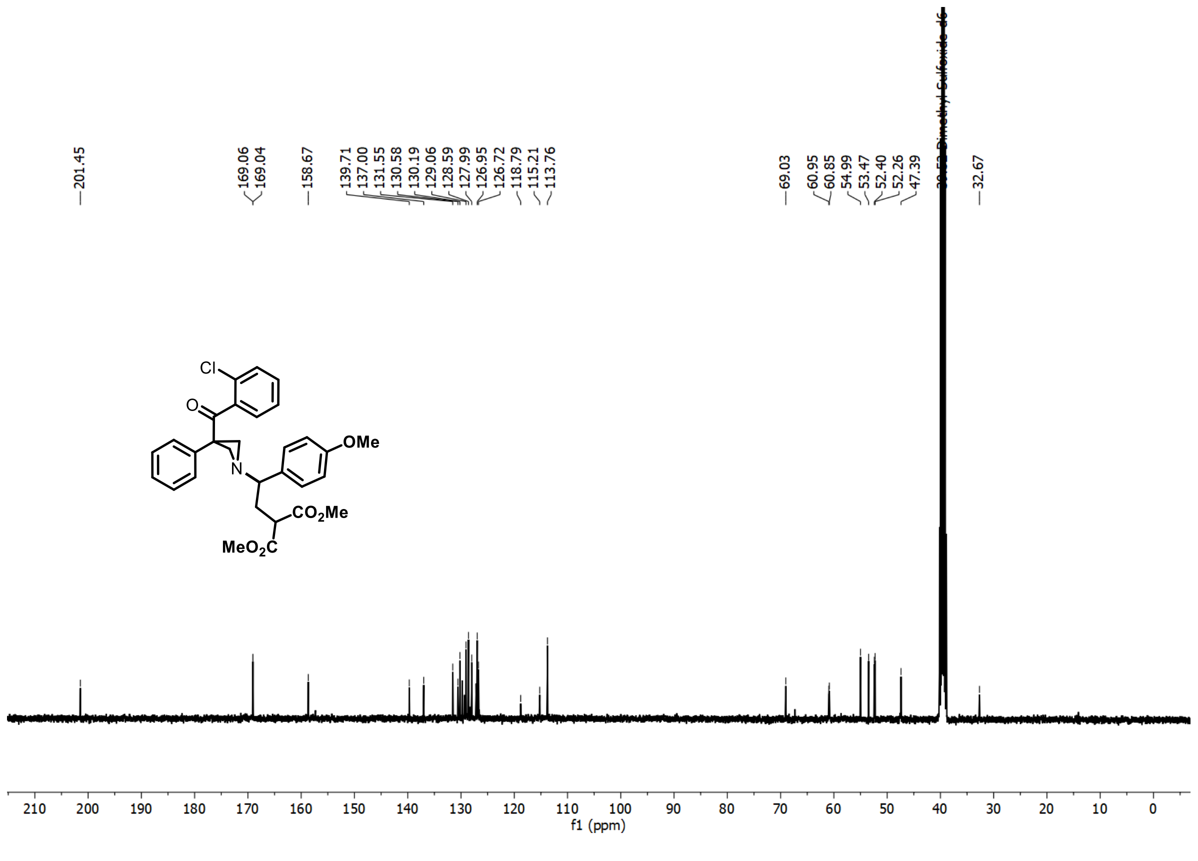

^{1}H spectra at 400 MHz and $^{13}C\{^{1}H\}$ NMR spectra at 100 MHz in DMSO-d6 (**28**)

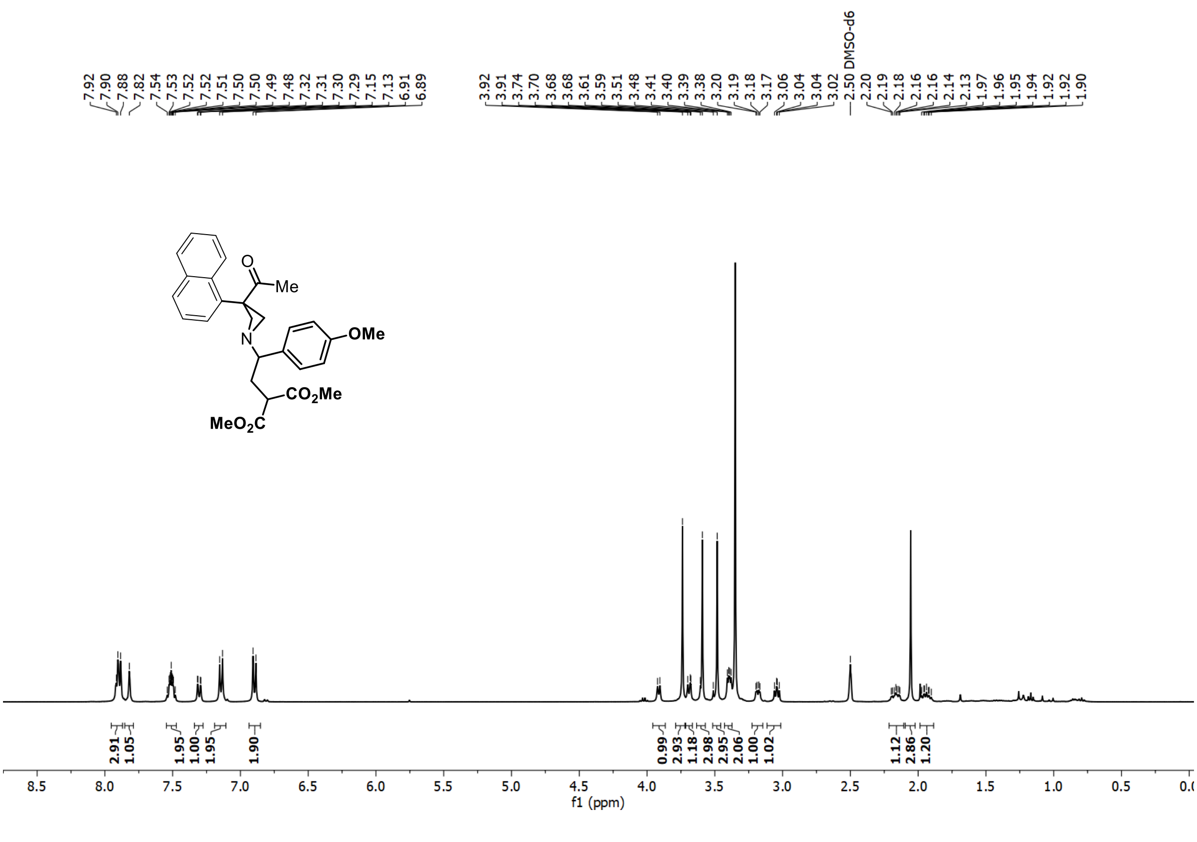

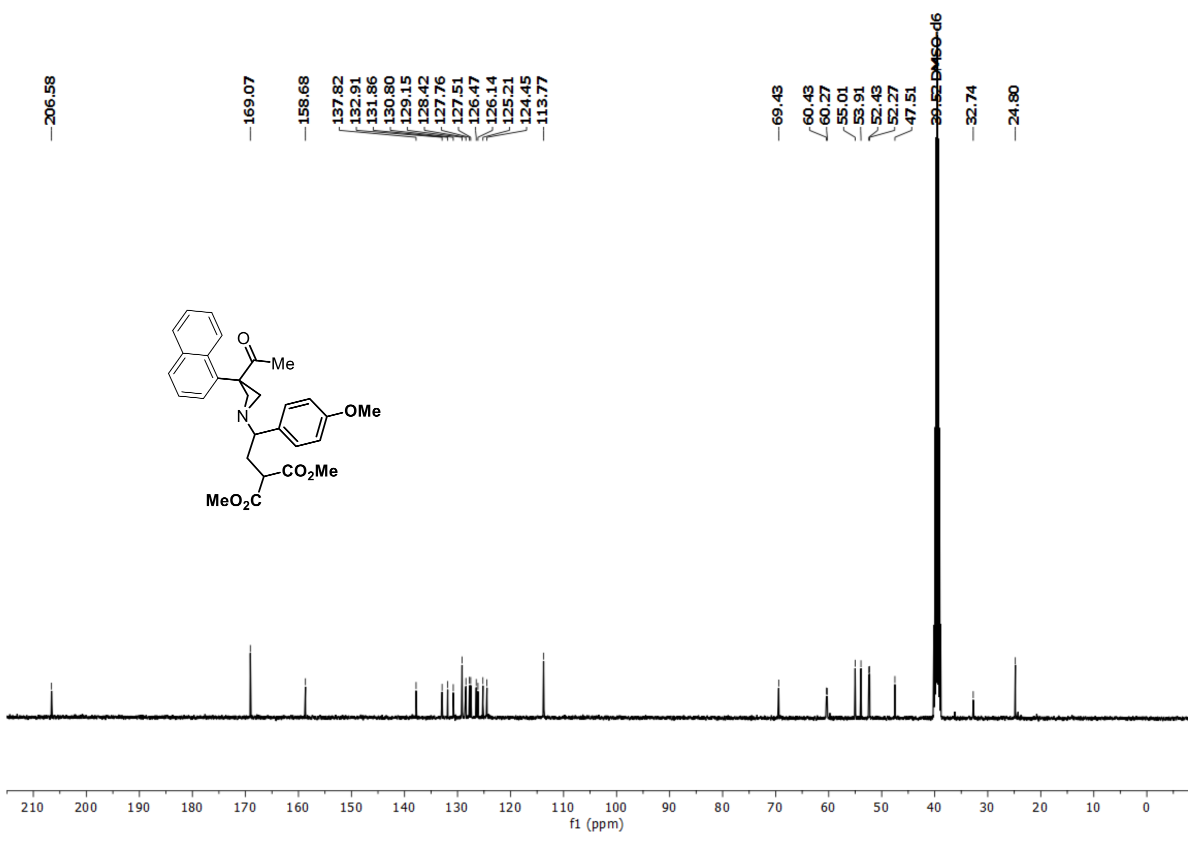

^{1}H spectra at 400 MHz and ^{13}C{^{1}H} NMR spectra at 100 MHz in CDCl3 (**29**)

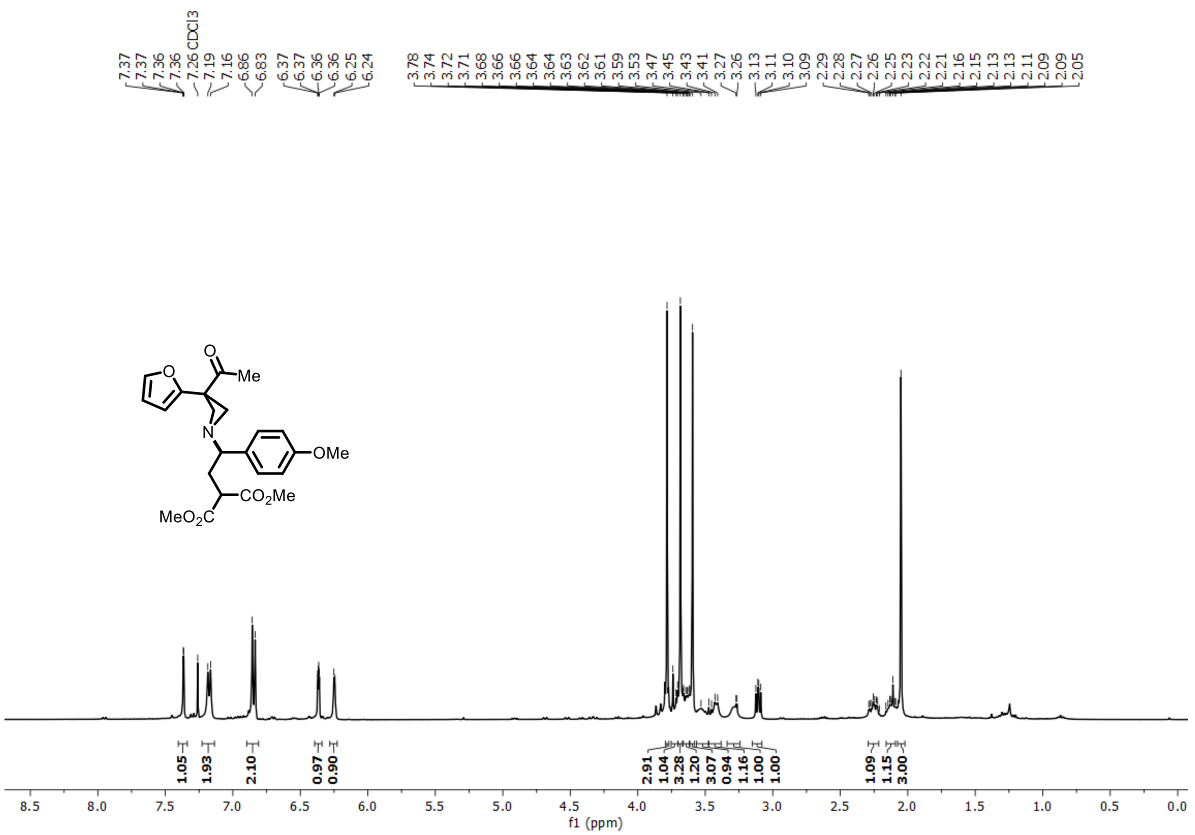

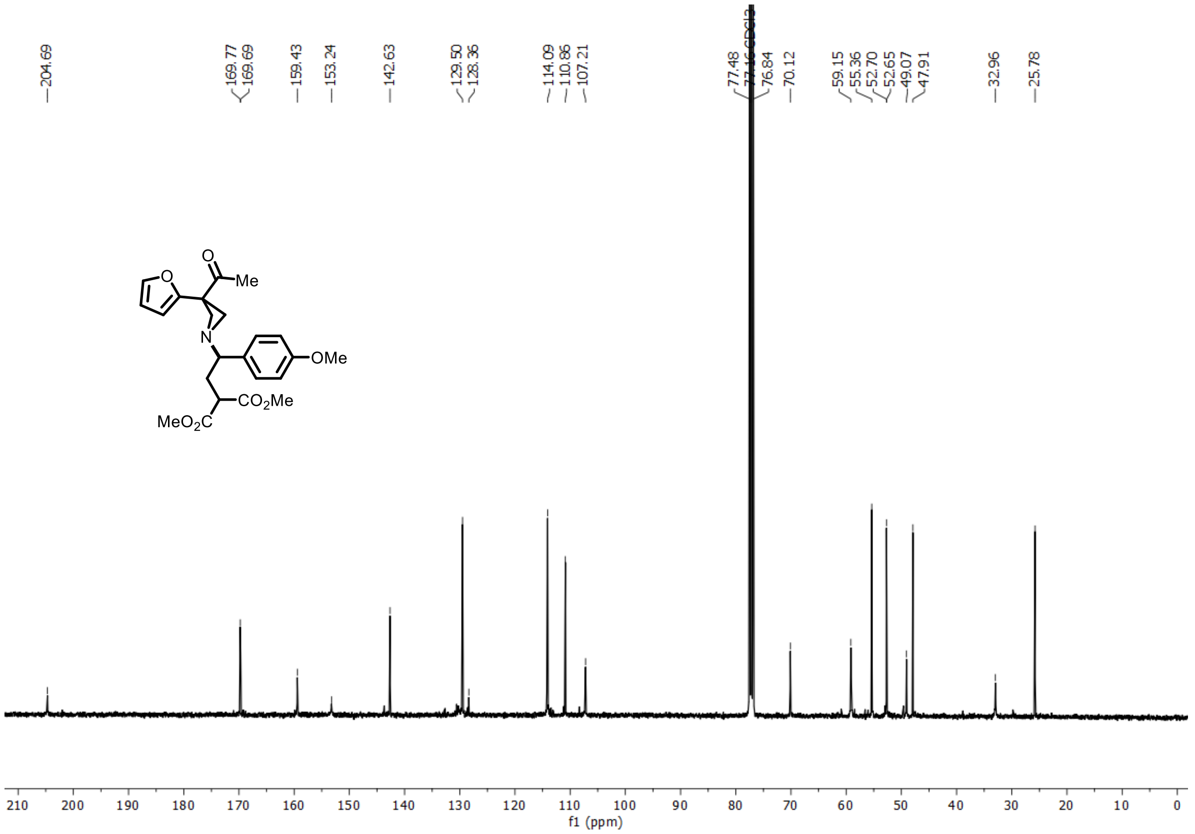

^{1}H spectra at 400 MHz and ^{13}C{^{1}H} NMR spectra at 100 MHz in DMSO-d6 (**30**)

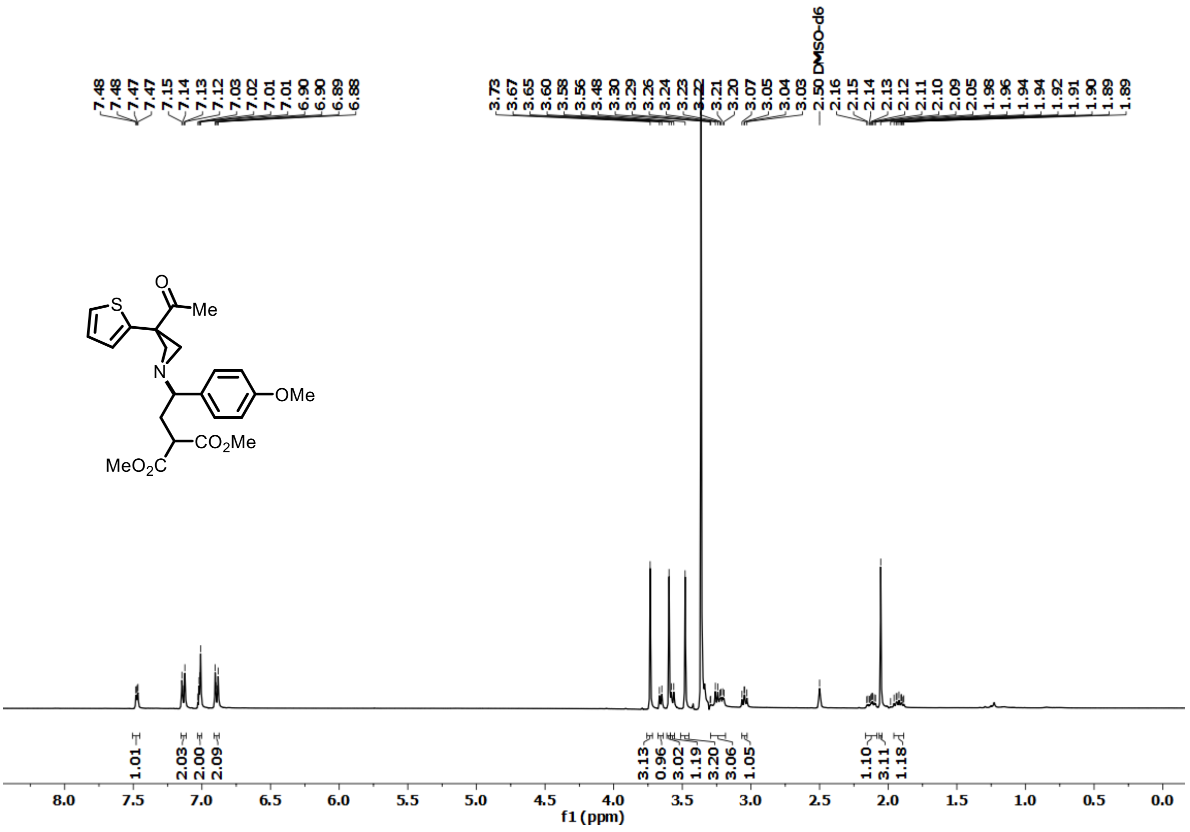

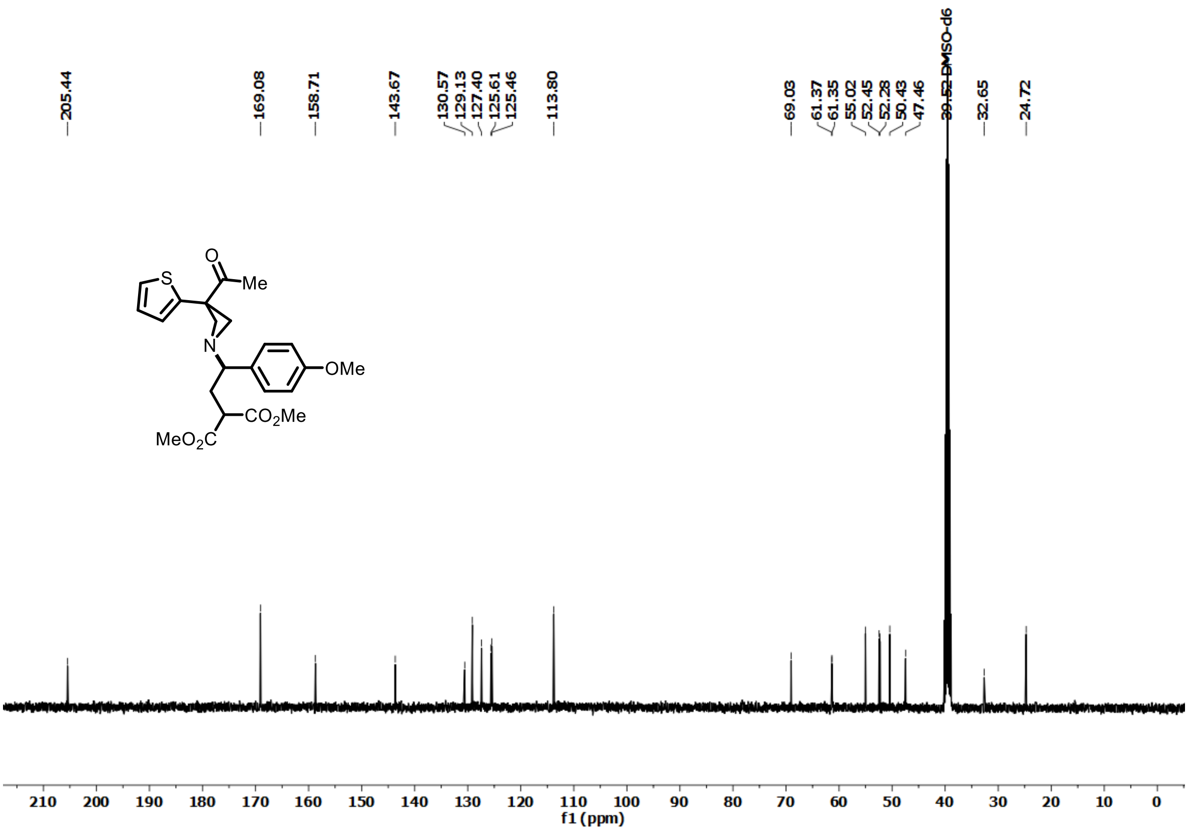

^{1}H spectra at 400 MHz and $^{13}C\{^{1}H\}$ NMR spectra at 100 MHz in DMSO-d6 (**31**)

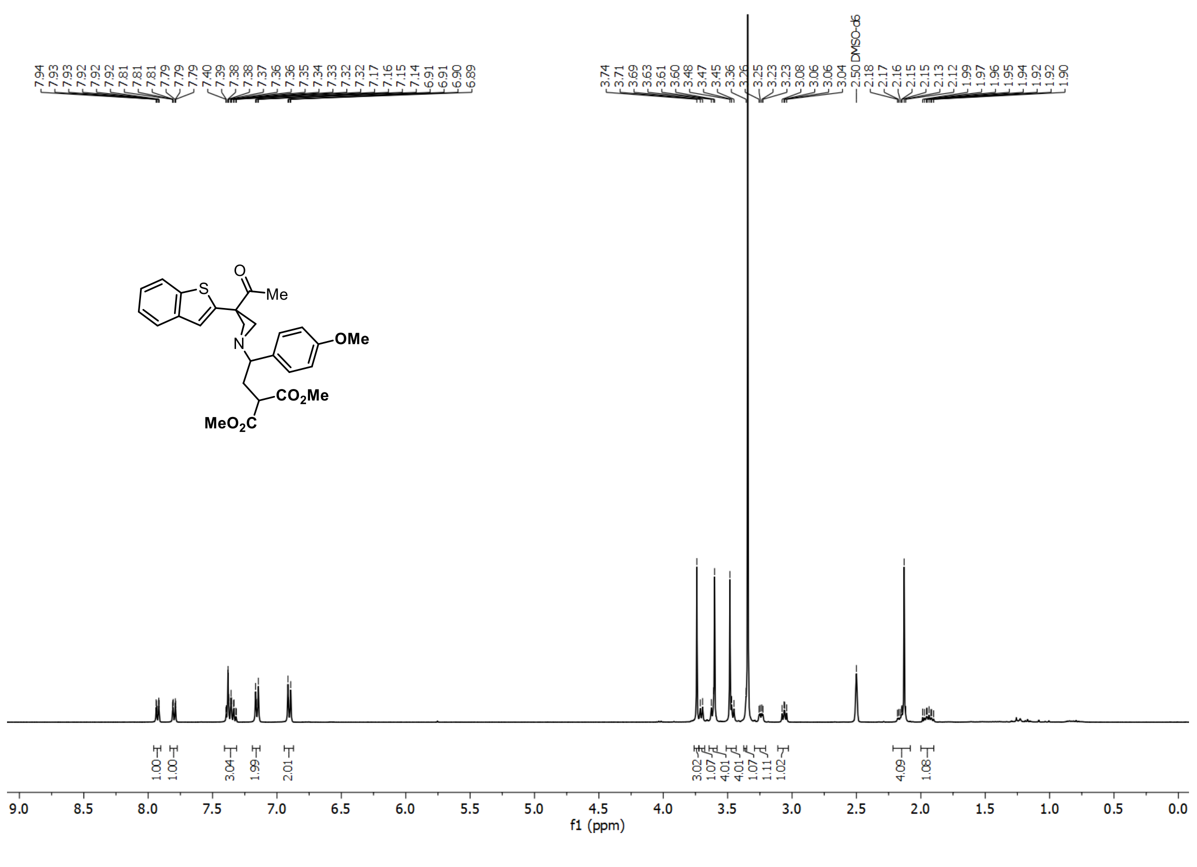

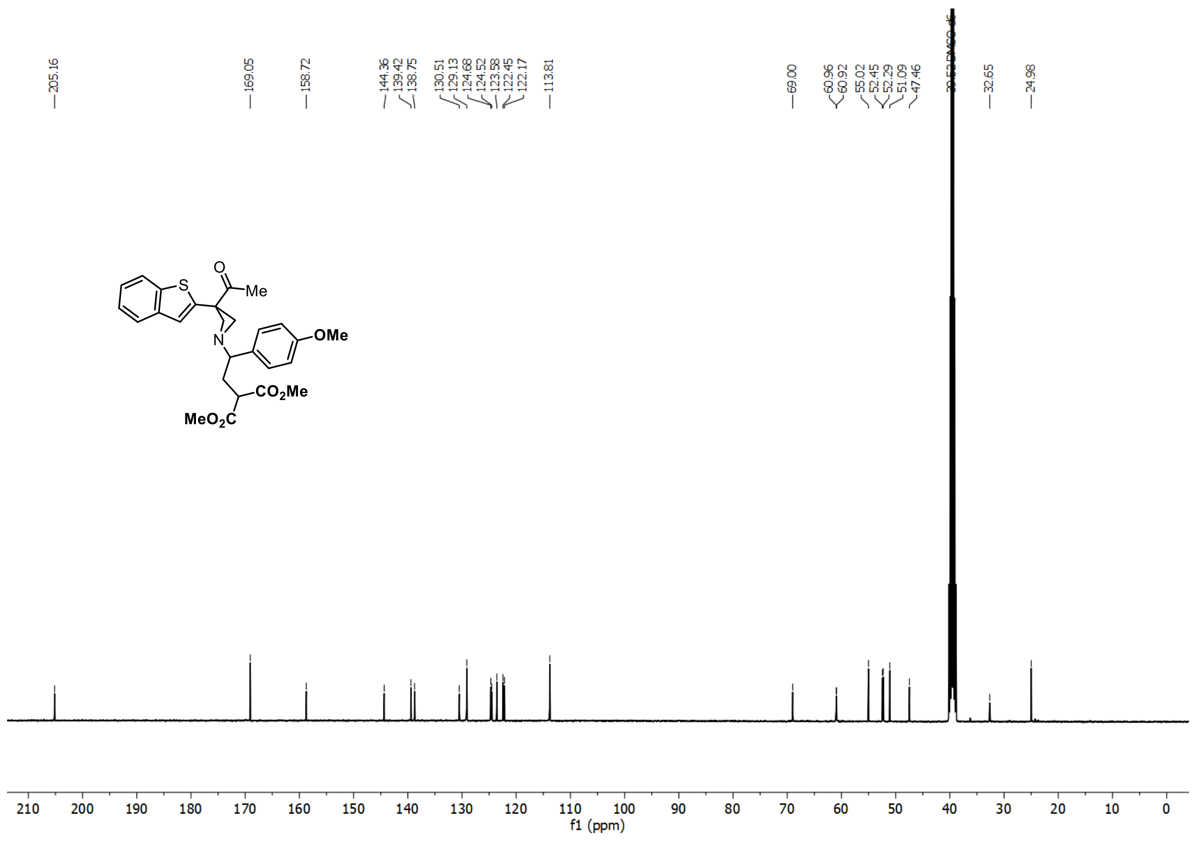

^{1}H spectra at 400 MHz and ^{13}C{^{1}H} NMR spectra at 100 MHz in DMSO-d6 (**32**)

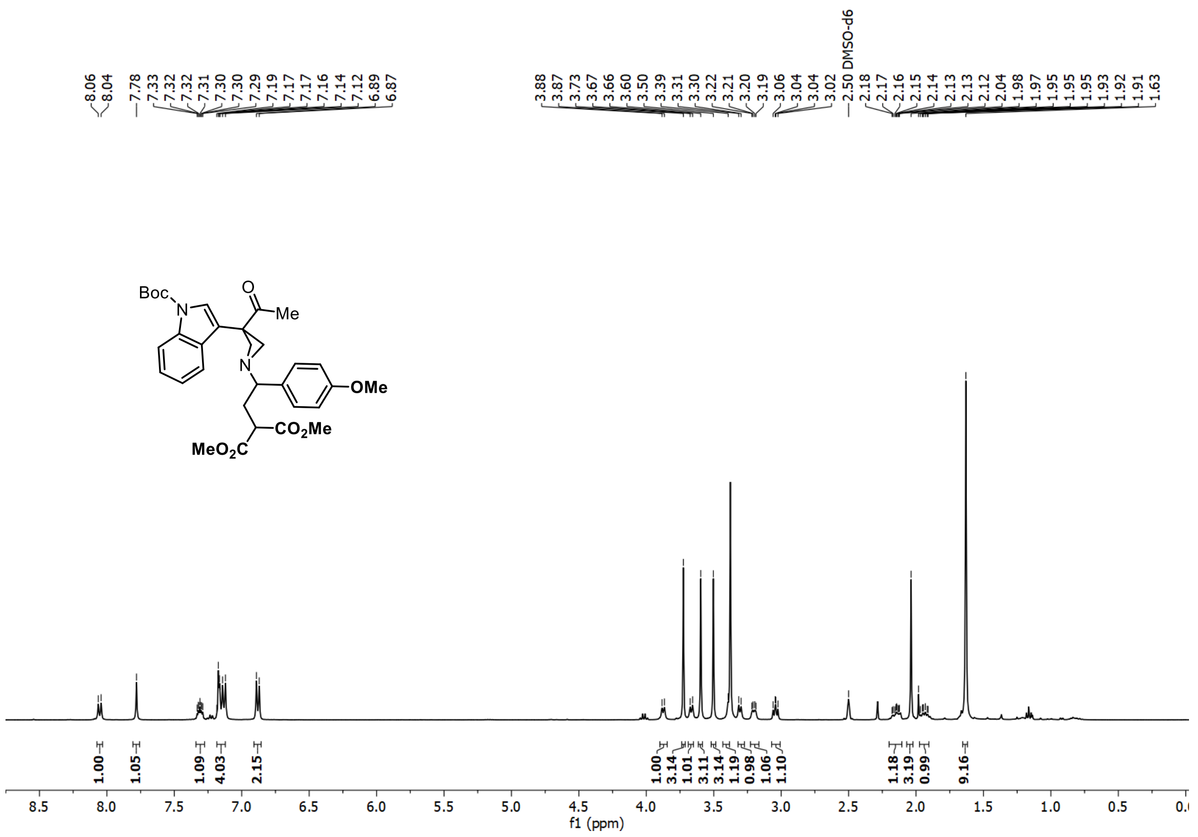

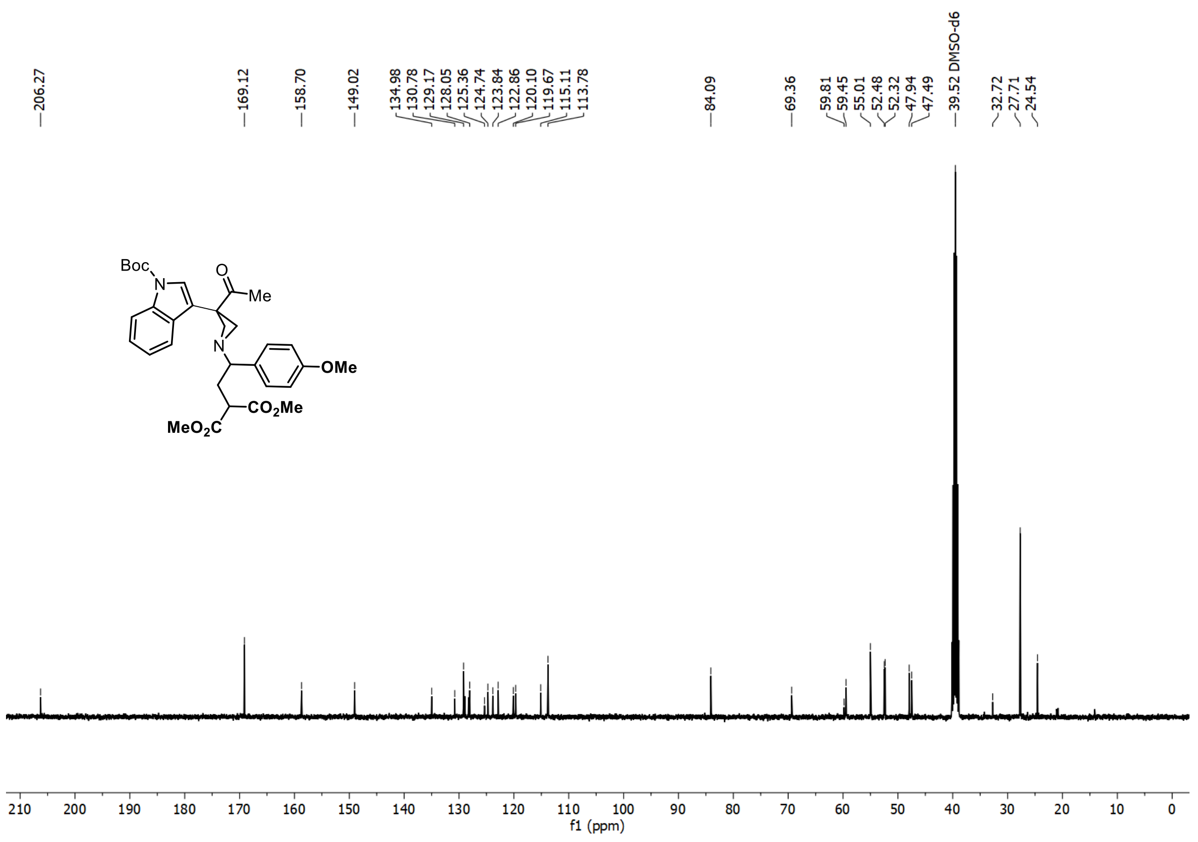

^{1}H spectra at 400 MHz and ^{13}C{^{1}H} NMR spectra at 100 MHz in DMSO- d6 (**33**)

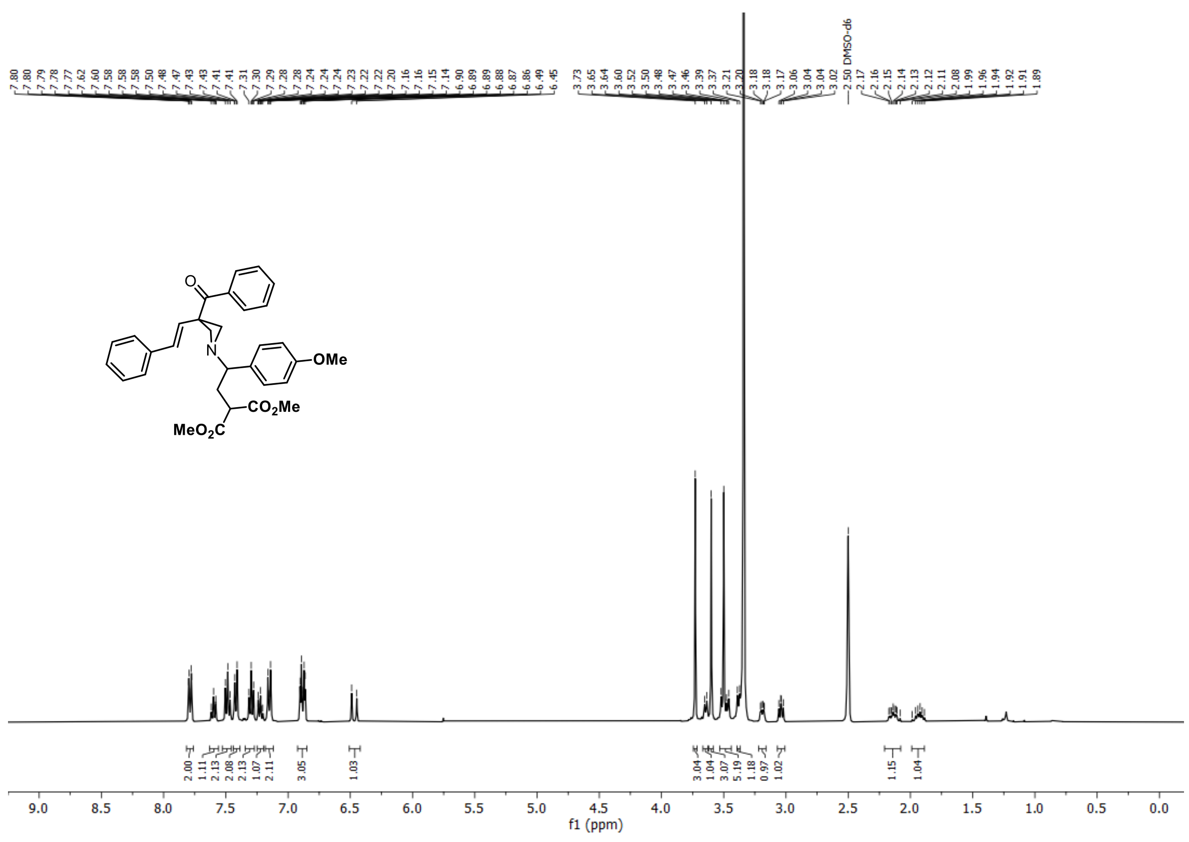

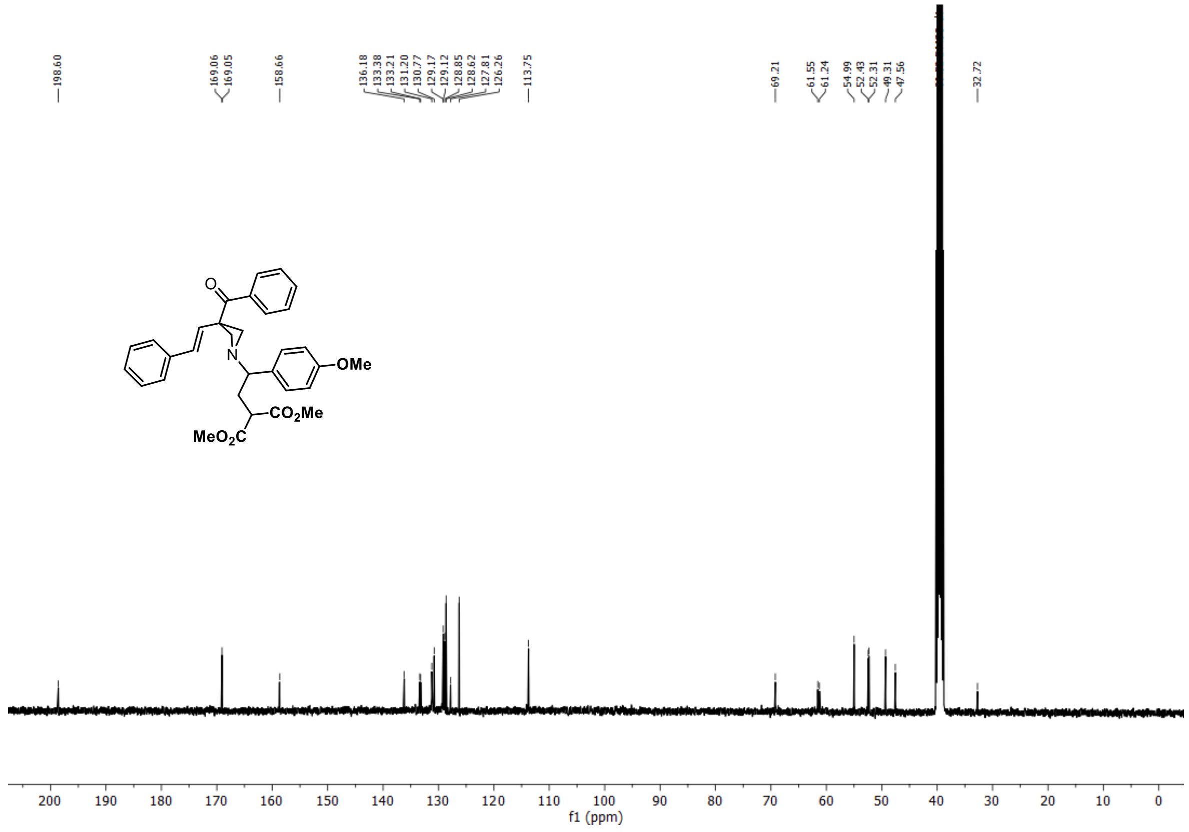

^{1}H spectra at 400 MHz and ^{13}C{^{1}H} NMR spectra at 100 MHz in CDCl3 (**34**)

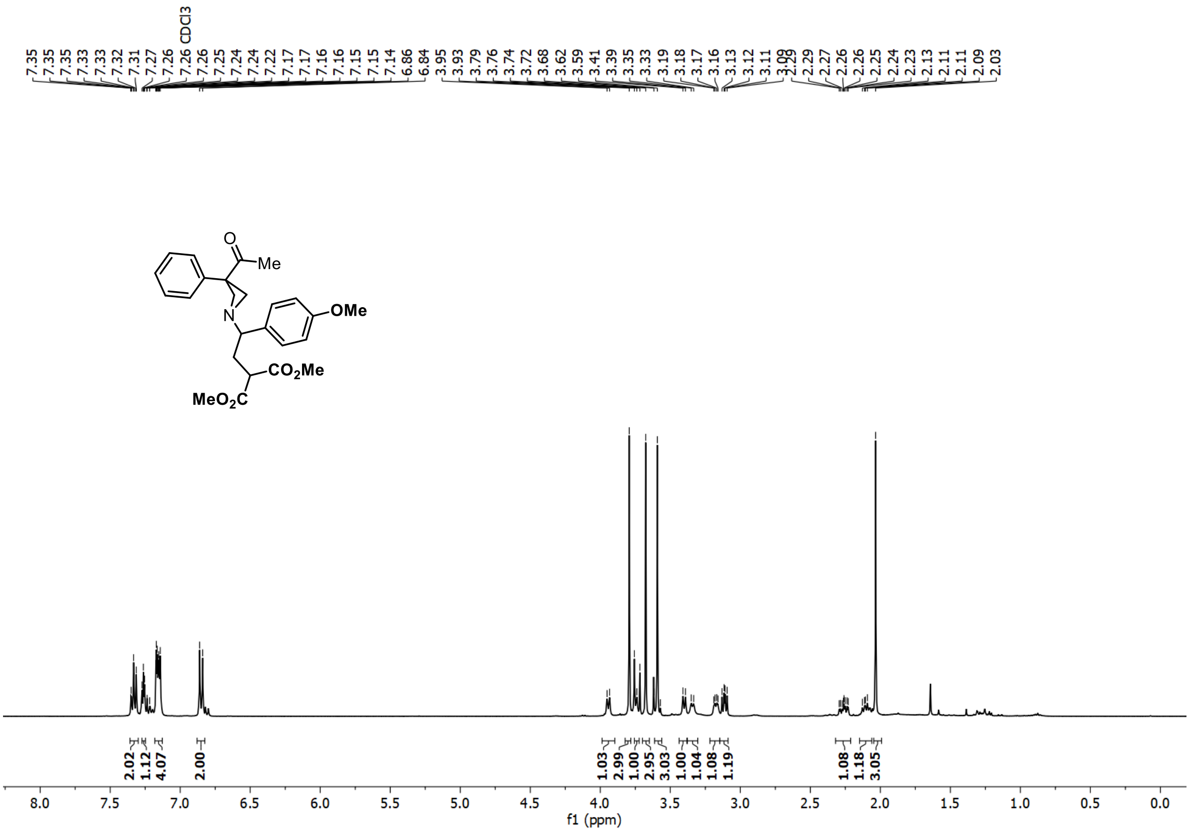

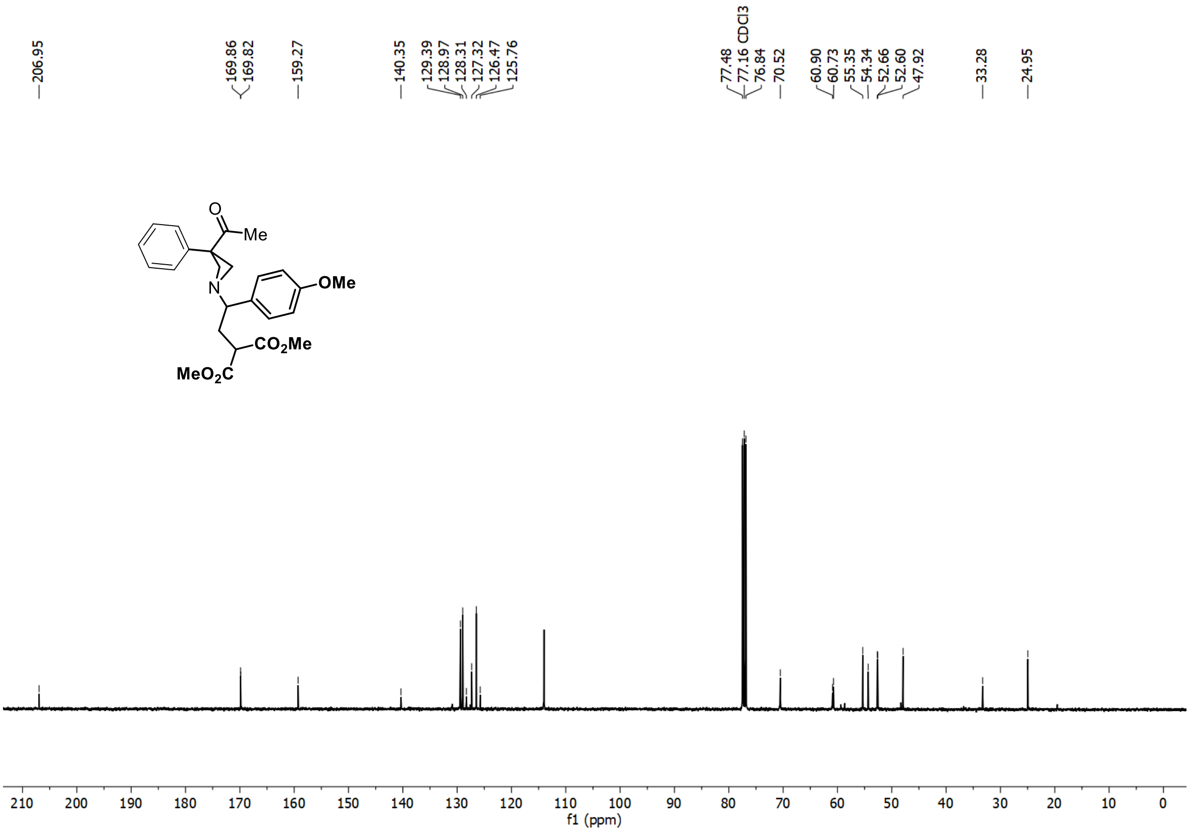

^{1}H spectra at 400 MHz and ^{13}C{^{1}H} NMR spectra at 100 MHz in DMSO-d6 (**35**)

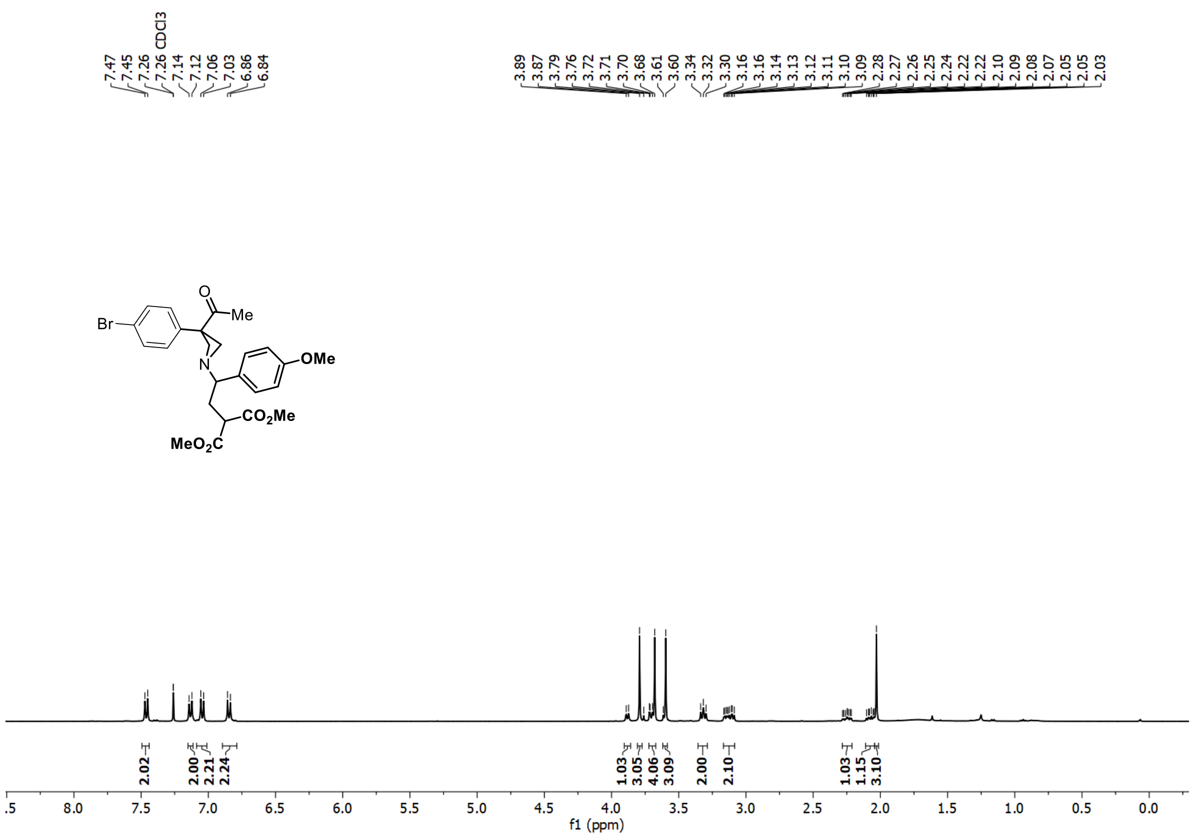

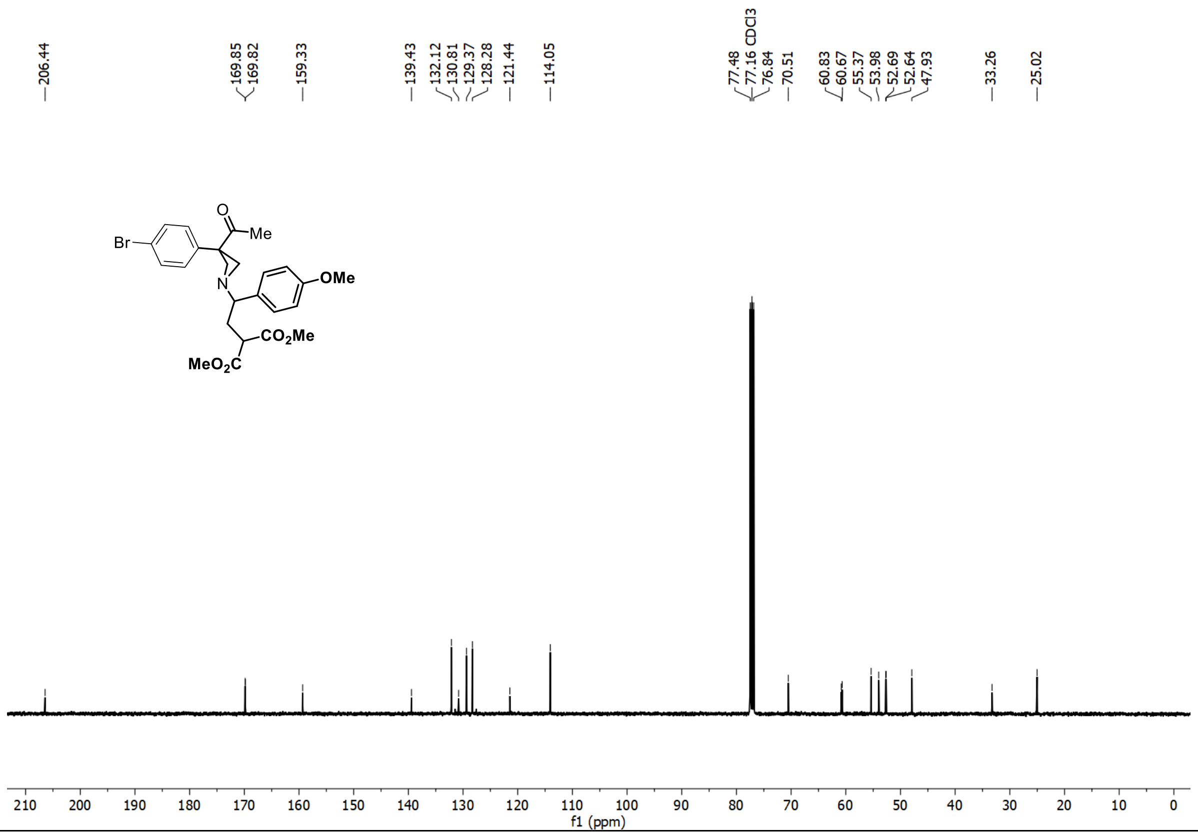

^{1}H spectra at 400 MHz and ^{13}C{^{1}H} NMR spectra at 100 MHz in DMSO-d6 (**36**)

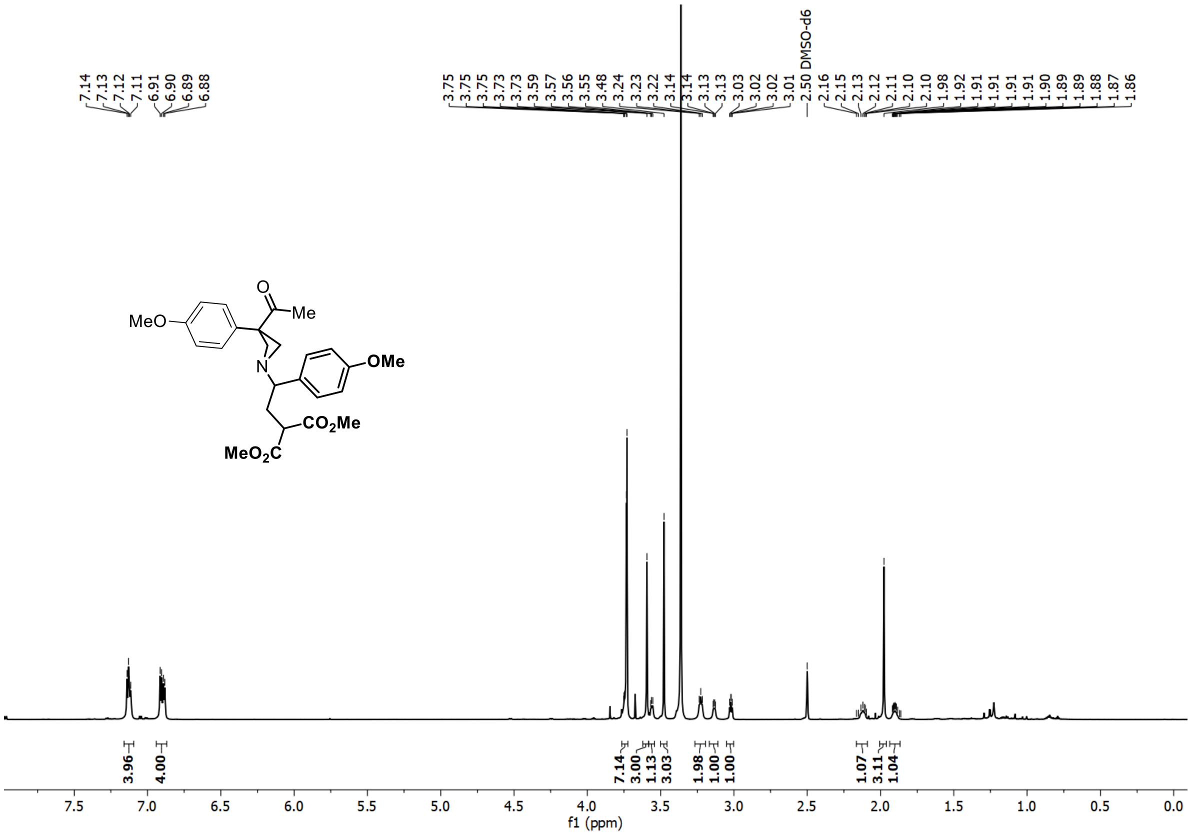

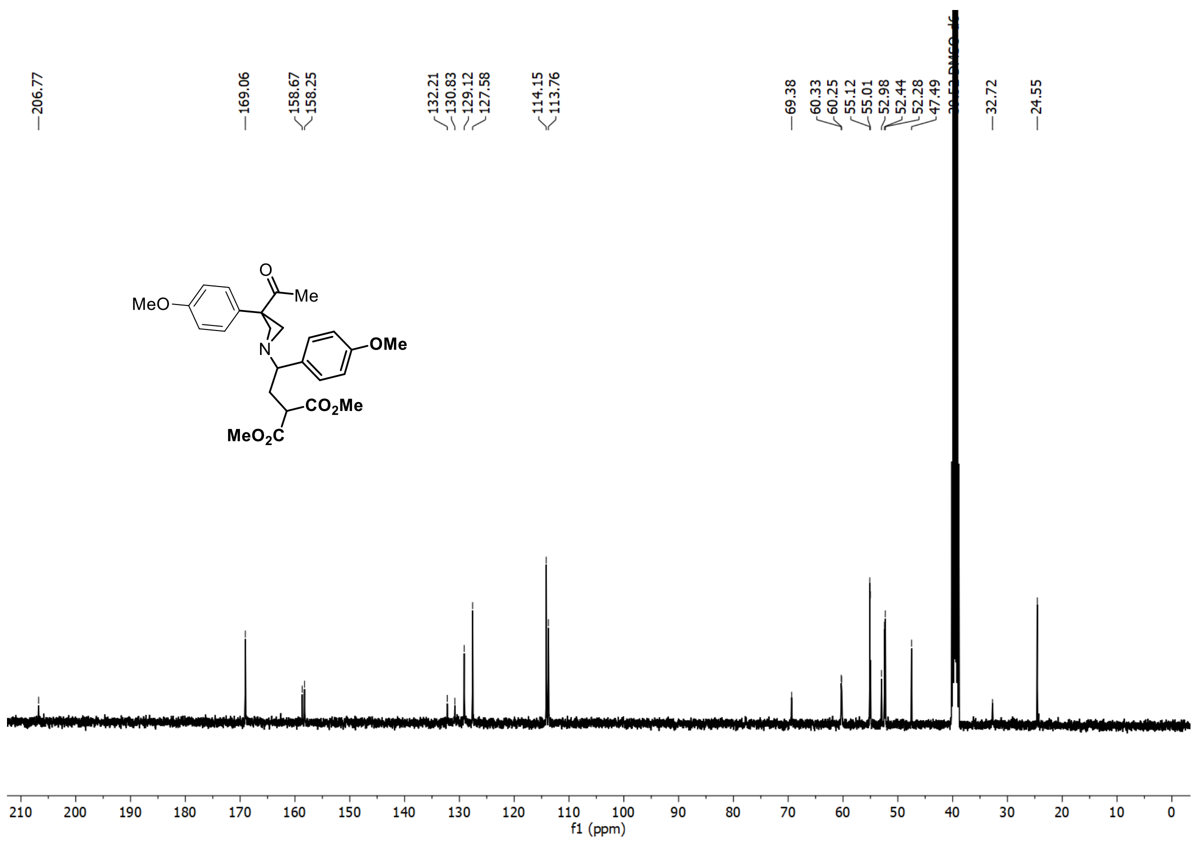

^{1}H spectra at 400 MHz and $^{13}C\{^1H\}$ NMR spectra at 100 MHz in $CDCl_3$ (**37**)

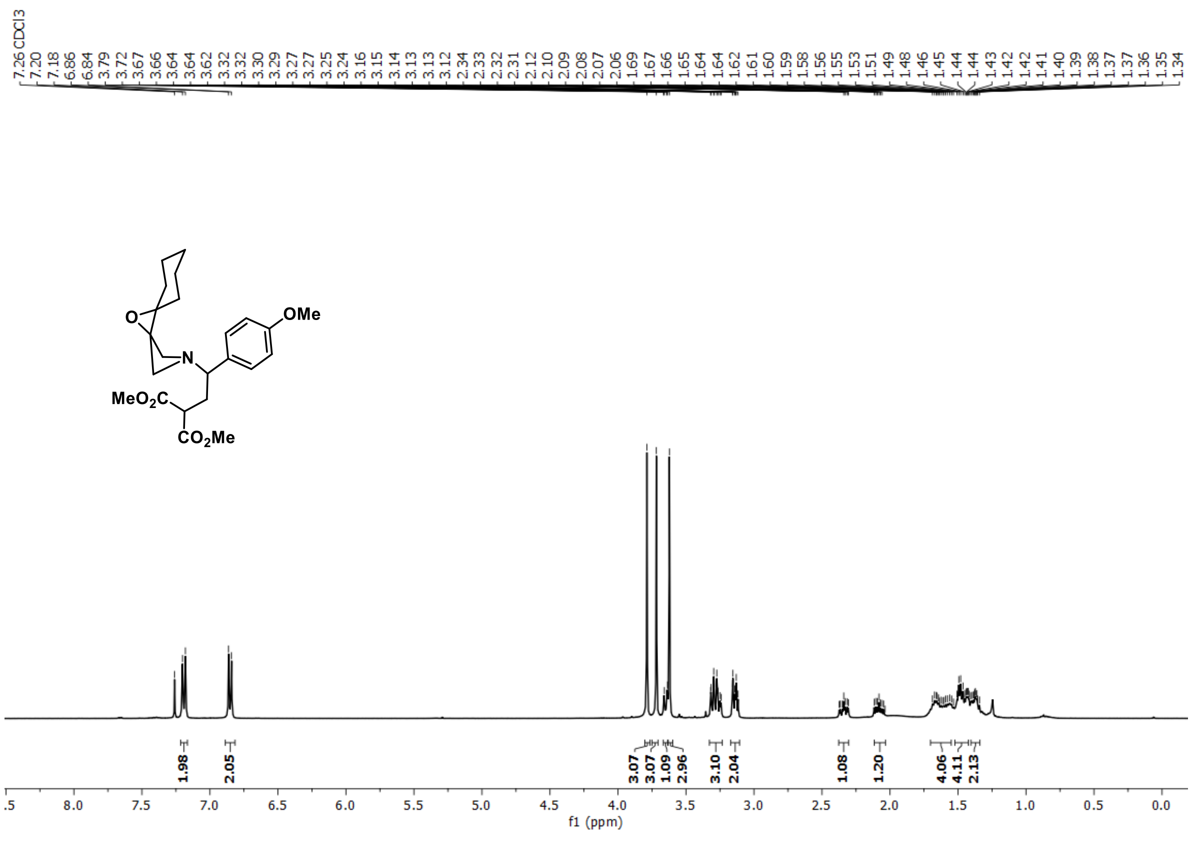

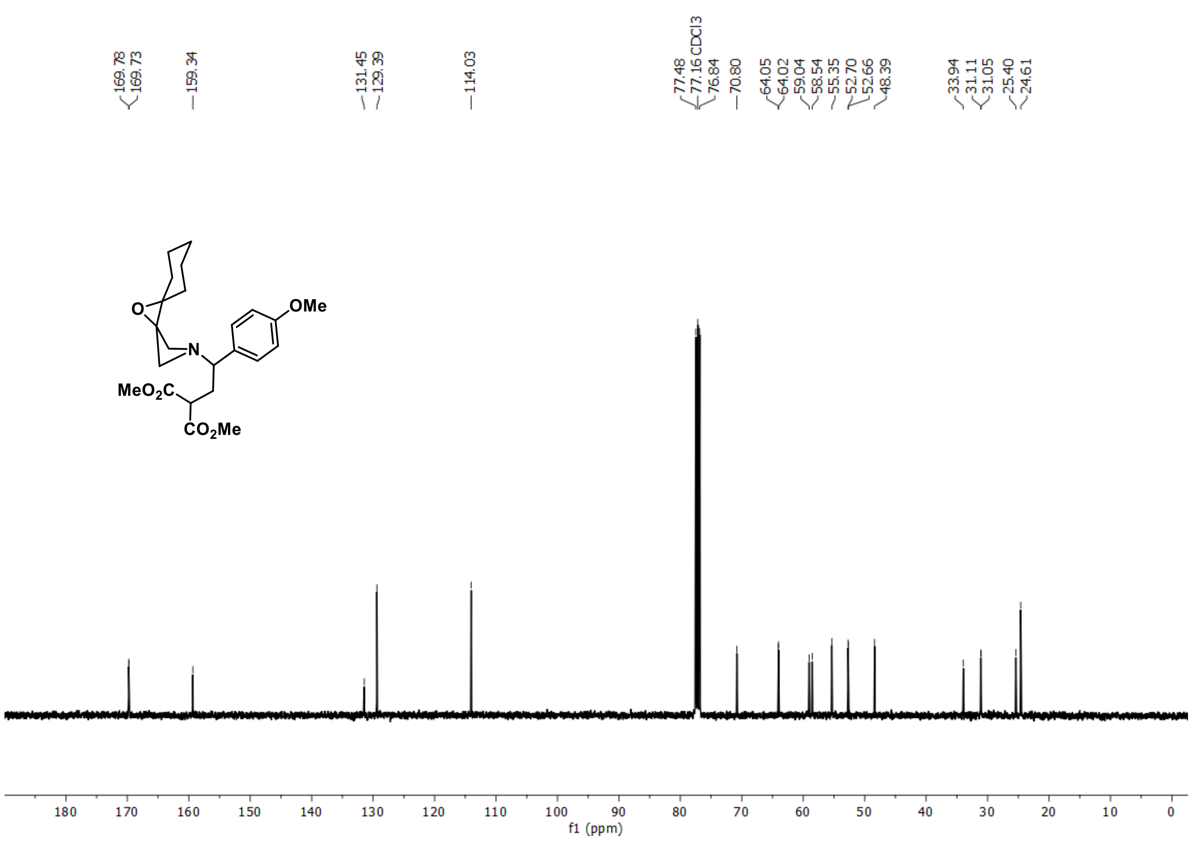

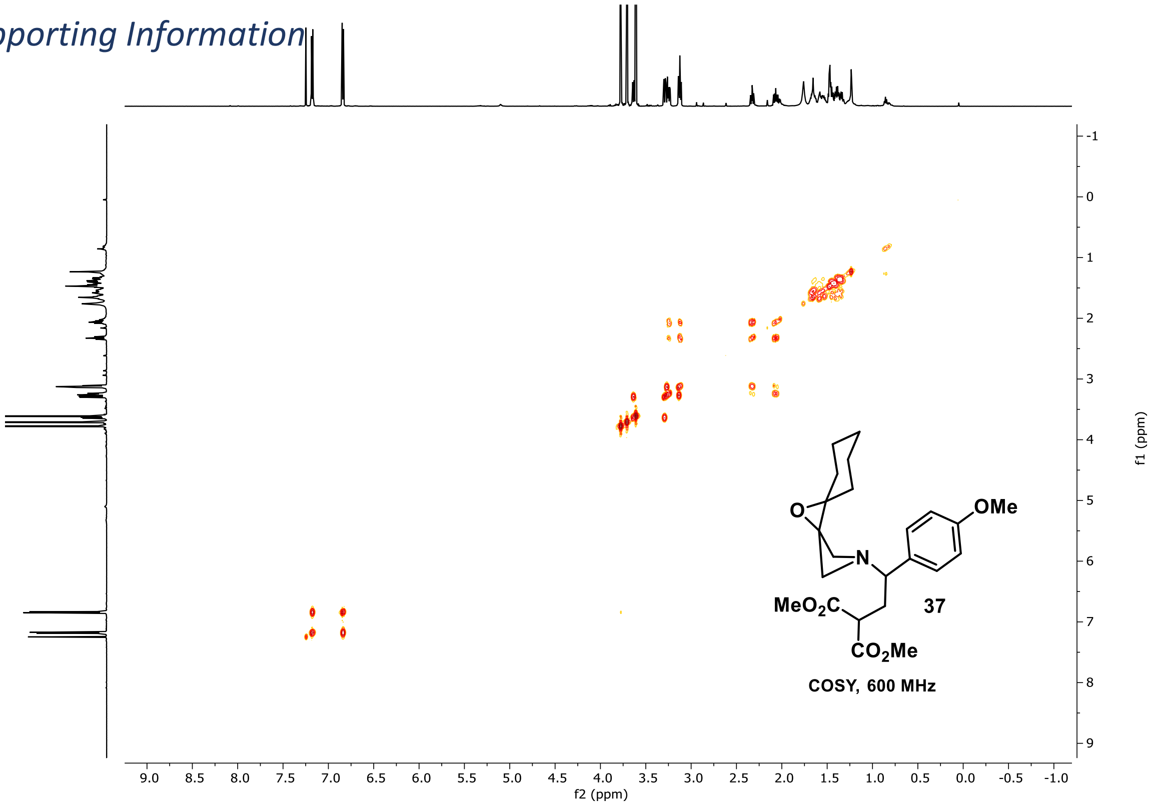
OMe
O
N
MeO2C
37
CO2Me
COSY, 600 MHz
f2 (ppm)
f1 (ppm)

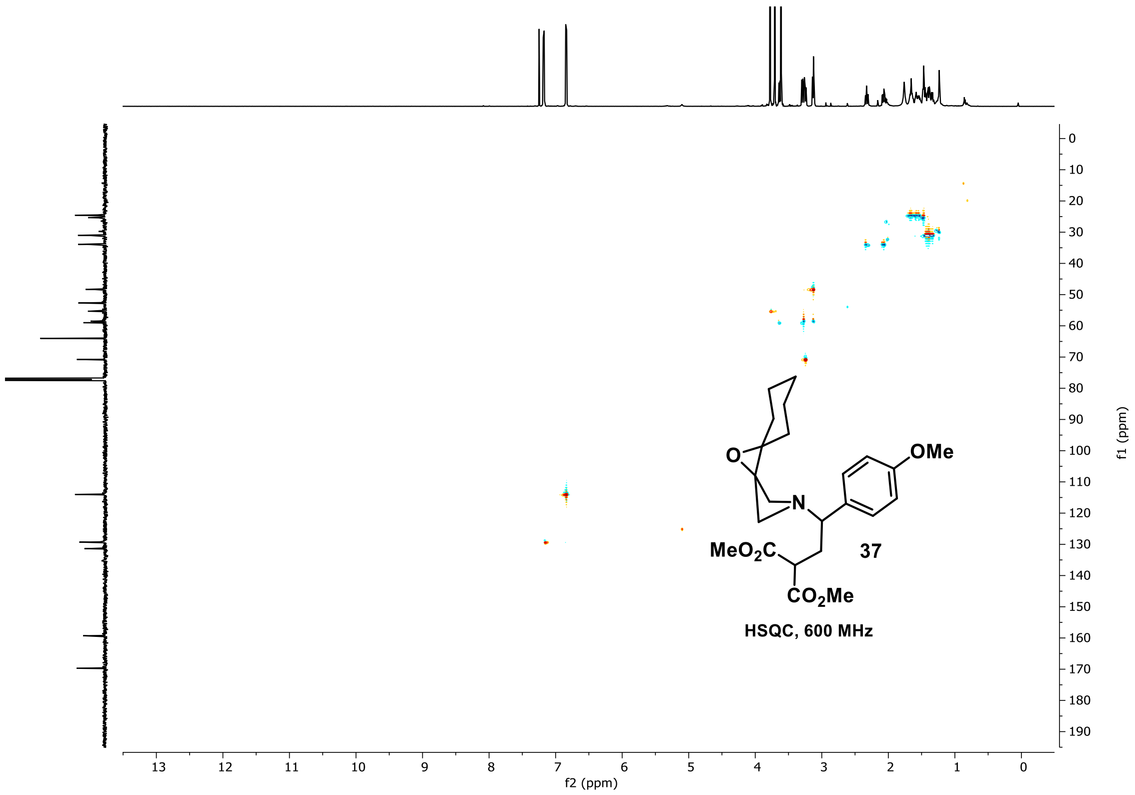
OMe
O
N
MeO2C
37
CO2Me
HSQC, 600 MHz
f2 (ppm)
f1 (ppm)

^{1}H spectra at 400 MHz and $^{13}C\{^{1}H\}$ NMR spectra at 100 MHz in $CDCl_3$ (**38**)

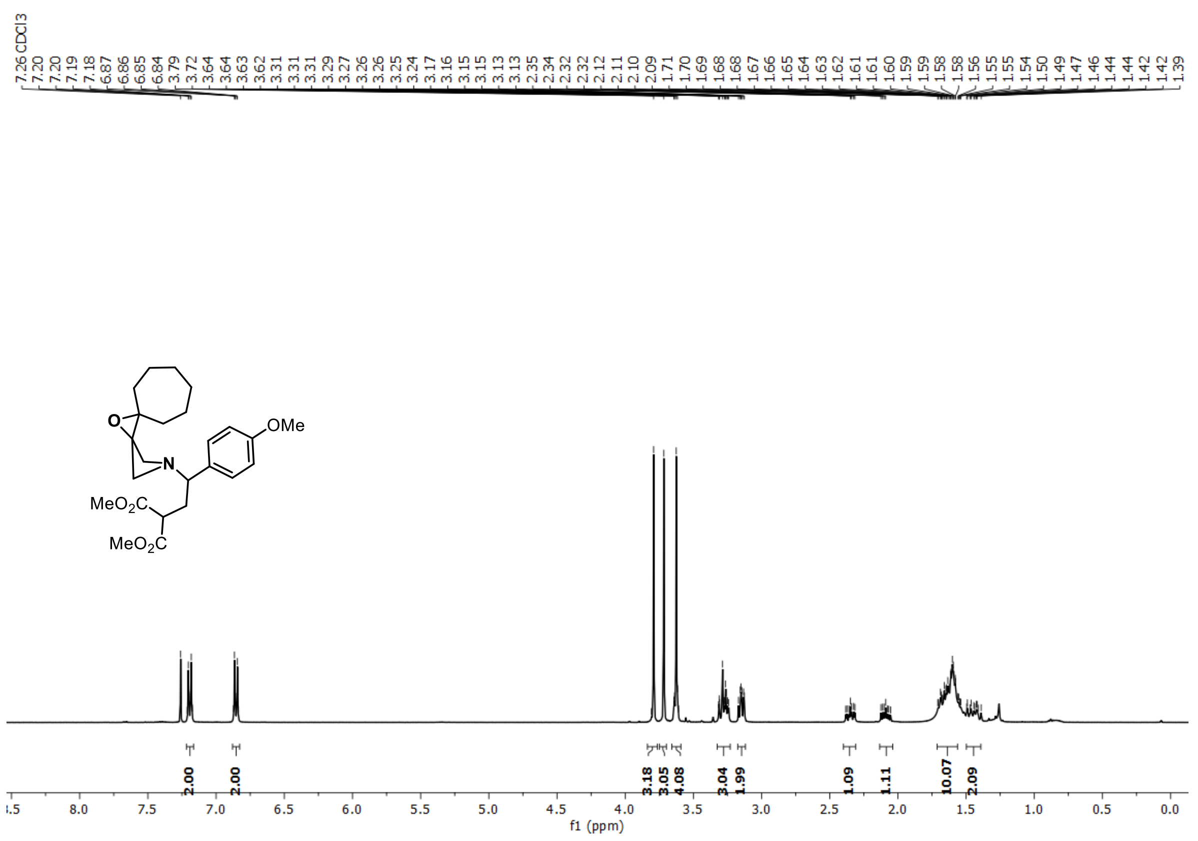

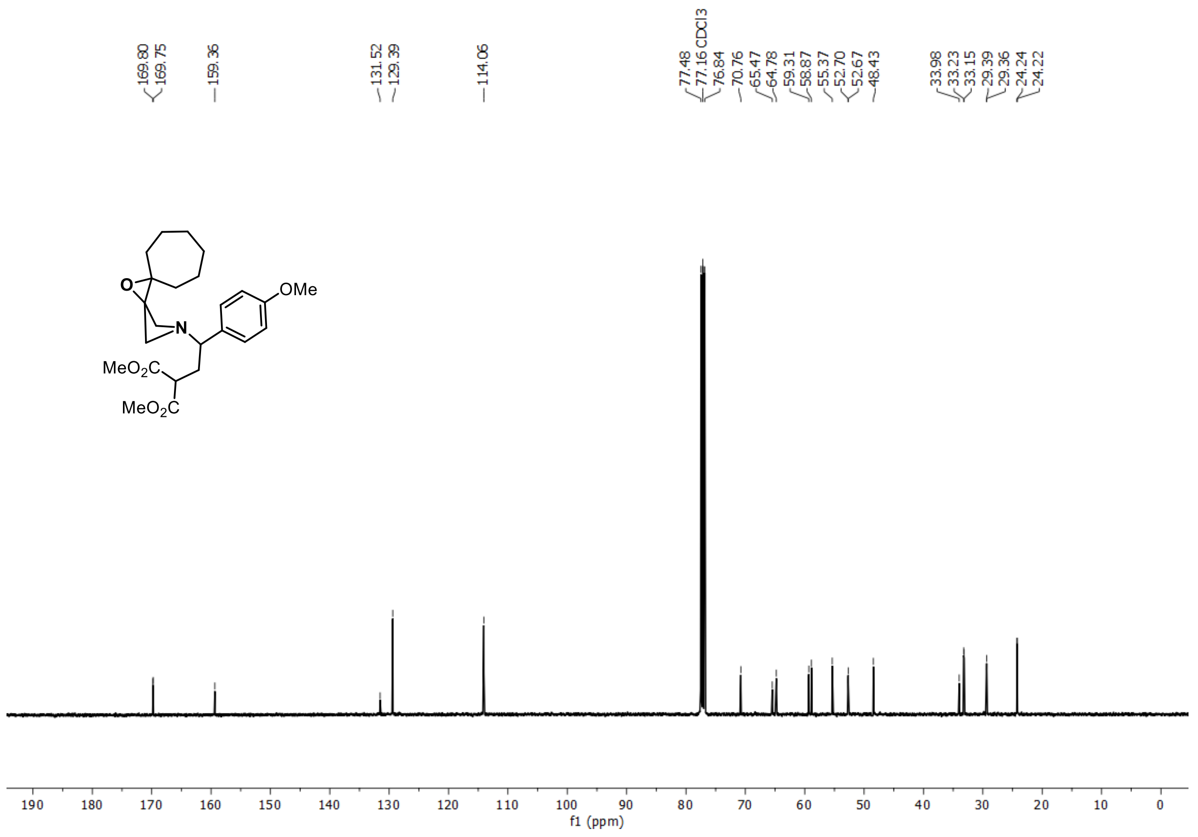

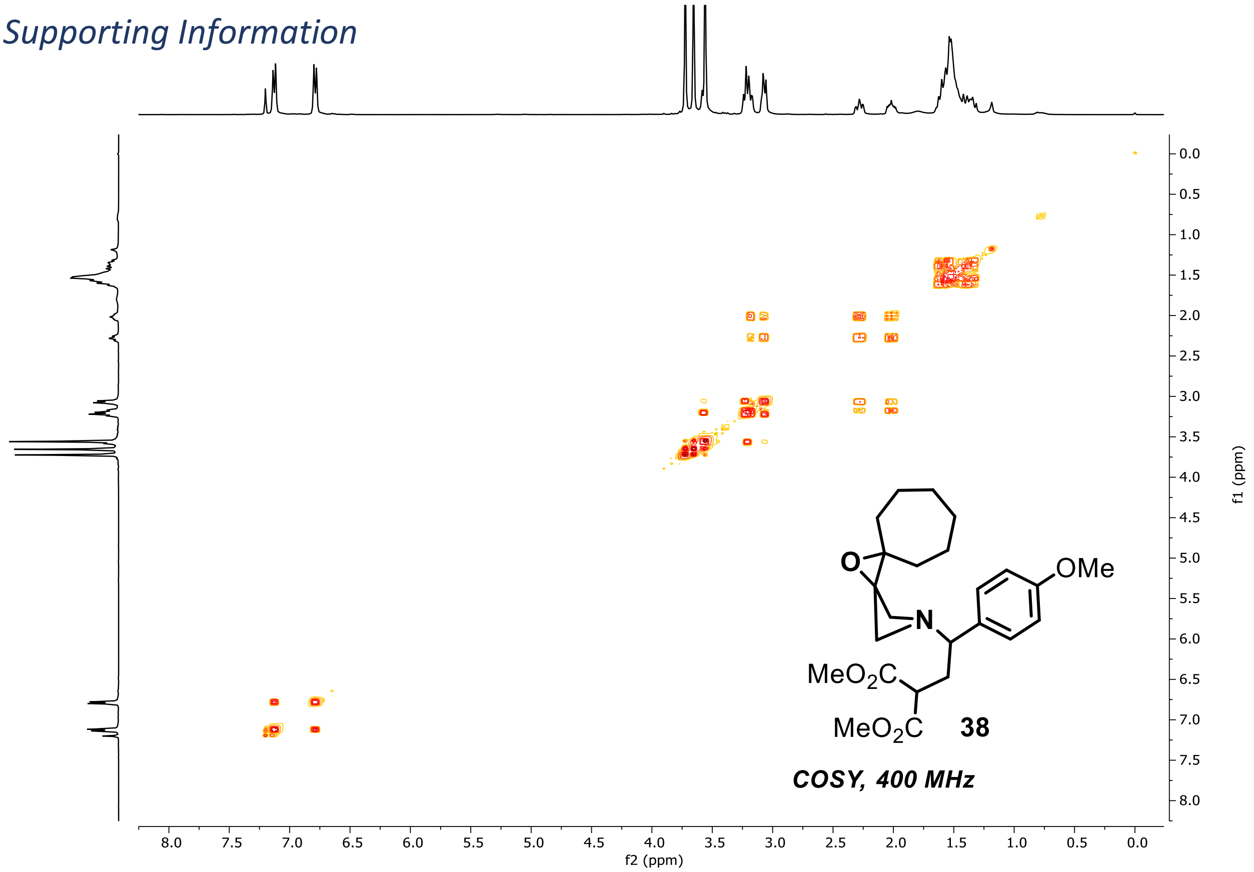
O
N
OMe
MeO2C
MeO2C
38
COSY, 400 MHz
f2 (ppm)
f1 (ppm)

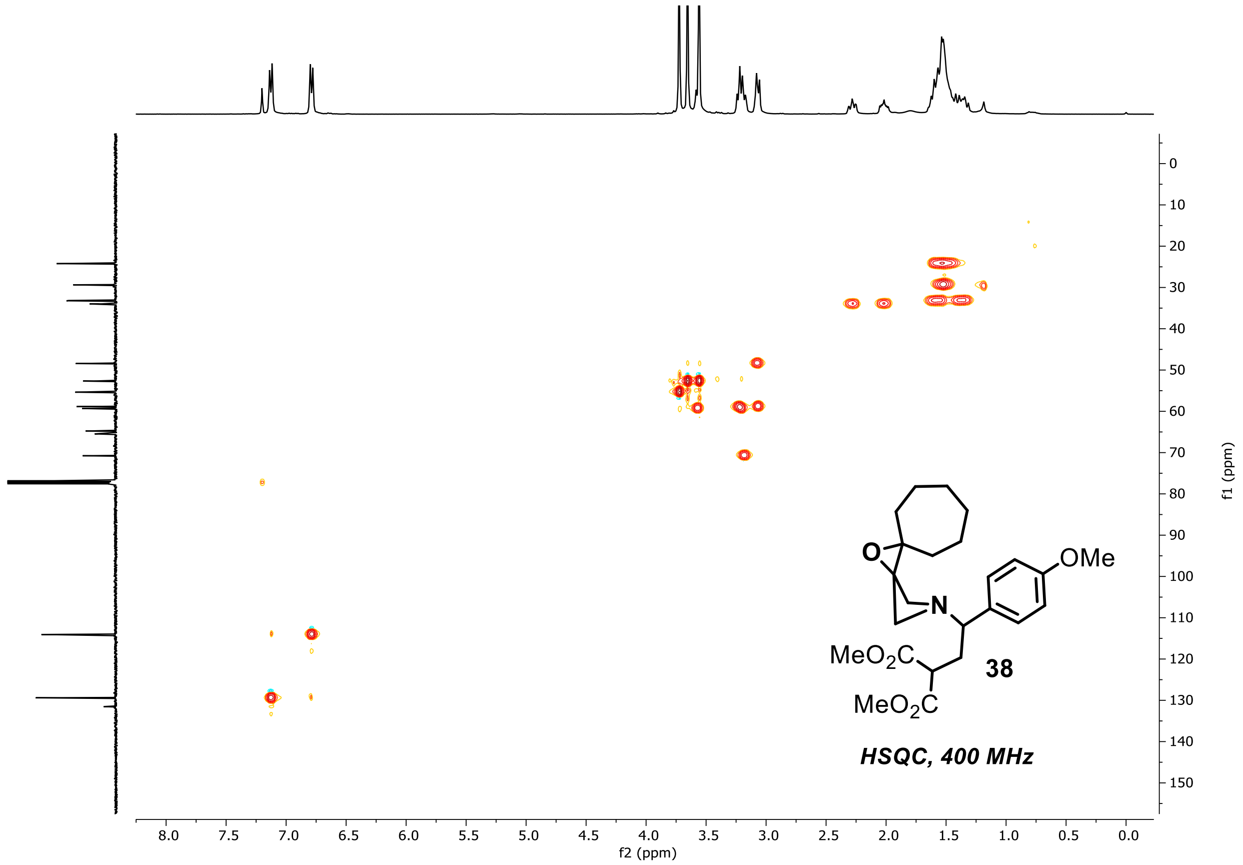
O
N
OMe
MeO2C
MeO2C
38
HSQC, 400 MHz
f2 (ppm)
f1 (ppm)

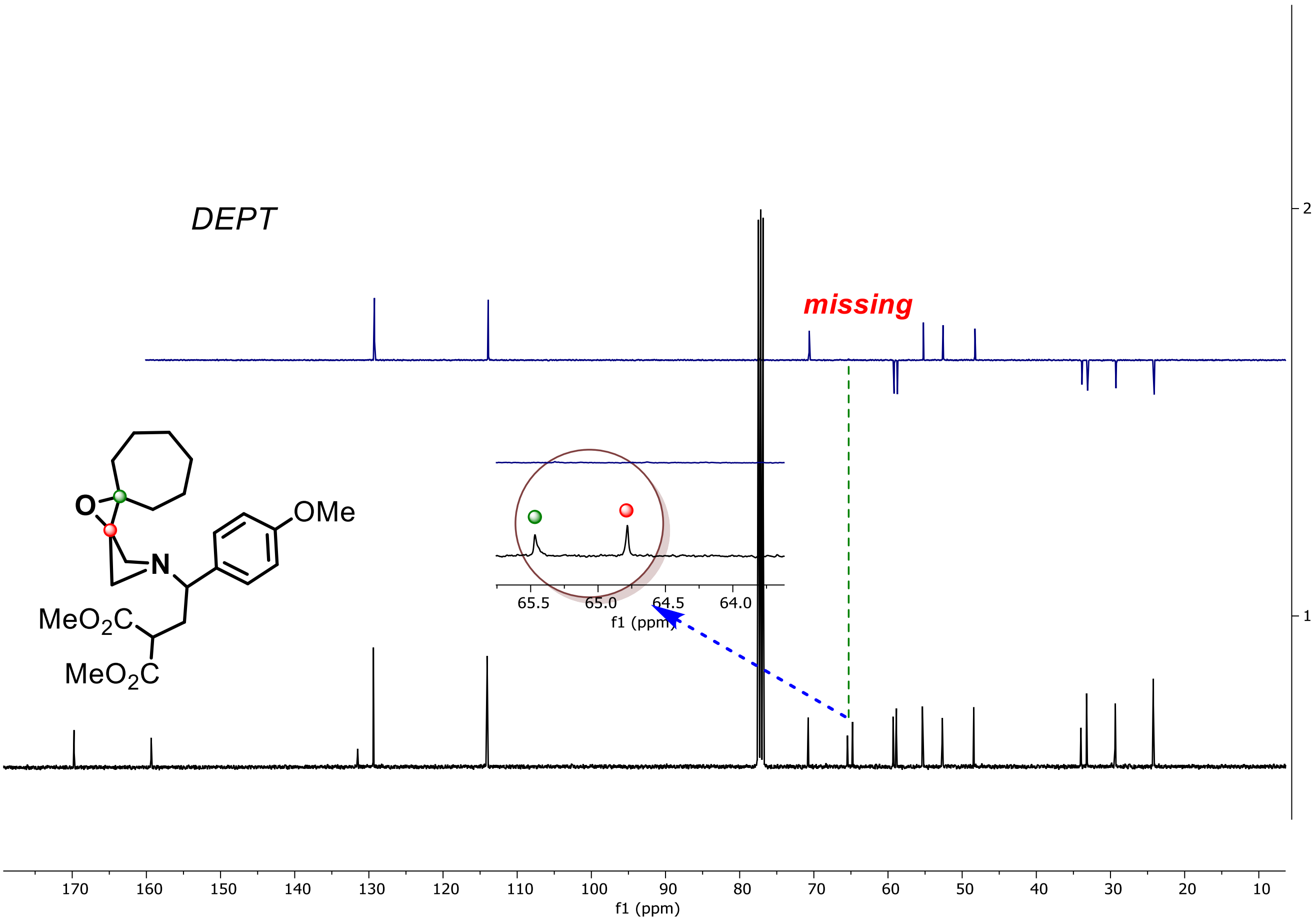
DEPT
missing
OMe
O
N
MeO2C
MeO2C
65.5
65.0
64.5
64.0
f1 (ppm)
2
1
170
160
150
140
130
120
110
100
90
80
70
60
50
40
30
20
10
f1 (ppm)

^{1}H spectra at 400 MHz and ^{13}C{^{1}H} NMR spectra at 100 MHz in DMSO- d6 (**39**)

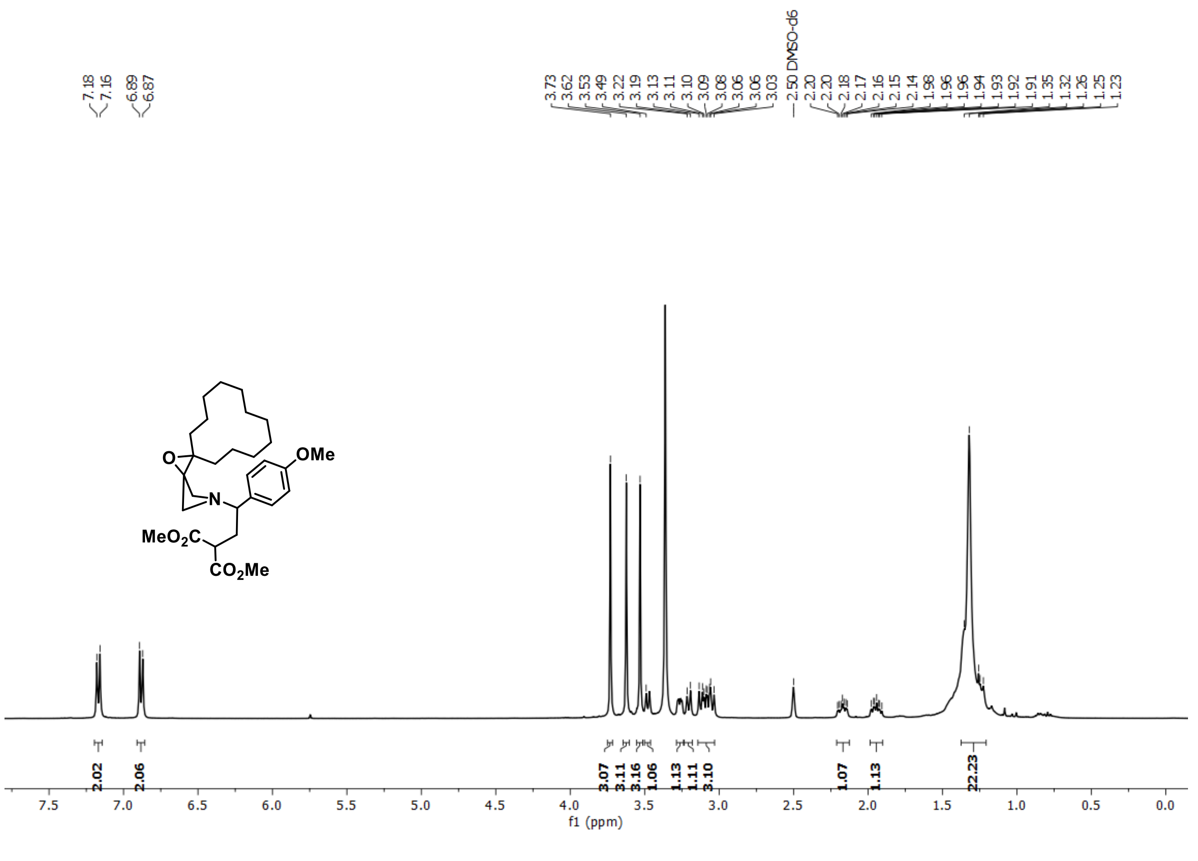

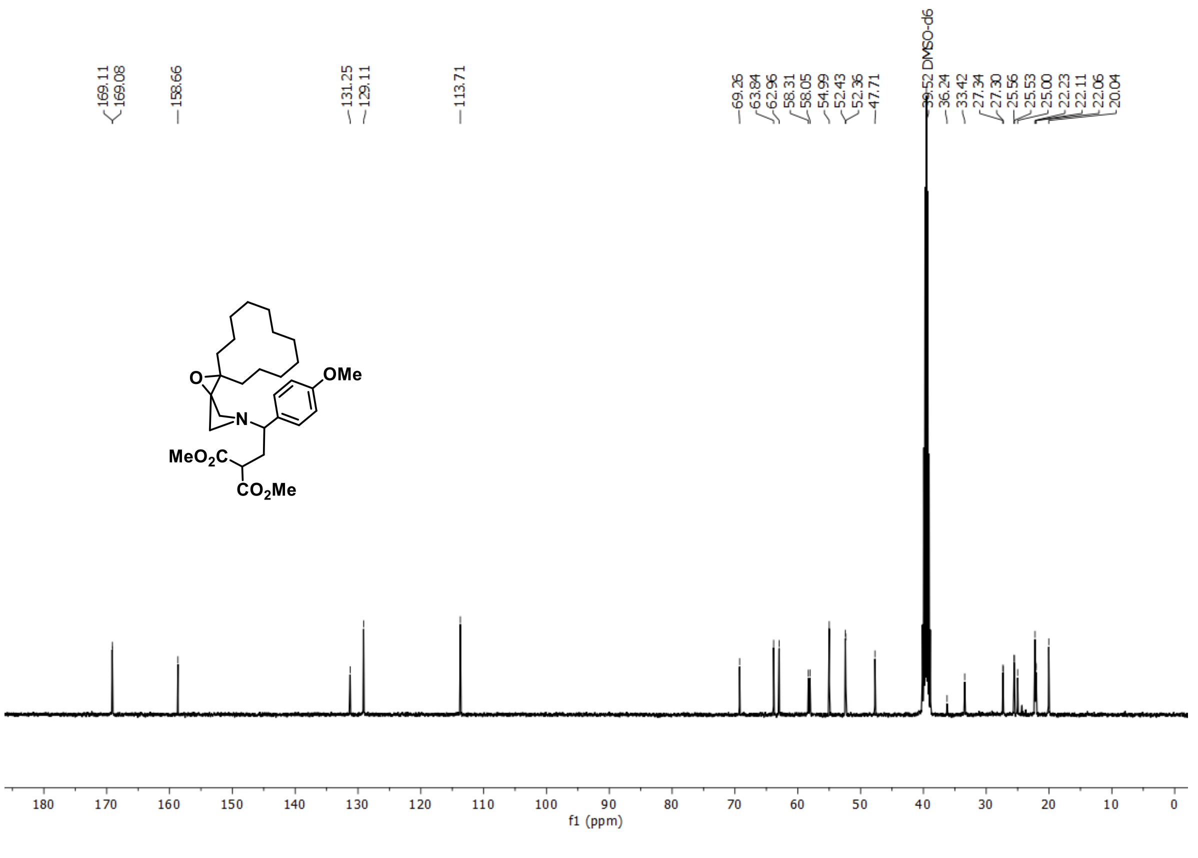

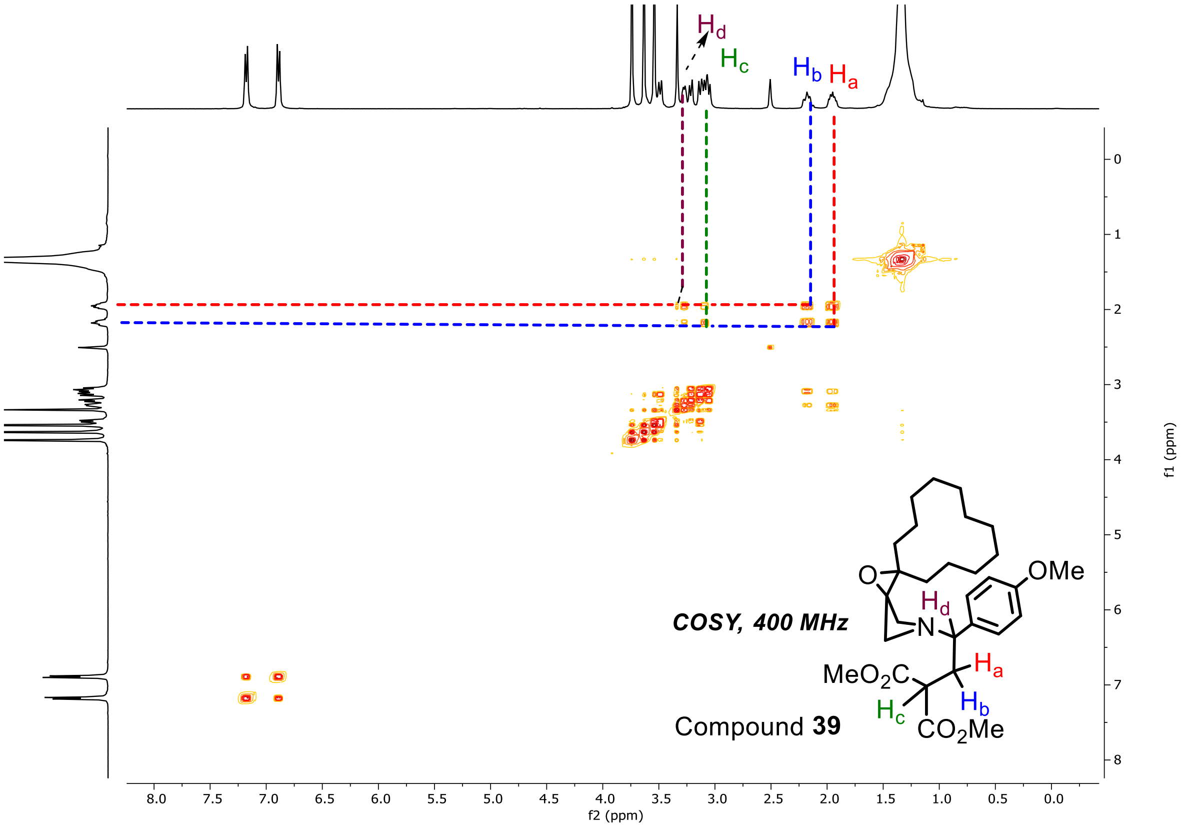
Hd
Hc
Hb
Ha
COSY, 400 MHz
MeO2C
CO2Me
OMe
O
N
Compound 39
f2 (ppm)
f1 (ppm)

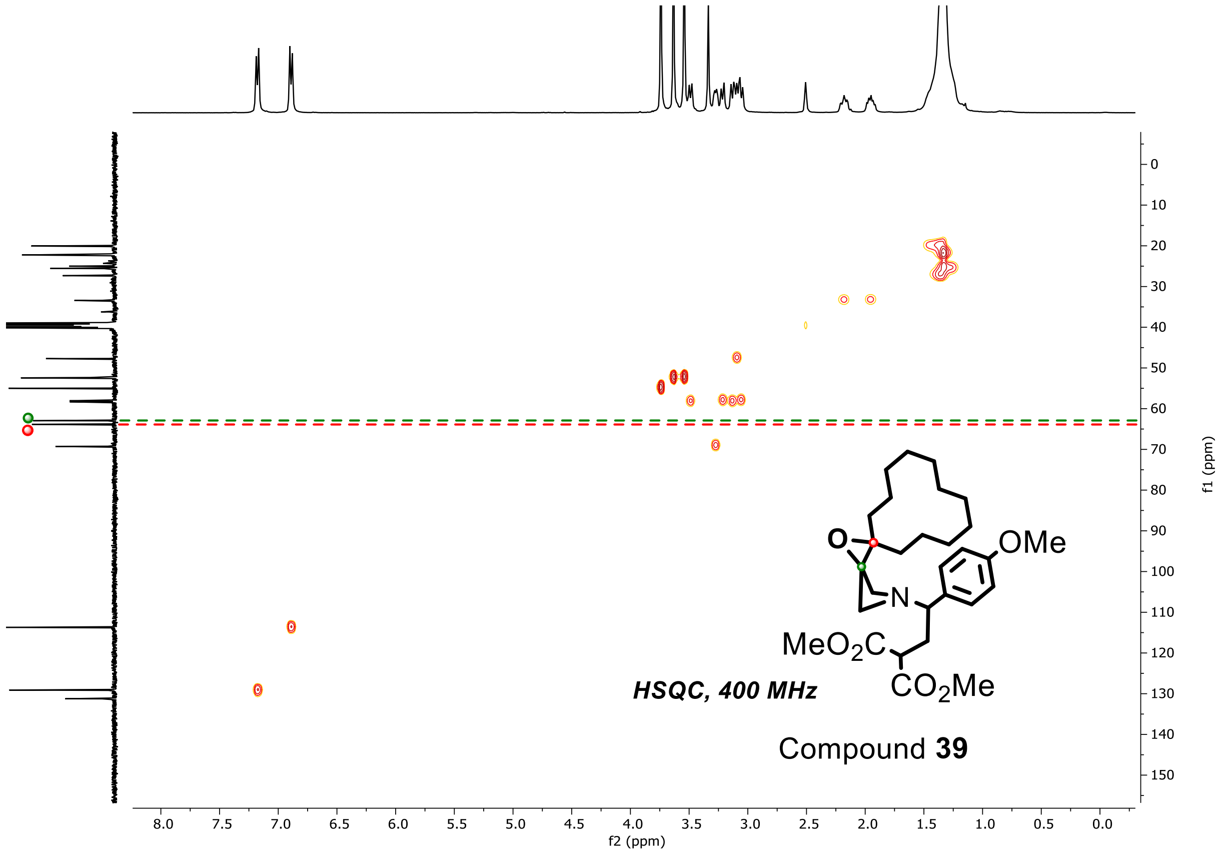
HSQC, 400 MHz
MeO2C
CO2Me
OMe
O
N
Compound 39
f2 (ppm)
f1 (ppm)

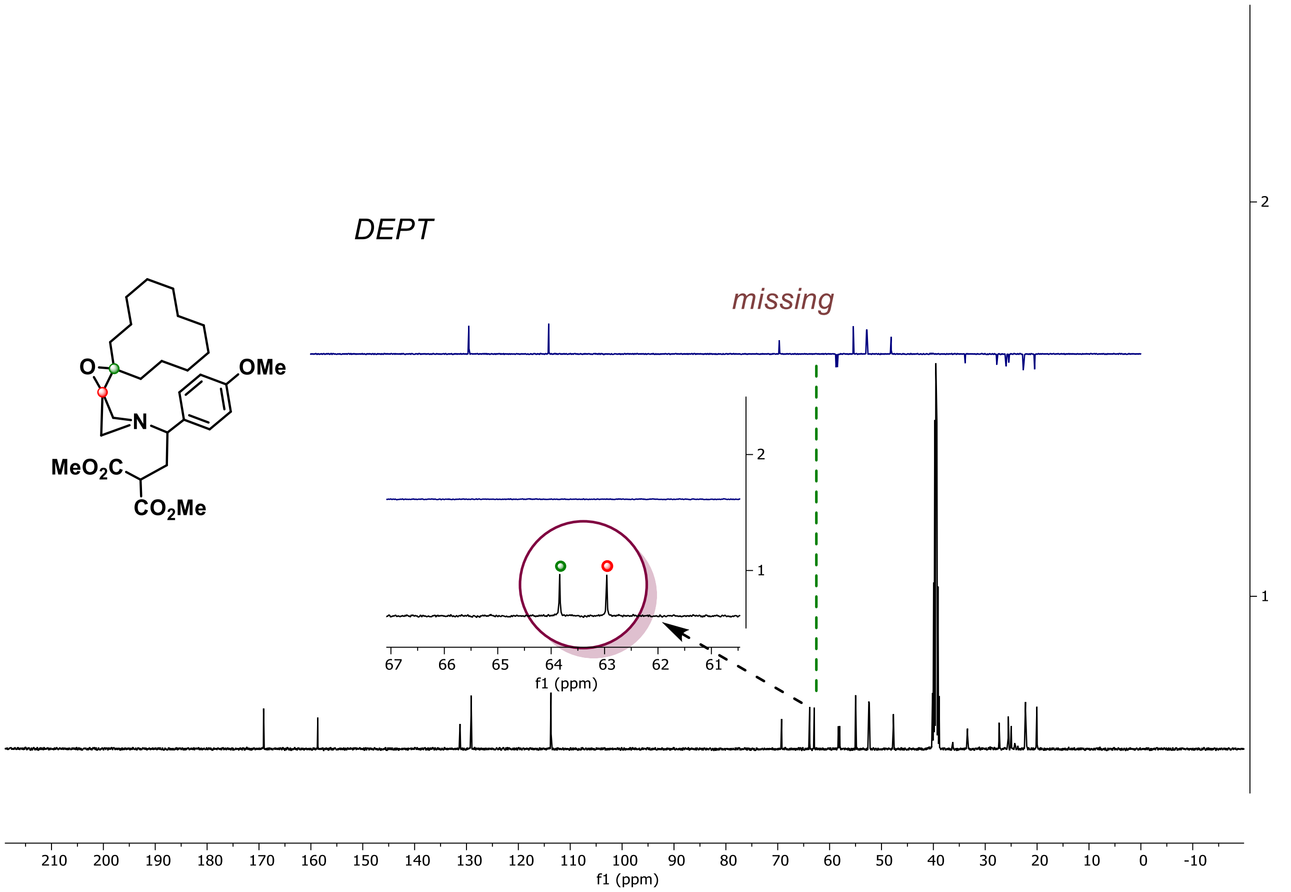

DEPT
missing
OMe
O
N
MeO2C
CO2Me
67
66
65
64
63
62
61
f1 (ppm)
210
200
190
180
170
160
150
140
130
120
110
100
90
80
70
60
50
40
30
20
10
0
-10
f1 (ppm)

^{1}H spectra at 400 MHz and ^{13}C{^{1}H} NMR spectra at 100 MHz in DMSO- d6 (**40**)

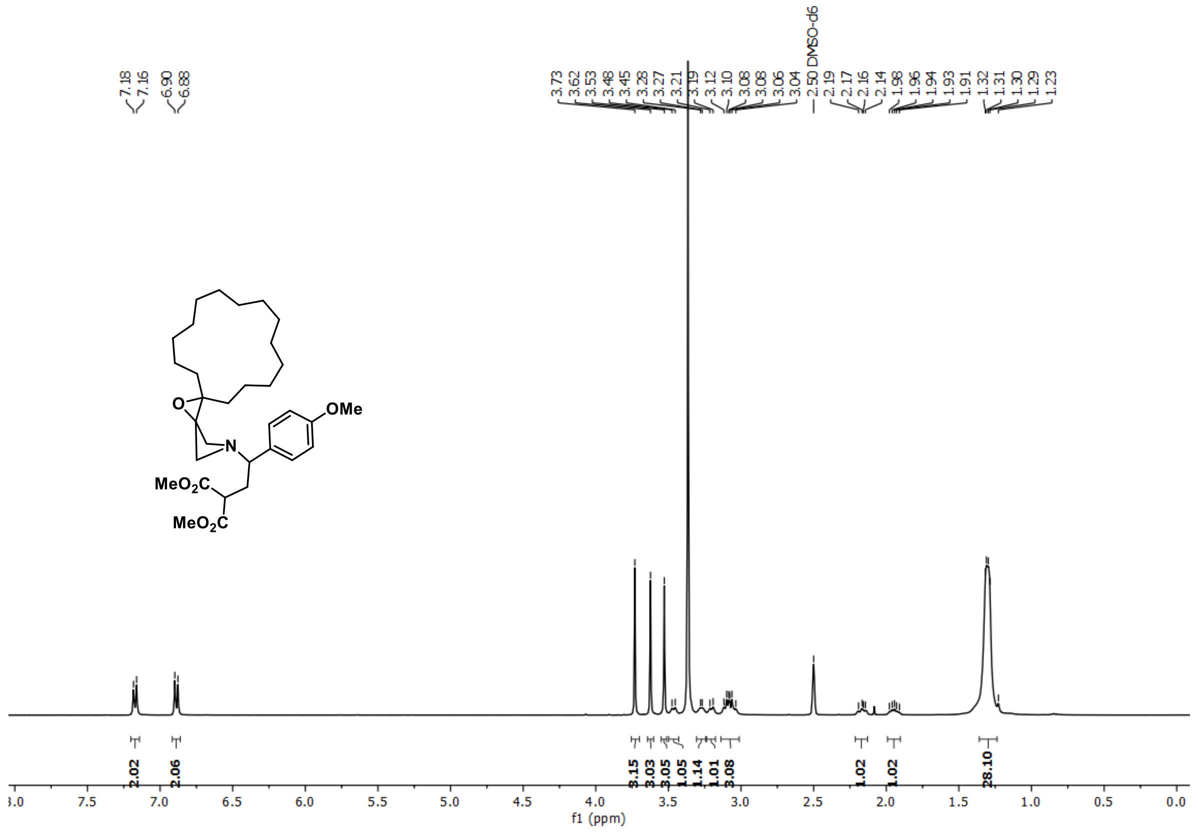

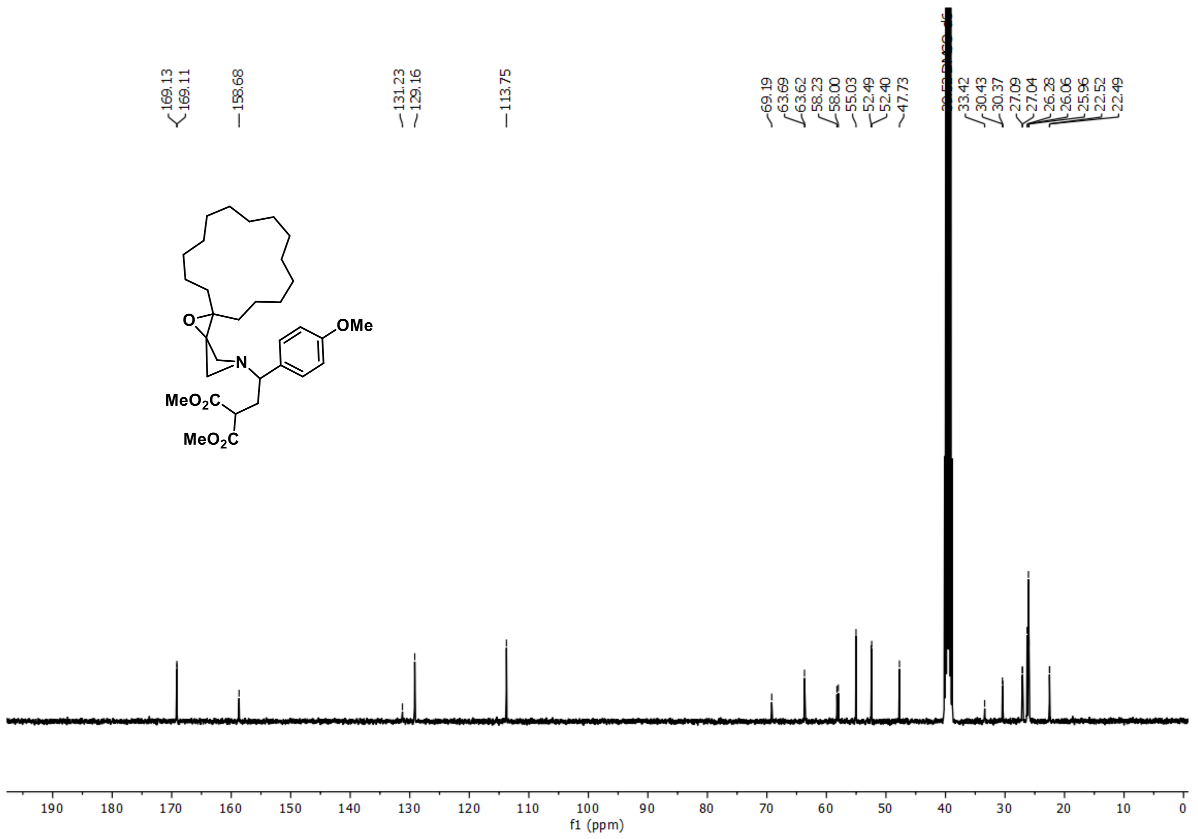

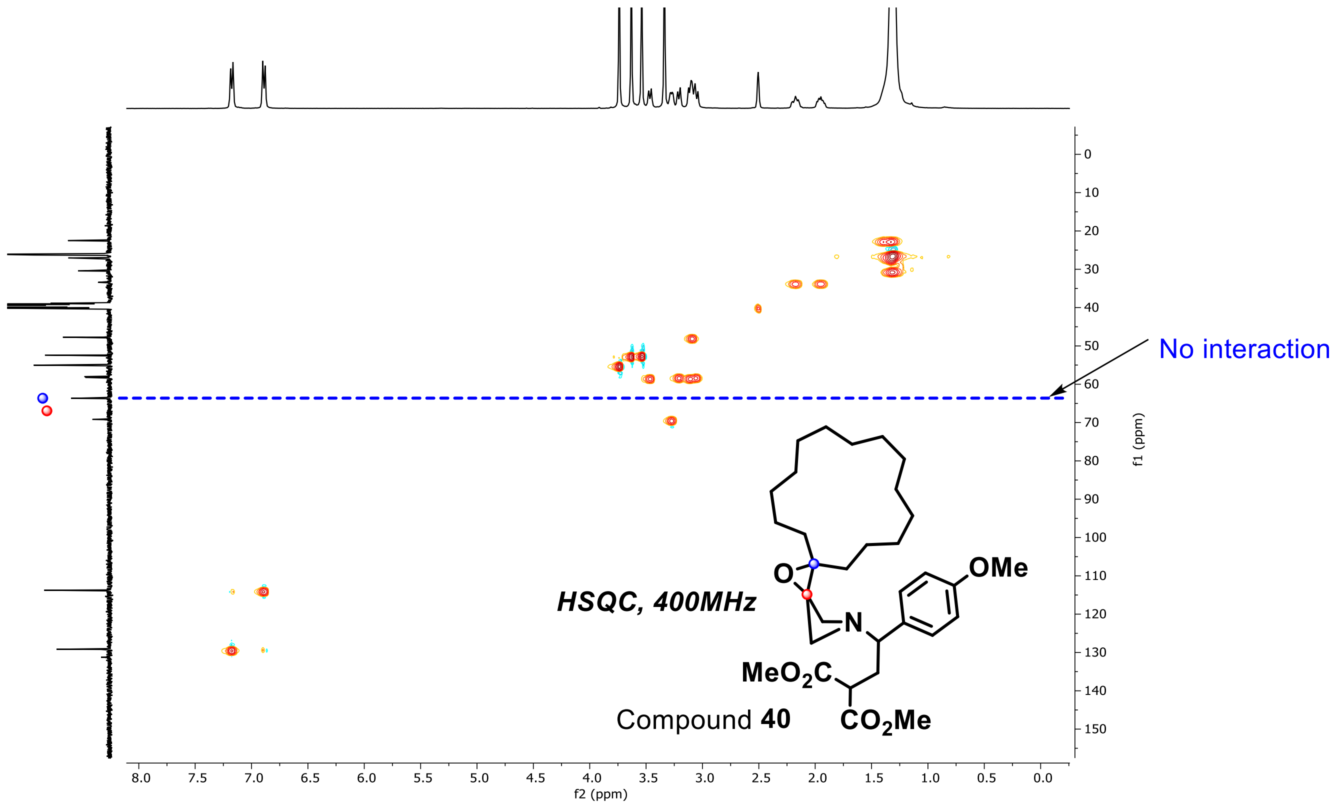
No interaction
HSQC, 400MHz
Compound 40
OMe
MeO2C
CO2Me
O
N
f1 (ppm)
f2 (ppm)

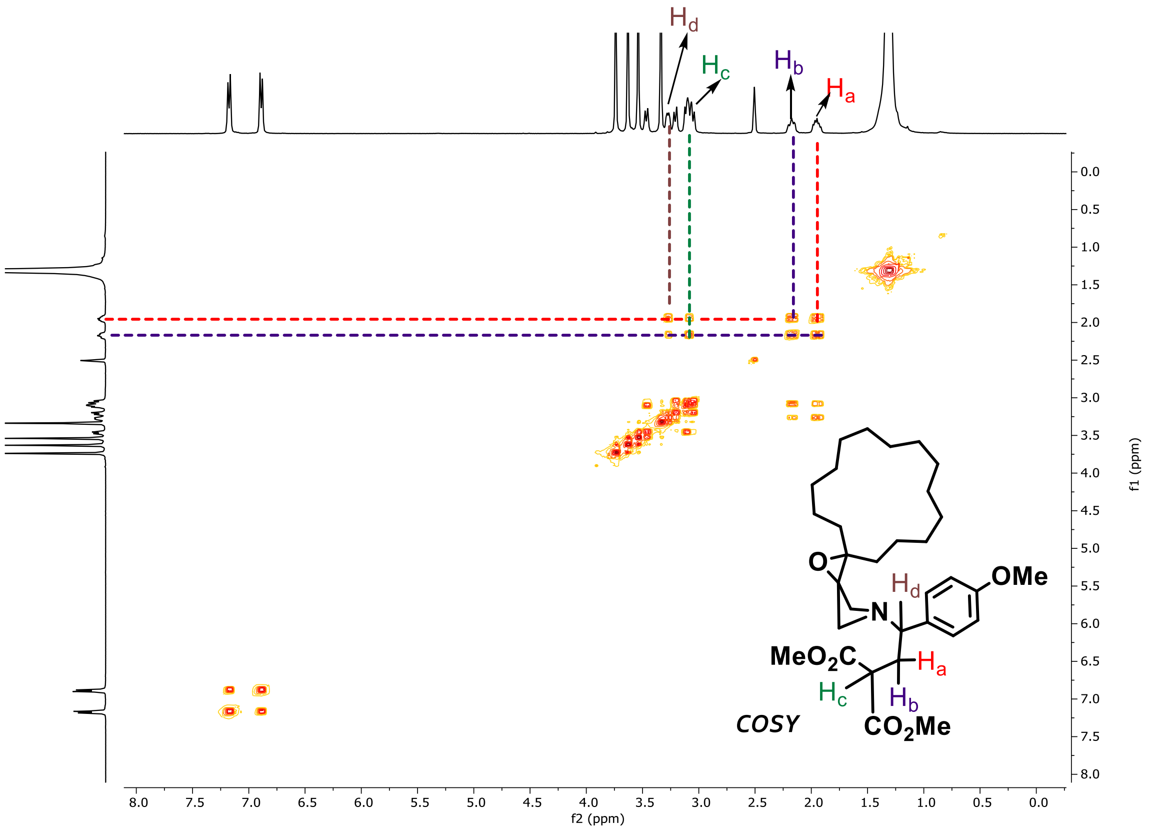
Hd
Hc
Hb
Ha
COSY
OMe
MeO2C
CO2Me
O
N
f1 (ppm)
f2 (ppm)

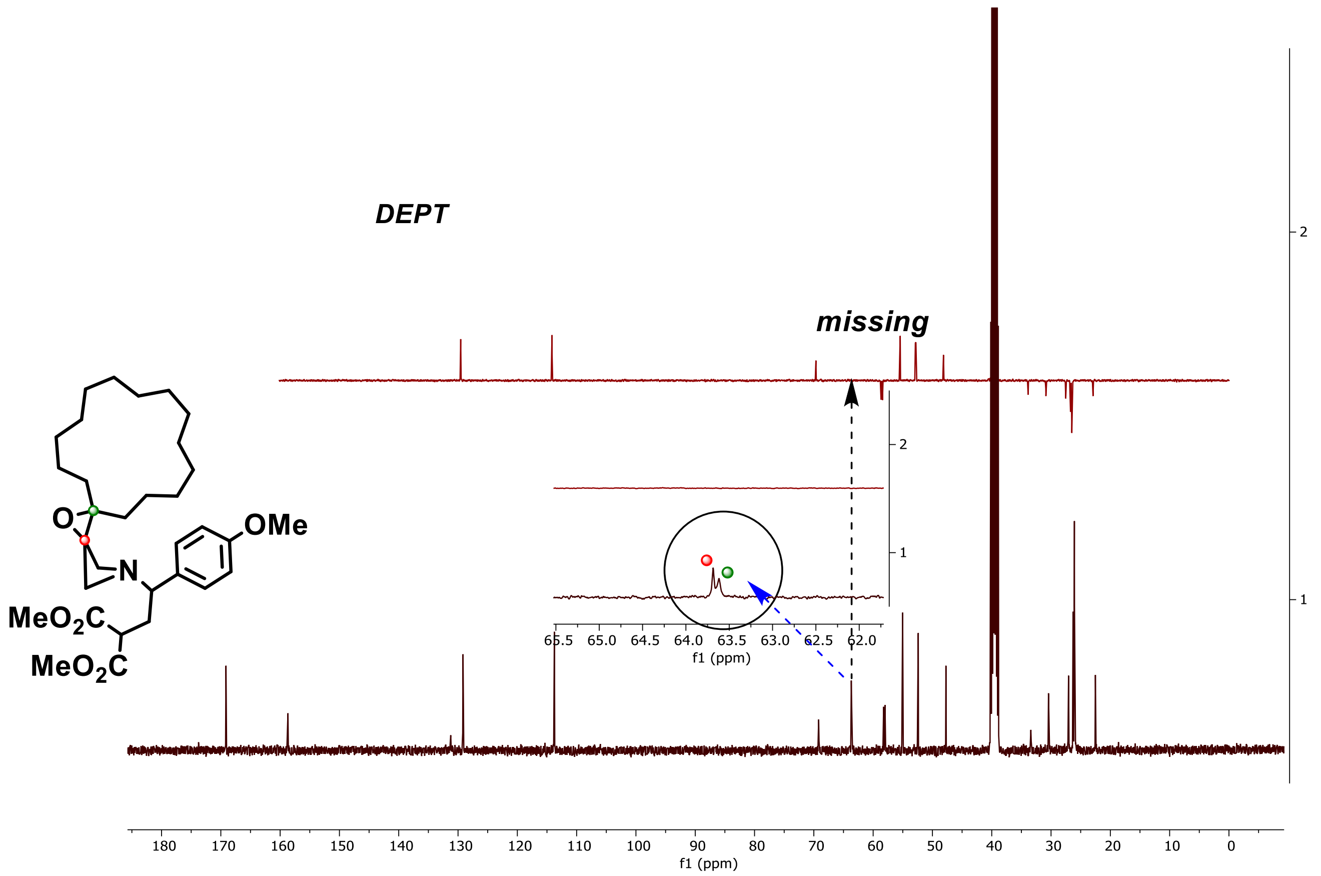
DEPT
missing
OMe
O
N
MeO2C
MeO2C
65.5 65.0 64.5 64.0 63.5 63.0 62.5 62.0
f1 (ppm)
180 170 160 150 140 130 120 110 100 90 80 70 60 50 40 30 20 10 0
f1 (ppm)

^{1}H spectra at 400 MHz and ^{13}C{^{1}H} NMR spectra at 100 MHz in DMSO- d6 (**41**)

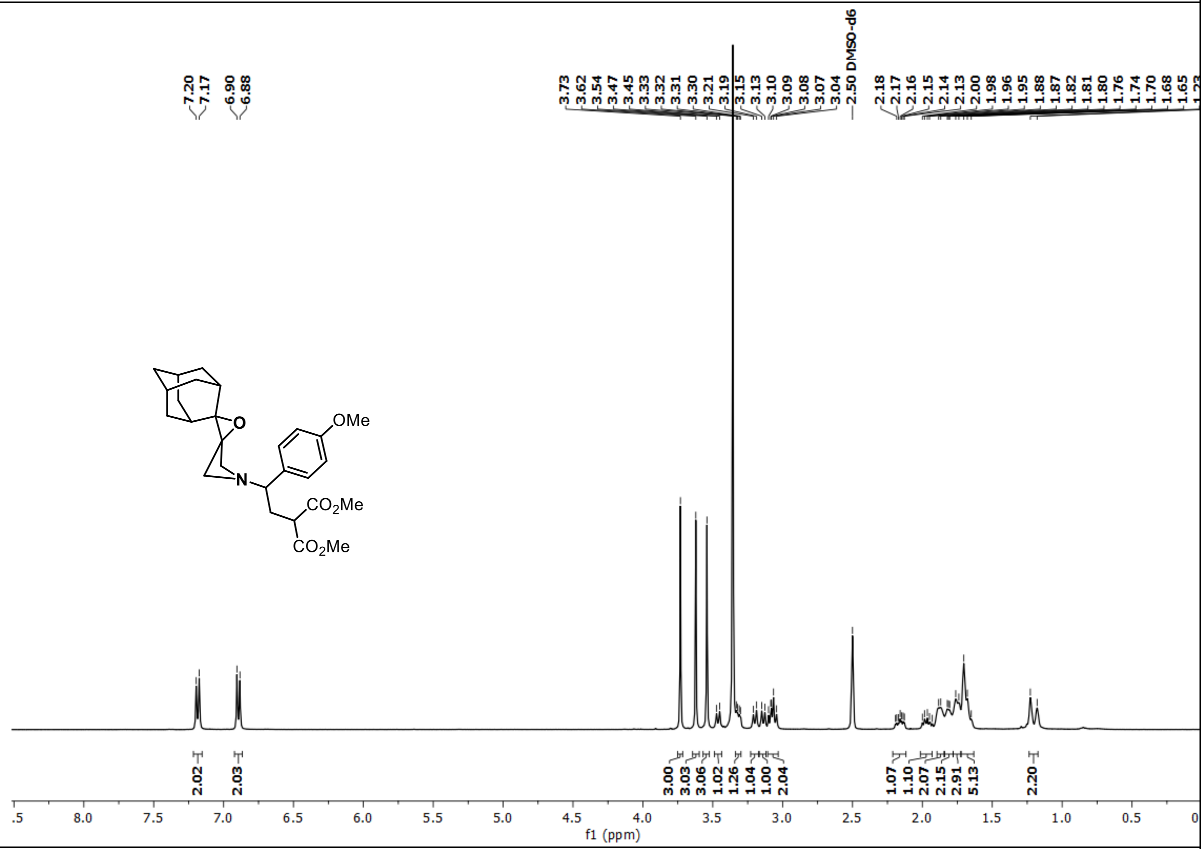

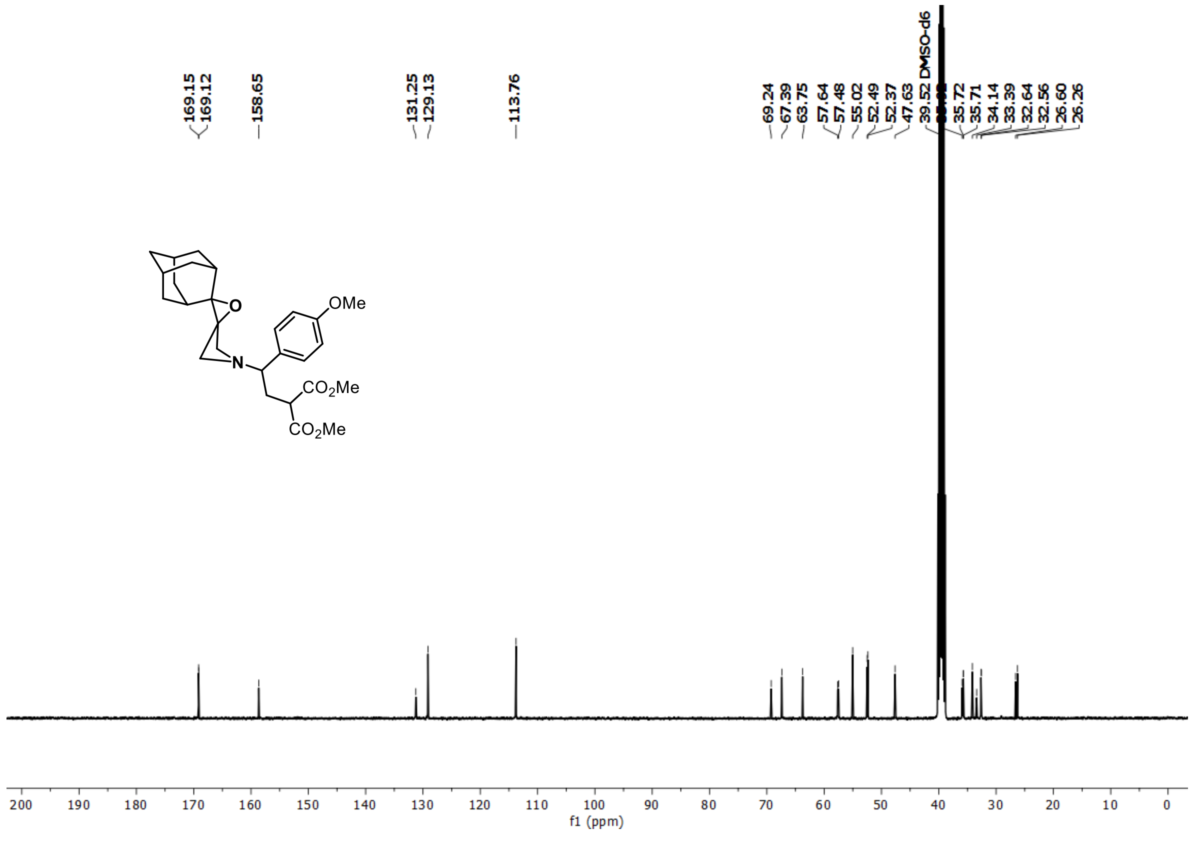

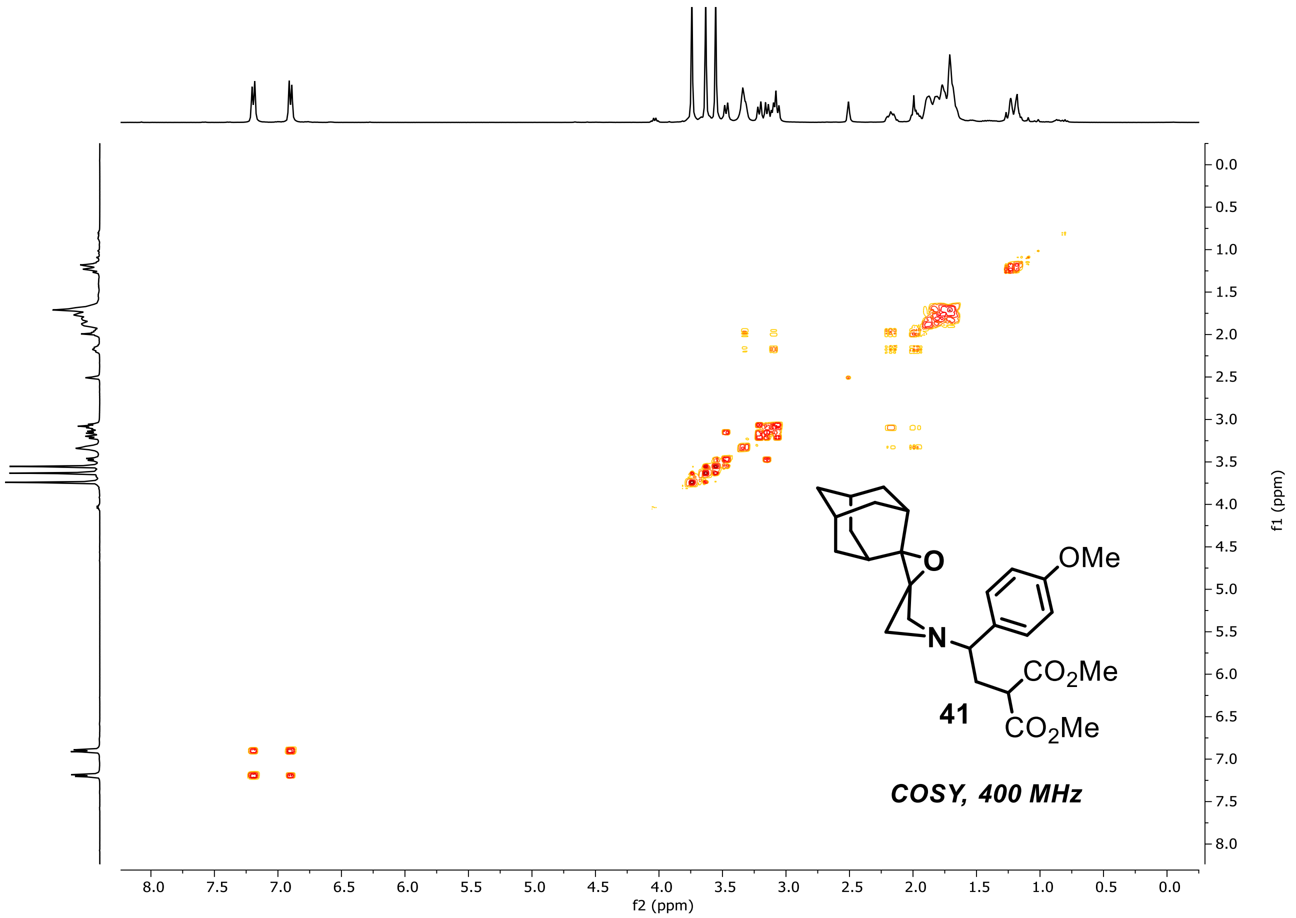
OMe
CO_2Me
41
CO_2Me
COSY, 400 MHz
f2 (ppm)
f1 (ppm)

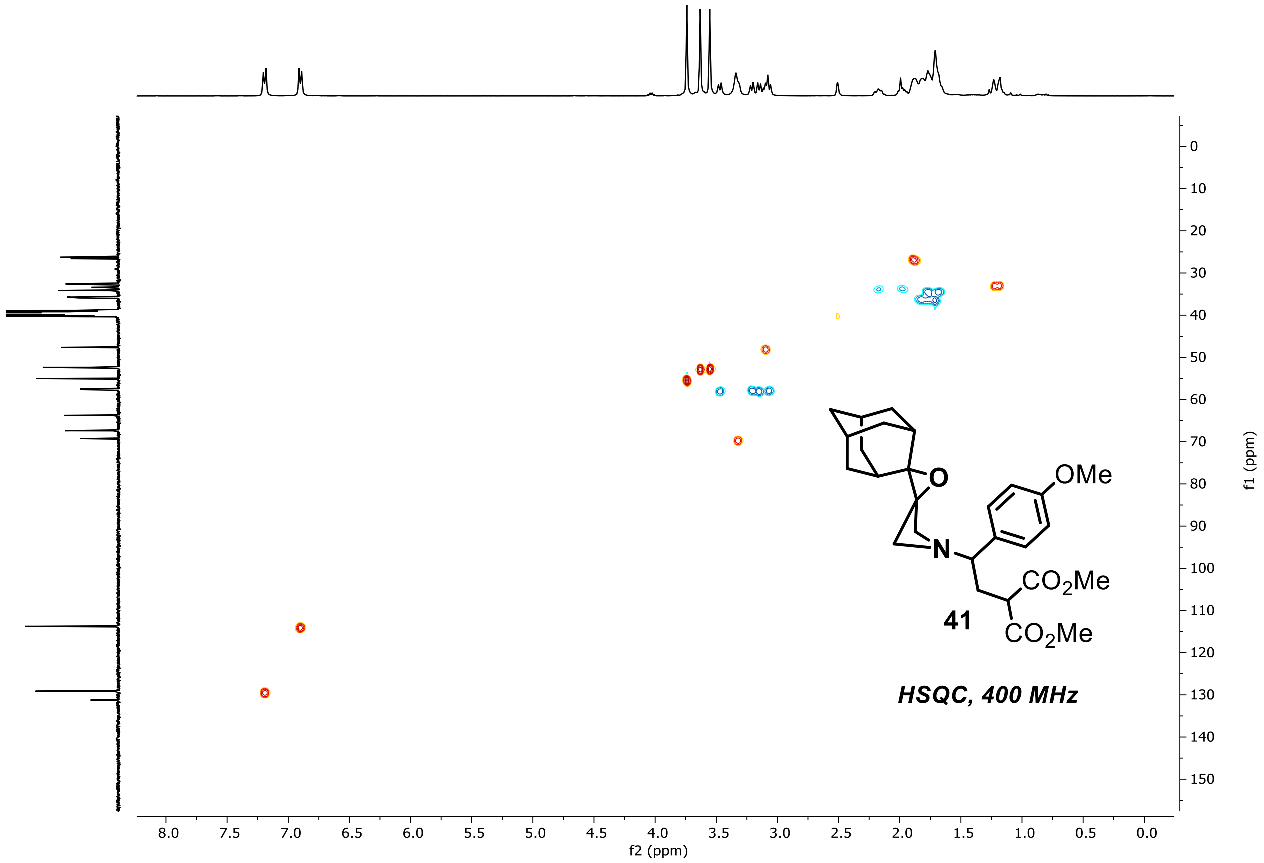
OMe
CO_2Me
41
CO_2Me
HSQC, 400 MHz
f2 (ppm)
f1 (ppm)

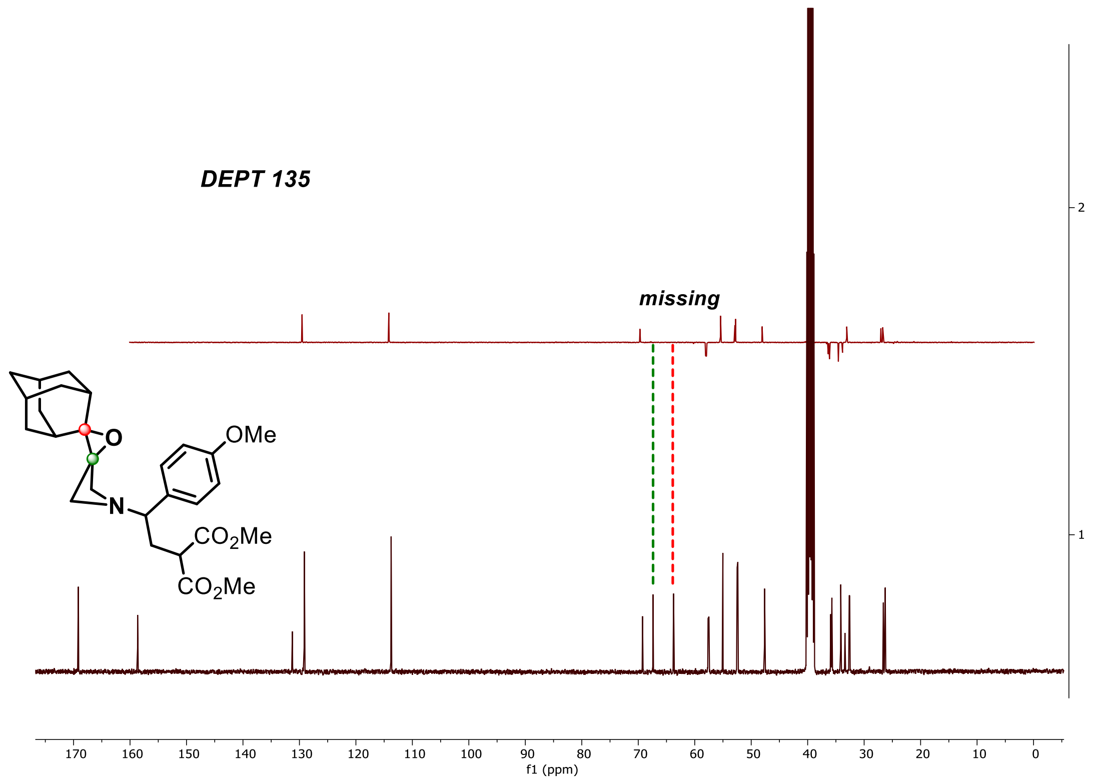
DEPT 135
missing
OMe
N
O
CO2Me
CO2Me
f1 (ppm)

^{1}H spectra at 400 MHz and ^{13}C{^{1}H} NMR spectra at 100 MHz in DMSO- d6 (**42**)

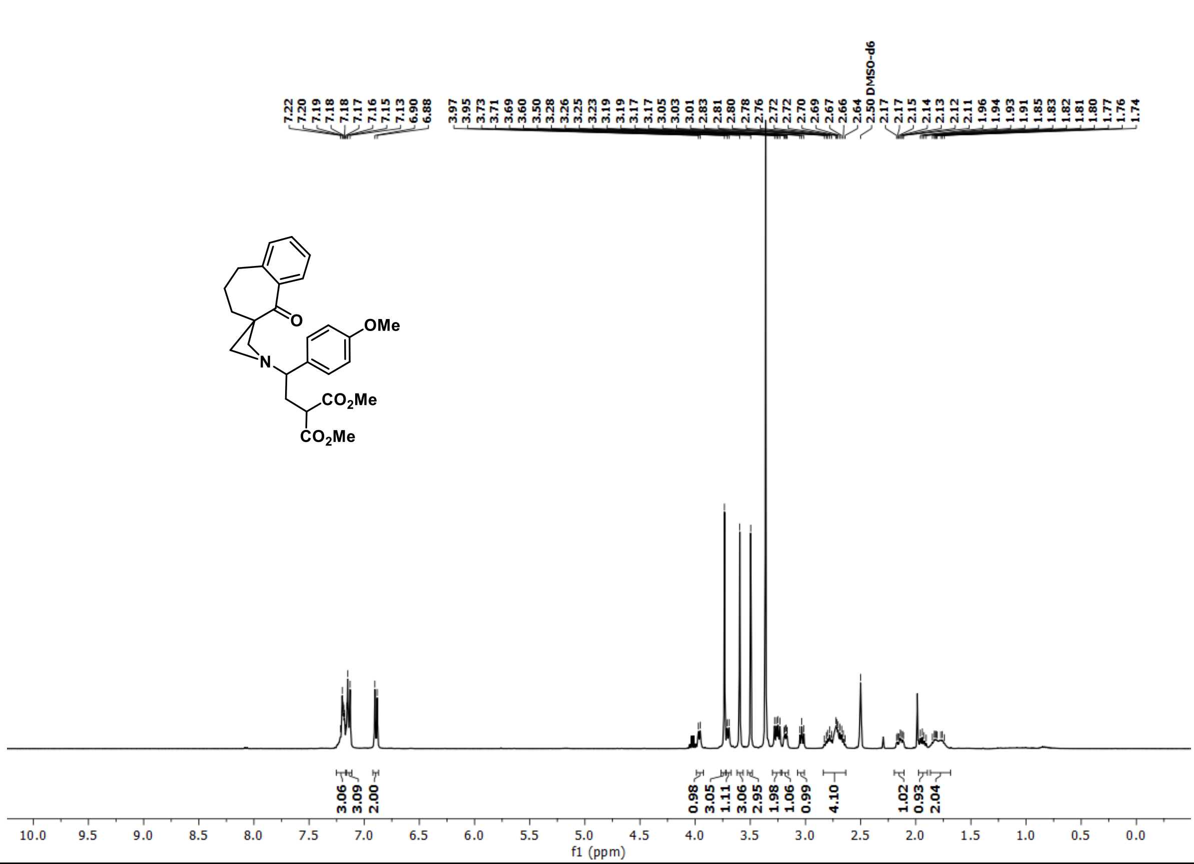

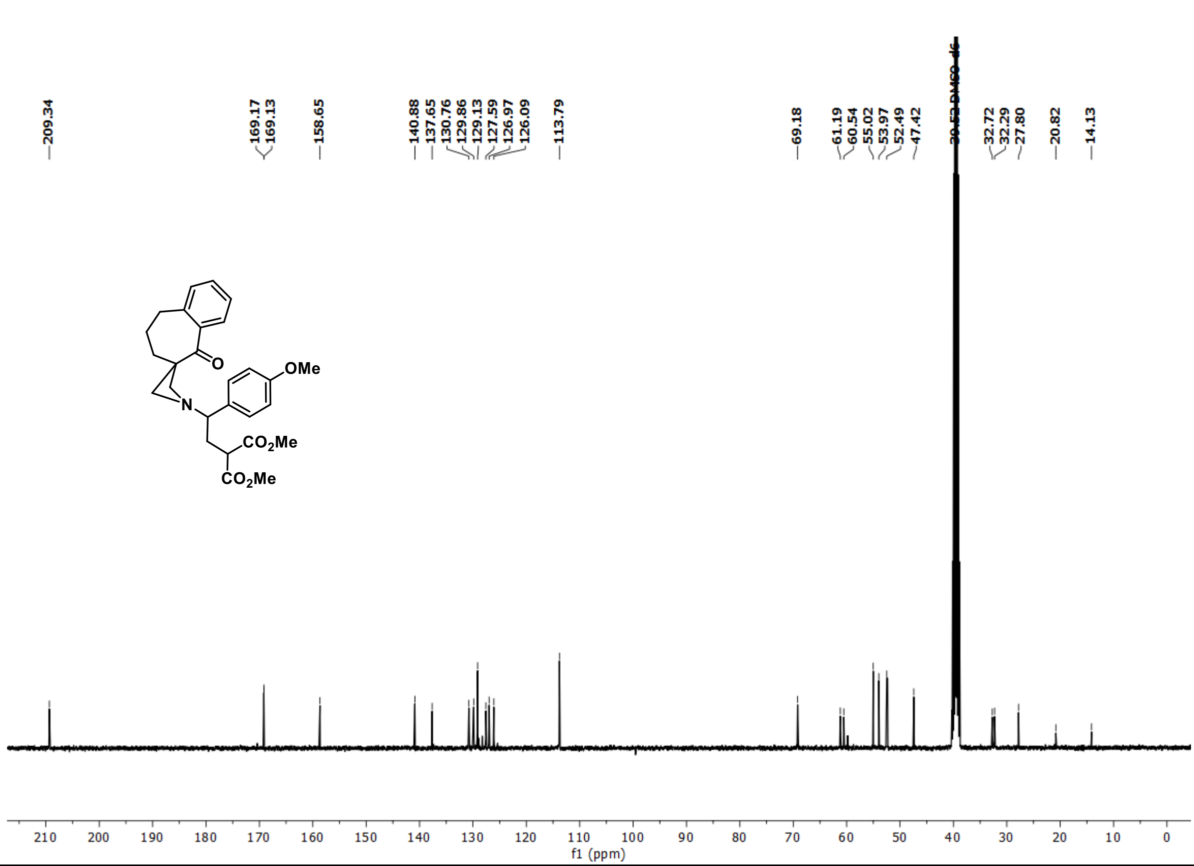

^{1}H spectra at 400 MHz and ^{13}C{^{1}H} NMR spectra at 100 MHz in DMSO- d6 (**43**)

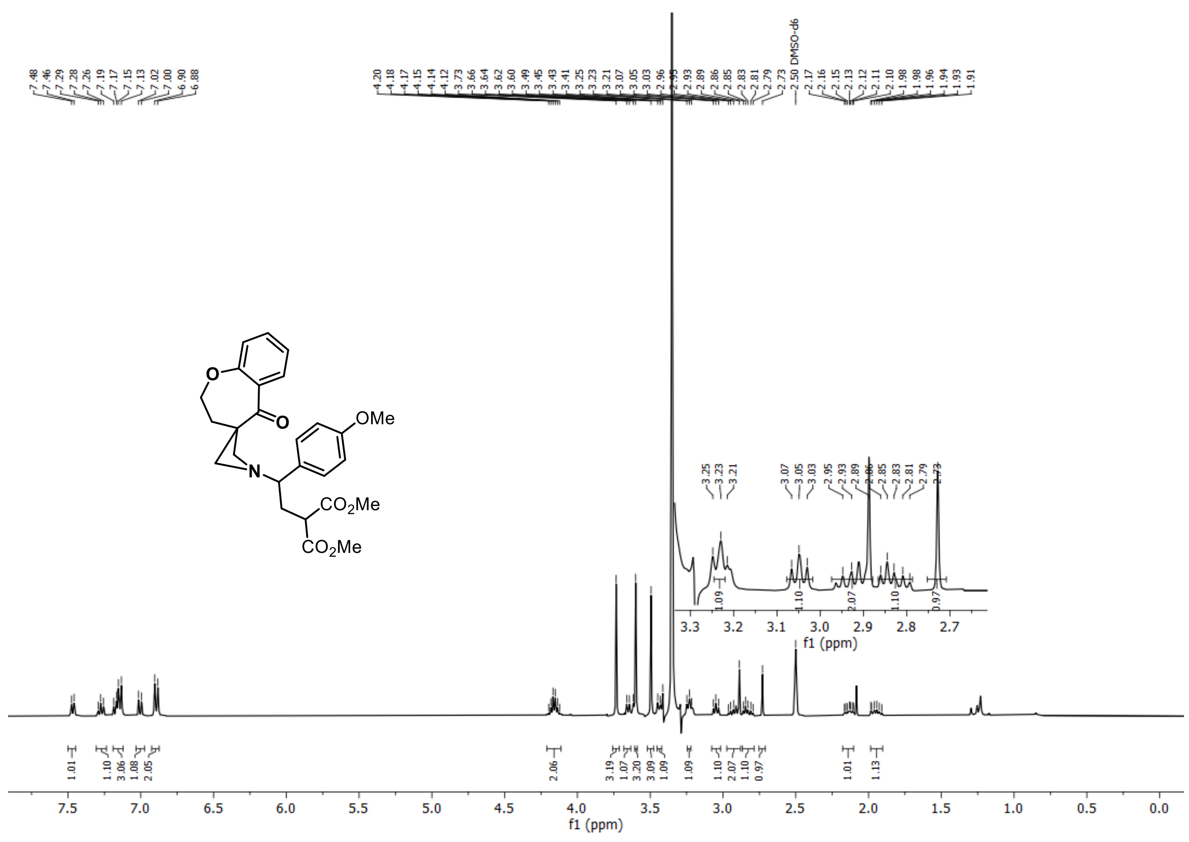

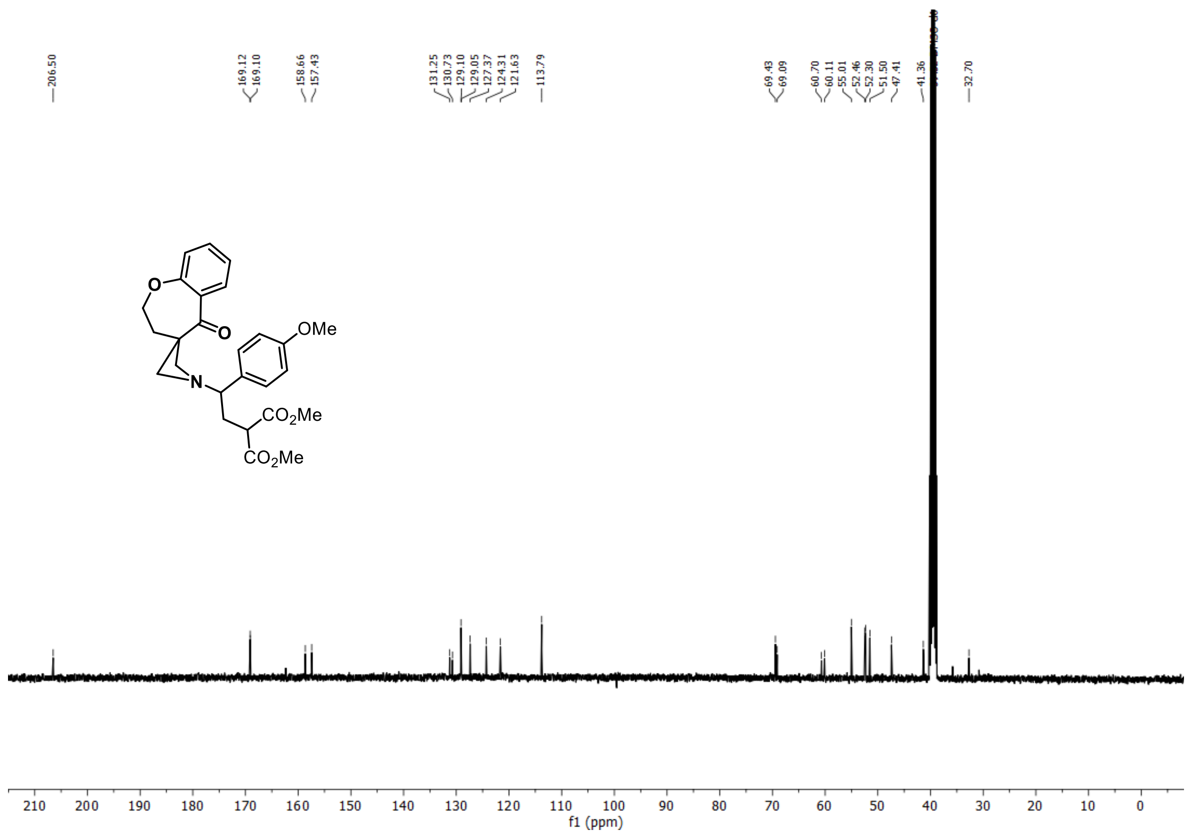

^{1}H spectra at 400 MHz and ^{13}C{^{1}H} NMR spectra at 100 MHz in DMSO- d6 (**44**)

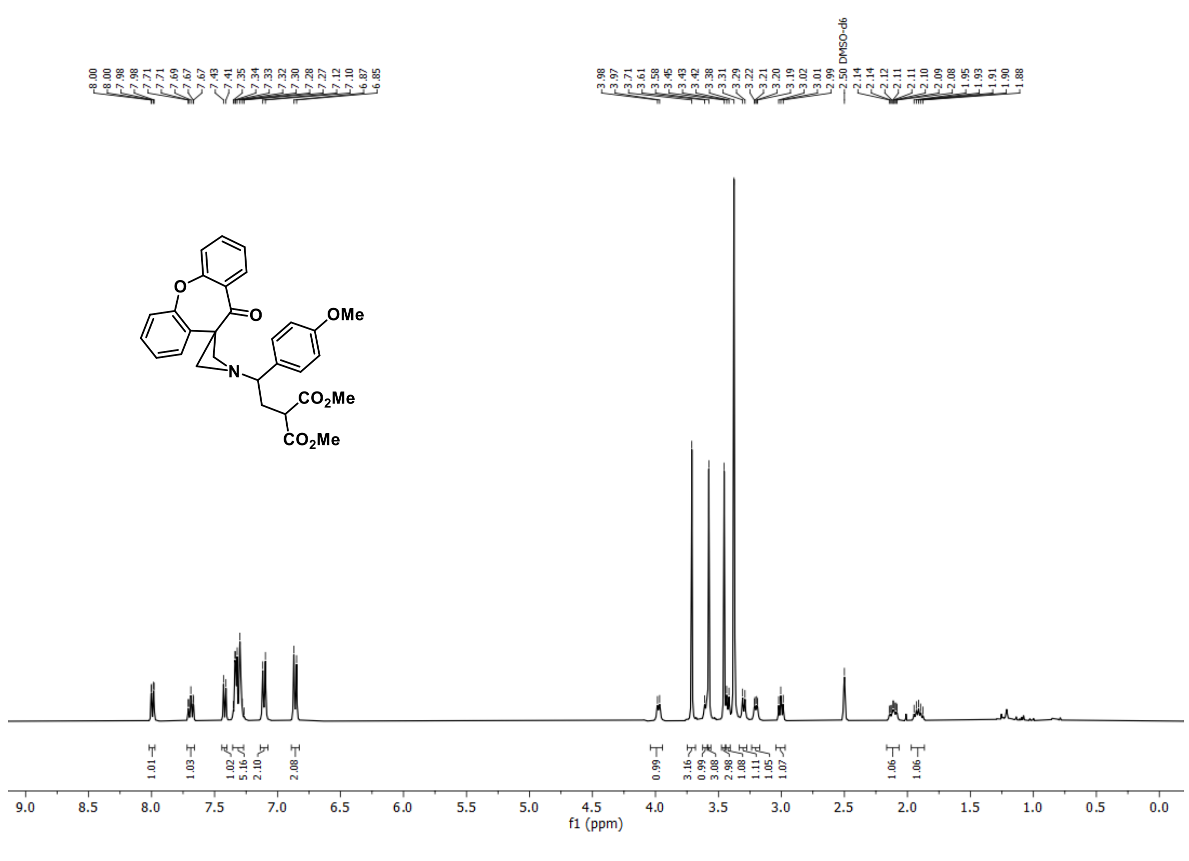

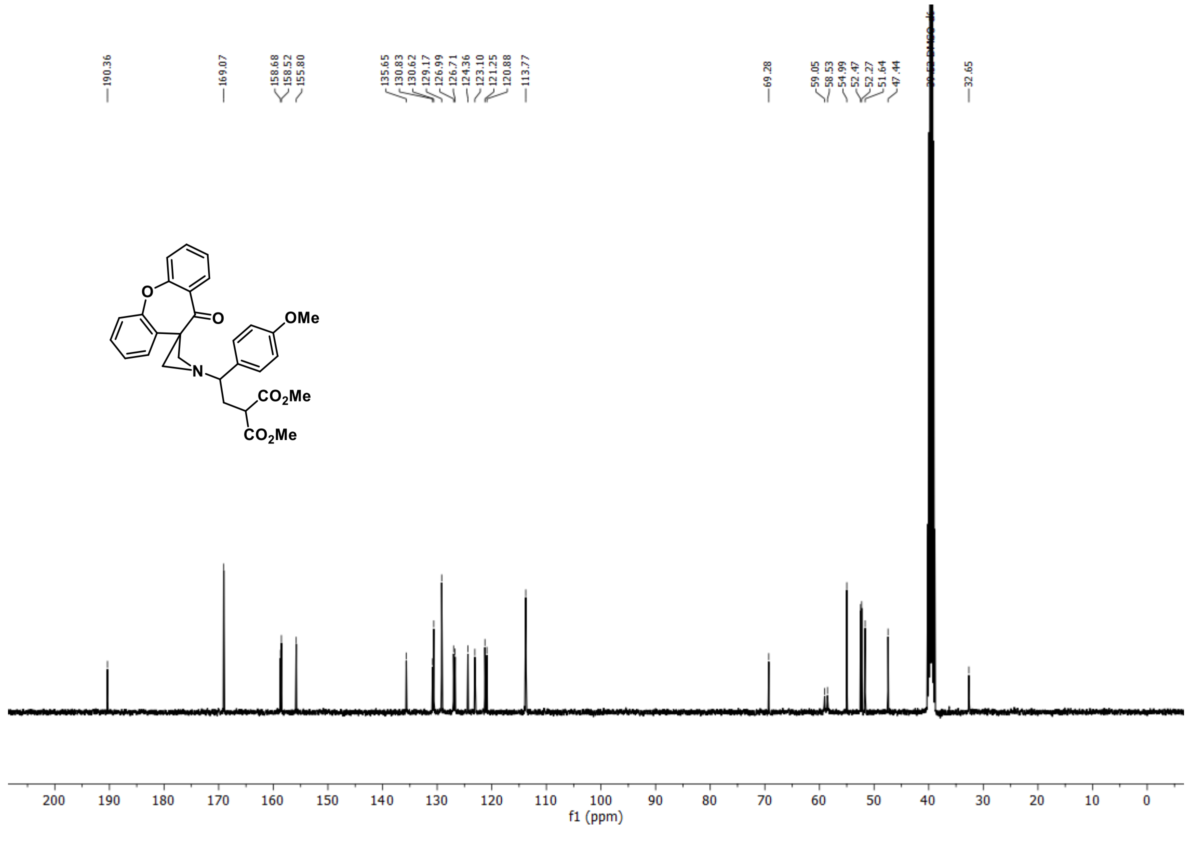

^{1}H spectra at 600 MHz and $^{13}C\{^{1}H\}$ NMR spectra at 150 MHz in DMSO-d6 (**45**)

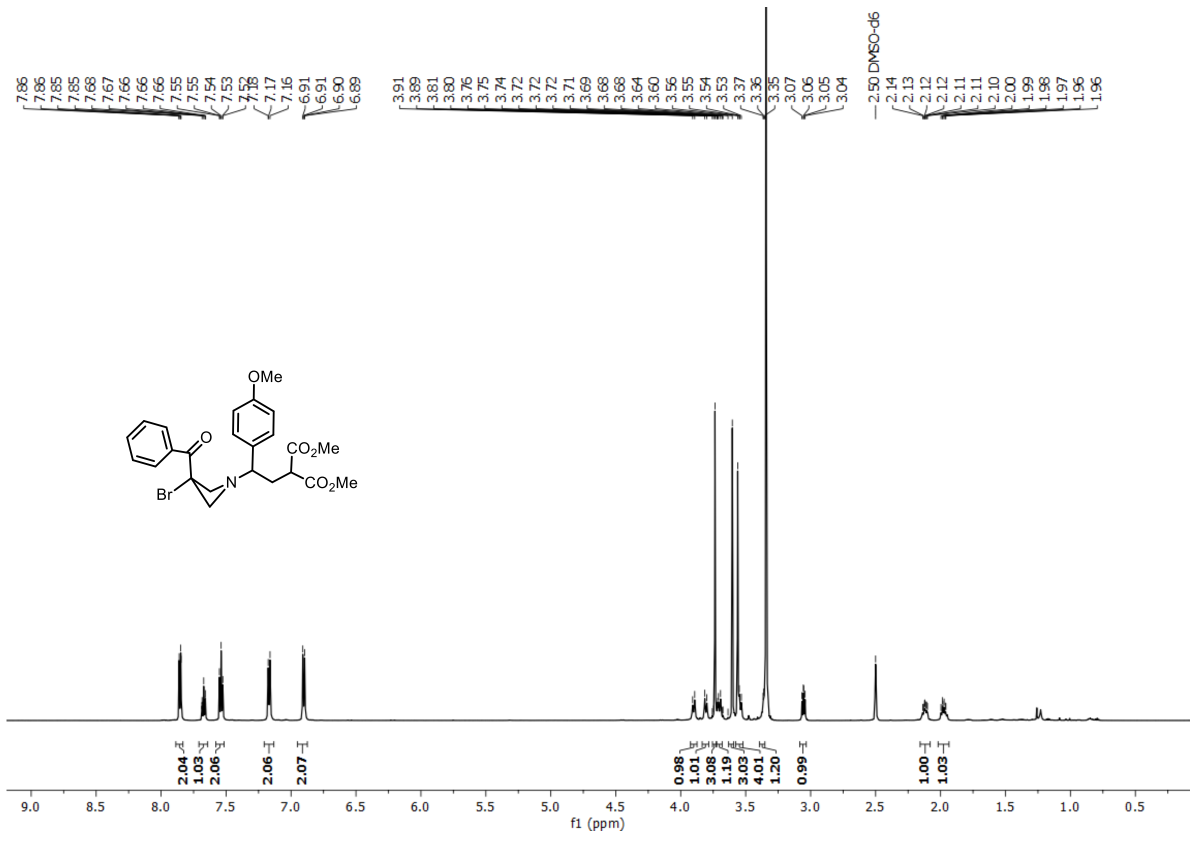

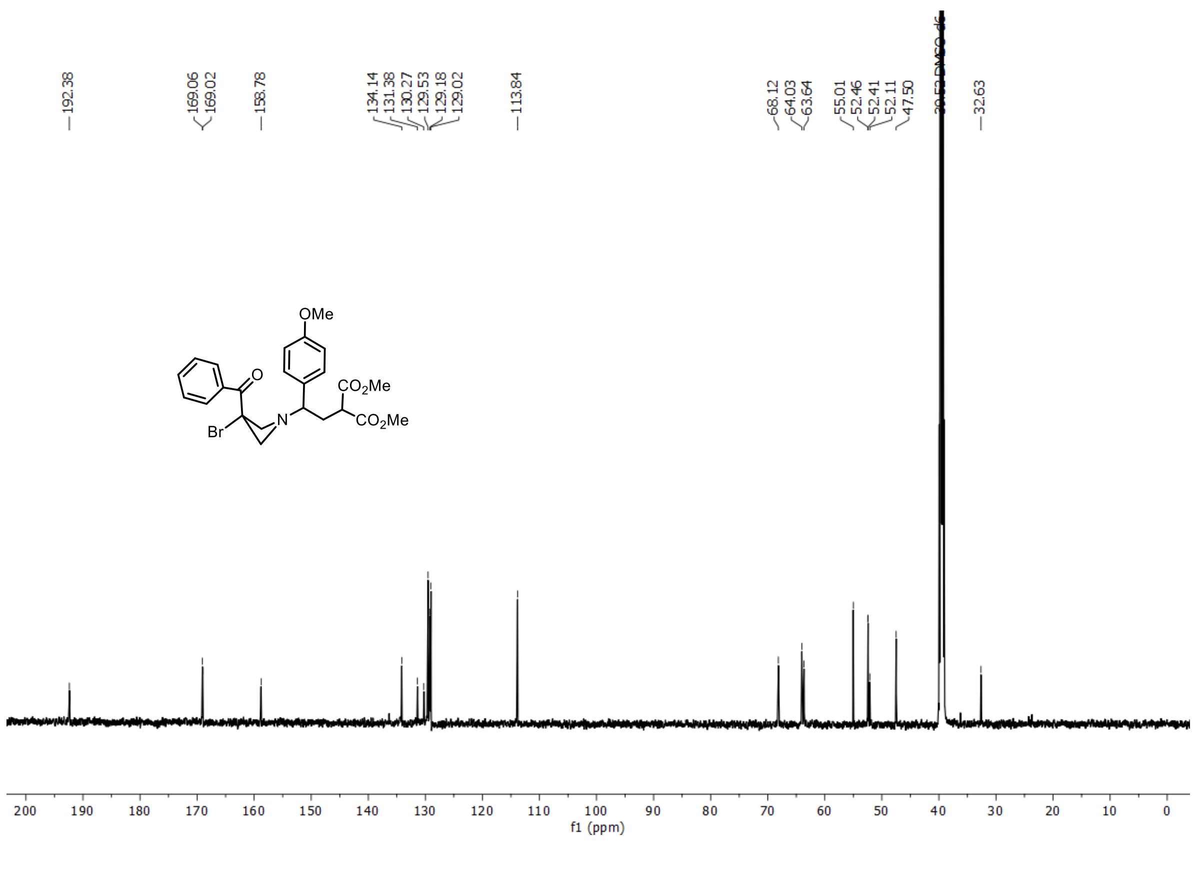

^{1}H spectra at 600 MHz and ^{13}C{^{1}H} NMR spectra at 150 MHz in DMSO-d6 (**46**)

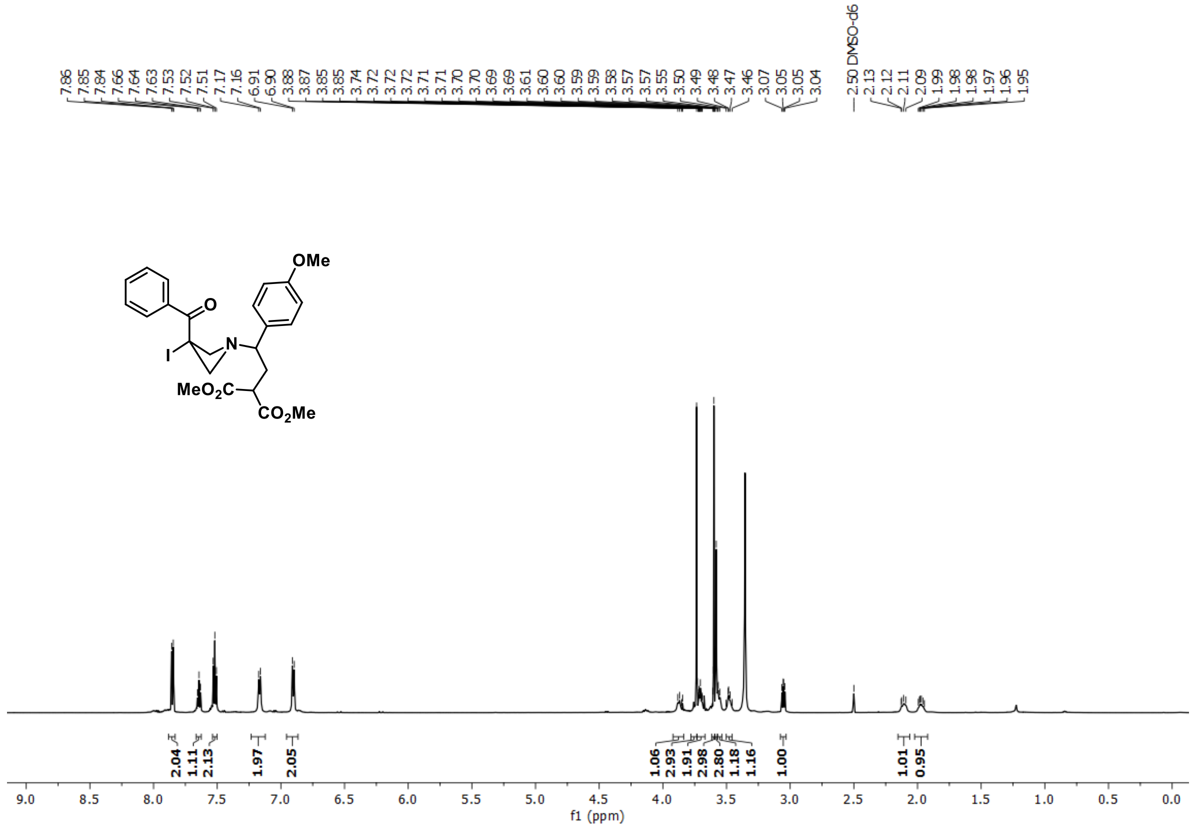

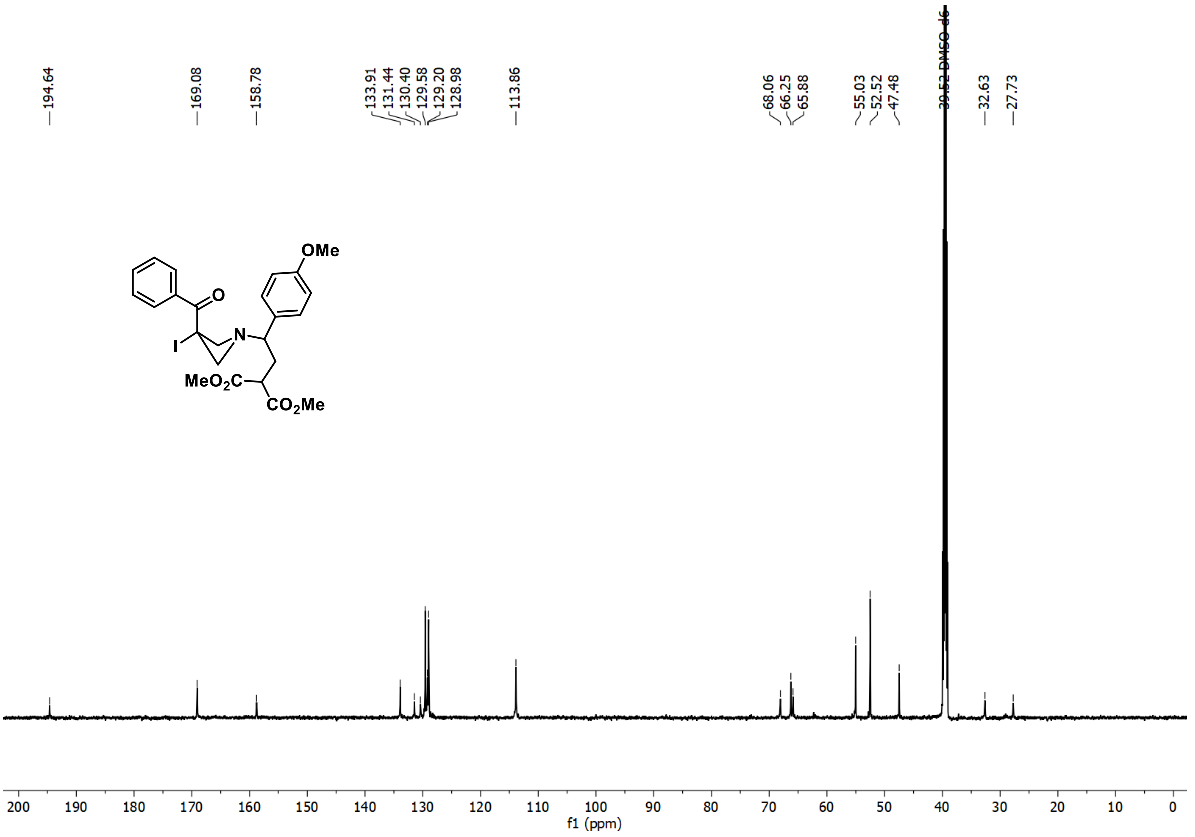

^1H spectra at 600 MHz and $^{13}C\{^1H\}$ NMR spectra at 150 MHz in DMSO-d6 (**47**)

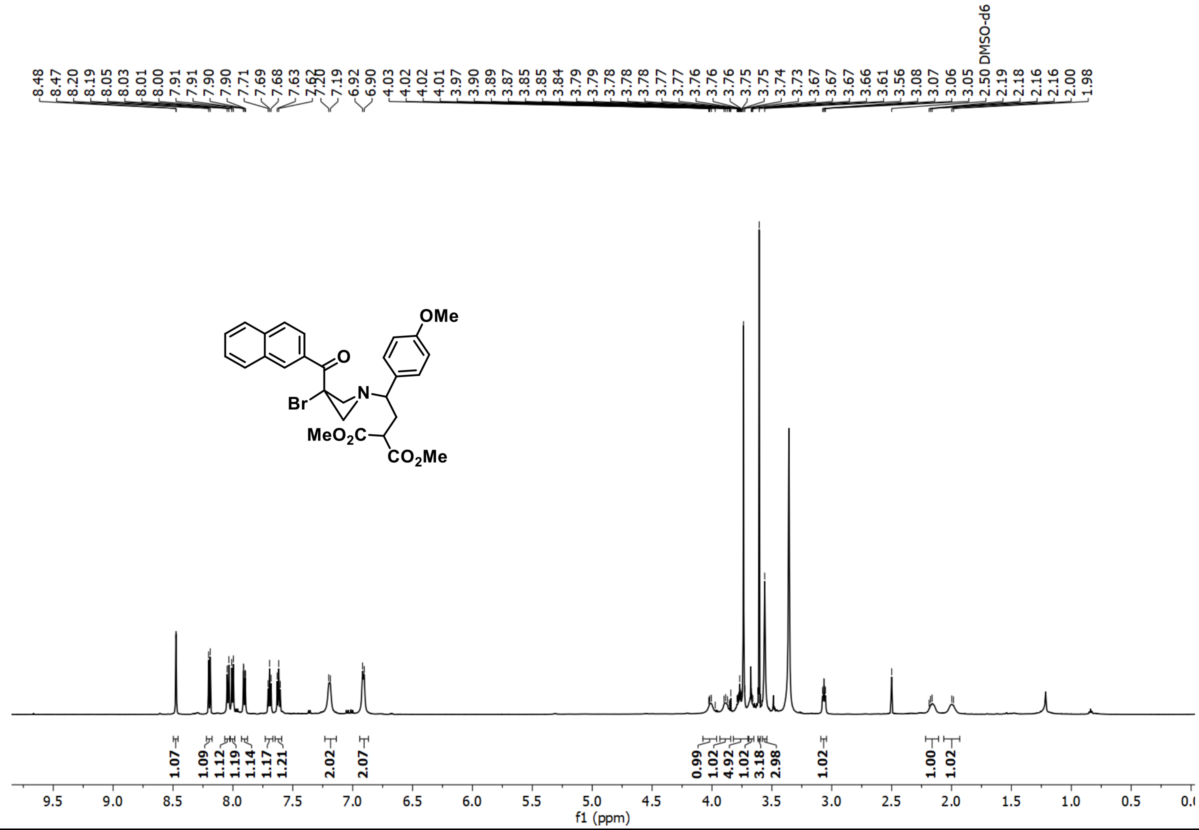

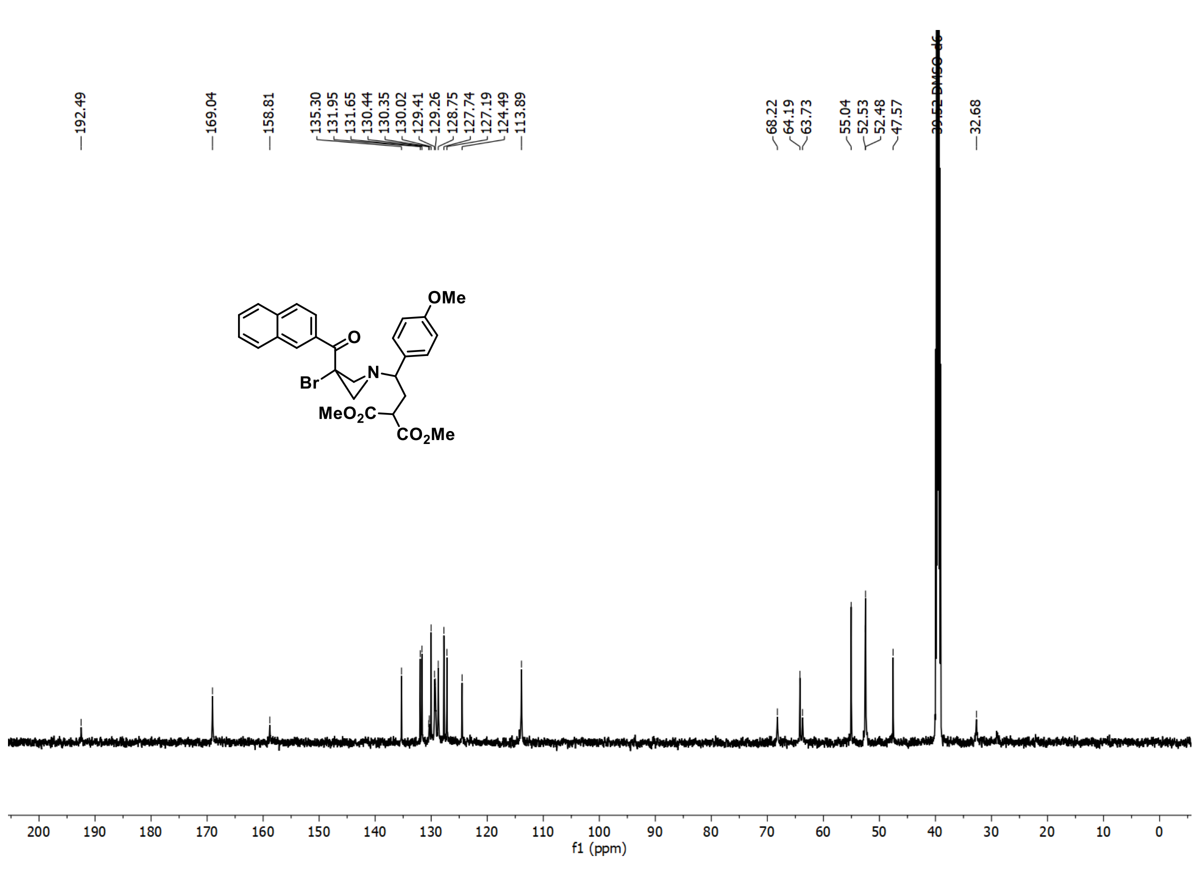

^{1}H spectra at 600 MHz and ^{13}C{^{1}H} NMR spectra at 150 MHz in DMSO-d6 (**48**)

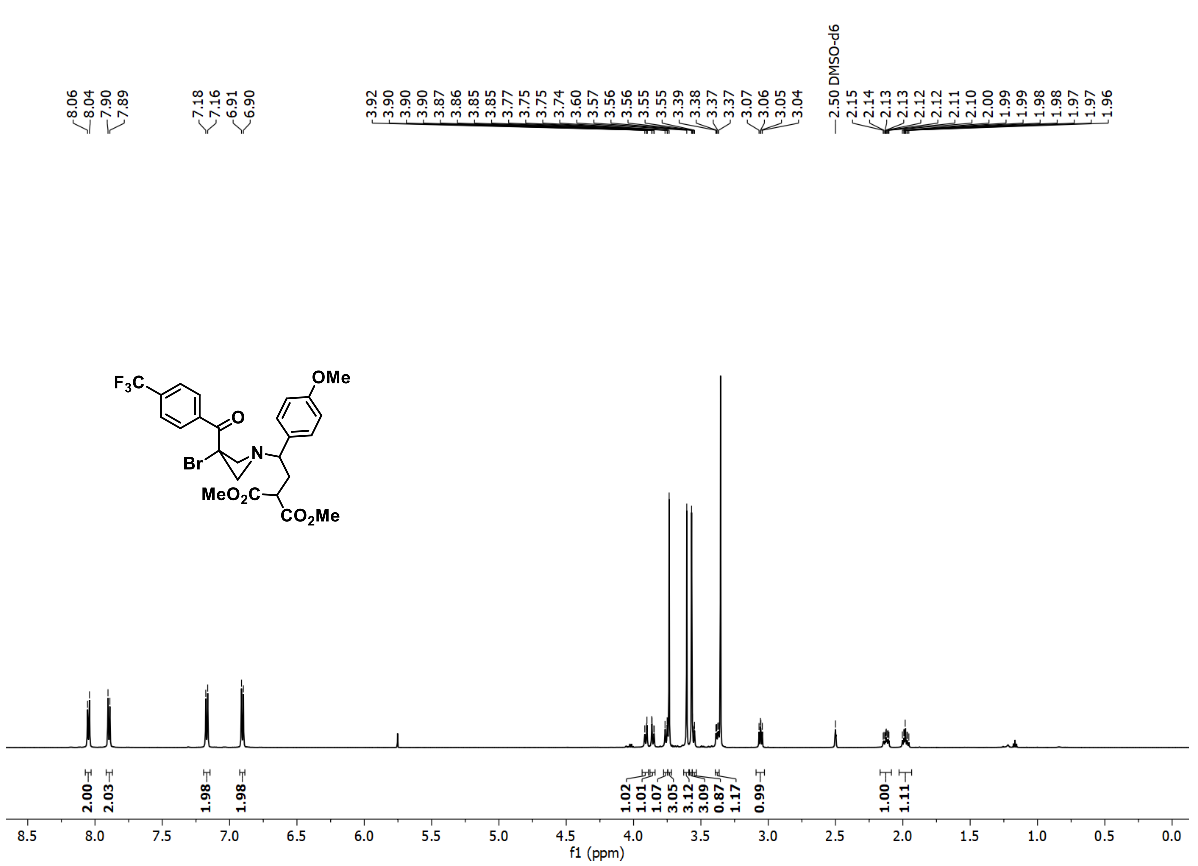

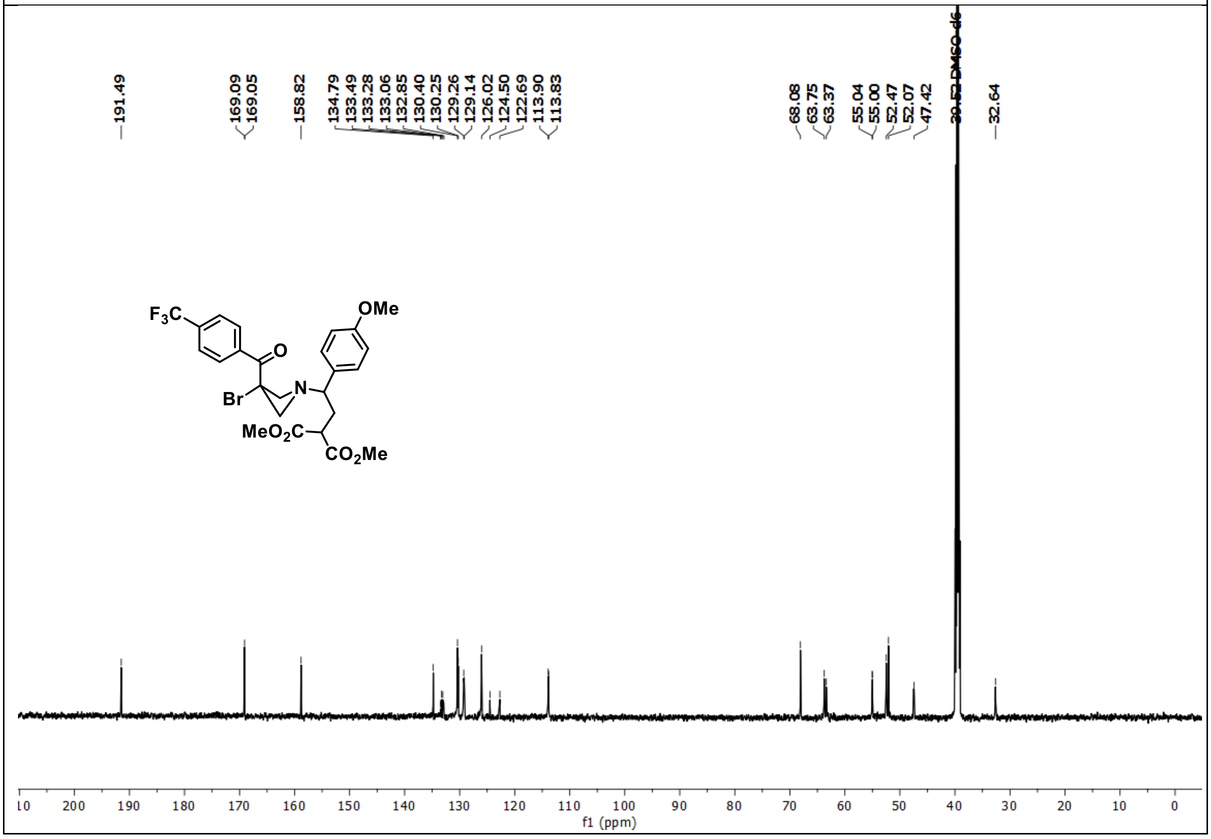

^{19}**F** spectra at 564 MHz DMSO-d6 (**48**)

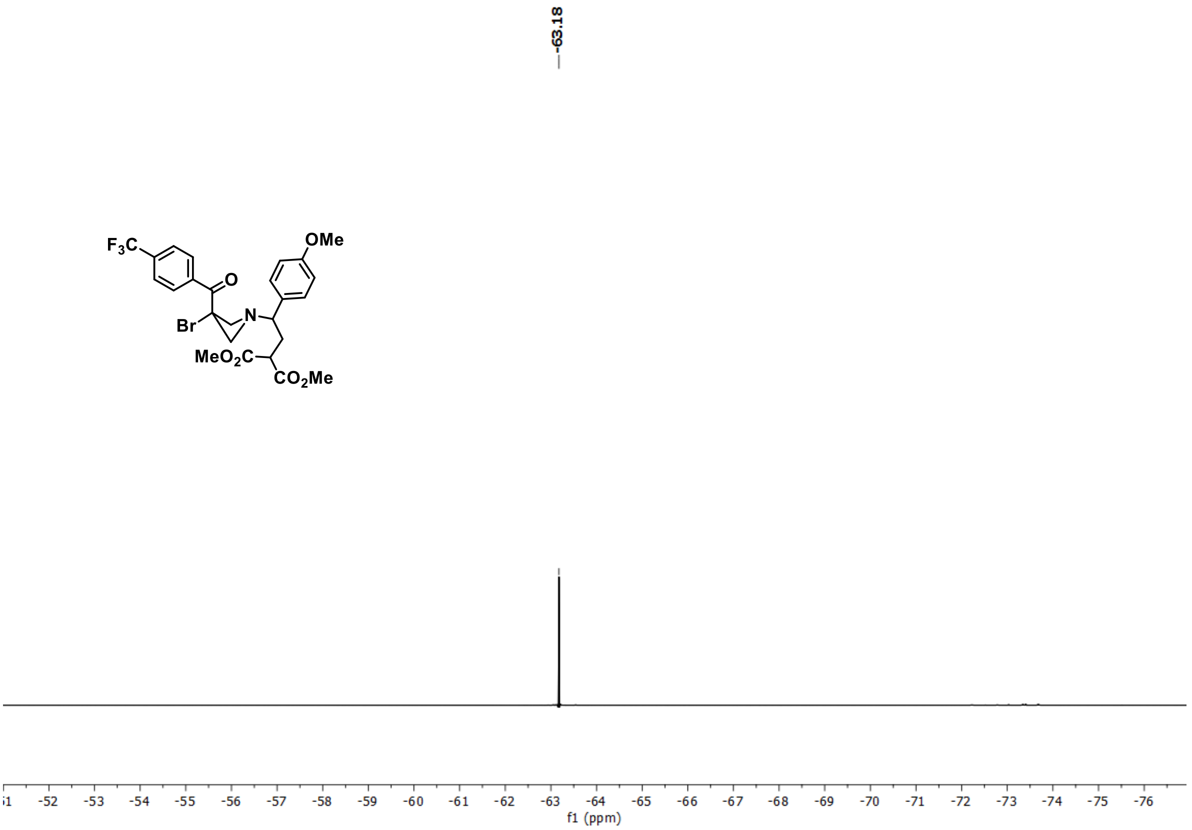

^{1}H spectra at 400 MHz and ^{13}C{^{1}H} NMR spectra at 100 MHz in DMSO-d6 (**49**)

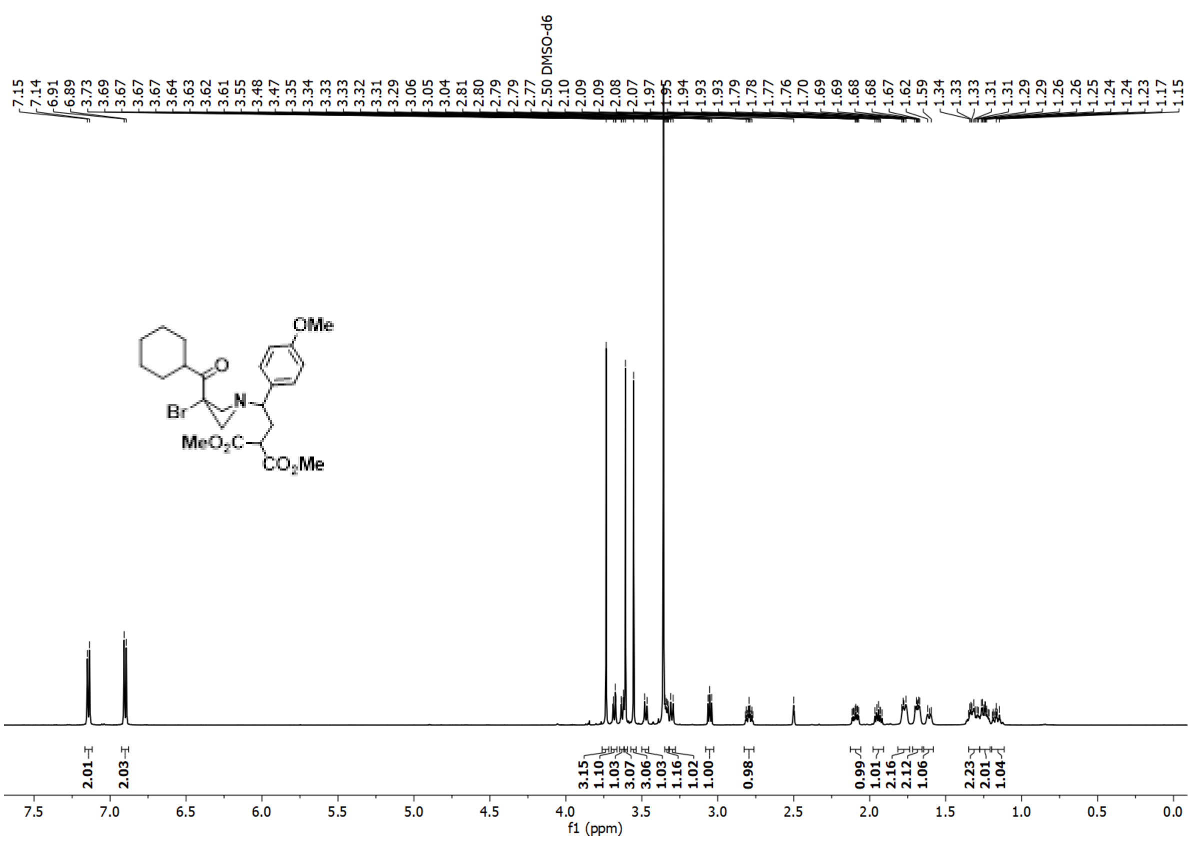

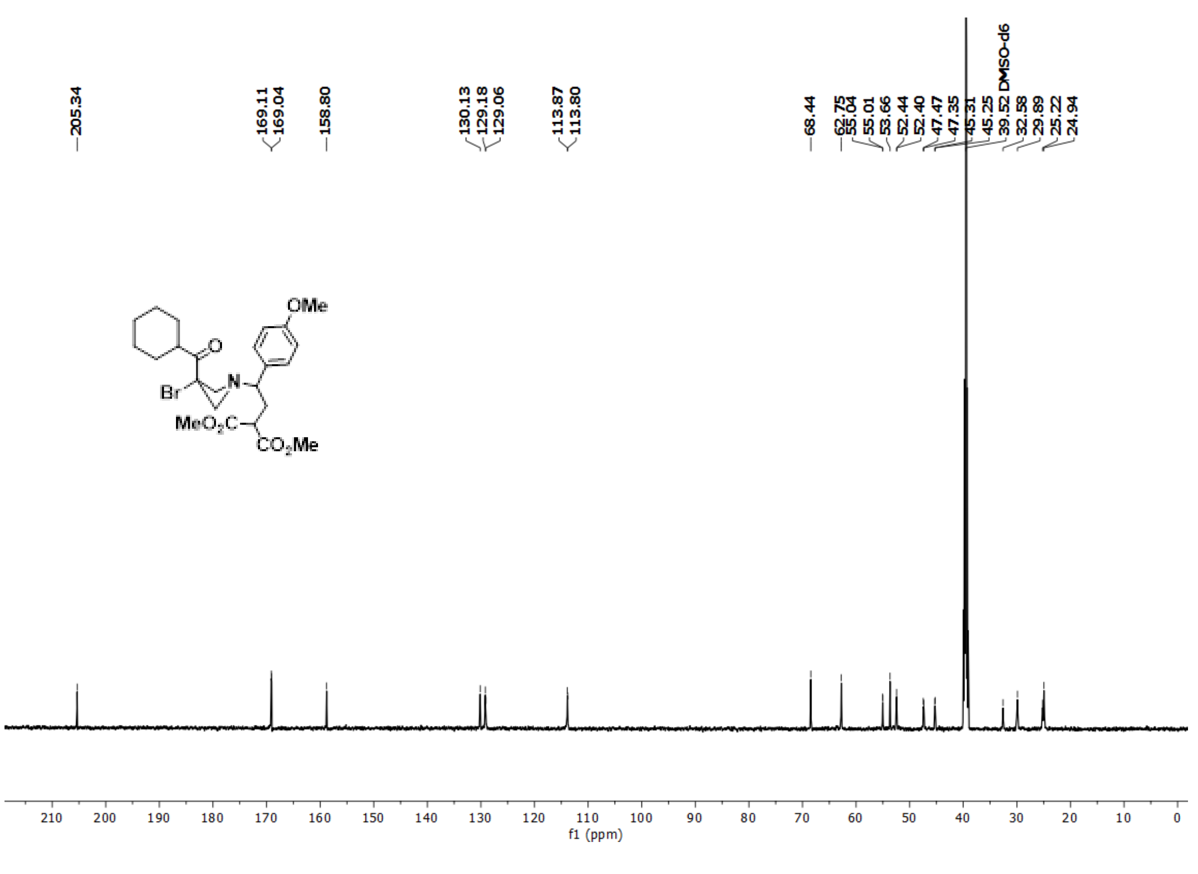

^{1}H spectra at 600 MHz and ^{13}C{^{1}H} NMR spectra at 150 MHz in CDCl3 (**52**)

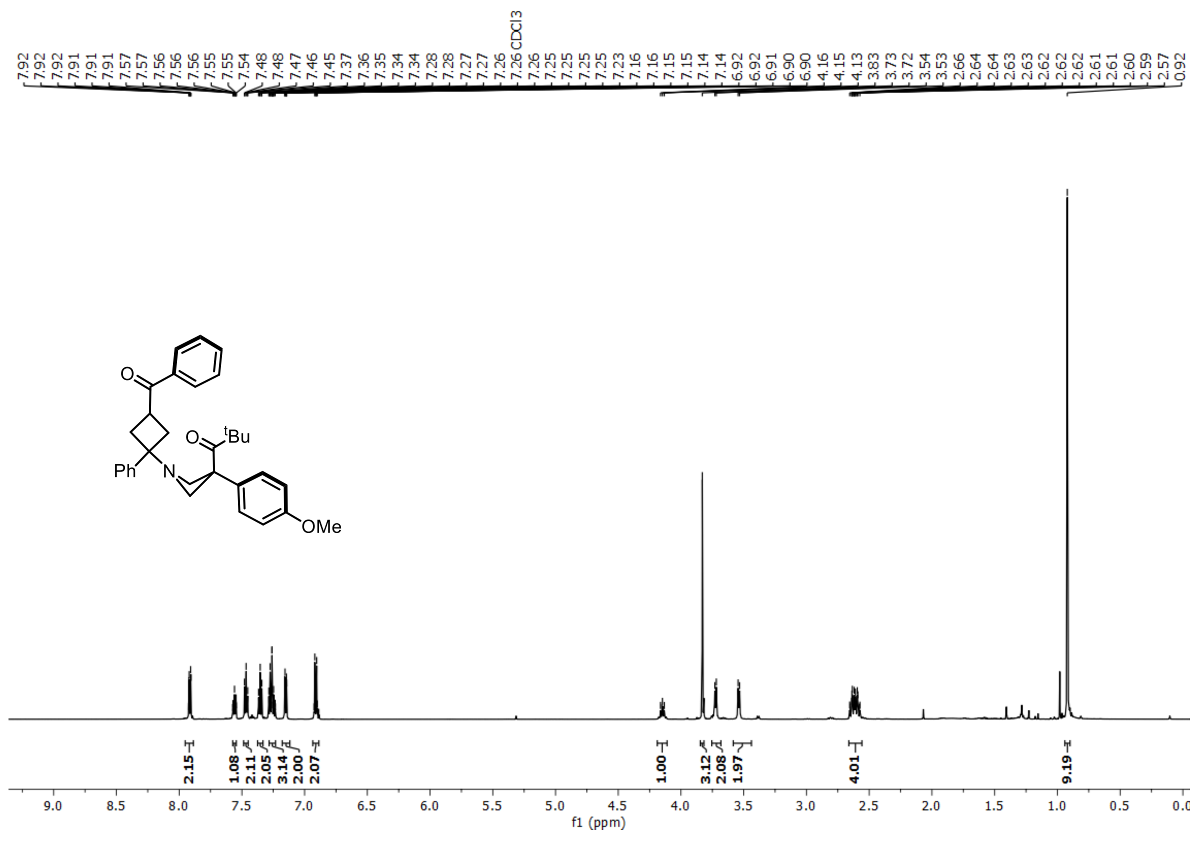

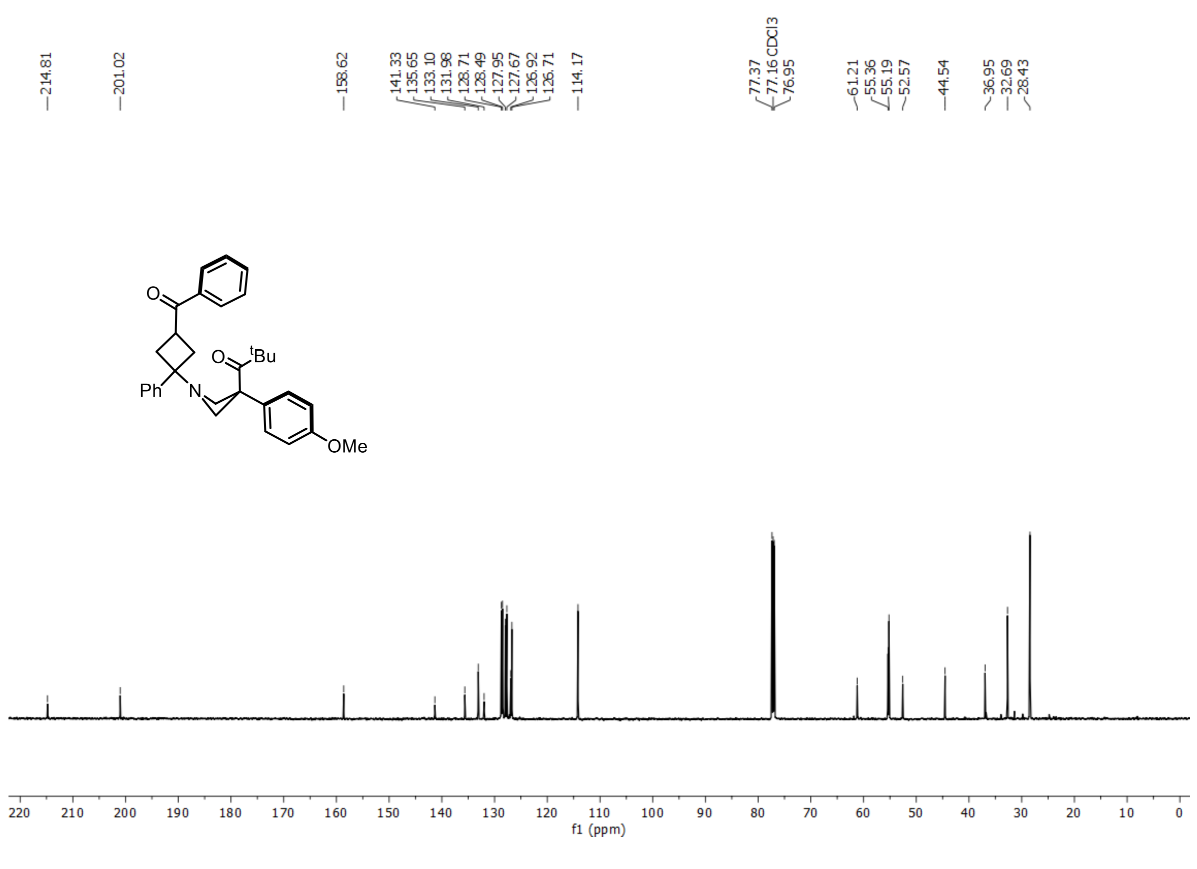

^{1}H spectra at 600 MHz and ^{13}C{^{1}H} NMR spectra at 150 MHz in CDCl3 (**53**)

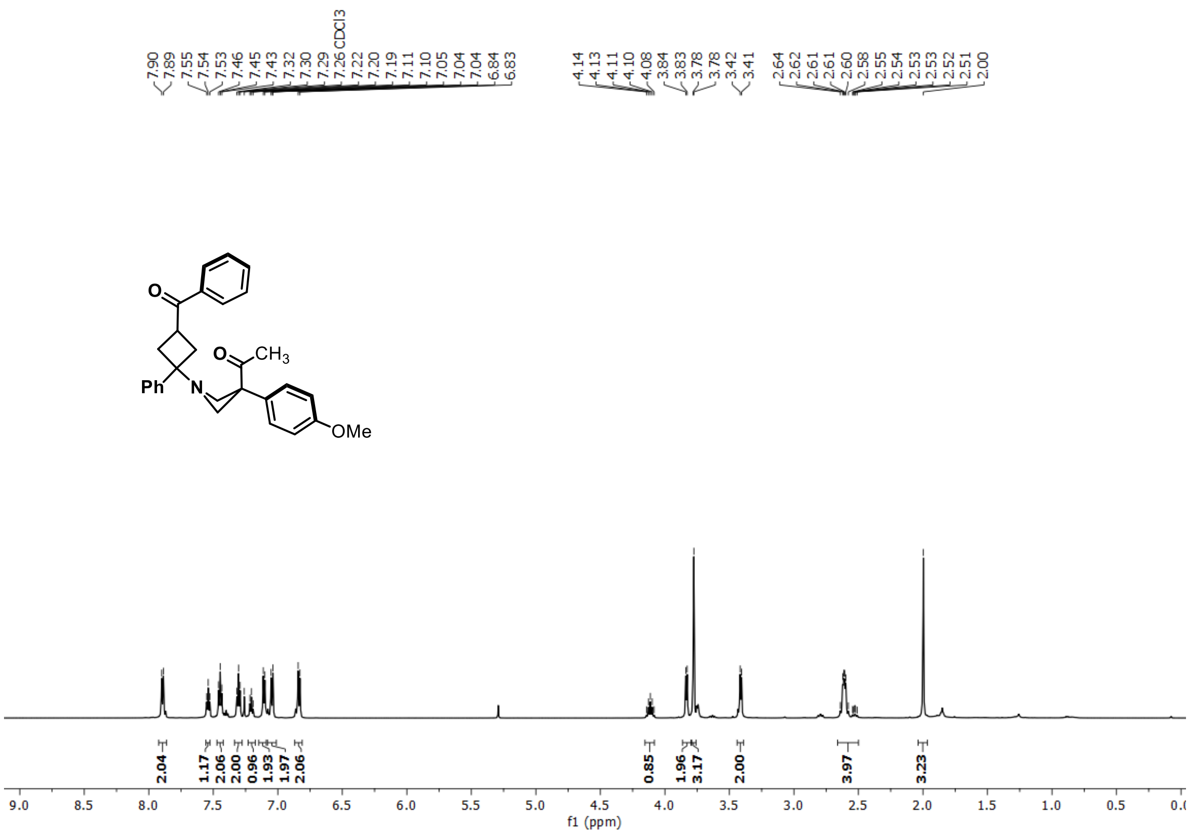

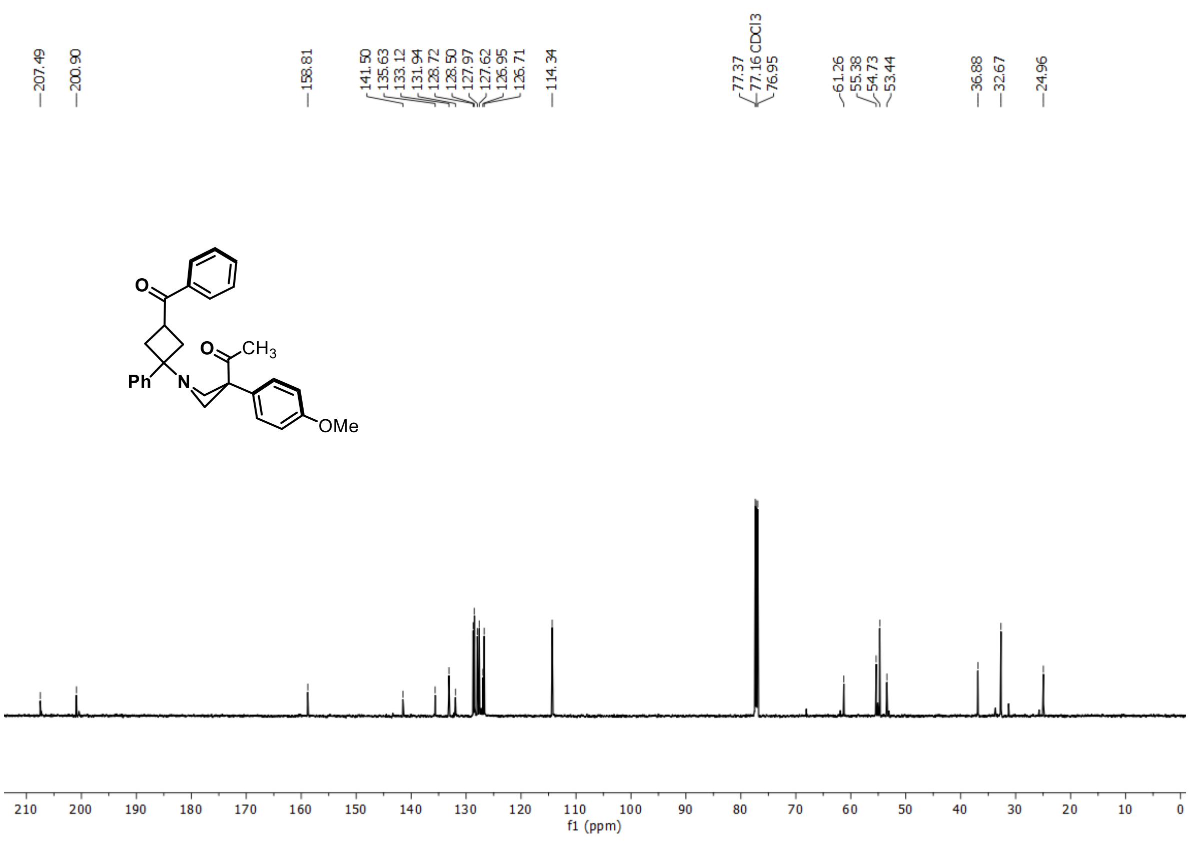

^{1}H spectra at 600 MHz and ^{13}C{^{1}H} NMR spectra at 150 MHz in CDCl3 (**54**)

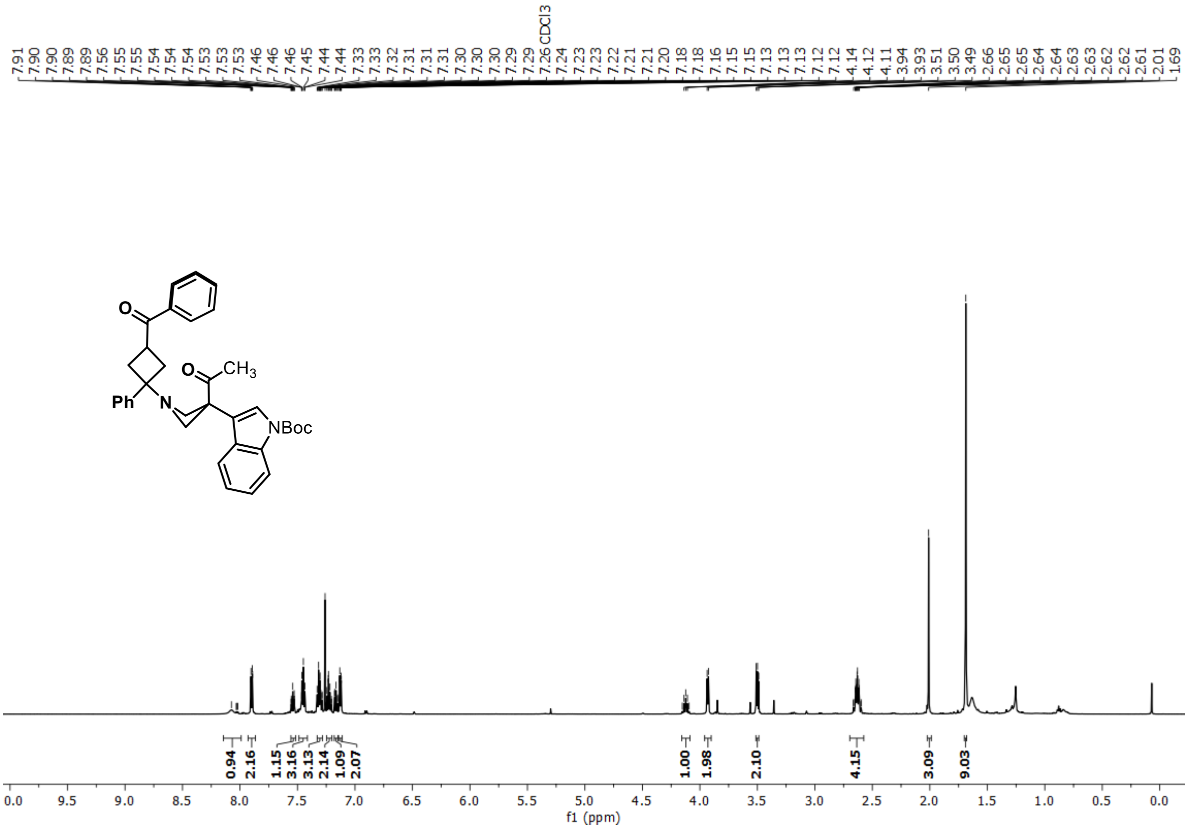

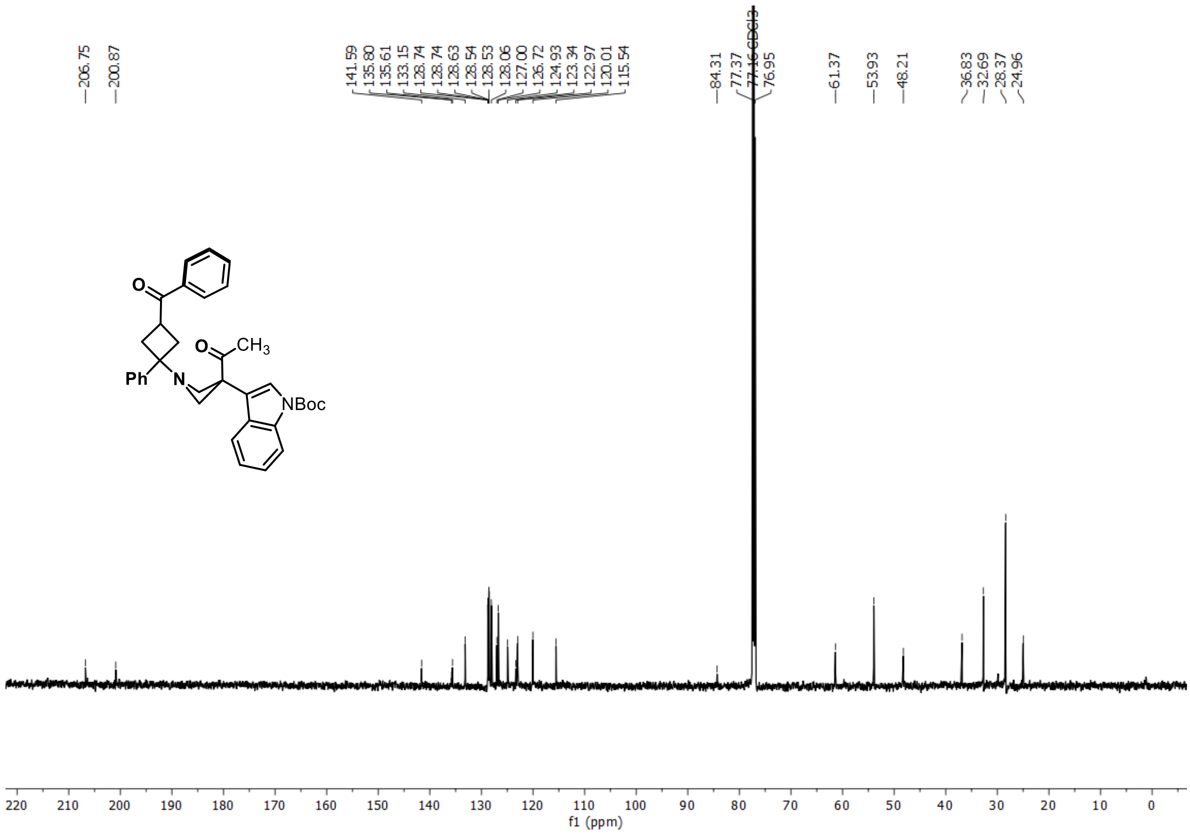

^{1}H spectra at 600 MHz and ^{13}C {^{1}H} NMR spectra at 150 MHz in DMSO-d6 (**55**)

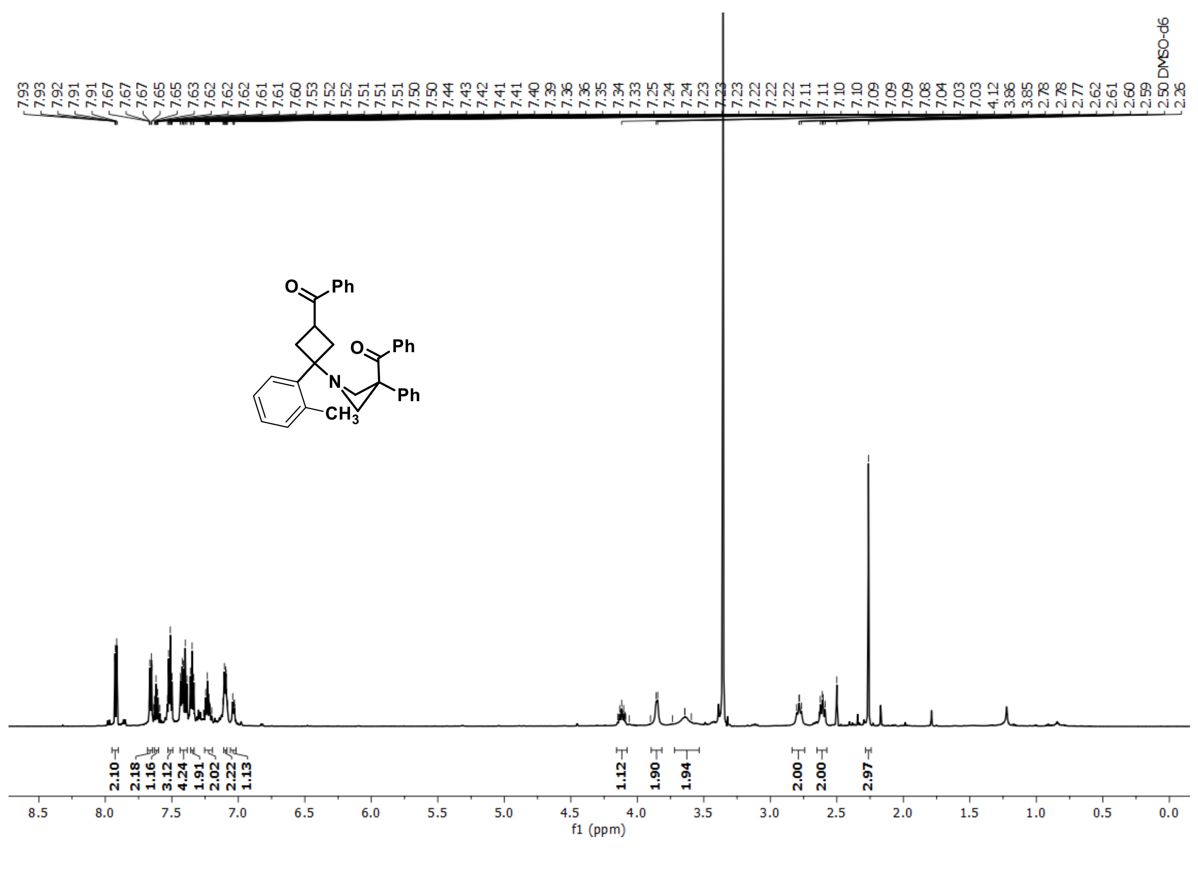

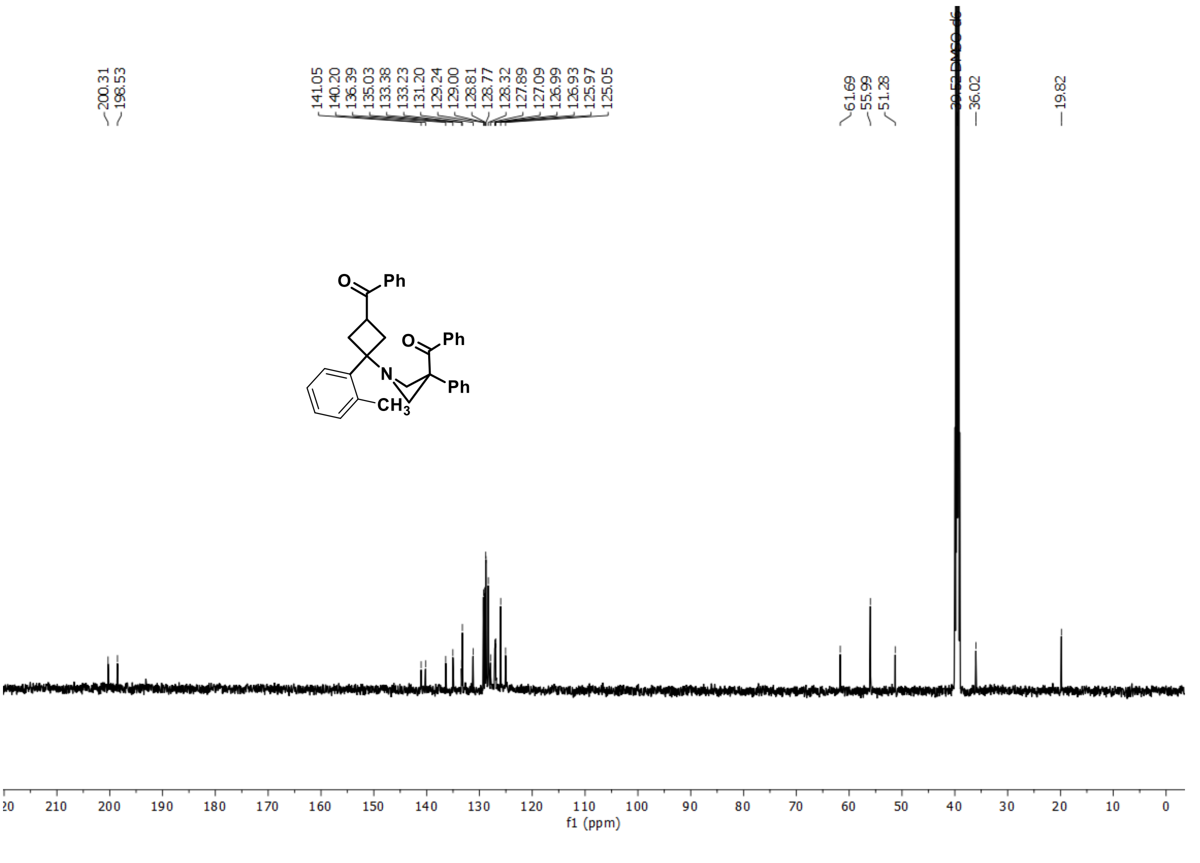

^{1}H spectra at 600 MHz and $^{13}C\{^{1}H\}$ NMR spectra at 150 MHz in CDCl3 (**56**)

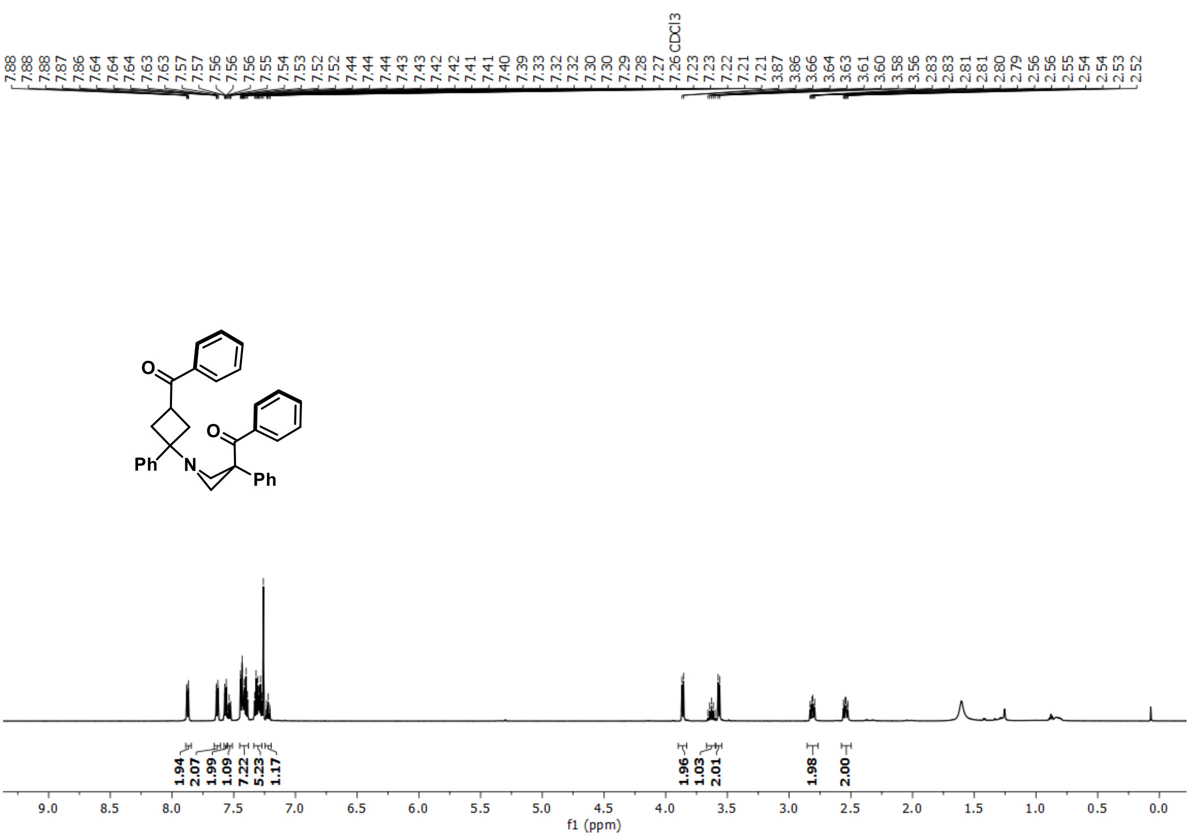

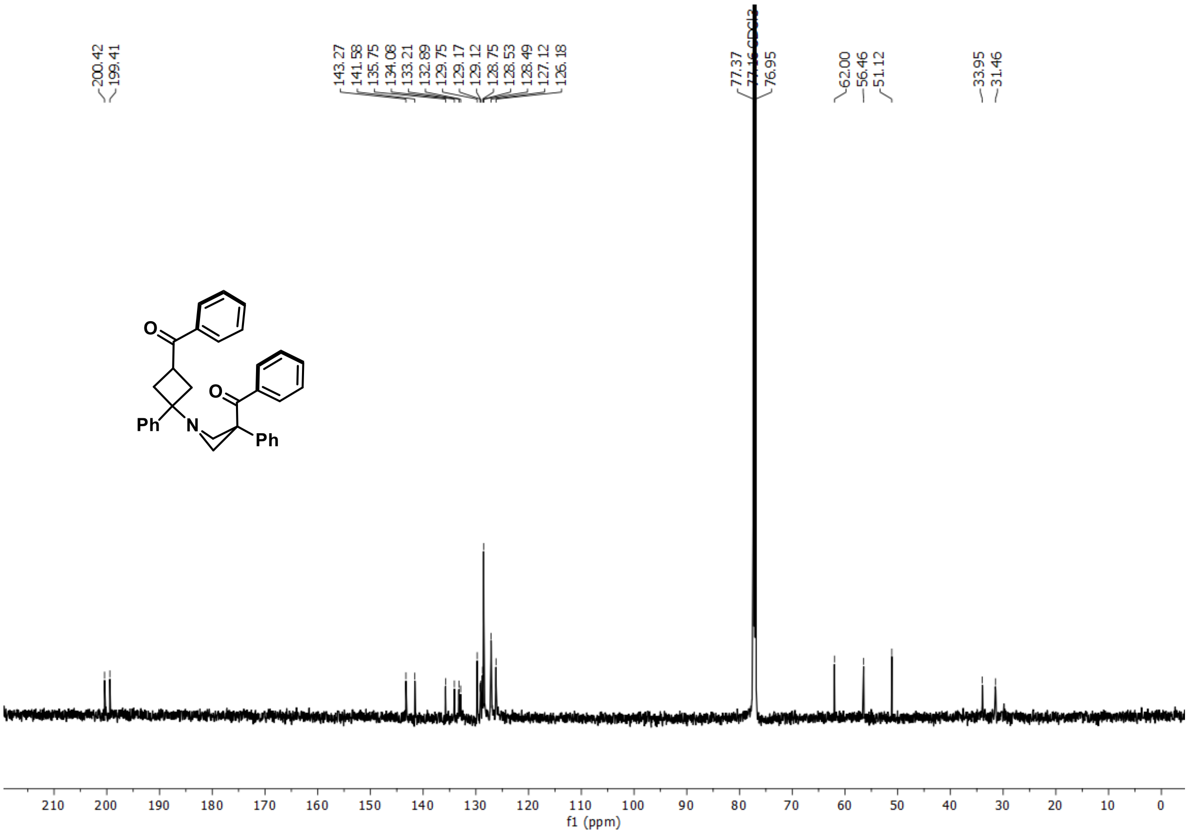

^{1}H spectra at 600 MHz and ^{13}C{^{1}H} NMR spectra at 150 MHz in CDCl3 (**57**)

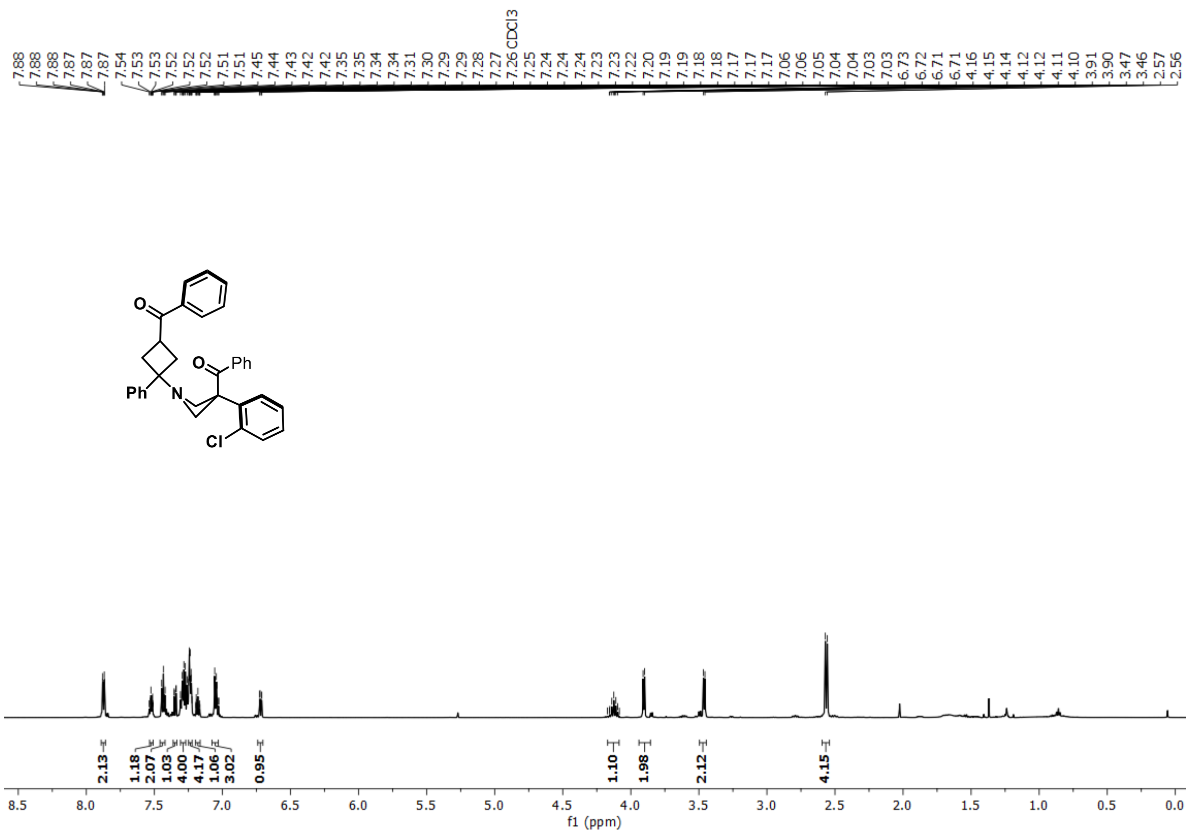

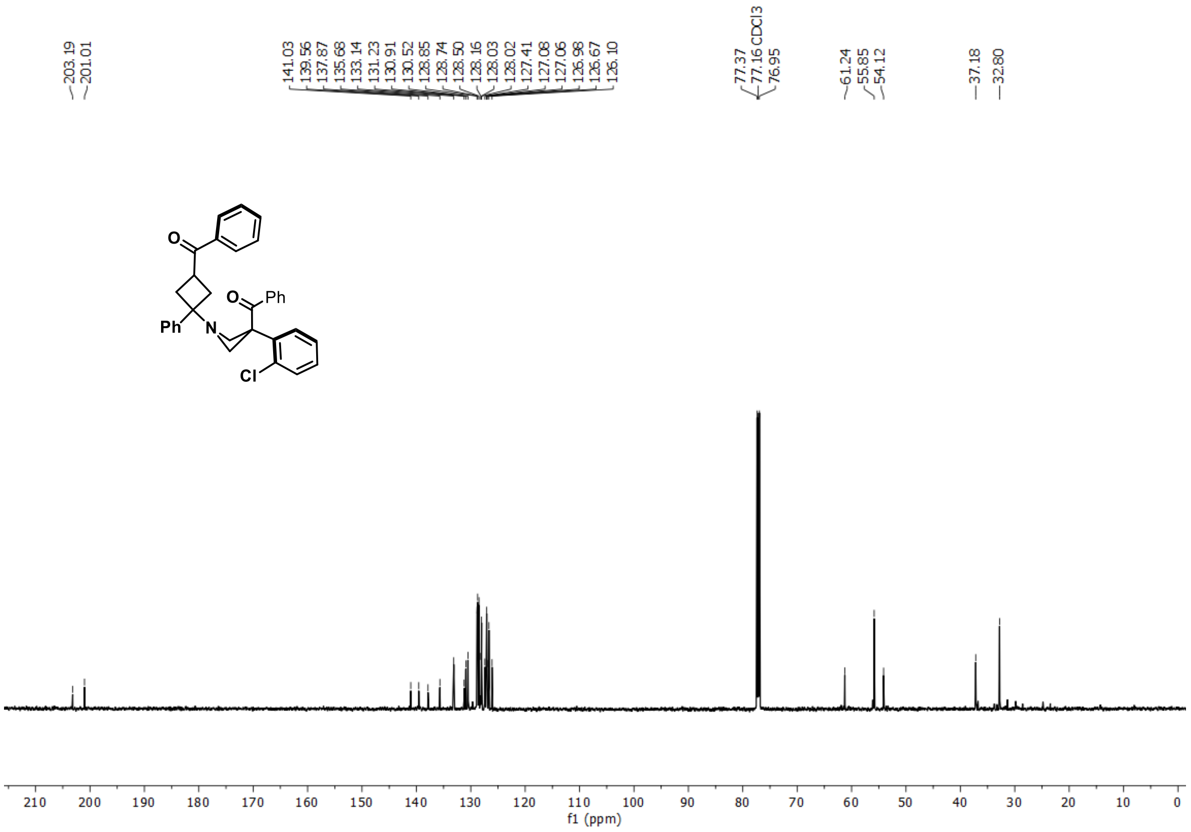

^{1}H spectra at 600 MHz and ^{13}C{^{1}H} NMR spectra at 150 MHz in DMSO-d6 (**58**)

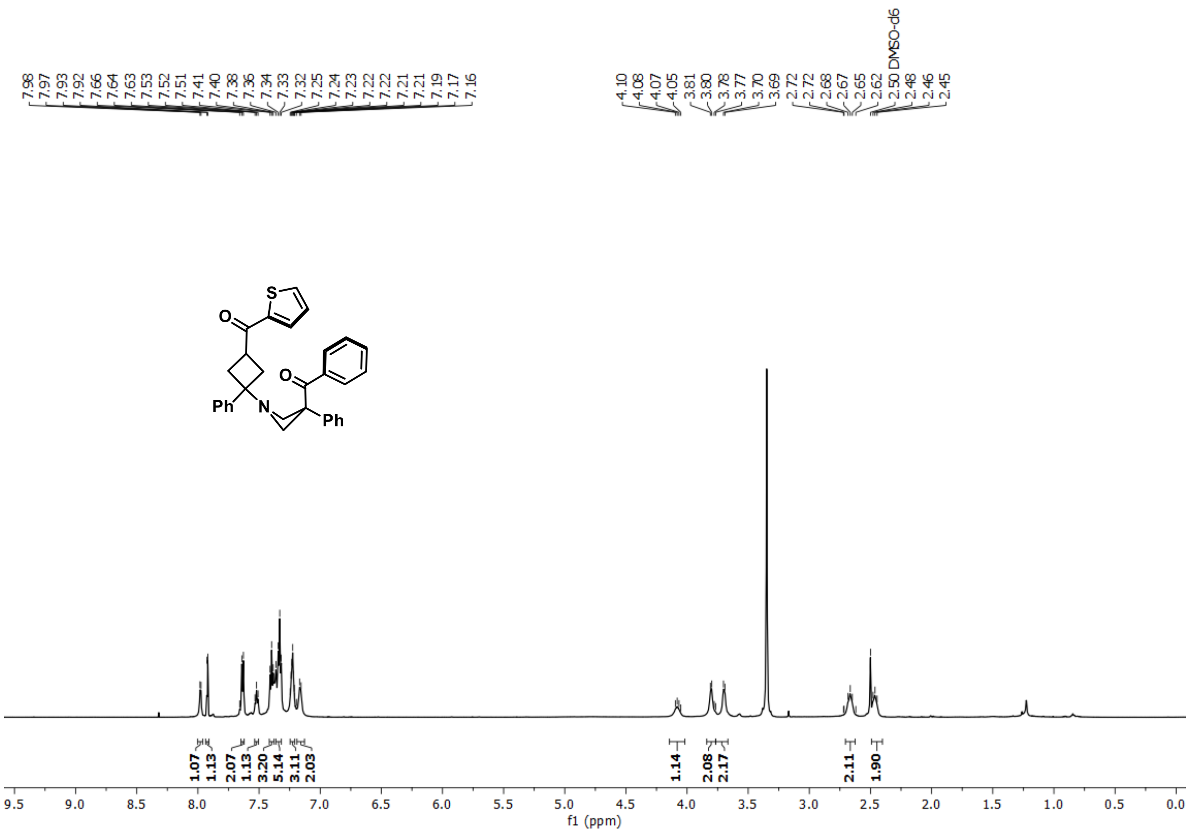

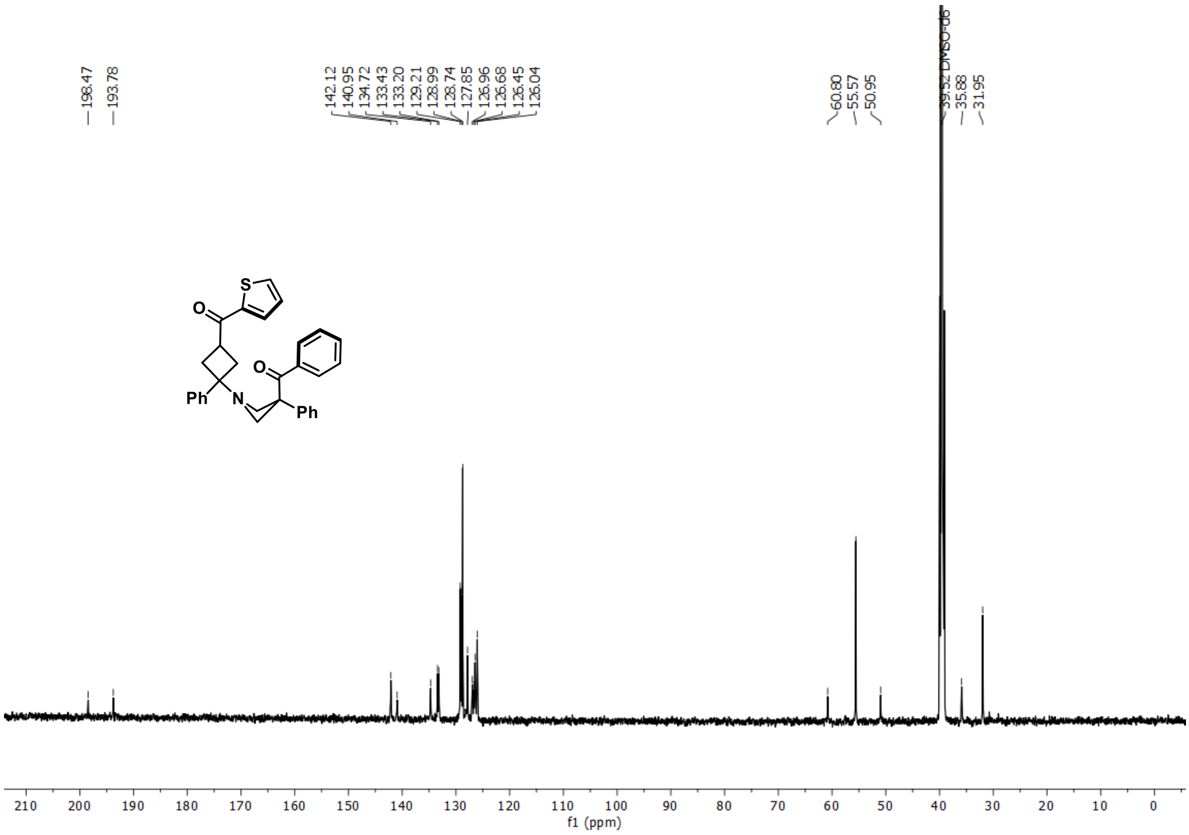

^{1}H spectra at 600 MHz and $^{13}C\{^{1}H\}$ NMR spectra at 150 MHz in CDCl3 (**59**)

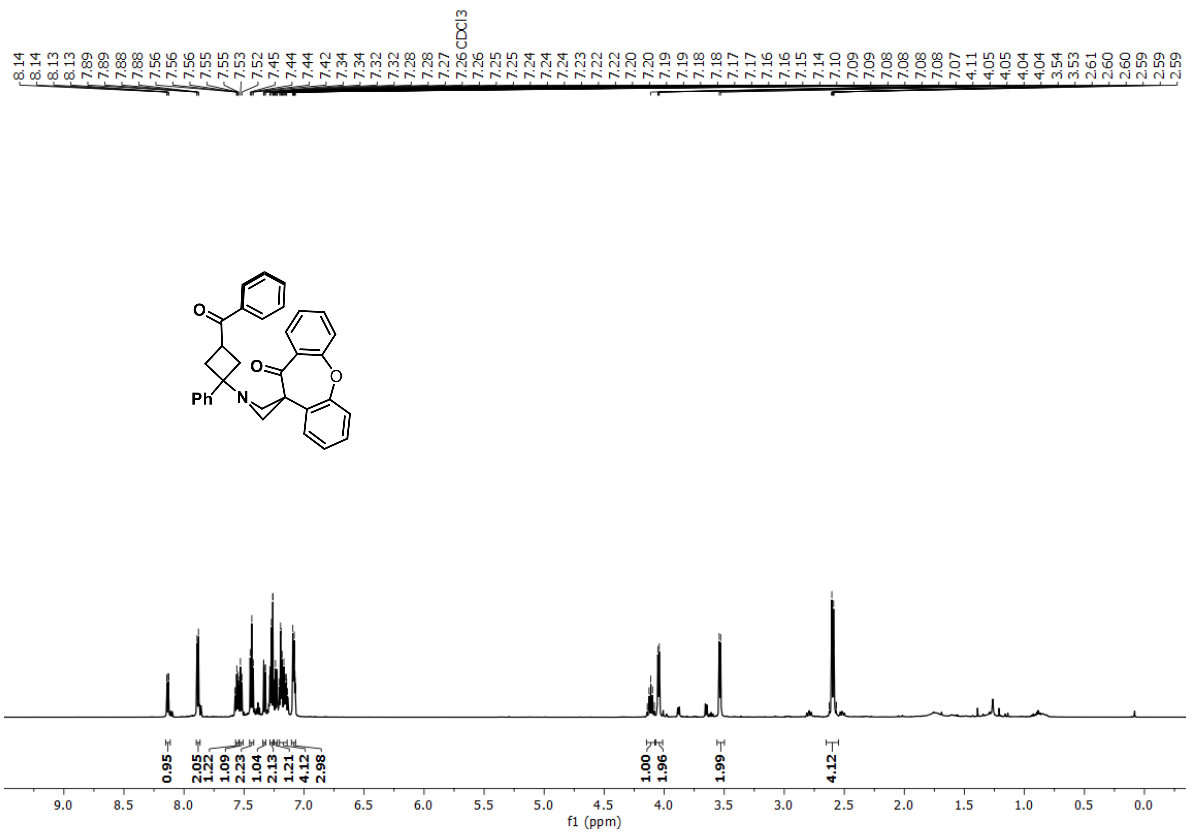

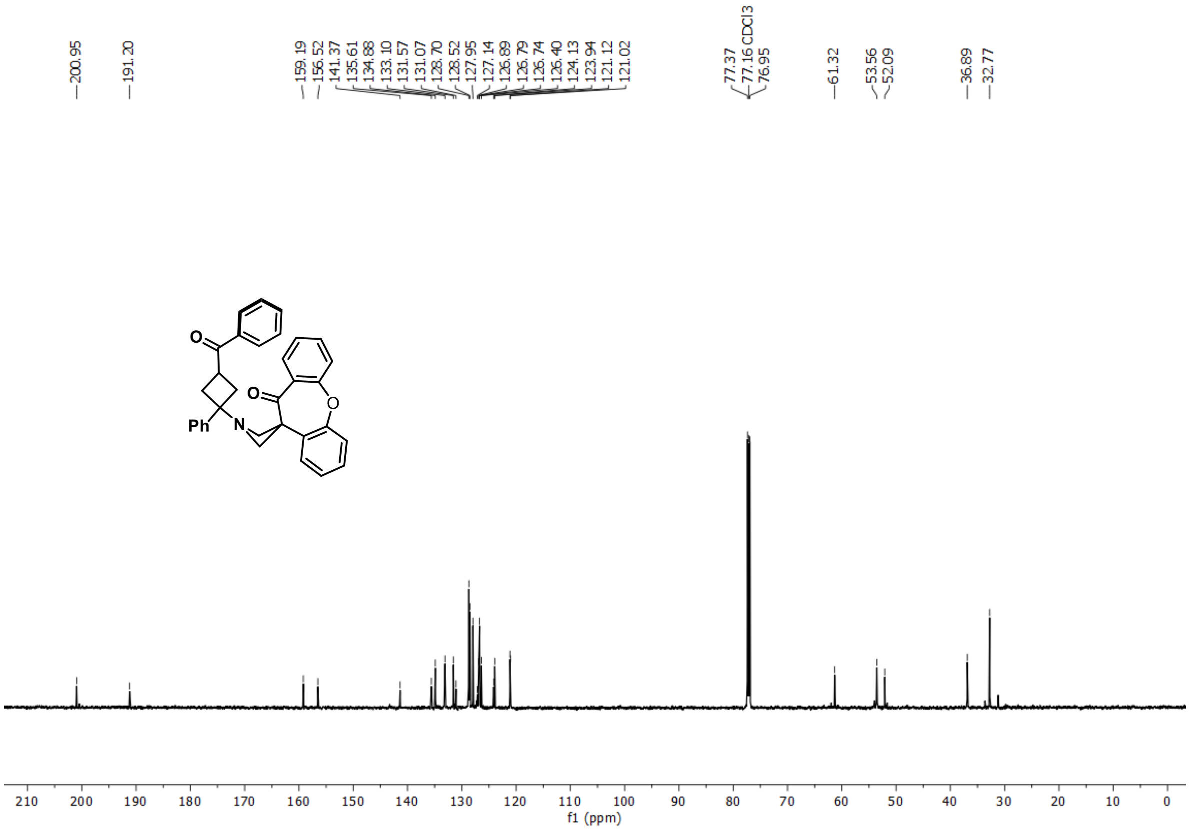

^{1}H spectra at 600 MHz and $^{13}C\{^{1}H\}$ NMR spectra at 150 MHz in CDCl3 (**60**)

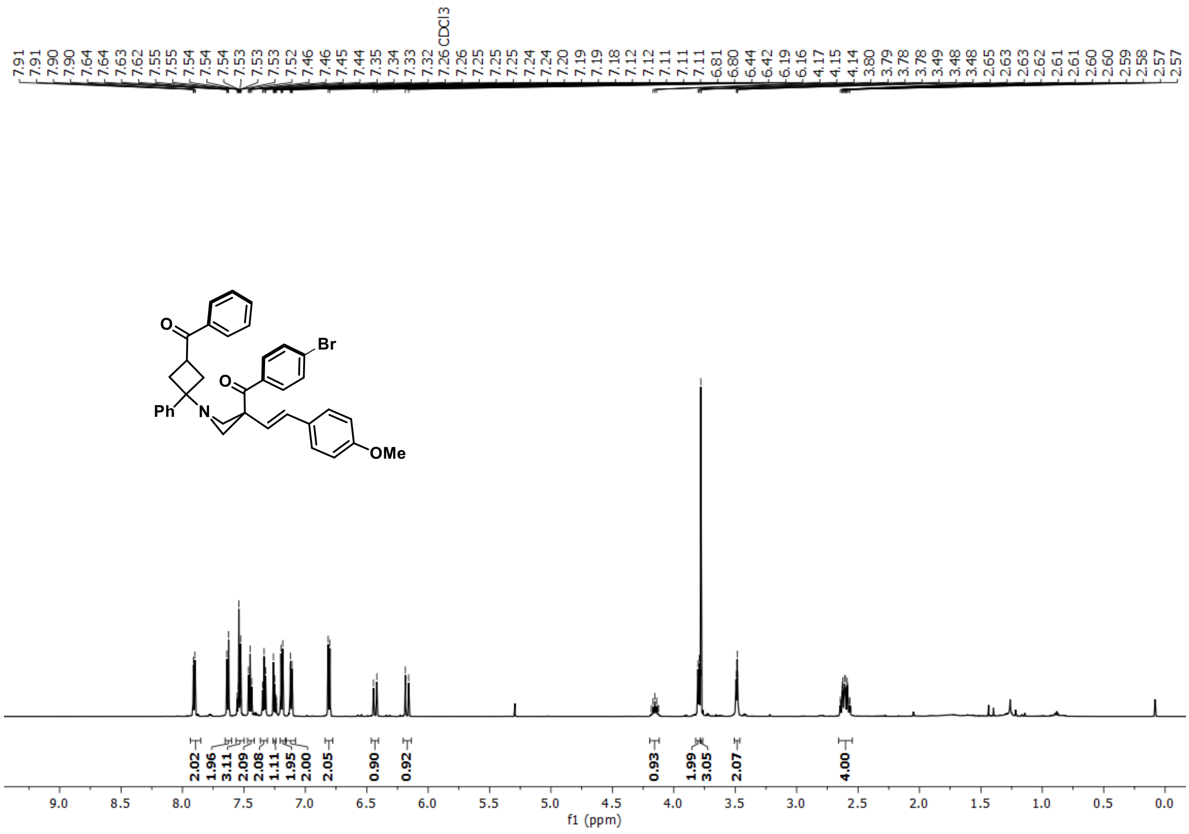

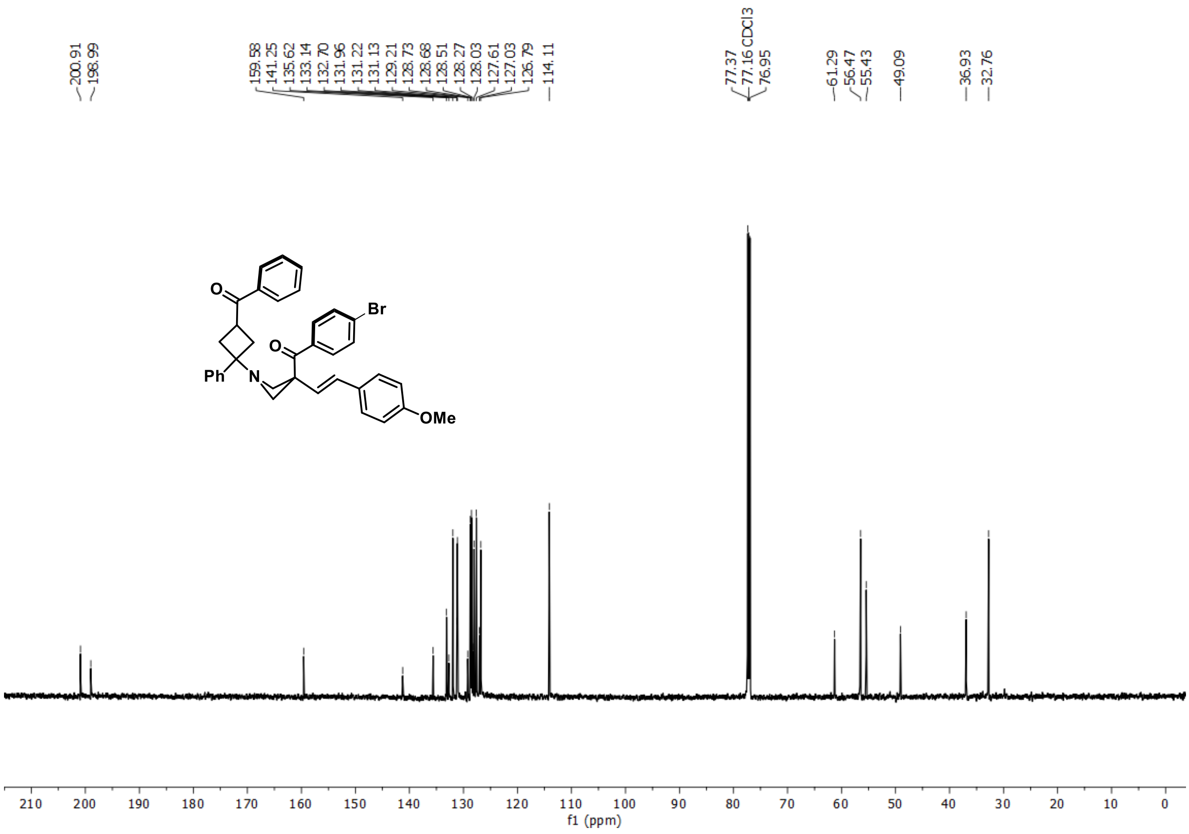

^{1}H spectra at 600 MHz and ^{13}C{^{1}H} NMR spectra at 150 MHz in CDCl3 (**63**)

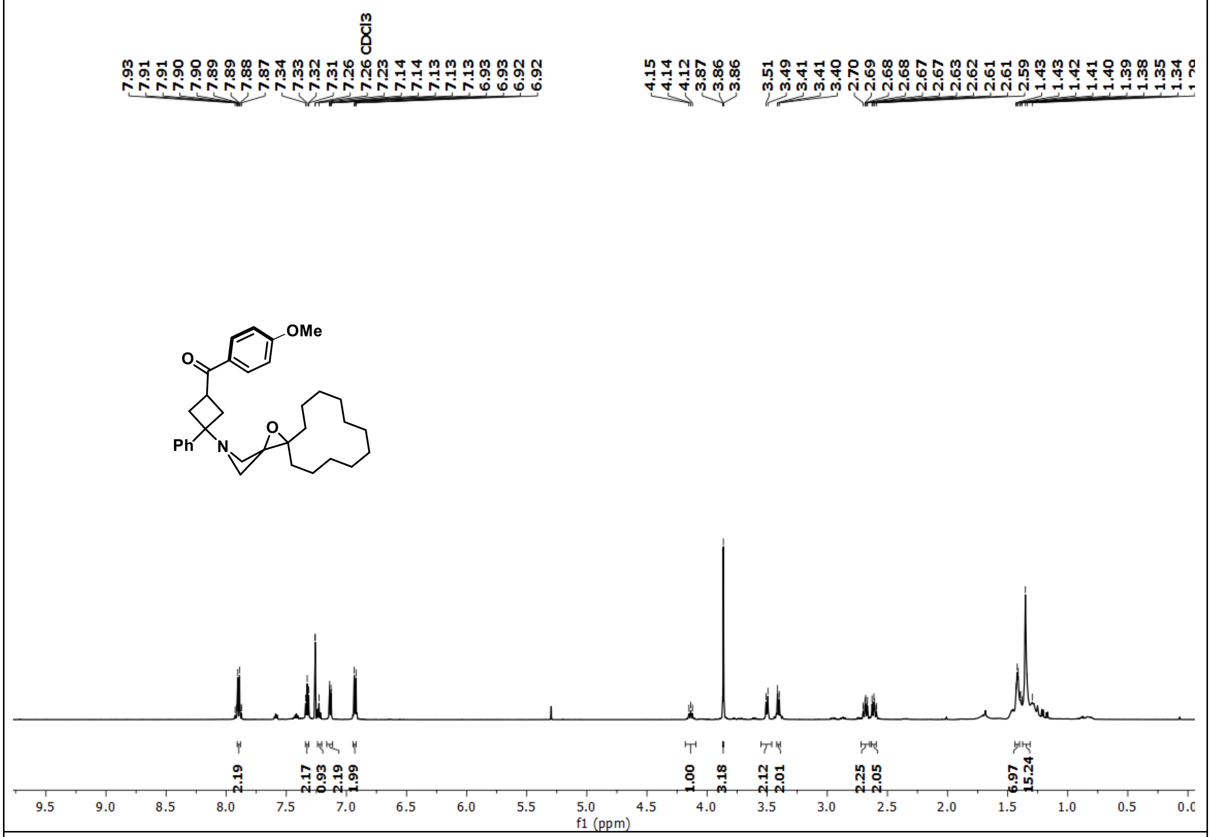

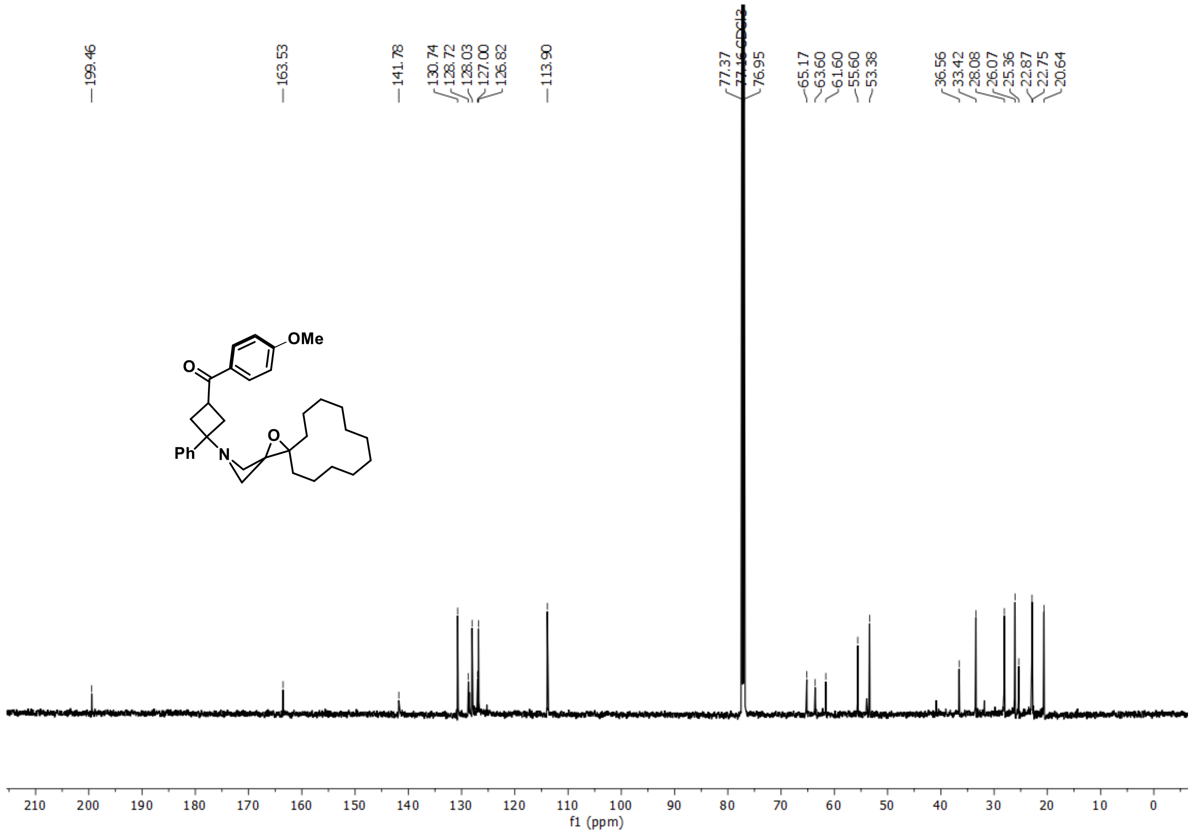

^{1}H spectra at 600 MHz and ^{13}C{^{1}H} NMR spectra at 150 MHz in CDCl3 (**64**)

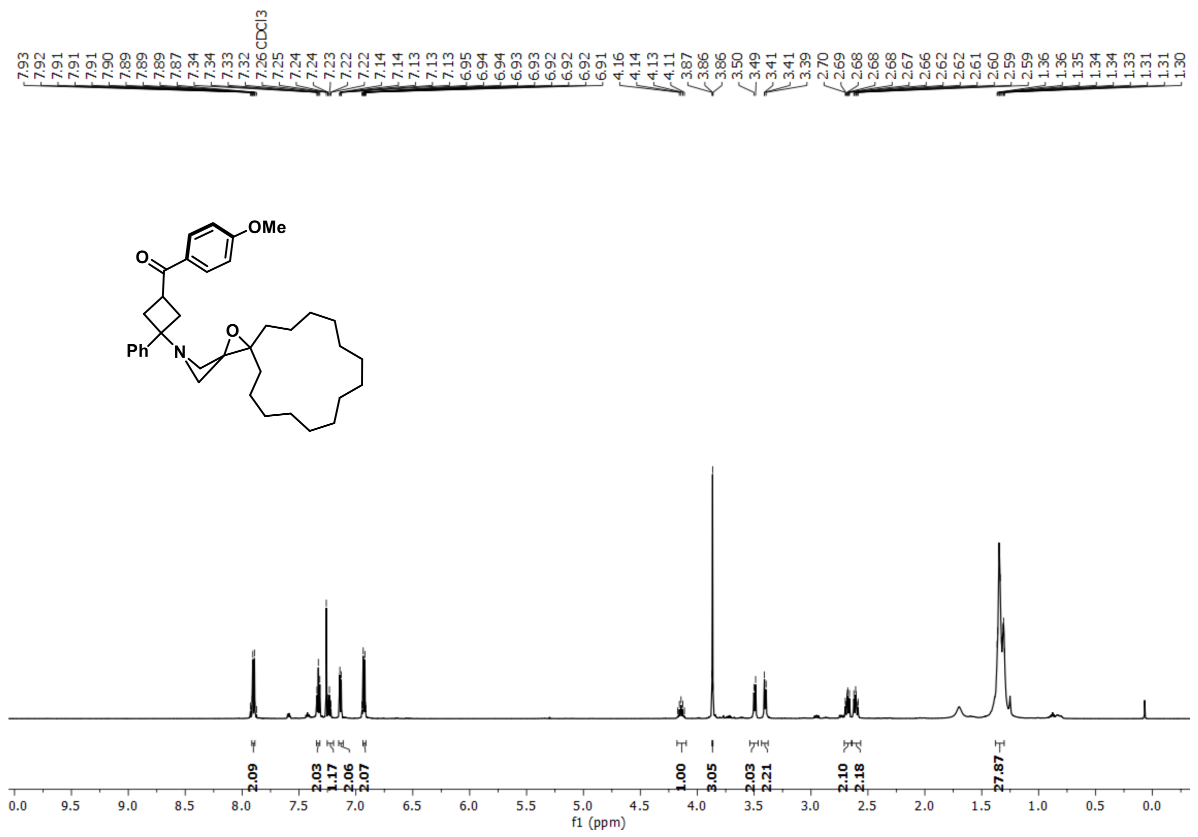

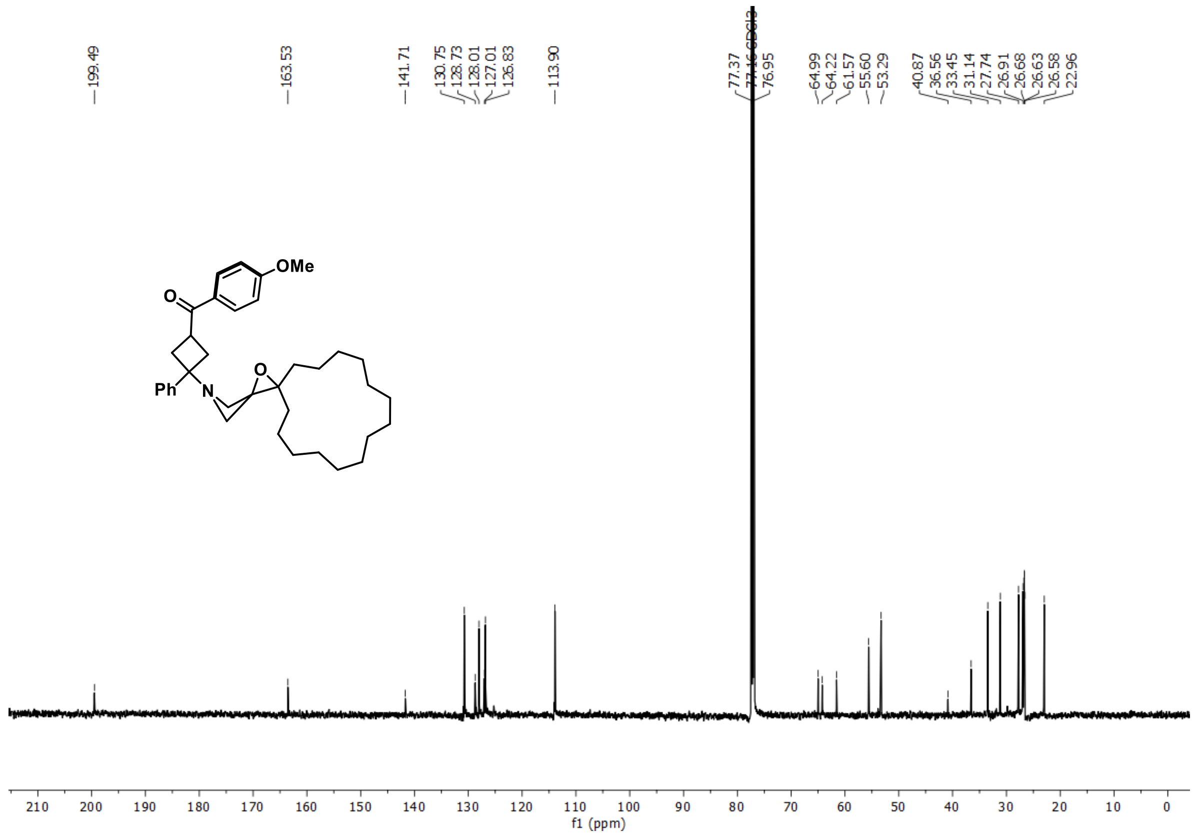

^{1}H spectra at 600 MHz and ^{13}C{^{1}H} NMR spectra at 150 MHz in CDCl3 (**65**)

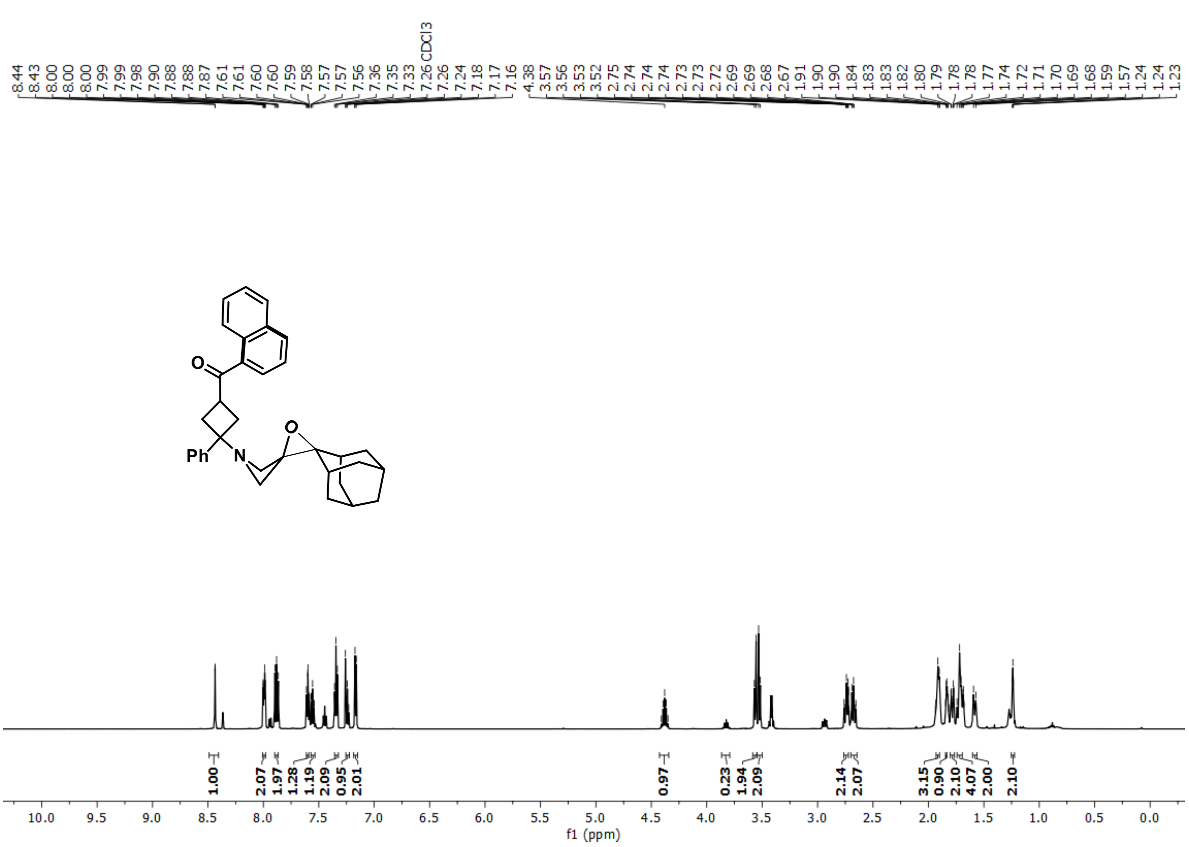

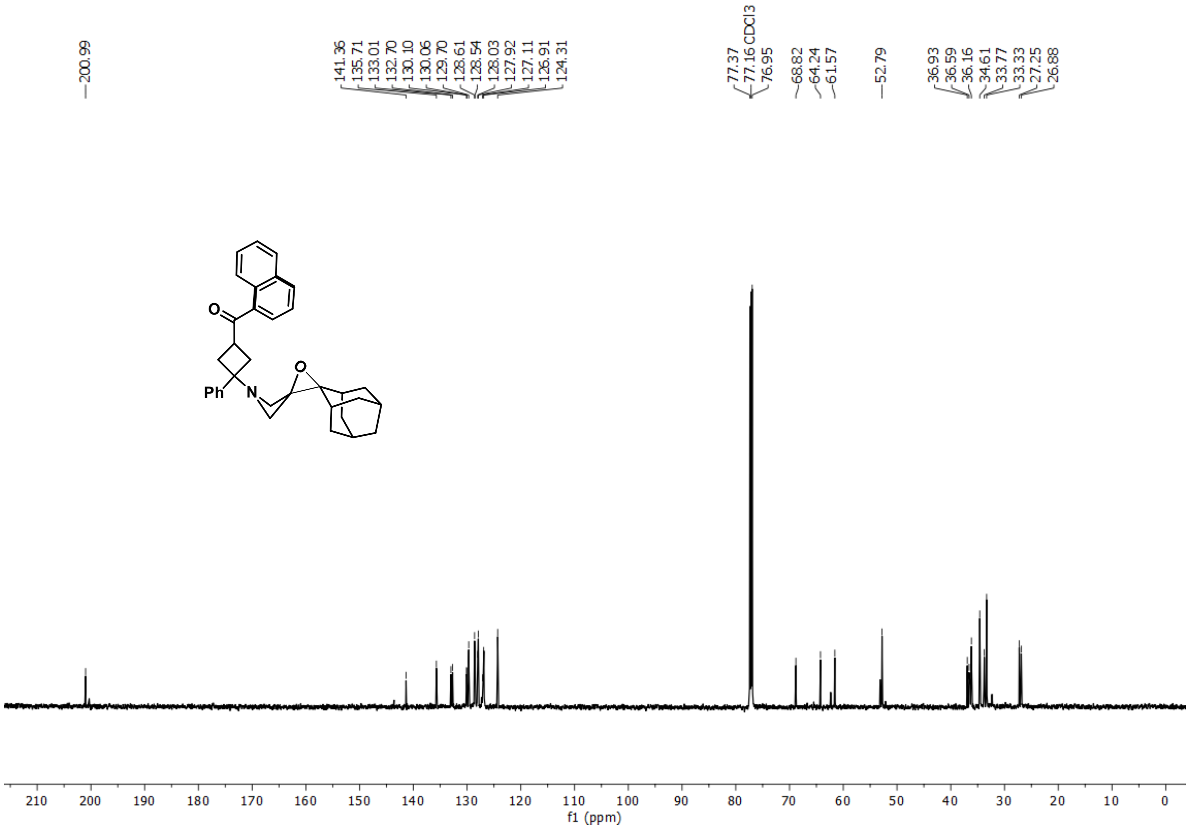

^{1}H spectra at 600 MHz and $^{13}C\{^{1}H\}$ NMR spectra at 150 MHz in CDCl3 (**66**)

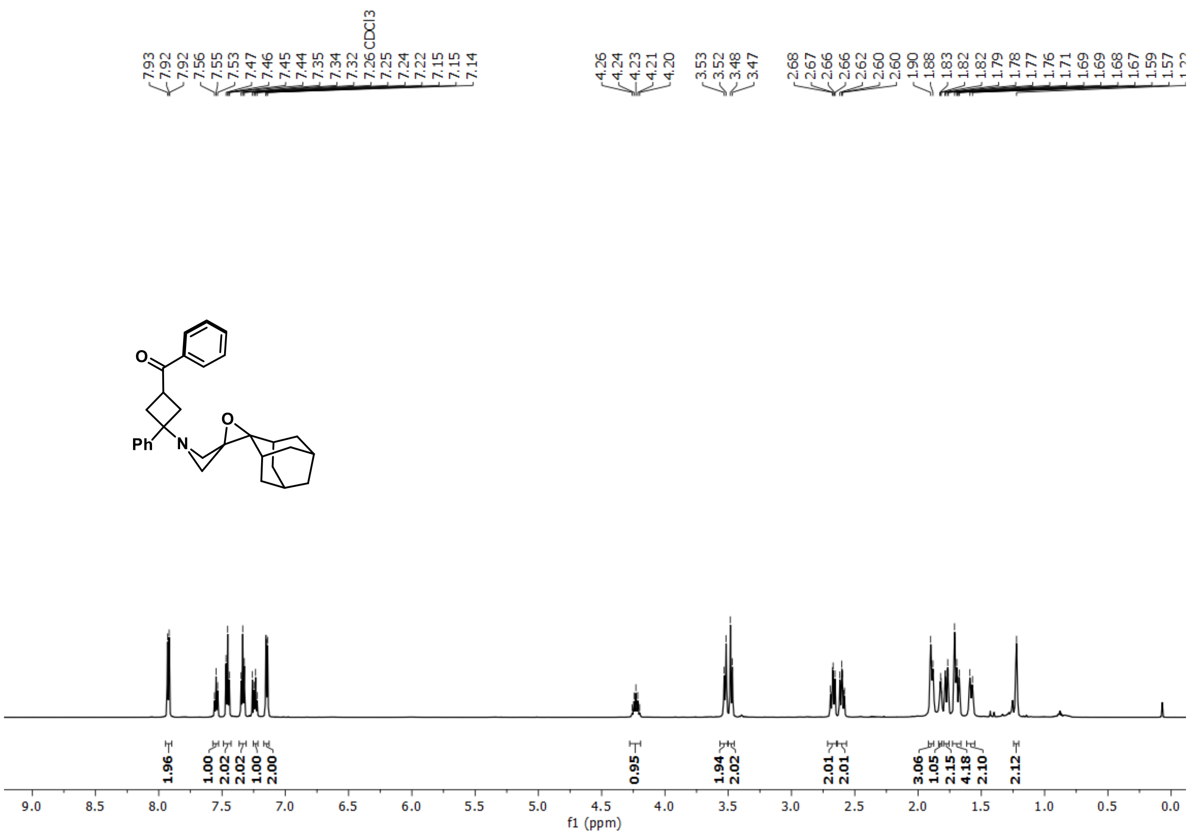

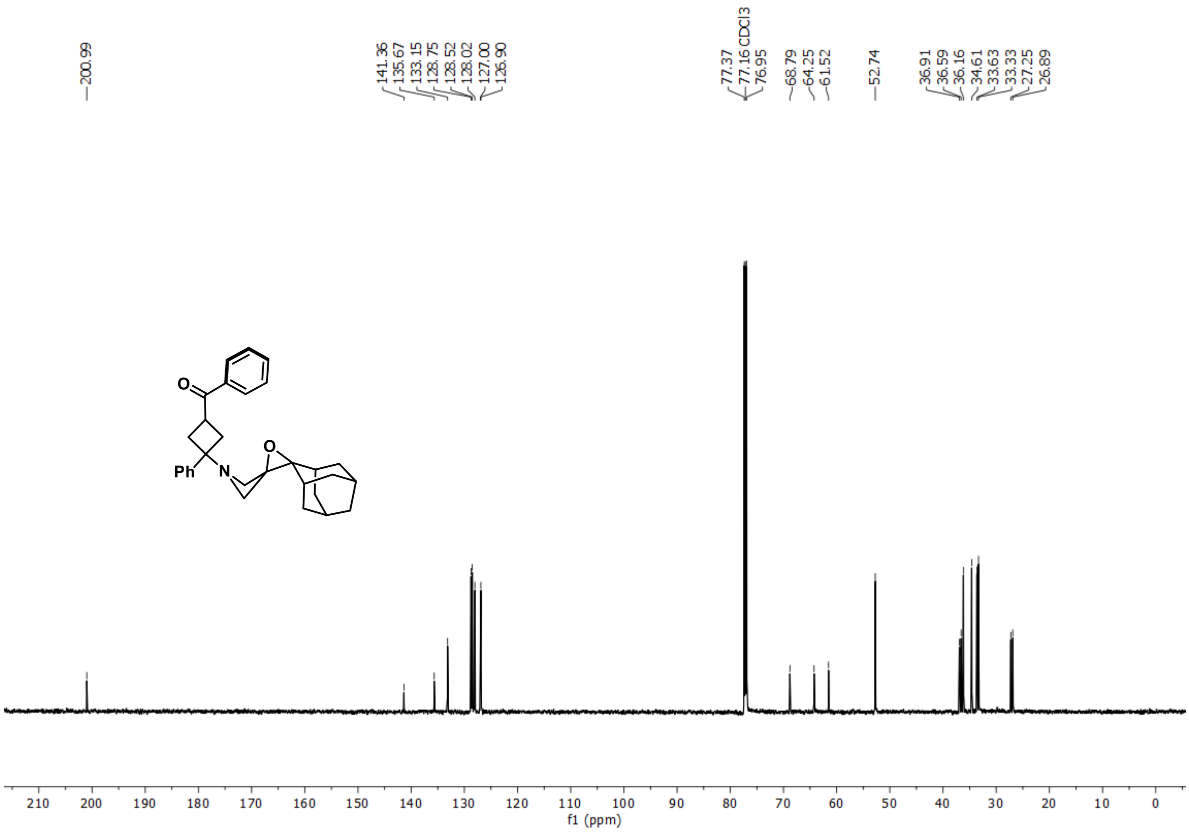

^{1}H spectra at 600 MHz in CDCl3 (**68**)

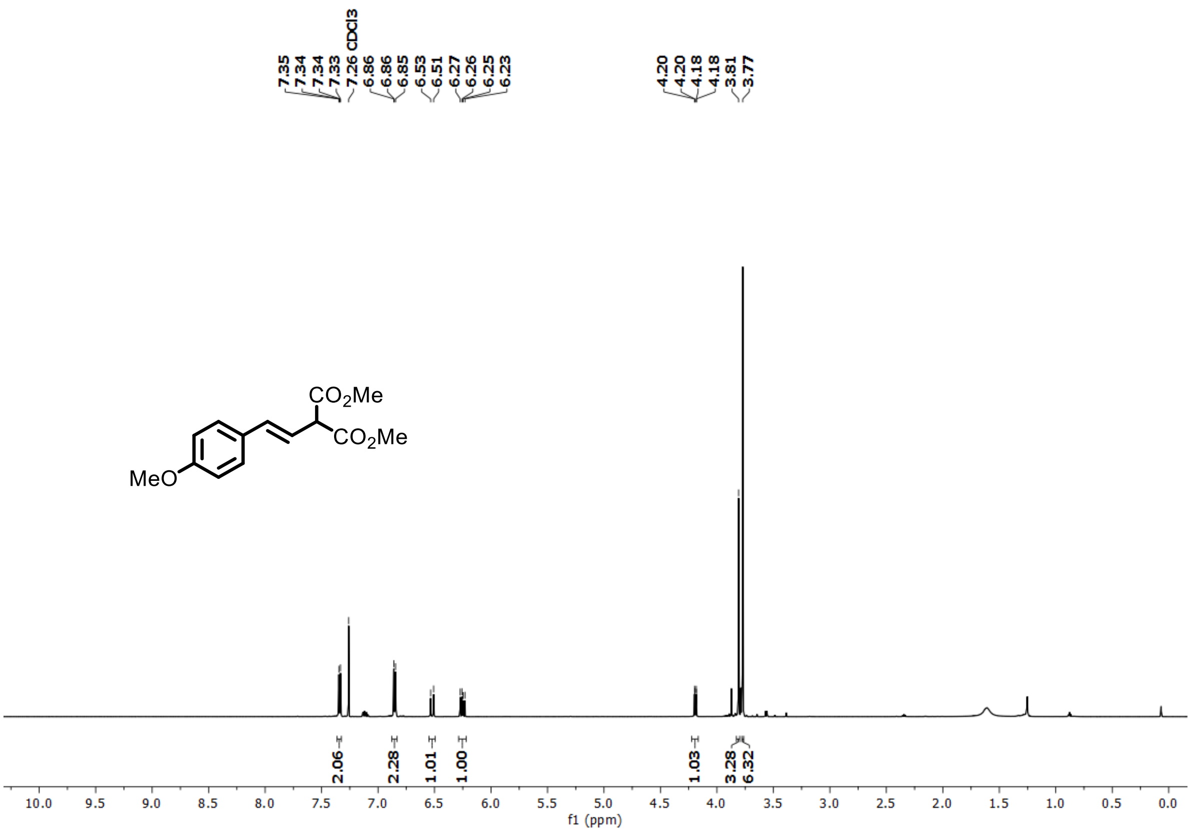

^{1}H spectra at 600 MHz in CDCl3 (**69**)

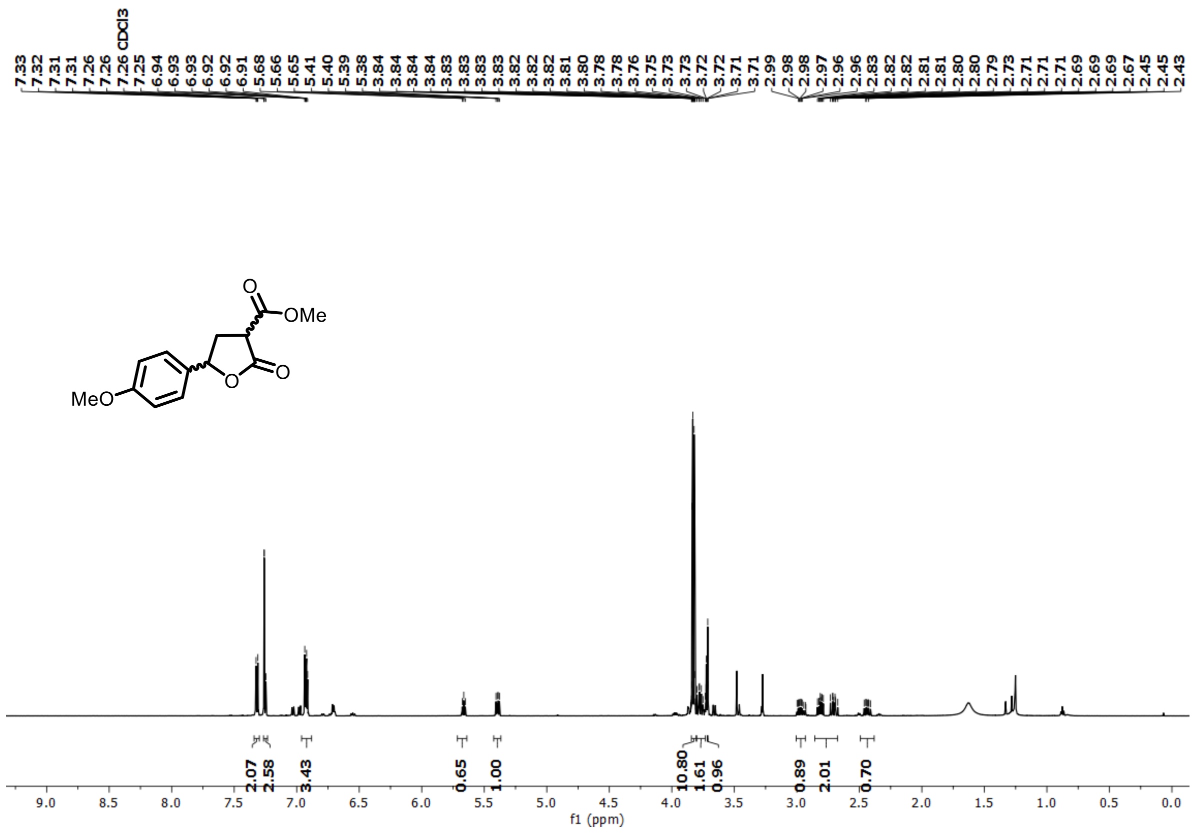

19. Reference

1. (a) B. Mondal, D. Das and J. Saha, *Org. Lett.*, 2020, **22**, 5115–5120; (b) S. R. Cochrane and M. A. Kerr, *Org. Lett.*, 2022, **24,** 5509–5512; (c) M. K. Ghorai, R. Talukdar and D. P. Tiwari, *Org. Lett.*, 2014, **16**, 2204–2207.

2. (a) V. Jaiswal, S. Mondal, B. Singh, V. P. Singh and J. Saha, *Angew. Chem. Int. Ed.*, 2023, **62,** e202304471; (b) B. Singh, P. Sasmal, A. Taites, S. Hazra and J. Saha, *Org. Lett.*, 2024, **26,** 9558–9563; (c) C. H. U. Gregson, A. Noble and V. K. Aggarwal, *Angew. Chem. Int. Ed.*, 2021, **60**, e202100583; (d) M. Zanini, A. Noble and V. K. Aggarwal, *Angew. Chem. Int. Ed.*, 2024, **63**, e202410207; (e) C.-M. Hsu, H.-B. Lin, X.-Z. Hou, R. V. P. P. Tapales, C.-K. Shih, S. Miñoza, Y.-S. Tsai, Z.-N. Tsai, C.-L. Chan and H.-H. Liao, *J. Am. Chem. Soc.*, 2023, **145**, 19049–19059.

3. (a) Agasti, S.; Beltran, F.; Pye, E.; Kaltsoyannis, N.; Crisenza, G. E. M.; Procter, D. J. *Nat. Chem.* 2023, **15**, 535–541. (b) Guo, R.; Chang, Y.-C.; Herter, L.; Salome, C.; Braley, S. E.; Fessard, T. C.; Brown, M. K. *J. Am. Chem. Soc.* 2022, **144**, 7988–7994. (c) Liu, Y.; Lin, S.; Li, Y.; Xue, J.-H.; Li, Q.-J.; Wang, H.-G. *ACS Catal.* 2023, **13**, 5096−5103. (d) Ren, H. S.; Li, T. X.; Xing, J. P.; Li, Z. Y.; Zhang, Y. X.; Yu, X. H.; Zheng, J. *Org. Lett.* 2024, **26***,* 1745–1750.

4. M. Dousset, J.-L. Parrain and G. Chouraqui, *Eur. J. Org. Chem.*, **2017**

5. (a) Zhang, M.; Chen, Y.; Liu, J.; Li, S.; Xu, W.; Qi, J.; Ma, X. Lewis Acid-Catalyzed Ring-Opening Reaction of Bicyclobutanes (BCBs) for the Highly Selective Synthesis of 1,1,3-Trisubstituted Cyclobutanes. *Org. Lett.* **2025**, *27,* 9471–9476. (b) Guin, A.; Deswal, S.; Harariya, M. S.; Biju, A. T. Lewis Acid-Catalyzed Diastereoselective Formal Ene Reaction of Thioindolinones/Thiolactams with Bicyclobutanes. *Chem. Sci.* **2024**, *15,* 12473–12479.

6. (a) Maity, A.; Balanna, K.; Daniliuc, C. G.; Studer, A. Diastereoselective 1,3-nitrooxygenation of bicyclo [1.1.0] butanes. *Chem. Sci*. **2025**, *16*, 7264−7269; (b) Guo, L.; Noble, A.; Aggarwal, V. K. α-Selective Ring-Opening Reactions of Bicyclo[1.1.0]butyl Boronic Ester with Nucleophiles. *Angew. Chem., Int. Ed.* **2021**, *60*, 212−216
